# Evolutionary analysis of TIR- and non-TIR-NBS-LRR disease resistance genes in wild strawberries

**DOI:** 10.3389/fpls.2024.1452251

**Published:** 2024-11-21

**Authors:** Ni Zhu, Yuxi Feng, Guangxin Shi, Qihang Zhang, Bo Yuan, Qin Qiao

**Affiliations:** ^1^ School of Agriculture, Yunnan University, Kunming, China; ^2^ College of Horticulture and Landscape, Yunnan Agricultural University, Kunming, China

**Keywords:** wild strawberry, NLR gene family, evolution, *Botrytis cinerea*, bioinformatics

## Abstract

**Introduction:**

NBS-LRR genes (NLRs) are the most extensive category of plant resistance genes (R genes) and play a crucial role in pathogen defense. Understanding the diversity and evolutionary dynamics of NLRs in different plant species is essential for improving disease resistance. This study investigates the NLR gene family in eight diploid wild strawberry species to explore their structural characteristics, evolutionary relationships, and potential for enhancing disease resistance.

**Methods:**

We conducted a comprehensive genome-wide identification and structural analysis of NLRs across eight diploid wild strawberry species. Phylogenetic analysis was performed to examine the relationships between TIR-NLRs (TNLs), Non-TIR-NLRs (non-TNLs), CC-NLRs (CNLs), and RPW8-NLRs (RNLs). Gene structures were compared, and gene expression was profiled across different NLR subfamilies. Additionally, in vitro leaf inoculation assays with *Botrytis cinerea* were performed to assess the resistance of various strawberry species.

**Results:**

Our analysis revealed that non-TNLs constitute over 50% of the NLR gene family in all eight strawberry species, surpassing the proportion of TNLs. Phylogenetic analysis showed that TNLs diverged into two subclades: one grouping with CNLs and the other closely related to RNLs. A significantly higher number of non-TNLs were under positive selection compared to TNLs, indicating their rapid diversification. Gene structure analysis demonstrated that non-TNLs have shorter gene structures than TNLs and exhibit higher expression levels, particularly RNLs. Notably, non-TNLs showed dominant expression under both normal and infected conditions. *In vitro* leaf inoculation assays revealed that *Fragaria pentaphylla* and *Fragaria nilgerrensis*, which have the highest proportion of non-TNLs, exhibited significantly greater resistance to *Botrytis cinerea* compared to *Fragaria vesca*, which has the lowest proportion of non-TNLs.

**Discussion:**

The findings of this study provide important insights into the evolutionary dynamics of NLRs in strawberries, particularly the significant role of non-TNLs in pathogen defense. The rapid diversification and higher expression levels of non-TNLs suggest their potential contribution to enhanced disease resistance. This research highlights the value of non-TNLs in strawberry breeding programs aimed at improving resistance to pathogens such as *Botrytis cinerea*.

## 1 Introduction

Enhancing plant innate disease resistance is crucial for effective and sustainable agriculture. Throughout the process of evolution, one of the key drivers for plants to activate innate immunity is their interaction with pathogens. Many plants have evolved specific genes to combat adverse conditions. Among these, the NLR gene family is notable for being the largest and most functionally significant group of disease-resistant genes in the plant kingdom (Liu et al., 2021).

Classic NLRs are generally divided into three principal subfamilies: TIR-NBS-LRR (TNL), CC-NBS-LRR (CNL), and RPW8-NBS-LRR (RNL). These subfamilies are distinguished by the presence of the Toll/interleukin-1 receptor (TIR) domain at the variable N-terminus, thereby classifying them into TNL and non-TNL types. Additionally, they can be functionally categorized into sensors (TNL and CNL), which are responsible for binding to or recognizing effectors, and helpers (RNL), which are activated by other NLRs or recognize effectors, thereby initiating downstream signaling cascades (Tamborski and Krasileva, 2020). Regardless of the classification method used, there are always significant evolutionary differences in NLRs among different species, such as *Arabidopsis thaliana*, *Oryza sativa*, *Sorghum bicolor*, *Malus x domestica*, *Glycine max*, and *Solanum tuberosum* (Akira et al., 2003; Kang et al., 2012; Arya et al., 2014; Yang and Wang, 2016; Hang et al., 2018). It has been revealed that the lineage of both TNLs and non-TNLs can be traced back to the most ancient group of land plants, bryophytes (Neupane et al., 2018). However, the proportion of different types of NLRs varies significantly among plant species. And TNLs are present only in dicotyledonous plants, while they are absent in monocotyledonous plants (Tarr et al., 2009). These differences likely arise from variations in life histories among species, coupled with exposure to diverse pathogenic environments, result in the formation of distinct evolutionary patterns within the NLR gene family. Therefore, investigating the evolutionary dynamics of NLRs across different species not only enhances our understanding of the evolution of the NLR gene family but also augments our comprehension and application of the plant innate immune system.

Previous studies on the evolution of NLRs in five Rosaceae species have shown that, compared to non-TNLs, TNLs exhibit faster evolutionary rates and stronger selective pressures in four out of five species, with *Fragaria vesca* being the exception, as it demonstrates a higher Ka/Ks ratio for non-TNLs (Zhong et al., 2015). Further research into specific *NLR* duplications in six different polyploid strawberry species has revealed a general trend of higher Ka/Ks ratios for TNLs compared to non-TNLs (Zhong et al., 2018). This difference in selective pressure is likely due to variations in species ploidy. Consequently, this study delves into eight diploid strawberry species to explore the evolutionary patterns of various *NLR* gene types within wild strawberries that share the same ploidy status.

Furthermore, in practical agricultural settings, cultivated strawberries (*Fragaria x ananassa*, 2n=8x=56) are often threatened by diseases such as gray mold, powdery mildew, and fruit rot, resulting in severe economic losses (Wu and Kamoun, 2021). Conversely, wild strawberries, which grow in diverse natural environments, generally exhibit enhanced disease resistance (Lei et al., 2017). This trait provides valuable resources for improving disease resistance in cultivated species. This study concentrates on wild strawberries, with a specific focus on investigating *NLR* disease resistance genes, and aims to offer effective strategies for breeding disease-resistant strawberry cultivars.

## 2 Materials and methods

### 2.1 Identification and classification of NLRs

The complete genome sequences and annotations of *F. mandschurica*, *F. daltoniana*, *F. pentaphylla*, *F. nilgerrensis*, *F. viridis*, *F. vesca*, *F. iinumae*, and *F. nubicola* were acquired from the Genome Database for *Rosaceae* (GDR, https://www.rosaceae.org/). In cases where genes had multiple transcripts, only the longest transcript was preserved for subsequent analysis. The proteomes were extensively explored for potential NB-ARC domain through HMMER v3.1 with an e-value cutoff of < 1, employing the NB-ARC (PF00931) Hidden Markov models (HMMs) obtained from Pfam (Finn et al., 2015). The NB-ARC (also known as NBS) seed sequences were initially acquired from the Pfam website as the query sequence for BLASTP against the entire genome with an expectation value ≤ 1e^-2^ across the eight strawberry species. Subsequently, all hits from both BLAST and HMM searches were consolidated, and redundant entries were removed.

All NB-ARC genes were further analyzed, the LRR (PF00560, PF18805, PF18831, PF18837, PF07723, PF07725, PF12799, PF13306, PF13516, PF13855, PF14580, PF01462, PF08263), TIR (PF01582) and RPW8 (PF05659) domains were detected by same analysis. The CC domain was predicted by the COILS command line with a threshold of 0.1 (Lupas et al., 1991; Lupas, 1996). Finally, in order to improve the accuracy of the putative predictions, all sequences were analyzed to further verify the presence of domains using CD-search tool (https://www.ncbi.nlm.nih.gov/Structure/bwrpsb/bwrpsb.cgi) and SMART (https://smart.embl.de/).

### 2.2 Chromosomal localization and gene cluster analysis of the NLR gene family

The TBtools was used to map all identified NLRs onto seven chromosomes based on their physical positions indicated in the genome database. Several genes were organized in diverse *NLR* clusters, in which at least two NLRs were localized in closer than 200 kb region and were separated by a maximum of eight non-NLRs (Erik et al., 2002; Chen et al., 2018).

### 2.3 Alignment and phylogenetic tree construction

The multiple alignment was performed using MAFFT v7 on NB-ARC domain sequences of NLRs under default parameters (Katoh et al., 2002). The resulting alignment was trimmed using TrimAl (Capella-Gutiérrez et al., 2009). Maximum Likelihood (ML) phylogenetic analyses of NLRs was performed using IQ-TREE v1.6.12, with branch supports reported as 1000 Ultrafast Bootstraps (UFBoot) (Lam-Tung et al., 2015). The most optimal model of aa evolution was selected by ModelFinder within IQ-TREE (Kalyaanamoorthy et al., 2017), the best scoring tree was used in all discussions. The tree was visualized using iTOL v6 (https://itol.embl.de/) (Bork, 2007). The same methods were also used to construct another three phylogenetic trees of the TNL, CNL and RNL families, respectively. Furthermore, to restore the NLRs duplication and loss events that occurred during evolution, the reconstructed TNLs and non-TNLs trees were compared with the real species tree lineage by lineage, using the Notung software (Maureen et al., 2012). The exogroup is the *NBS-LRR* gene of *Rosa indica* (GenBank: MK689860.1).

### 2.4 Gene structures, conserved motif and biological analysis of NLRs

The exon/intron structure of the NLRs was retrieved from the general feature format (GFF) file of genome annotation. The protein sequences were analyzed using the online tool MEME Suite (https://meme-suite.org/meme/tools/meme) with default iterative cycles and the maximum number of motifs set to 20. The Compute pI/MW tool was used on the ExPasy (http://au.expasy.org/tool.html) for analysis to obtain the various physical and chemical properties of the NLRs, including length, molecular weight, aliphatic index and isoelectric point.

### 2.5 Ka/Ks selective pressure and covariance analysis

The genome analysis of *NLR* duplications was carried out using the default settings of MCScanX 1.1 and TBtools 1.098 to identify orthologous and paralogous genes. Gene duplication patterns were categorized according to their chromosomal locations, encompassing tandem and segmental duplications (Yupeng et al., 2012). Each duplication event’s nonsynonymous substitution rate (Ka), synonymous substitution rate (Ks), and their ratio (Ka/Ks) were calculated using KaKs Calculator (Zhenglong et al., 2002). An all-versus-all BLASTN search was performed to compare nucleotide coding sequences (CDS) of TNLs and non-TNLs across eight wild strawberry species, with an E-value set at 1.0. The *NLR* multi-gene families were delineated based on a sequence coverage and identity threshold greater than 70%, with further analysis of recent duplications in NLRs among the eight strawberry species by raising the coverage and identity thresholds to >80% and >90% (Wang et al., 2010).

### 2.6 RNA-seq data analysis of the NLR gene family members

The analysis utilized publicly available RNA-Seq data from five wild diploid strawberry species, encompassing various tissues (PRJEB187983, PRJNA634576, PRJNA634576, PRJNA634576, PRJNA804380, PRJEB4896, PRJNA272956, PRJNA530684, PRJNA656765). Transcriptome samples were acquired in their original FASTQ format from the ENA database (https://www.ebi.ac.uk/ena/browser/home) using the respective NCBI project identifiers. Alignment of the transcriptomes to the reference genome was conducted employing Hisat2, SAMtools, and R, resulting in an expression matrix. To visualize gene expression levels of the NLRs, a heatmap was generated based on log2 (TPM+1) values. Differential expression analysis was carried out using either edgeR or DESeq2 packages from the Trinity program suite, with a significance threshold set at FDR (or padj) ≤ 0.05 and |log2 fold change| >2. Additionally, TBtools software, the Draw Venn Diagram website (http://bioinformatics.psb.ugent.be/webtools/Venn/), and the Venny 2.1 website (https://bioinfogp.cnb.csic.es/tools/venny/index.html) were utilized for creating UpSet or Venn diagrams.

### 2.7 qRT-PCR analysis of strawberry NLRs after pathogen infection

RNA was extracted according to the instructions of the Omega Plant RNA Kit R6827. cDNA synthesis was performed using the Takara RR037A PrimeScript RT Reagent Kit (Japan). For qRT-PCR analysis, we used the QuantStudio™ 7 Flex Real-Time PCR System (96-well format) and the Takara TB Green Premix Ex Taq II (Tli RNaseH Plus) kit. The qRT-PCR was conducted under the following conditions: initial denaturation at 95°C for 30 seconds, followed by 40 cycles of 95°C for 5 seconds and 60°C for 30 seconds. Each reaction was performed in triplicate, and the experiment included three biological replicates. Primer sequences listed in the **Supplementary Table S1**. All experiments were conducted under low-temperature conditions.

### 2.8 Identification of resistance to *B. cinerea* in isolated leaves

The identification and cultivation of *B. cinerea* were performed on agar plates. Leaf samples from six wild strawberry plants exhibiting healthy and similar-sized growth were collected and wiped clean. Under aseptic conditions, uniformly sized fungal pieces were excised and inoculated on the back of the leaves, avoiding the midrib. The samples were initially cultured in a growth chamber at 20°C and 85% relative humidity for 24 hours in darkness, followed by a 16-hour light/8-hour dark cycle. After 48 hours, the fungal pieces were removed, and the cultivation was continued. Photos were taken daily, and Image J software was used to measure and calculate the relative area of disease on each treated leaf. Leaves inoculated with sterile PDA medium served as controls.

## 3 Results

### 3.1 Identification and classification of *NBS–LRR* genes

**Figure 1:**
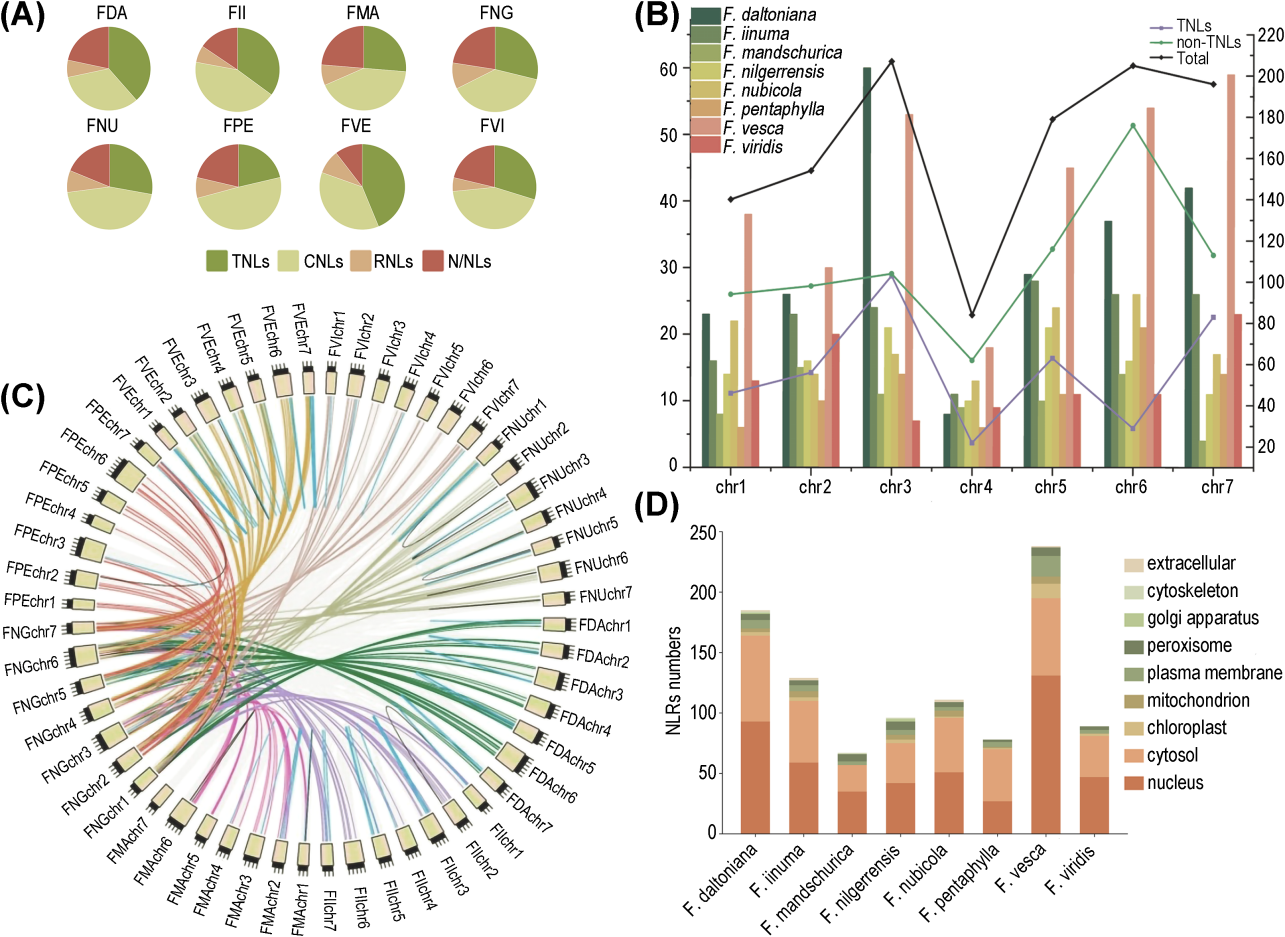
Distribution of NLRs in 8 wild strawberry species. **(A)** Quantifying the diversity of different NLRs types across eight strawberry species. **(B)** Chromosomal distribution of NLRs among different species, where the bar graph corresponds to the left y-axis indicating the number of NLRs; the line graph corresponds to the right y-axis representing the number of genes for different types of NLRs. **(C)** Comparative collinearity relationships of NLRs across eight strawberry species. **(D)** Statistics of the number of different strawberry NLRs in various organelles.

A total of 1,180 NLRs were discerned spanning eight *Fragaria* genomes. The proportion of NLRs within these genomes exhibited considerable variation, ranging from 0.37% in *F. daltoniana* to 1.25% in *F. vesca*. Notably, no substantial correlation was observed between the number of NLRs and the total gene count. For instance, *F. daltoniana*, with the highest number of genes in its genome, only contained 226 NLRs, whereas *F. vesca*, with a lower gene count, hosted the largest number of NLRs (**Table 1**). Based on distinct structures, we categorized the NLR gene family into eight subfamilies: CC-NBS-LRR (CNL), TIR-NBS-LRR (TNL), RPW8-NBS-LRR (RNL), CC-NBS (CN), TIR-NBS (TN), RPW8-NBS (RN), NBS-LRR (NL), and NBS (N). According to the presence or absence of the TIR domain in the variable N-terminal, TNL and TN are classified as TNL types, while the others are labeled as non-TNL types. Moreover, the non-TNLs accounts for over 50% of the NLR gene family in all eight strawberry species (**Figure 1A**). *F. vesca* exhibited the lowest ratio of non-TNLs to TNLs (1.28), whereas *F. pentaphylla* showed the highest (3.68), indicating a potential species-specific expansion of non-TNL genes. Different types of NLRs not only vary in quantity but also exhibit differences in chromosomal localization and physicochemical properties (**Table 1**).

**Table 1:** Classification of different NLRs in eight species of *Fragaria*.

Protein domains	Letter Code	*F. daltoniana*	*F. iinuma*	*F. mandschurica*	*F. nilgerrensis*	*F. nubicola*	*F. pentaphylla*	*F. vesca*	*F. viridis*
Total NLRs	NLR	226	154	76	111	133	89	297	94
non-TNLs subclass		139	100	56	79	96	70	167	66
Percentage of non-TNLs		61.50%	64.94%	73.68%	71.17%	72.18%	78.65%	56.23%	70.21%
CC-NBS	CN	19	25	13	13	19	20	22	9
CC-NBS-LRR	CNL	56	41	19	30	41	24	87	32
NBS	N	23	15	12	12	12	9	18	10
NBS-LRR	NL	26	9	6	13	13	10	13	10
RPW8-NBS	RN	3	3	0	2	9	1	5	1
RPW8-NBS-LRR	RNL	12	7	6	9	2	6	22	4
TNLs subclass		87	54	20	32	37	19	130	28
Percentage of TNLs		38.50%	35.06%	26.32%	28.83%	27.82%	21.35%	43.77%	29.79%
TNL-NBS	TN	22	11	4	9	6	6	18	5
TNL-NBS-LRR	TNL	65	43	16	23	31	13	112	23
CNLs/TNLs		0.86	1.22	1.6	1.34	1.62	2.32	0.84	1.46
non-TNLs/TNLs		1.6	1.85	2.8	2.47	2.59	3.68	1.28	2.36
exon mean of non-TNLs		3.91	3.2	3.79	3.35	3.23	3.31	4.7	3.7
exon mean of TNLs		4.98	6.35	5	5.35	5.22	4.89	7.17	6.46
Estimated gene num		28588	28131	23665	27594	24491	24779	23853	25411
Proportion of NLRs		0.79%	0.55%	0.32%	0.40%	0.54%	0.36%	1.25%	0.37%

CC, coiled coil; LRR, leucine-rich repeat; NBS, nucleotide binding; RPW8, resistance to powdery mildew 8; TIR, toll/interleukin receptor.

### 3.2 Chromosomal location and physicochemical properties of NLRs

We identified the highest abundance of NLRs on chr.3 and the fewest on chr. 4. Among them, chr. 3 contains the greatest number of TNLs, while chr. 6 harbors the most non-TNLs (**Figure 1B**). Our chromosomal mapping revealed a non-random distribution of NLRs, with the RNLs predominantly situated on chr. 1, the TNLs exhibiting the greatest density on chr. 3, and the CNLs being notably enriched on chr. 6. In most strawberry species, over half of the NLRs were found to form gene clusters rather than existing independently. Only three species (*F. nilgerrensis*, *F. pentaphylla*, and *F. mandschurica*) exhibited a relatively lower proportion of clustered NLRs. (**Supplementary Figure S1**).

Subsequently, we conducted synteny analysis of homologous NLRs across eight strawberry species using *F. nilgerrensis* as a reference. The results revealed that the synteny relationships are primarily associated with chromosomes. For instance, synteny was observed at high levels on chr. 6, followed by chr. 2. This suggests that these chromosomes may play a crucial role in the expansion and diversity of the NLR gene family. Furthermore, the number of orthologous gene pairs significantly surpasses that of paralogous gene pairs. In particular, *F. mandschurica* showed the highest number of both orthologous and paralogous gene pairs, while *F. pentaphylla* and *F. vesca* correspond to the smallest number of orthologous and paralogous genes, respectively (**Figure 1C**; **Supplementary Table S2**).

Physicochemical property analysis of the NLR proteins from the eight strawberry species indicated that the majority were relatively short, with over 90% containing fewer than 2000 amino acid residues. Isoelectric point analysis unveiled a preponderance of alkaline proteins. Furthermore, in terms of hydrophilicity and stability index, it is evidenced that the majority of NLR proteins exhibit hydrophilic yet unstable characteristics (**Supplementary Table S3**). Lastly, in the prediction of subcellular localization, it is observed that NLRs are predominantly concentrated within the nucleus, followed by the cytoplasm, with *F. pentaphylla* representing an exception (**Figure 1D**).

### 3.3 Phylogenetic analysis of NLRs

**Figure 2:**
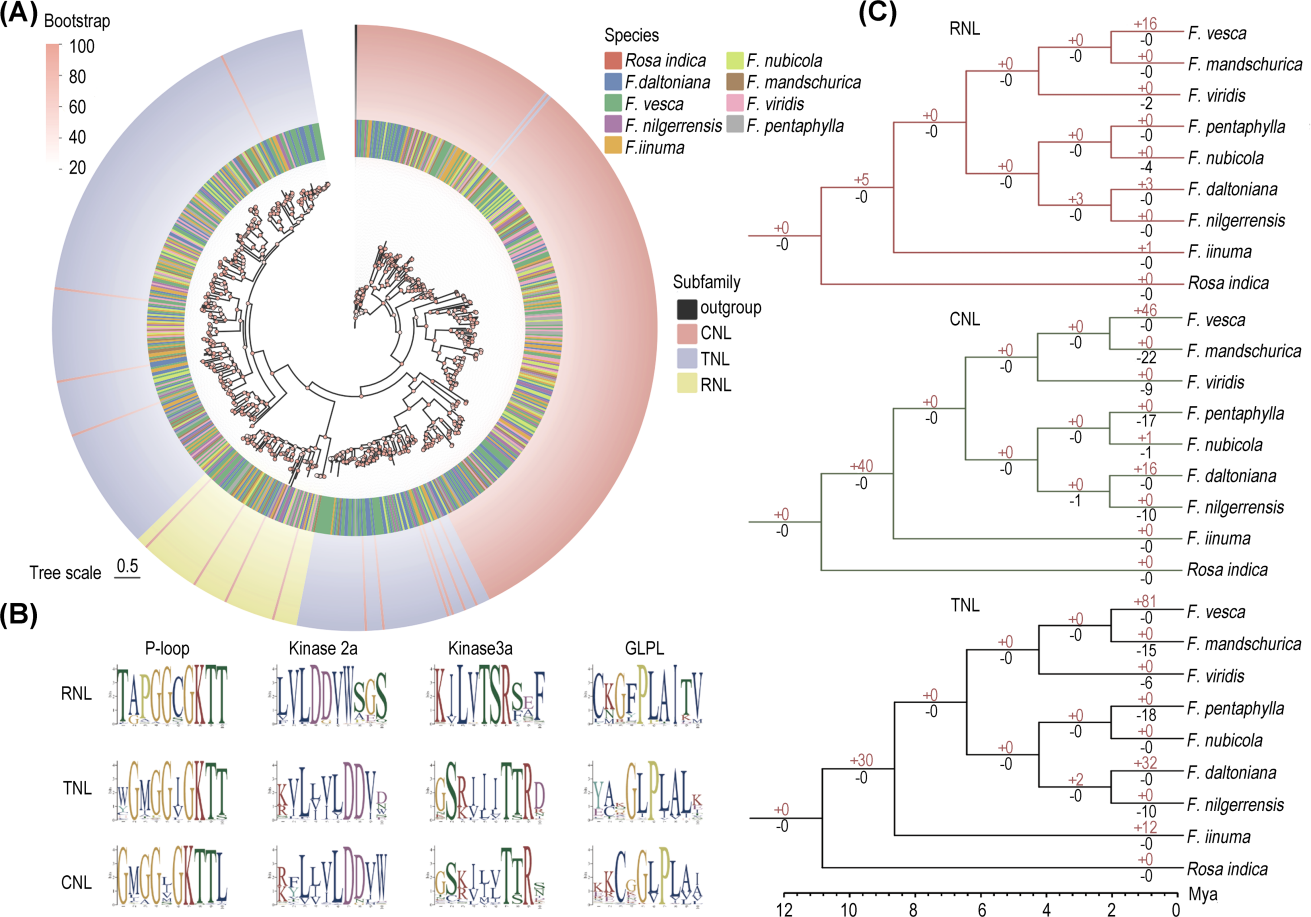
Evolution of NLRs in eight strawberry species. **(A)** Maximum likelihood phylogenetic tree of NLRs in eight strawberry species. The circles represent different species and different subfamilies from inside to outside respectively. **(B)** Conservative motifs of the NB-ARC structural domains in the three major subfamilies analyzed in Logo. **(C)** Gene duplication and loss events in the three major subfamilies, with red numbers on the branches denoting gene duplication and black numbers denoting gene loss.

To understand the evolutionary patterns of NLRs among eight wild strawberry species, a maximum likelihood phylogenetic tree was constructed for full-length NLRs (RNLs, CNLs, TNLs) (**Figure 2A**). The tree revealed that CNLs formed several successive clades and occupy a basal position. RNLs form an independent branch sister to the major clade of TNLs, with a few CNLs interspersed within them. Notably, TNLs are polyphyletic, with one clade clustering with RNLs and another clade (comprising 72 TNL genes) grouping with CNLs (70 genes). This phenomenon may be associated with factors such as gene recombination or functional transfer (Song et al., 2017).

To elucidate the cluster pattern of TNLs, we conducted domain analyses on five randomly selected genes from branches of CNLs and both TNLs. The results show that TNLs clustering with CNLs all possess the same P-loop domain (motif 9), which is a conserved region within the NBS domain. In contrast, TNLs that cluster with RNLs lack this domain (**Supplementary Figure S2**).

Further phylogenetic analysis showed that the phylogenetic topology of NLRs was consistent in eight strawberry species, indicating a conservative evolutionary of NLRs within the *Fragaria* genus (**Supplementary Figure S3**). Moreover, the proportion of genes lacking the LRR domain is higher among CNLs compared to TNLs, suggesting that this subfamily may have experienced a higher mutation rate and faster evolutionary rate during its evolutionary history (**Table 1**).

### 3.4 Analysis of conserved motifs in NLRs

To unveil the distinctive motif sequence characteristics of each NLRs subfamily, we conducted MEME analysis with a particular focus on the structural composition of their conserved motifs (**Supplementary Figure S3**). Consistent with previous studies, we first identified four highly conserved motifs (P-loop, Kinase2, GLPL, and MHDV) within the core NB-ARC domain of the NLR gene family (Biezen and Jones, 1998). Among them, the P-loop motif is the most conserved across all three subfamilies, characterized by the amino acid sequence “GKTT”. The Kinase3a motif varies, with “TSR” in RNLs and “TTR” in CNLs and TNLs. The GLPL motif also shows variation, with “GFPLA” in RNLs and “GLPLA” in CNLs and TNLs (**Figure 2B**). Therefore, the Kinase2 motif can be used to differentiate non-TNLs (CNL and RNL) from TNLs, while the Kinase3a motif allows for further distinction of RNLs from other subfamilies.

### 3.5 Evolution and duplication events of NLRs

According to the result of Notung analysis, we found the RNL, CNL, and TNL subfamilies have undergone divergent degrees of gene loss and duplication across eight wild strawberries (**Figure 2C**). The non-TNLs exhibit a notable expansion within the ancestral group of strawberries, with the CNL subfamily showing a particularly significant increase, reaching up to 40 genes. This observation explains why the CNL subfamily predominates among non-TNL subclasses. Across different species, *F. mandschurica* exhibited the highest frequency of NLRs loss, while *F. vesca* showed the most frequent NLRs duplication. Consequently, despite the comparable overall gene number between these two *Fragaria* species, there is a significant 3.9-fold variation in their *NLR* gene counts. Overall, in contrast to the relatively rapid expansions observed in the TNL and CNL subfamilies, RNL exhibits a comparatively moderate expansion, implying a tendency towards conservatism throughout its evolutionary history.

MCScanX analysis was also employed to examine the repetition rate of NLRs in eight strawberry species, it becomes apparent that the rate of duplication for non-TNLs surpasses that of TNLs (**Supplementary Table S4A**). Furthermore, by classifying the duplication patterns according to their chromosomal positions, we can distinguish between tandem and segmental duplicates. It is clear that tandem duplications significantly outnumber segmental duplications, indicating that tandem duplication plays a major role in gene amplification (**Table 2**). Remarkably, only *F. viridis* exhibited an absence of segmental duplications, implying a higher incidence of segmental deletions relative to duplications in this species. Furthermore, we distinguished between orthologous and paralogous genes. Observations revealed a marked abundance of orthologous genes over paralogous ones. Among these, the number of TNL-type homologous genes (479 pairs) is significantly lower than that of non-TNL-type homologous genes (1,295 pairs) (**Supplementary Table S4B**).

**Table 2:** Duplication and types of NLRs in eight strawberries.

duplication types	gene pairs	*F. daltoniana*	*F. iinuma*	*F. mandschurica*	*F. nilgerrensis*	*F. nubicola*	*F. pentaphylla*	*F. vesca*	*F. viridis*
ALL		138	58	29	28	60	30	167	10
Tandem duplication	ALL	94	41	15	17	36	14	129	10
	TNLs	47	13	8	2	12	1	64	4
	non-TNLs	47	28	7	15	24	13	65	6
Segmental duplication	ALL	73	19	15	15	33	21	87	0
	TNLs	26	10	4	7	3	4	40	0
	non-TNLs	47	9	11	8	30	17	47	0

### 3.6 Selection pressure analysis of NLRs

**Figure 3:**
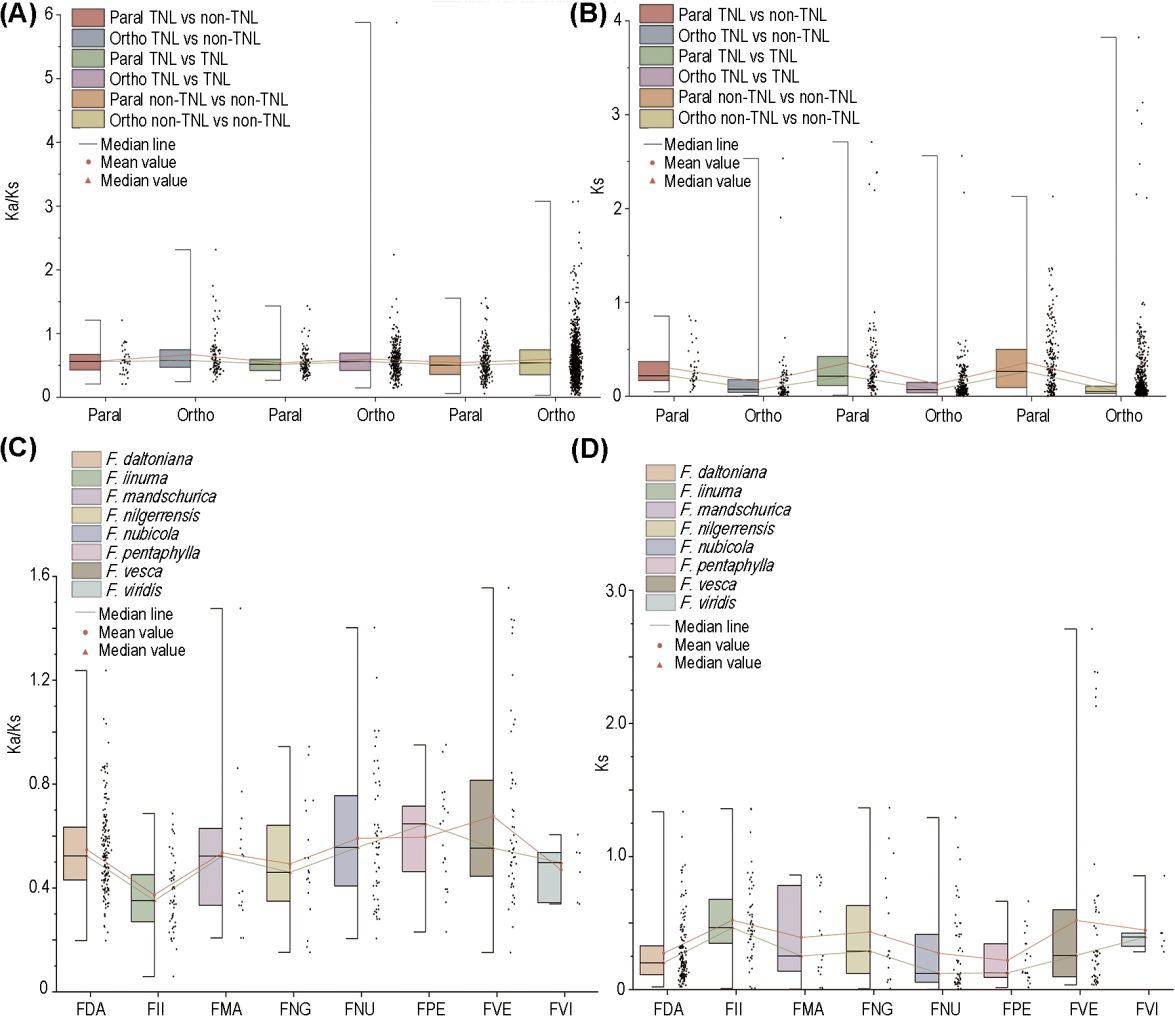
Selective pressure of NLRs. **(A)** The Ka/Ks values for orthologous and paralogous genes of different types of NLRs. **(B)** The Ks values for orthologous and paralogous genes of different types of NLRs. **(C)** The Ka/Ks values for NLRs in 8 strawberry species. **(D)** The analysis of Ks values for NLRs in 8 strawberry species. The red line represents the mean, while the green line represents the median.

The ratio of non-synonymous nucleotide substitutions to synonymous nucleotide substitutions (Ka/Ks) is a key parameter for assessing whether genes are under selective constraint. A Ka/Ks value >> 1 indicates positive selection on the gene; a Ka/Ks value ≈ 1 suggests neutral selection; and a Ka/Ks value << 1 implies purifying selection. In our comparative analysis encompassing eight strawberry species, no substantial disparities were observed in the Ka/Ks and Ks values between TNLs and non-TNLs (**Figure 3A**). Nevertheless, the comparation between orthologous and paralogous gene pairs revealed significantly higher Ks values in paralogous genes, suggesting a more conservative evolutionary trajectory of *NLR* paralogs within their respective species. On the whole, most of homologous gene pairs have Ka/Ks values below 1, generally around 0.5, suggesting NLRs in strawberries underwent relaxed selection. Gene pairs with Ka/Ks > 1 were found exclusively in *F. daltoniana*, *F. mandschurica*, *F. nubicola*, and *F. vesca* (**Figures 3A–D**). Moreover, among gene pairs with Ka/Ks > 1, the number of non-TNLs significantly exceeded that of TNLs, suggesting a stronger degree of positive selection on non-TNLs (**Supplementary Table S5**). Moreover, within these gene pairs with Ka/Ks > 1, the tally of non-TNLs significantly eclipsed that of TNLs, implying a heightened degree of positive selection exerted on non-TNLs (**Supplementary Table S5**).

### 3.7 Gene structure of NLRs

**Figure 4:**
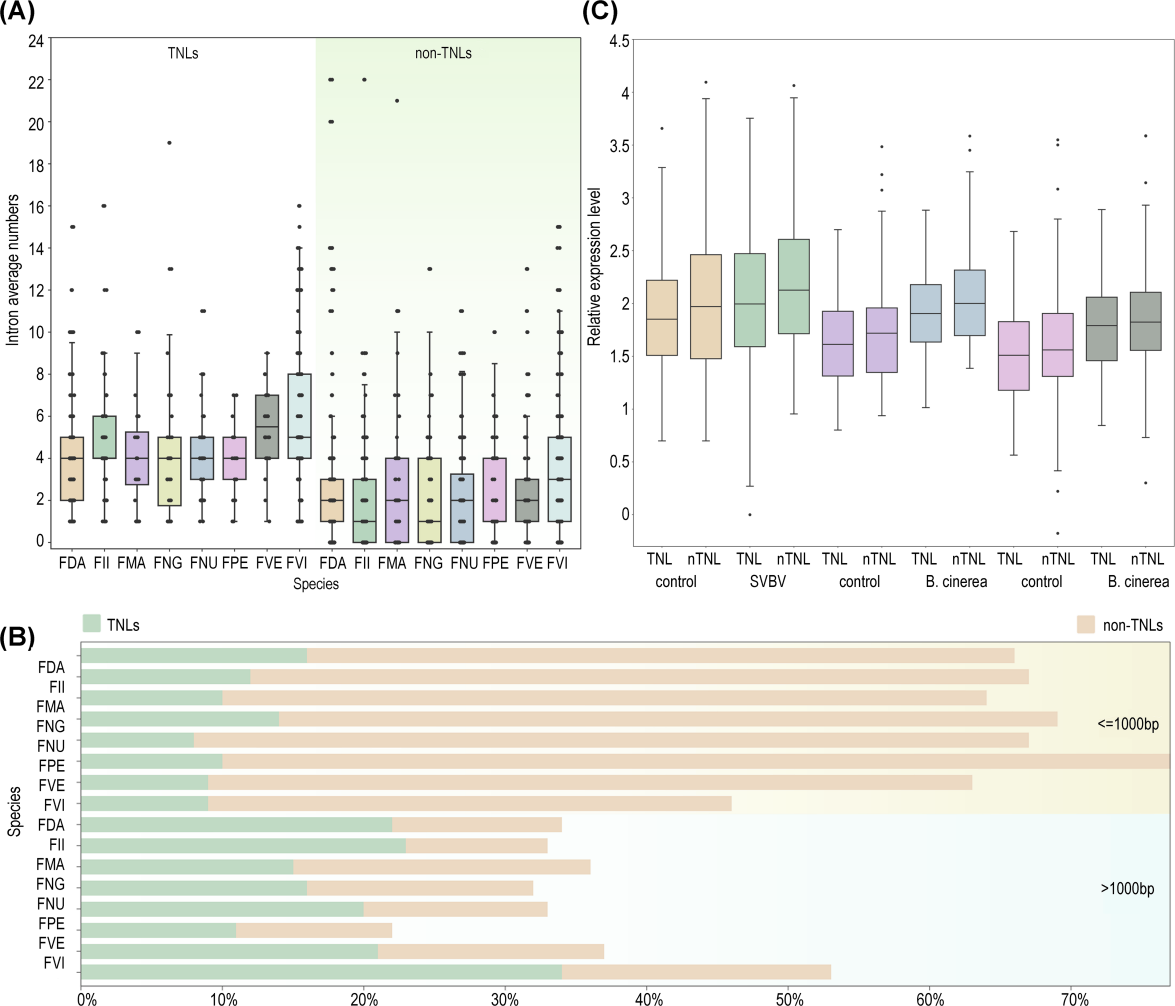
Characteristics of NLRs structure in different strawberry species. **(A)** Variation of intron numbers in two types of NLRs (TNLs, non-TNLs) across eight strawberry species. **(B)** Comparison of standardized expression levels between non-TNLs and TNLs in *F*. *vesca* under three infection scenarios (leaf infection by SVBV virus and red/white fruit infection by *B*. *cinerea*). **(C)** The proportions of genes with fewer than or equal to 1000 amino acids and those with more than 1000 amino acids in TNLs and non-TNLs types across eight strawberry species are analyzed.

To investigate the difference of gene structures between TNLs and non-TNLs, we examined the exon/intron architecture of NLRs across eight strawberry species. The findings revealed that the average number of exons in non-TNLs (3.6) was significantly lower than that in TNLs (5.7). Consistently, non-TNLs exhibited fewer average introns and shorter protein lengths compared to TNLs (**Figures 4A, B**). Studies have indicated that genes with relatively shorter protein lengths typically demonstrate higher expression levels (Chiaromonte et al., 2004; Grishkevich and Yanai, 2014). As the number of introns increases, cells must expend more energy for accurate splicing and the removal of introns at precise locations within complex spliceosomal machinery, leading to reduced expression levels (Jo and Choi, 2015; Caldas et al., 2024). In alignment with this hypothesis, our analysis of expression profiles in infected foliage, fruits, and control samples of *F. vesca* confirmed that the expression levels of structurally shorter non-TNLs exceeded those of TNLs (**Figure 4C**).

### 3.8 Expression pattern of NLRs

**Figure 5:**
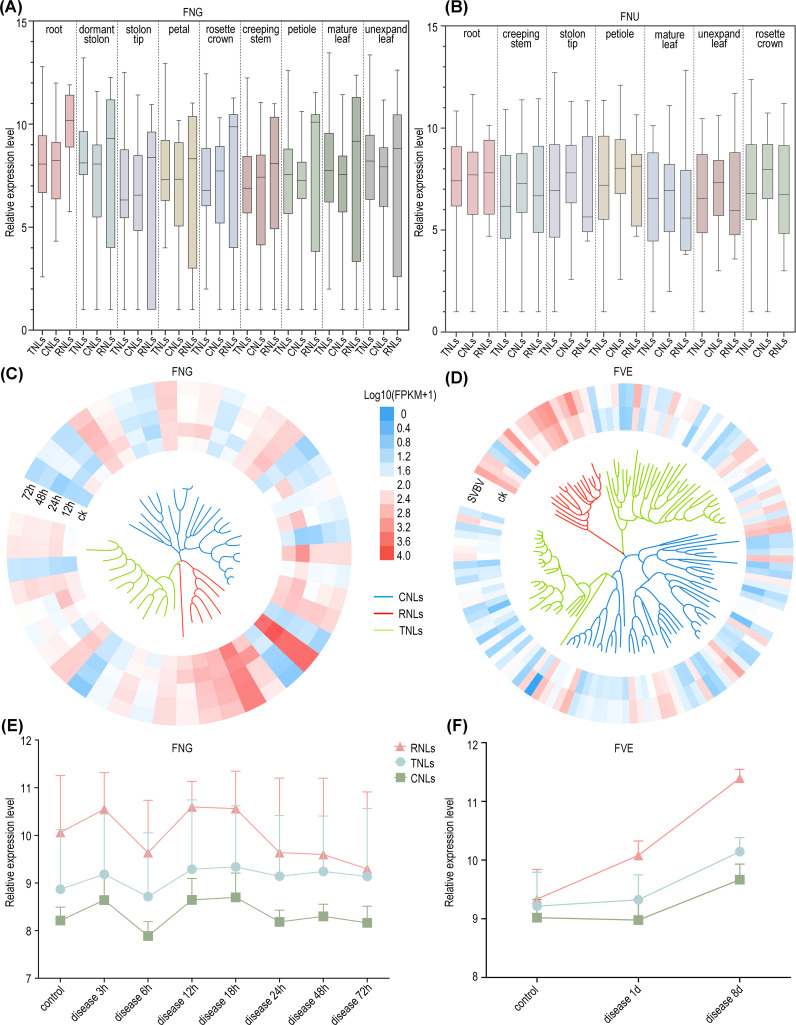
Expression profile of NLRs. **(A)** Normalized expression profiles of three types of NLRs in nine tissues of *F*. *nilgerrensis*. **(B)** Normalized expression profiles of NLRs in seven tissues of *F*. *nubicola*. **(C)** Expression heatmap of NLRs in *F*. *nilgerrensis* leaves at different time points infected with *Colletotrichum*. **(D)** Expression heatmap of NLRs in *F*. *vesca* leaf infection by SVBV virus. The inner part shows the phylogenetic tree with color-coded branches for RNLs (red), CNLs (blue), and TNLs (green). **(E)** The temporal changes of NLRs expression following *Colletotrichum* infection in *F*. *nilgerrensis* leaf. **(F)** The temporal changes of NLRs expression in *F*. *vesca* leaf after *E*. *graminis* infection.

Subsequently, we conducted a comprehensive analysis of additional transcriptomic data for five diploid wild strawberry species available in public databases. Our analysis revealed that NLRs are ubiquitously expressed across various tissues in these strawberry species, with no distinct tissue specificity. However, different NLRs types exhibited varying expression dominance in different species, while maintaining consistent expression pattern across tissues within a single species. For instance, in *F. pentaphylla* and *F. nubicola*, CNLs exhibit higher expression levels than TNLs and RNLs across all tissues, while in *F. vesca*, *F. viridis*, and *F. nilgerrensis*, RNLs show higher expression levels across all tissues (**Figures 5A, B**; **Supplementary Figure S4**).

From the perspective of disease expression, RNLs, which are categorized as non-TNLs, highly expressed in the post-infection expression profiles of *F. vesca* and *F. nilgerrensis* (**Figures 5C, D**). This suggests that RNLs, traditionally considered auxiliary genes, play significant roles in the process of wild strawberries combating various pathogens. Consistent with prior studies, our findings suggest that RNLs may not only be responsive to *E. graminis* but also show significant reactions to other pathogens (Xiao et al., 2001; Ghelder et al., 2019). Moreover, by examining the expression levels at different time points post-infection in two strawberry species, the rate increase in expression levels of both RNLs and CNLs surpasses that of TNLs in *F. nilgerrensis* following the nascent phases of infection (3 hours into the disease); after 18 hours, the expression of RNLs and CNLs significantly decreased, while TNLs did not show a notable decline and maintained a stable expression level. In *F. vesca*, RNL expression rapidly increased during the initial phase of infection (1d), whereas in the later stages of infection (8d), the expression of all three types of NLRs increased significantly (**Figures 5E, F**). This suggests that in this study, non-TNLs, especially RNLs, exhibit a faster response rate than TNLs during the early stages of infection, and this difference in response rate may be associated with the distinct disease resistance mechanisms of different *NLR* types.

**Figure 6:**
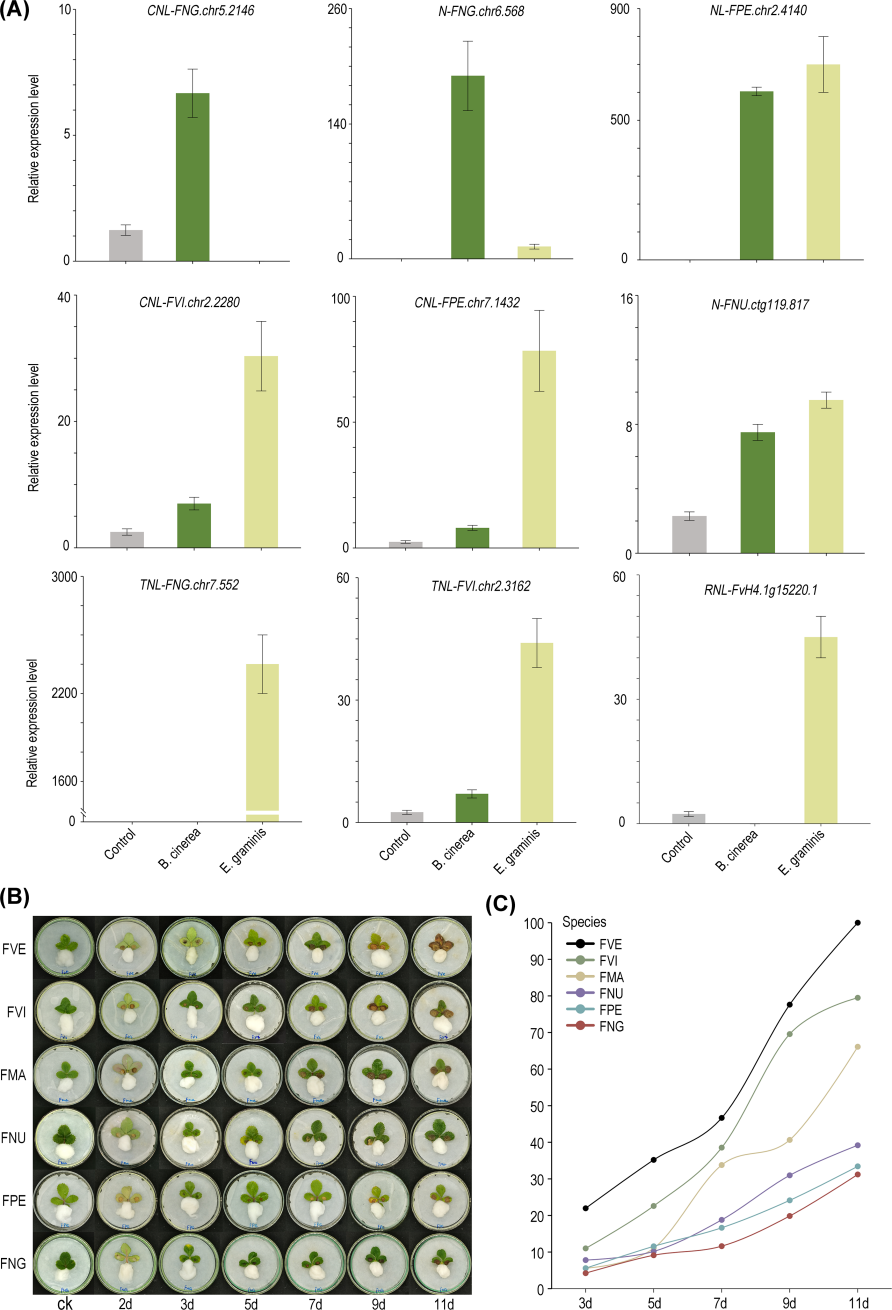
Differences in disease resistance among eight strawberry species. **(A)** The qRT-PCR verification of 9 NLRs. Bars representing means from three biological replicates. **(B)** Morphological images of the susceptibility of six strawberry species to *B*. *cinerea* infection in leaf tissues. **(C)** The calculation of leaf disease area ratios in six strawberry species at different time points.

According to the transcriptomes data, 15 differentially expressed NLRs were chosen from the six diploid wild strawberry species in our lab. These NLRs were analyzed with qRT-PCR to examine their expression level after infection with *B. cinerea* or *E. graminis* (**Supplementary Figure S6**). Among them, 9 genes exhibited significantly increased expression in post-infection period, while the expression levels of the remaining 6 genes decreased compared to the control group. Regarding gene types, among the five TNLs, 2 genes exhibited higher expression levels after infection compared to the control group. In contrast, among the ten non-TNLs, 8 genes showed higher expression levels in post-infection period relative to the control group. Notably, among the upregulated genes, 5 of them showed enhanced expression levels following infections by either *B. cinerea* or *E. graminis*, suggesting their ability to mount a defense response against multiple pathogens. Furthermore, *FNG.chr5.2146* (*CNL*) and *FNG.chr6.568* (*N*) were significantly upregulated only after infection with *B. cinerea*, while *FNG.chr7.552* (*TNL*) and *FvH4.1g15220.1* (*RNL*) showed significant upregulation exclusively following infection with *E. graminis*, indicating their potential specificity in response to particular pathogens (**Figure 6A**).

### 3.9 Differences in resistance of wild strawberry leaves to *B. cinerea*

Here, we assessed the resistance of six diploid wild strawberry species to *Botrytis cinerea*, a common fungal pathogen, using *in vitro* leaf infection models. The severity of the disease (DS) was graded by defining it as the average lesion area divided by the total average leaf area multiplied by 100%. Based on previous research, the disease resistance of each strawberry was classified as follows: VR grade: DS = 0; MR grade: 0 < DS ≤ 5%; R grade: 5% < DS ≤ 15%; T grade: 15% < DS ≤ 30%; S grade: 30% < DS ≤ 50%; MS grade: 50% < DS ≤ 75%; VS grade: 75% < DS ≤ 100% (Perdones et al., 2012; Hahn, 2014). With the exception of *F. vesca*, characterized by a higher ratio of non-TNLs/TNLs and classified as the VS class, the remaining strawberry species, with relatively fewer non-TNLs/TNLs, are all categorized as MS class or lower (**Supplementary Table S6**). Seven days post-inoculation, apart from *F. nubicola*, *F. nilgerrensis*, and *F. pentaphylla*, the DS of other strawberry leaves significantly increased. By the 11th day, two small leaves of *F. vesca* were almost entirely infected. Notably, *F. nilgerrensis* and *F. pentaphylla* maintained the lowest disease severity (DS) scores, averaging around 30%, with *F. nilgerrensis* being the most resistant, showcasing its robust defense mechanisms against *B. cinerea* (**Figures 6B, C**).

## 4 Discussion

Cultivated strawberries are vulnerable to a multitude of diseases, and the most straightforward method for enhancing their disease resistance lies in the breeding of resilient plants. The establishment of the strawberry pan-genome has made genomic research on disease-resistant *NBS-LRR* genes attainable, thus providing a foundation for the discovery of superior disease-resistant genes in wild strawberries.

### 4.1 Variations in NLRs quantity across different strawberry species

In current research, NLRs are categorized into two types: TNLs and non-TNLs, with their proportions exhibiting intricate diversity across different plants (Bahler et al., 2008; Zhong and Cheng, 2016). For instance, in *Arabidopsis* and soybean, over half of the NLRs belong to the TNL type (Akira et al., 2003; Perazzolli et al., 2014). Conversely, in potato, cassava, and water lily, the non-TNLs are more abundant among NLRs (Jia et al., 2000; Parker, 2020). In accordance with the latter, our examination of eight strawberry species reveals a predominance of non-TNL NLRs. This observation is likely arises from the diverse life histories of different plants, suggesting that the varied evolution of TNLs and non-TNLs may be driven by distinct ecological factors (e.g. adaptation to different pathogens), leading to distinct evolutionary patterns (Zhong et al., 2015).

The disparity in NLRs quantity among different species during the evolutionary process is most likely caused by genetic duplication events. In *Arabidopsis thaliana*, the *NLR* family exhibits moderate levels of tandem duplications and low levels of segmental duplications (Wei et al., 2020). Similarly, within the *Rosaceae* lineage, *Prunus persica* exhibits a replication pattern with a moderate prevalence of tandem repeats (36%) and a low occurrence of segmental duplications (2%). This indicates a tendency to enhance the diversity of the NLR gene family primarily through tandem repetition in these species. It is noteworthy that within the wild strawberry *NLR* family, the occurrence of duplications is predominantly concentrated in two forms: tandem duplications (58%) and segmental duplications (42%). This observation suggests that while tandem duplications prevail in frequency, the role of segmental duplications in the expansion of the NLR gene family within wild strawberries should not be overlooked. Additionally, these tandemly duplicated genes often cluster along chromosomes, aligning with the clustering phenomenon of tandem NLRs previously observed in tomatoes by researchers (Yang et al., 2008). Clustered NLRs are esteemed as repositories of genetic diversity, for they harbor the potential to foster the accrual of variations such as gene unequal crossing over, gene conversion, and ectopic recombination. These processes may culminate in the emergence of non-functional pseudogenes or NLRs endowed with novel functionalities (Takken et al., 2006). Frequent duplication events have led to most strawberry species having NLRs belonging to multi-gene families (**Supplementary Table S7**). Within these families, the abundance of non-TNL subclass surpasses that of the TNL subclass. On one hand, the relatively subdued duplication rate of TNLs during the course of evolution may be attributed to sporadic replication or swift deletion following duplication (Feliner and Rosselló, 2012). On the other hand, it implies that non-TNLs with high duplication frequencies are more likely to demonstrate a greater diversity in disease resistance functions in wild strawberries.

### 4.2 Variation in selective pressure on NLRs

Moreover, NLRs not only vary in abundance but also exhibit disparities in selection pressure. Within the Rosaceae lineage, encompassing species such as apple, pear, peach, and mei, TNLs demonstrate markedly higher Ka/Ks values compared to non-TNLs (Zhong et al., 2015). However, our findings differ from previous studies on the selection pressure of NLRs in strawberries with varying ploidy levels (Zhong et al., 2018). In our exploration of eight diploid strawberries, we did not find significant differences in the mean Ka/Ks and Ks values between TNLs and non-TNLs. However, it is evident that a higher proportion of non-TNLs are found among genes with Ka/Ks > 1. Therefore, we speculate that in high-ploidy strawberries, particularly in cultivated species, more TNLs might have been retained. Conversely, during artificial domestication, there may have been a greater loss of non-TNLs, leading to the loss of many valuable disease-resistant genes in cultivated strawberries.

Furthermore, comparing the selection pressure of homologous genes also reveals that the Ks value of paralogous genes is significantly higher than that of orthologous genes, possibly due to paralogous genes often originating from gene family expansion events within the same species, implying closer evolutionary relationships and functional connections among these genes, leading to higher conservation. In contrast, orthologous genes, which may come from different species or distinct evolutionary paths, generally show lower conservation.

### 4.3 Subfamily classification of NLRs

Previous phylogenetic studies suggested that the RNL subfamily is a branch of the CNL subfamily. However, recent research indicates that in the evolutionary process of dicotyledonous plants, RNLs may have evolved independently from CNLs, a viewpoint confirmed in our analysis of the strawberry NLR gene family’s evolution (Zhong and Cheng, 2016). Additionally, we observed that RNLs appear phylogenetically closer to TNLs, possibly because genetic recombination or gene transfer events may have allowed some level of gene segment sharing or exchange between them. Moreover, their close functional role in disease resistance mechanisms might explain their proximity in evolutionary trees.

### 4.4 Disease resistance mechanisms of different type of NLRs

The nuances in disease resistance mechanisms can significantly influence the efficacy of various *NLR* types in combatting diseases. Previous studies have elucidated that CNLs possess the ability to directly discern pathogen effector proteins, thereby initiating effector-triggered immunity (ETI) (Sun et al., 2020; Förderer et al., 2022). In contrast, the resistance mechanism of TNLs is more intricate, as the activated TNL resistance complex necessitates helper NLRs (RNLs) as a signaling intermediary to initiate ETI. Acting primarily as a conduit, auxiliary RNLs cannot independently recognize pathogen effector proteins; however, their presence is indispensable for eliciting robust responses from NLRs (including some CNLs and all TNLs) to fully activate downstream immunity (Cannon et al., 2002; Jubic et al., 2019; Wan et al., 2019). This could be a significant factor contributing to the swifter and more robust response exhibited by RNLs in disease resistance processes.

Therefore, there may be interactions between RNLs and TNLs. As reported in *Arabidopsis*, In the RNL family, NRG1 mediates cell death downstream of TNLs, while ADR1 promotes resistance downstream of TNLs. Current models suggest that, upon activation, RNLs aggregate on the cell membrane to form resistance bodies and function as calcium channels (Wang et al., 2023).

### 4.5 Genetic structural differences among NLRs

However, the factors influencing the response rate of NLRs may derive not solely from differences in disease resistance mechanisms but also from variations in gene structure. For instance, research has suggested that genes with fewer introns incur lower costs in transcription and splicing dynamics, thereby facilitating expedited reactions to environmental fluctuations and furnishing heightened selective benefits for species (Parker, 2020). In our research, non-TNLs with a lower average number of introns indeed demonstrated a more rapid response than TNLs during the initial stages of strawberry infection. Notably, RNLs, a subset of non-TNLs, demonstrated not only a swift response but also sustained high-level expression. We speculate that this might be linked to their function as signaling transducers, continuously participating in the activation pathways of multiple genes. Additionally, their elevated GC content may also play a role in this phenomenon.

### 4.6 qRT-PCR analysis of NLRs

qRT-PCR analysis identified a TNL gene on chr. 3, which has the highest number of TNLs. However, *TN-FvH4.3g44370.1* showed low expression under the stress of both pathogens, suggesting that this gene may be negatively regulated in leaf disease resistance. On chr. 6, which has the most non-TNLs, *N-FNG.chr6.568* had a significant increase in expression under *B. cinerea* stress, indicating its potential role in responding to specific pathogens. In *F. pentaphylla*, which has the highest proportion of non-TNLs, three genes were analyzed, with two non-TNL genes showing strong responses to both *B. cinerea* and *E. graminis*. In contrast, *F. vesca*, which has the lowest proportion of non-TNLs, also had three genes analyzed, but only one non-TNL gene responded specifically to *E. graminis*. Additionally, qRT-PCR of genes on chr. 2 showed that three non-TNL genes had significant responses to both *B. cinerea* and *E. graminis*, while among the three TNL genes, only *TNL-FVI.chr2.3162* had higher expression than the control after infection with both pathogens. This supports the idea that non-TNLs might offer better disease resistance compared to TNLs.

It is noteworthy that all five TNLs tested showed a stronger response to *E. graminis* stress compared to *B. cinerea* stress. This suggests that TNLs may have a higher specificity in responding to *E. graminis*.

### 4.7 Expression pattern of NLRs

Additionally, an analysis of RNA-Seq expression data reveals that, whether under normal or infected conditions, non-TNLs show higher expression levels, aligning with the aforementioned notion that non-TNLs play a primary role in strawberry growth. Similarly, in our *in vitro* inoculation experiment with *B. cinerea*, strawberry species with a higher proportion of non-TNLs generally show better disease resistance. However, factors contributing to differences in resistance include not only the proportion and structure of resistance genes but also require a thorough analysis from various perspectives, including individual plant variation, growth conditions, pathogen types, among others.

## 5 Conclusion

In summary, we have elucidated the evolutionary divergences of various *NLR* gene categories among eight wild strawberry species and their correlation with disease resistance. The superiority of non-TNLs in terms of quantity, structure, selection pressure, and expression levels highlights their potential as primary candidates for breeding disease-resistant strawberry species. Notably, RNLs exhibit a significant response following strawberry infection. Additionally, distinct wild strawberry species exhibit varying degrees of resistance to *B. cinerea* invasion. Among these, *F. vesca*, with a lower proportion of non-TNLs, displays susceptibility, whereas *F. nilgerrensis*, with a higher proportion of non-TNLs, exhibits advantageous resistance.

## Data Availability

The datasets presented in this study can be found in online repositories. The names of the repository/repositories and accession number(s) can be found in the article/**Supplementary Material**.

## Supplementary material

Supplementary Figure S1 Chromosomal mapping analysis of NLR gene family in eight species of strawberries.

Supplementary Figure S2 Motif analysis of different TNL cluster genes.

Supplementary Figure S3 Evolutionary tree of the NLR gene family in eight species of strawberries.

Supplementary Figure S4 Motif distribution map of three subfamily (TNL, CNL, RNL) genes in eight strawberries.

Supplementary Figure S5 Expression levels of NLRs in different parts of five strawberry species under normalized conditions.

Supplementary Figure S6 qRT-PCR was used to detect the expression of 15 genes infected with *B. cinerea* or *E. graminis.*
