# Supplementary material for: Evolutionary analysis of TIR- and non-TIR-NBS-LRR disease resistance genes in wild strawberries
Source: Front Plant Sci. 2024 Nov 21;15:1452251. doi: 10.3389/fpls.2024.1452251 (PMC11617207; doi:10.3389/fpls.2024.1452251)
Supplement: Supplementary file 1 [file DataSheet1.docx]

Supplementary Information

**Evolutionary analysis of TIR- and non-TIR-NBS-LRR disease resistance genes in wild strawberries**

Ni Zhu^a#^, Yuxi Feng^a#^, Guangxin Shi^a^, Qihang Zhang^a^, Bo Yuan^a^, Qin Qiao^b*^

1. School of Agriculture, Yunnan University, Kunming 650091, China
2. College of Horticulture and Landscape, Yunnan Agricultural University, Kunming 650201, China

^#^ These authors contributed equally to this work.

^*^ Correspondence: Q.Q. (qiaoqin@ynau.edu.cn)

**Table S1** Primer list

| Primers | Base sequence(5'-3') | Purpose |
| --- | --- | --- |
| FMAActin-F | TCCACCATGTTCCCGGGA | qRT-PCR reference gene |
| FMAActin-R | TGGAGCCTCCGATCCACA | qRT-PCR reference gene |
| FNGActin-F | CTTTTGGATTGAGCCTCGTC | qRT-PCR reference gene |
| FNGActin-R | ACGAGCTGTTTTCCCTAGCA | qRT-PCR reference gene |
| FNU18S-F | CACTGCAGGGCCCAATCA | qRT-PCR reference gene |
| FNU18S-R | GCGCCCGAAAACAACGTT | qRT-PCR reference gene |
| FPEActin-F | TCCACCATGTTCCCGGGA | qRT-PCR reference gene |
| FPEActin-R | TGGAGCCTCCGATCCACA | qRT-PCR reference gene |
| FVHActin-F | GCCAGAAAGATGCTTATGTCGGTG | qRT-PCR reference gene |
| FVHActin-R | TGGGGCAACACGAAGCTCAT | qRT-PCR reference gene |
| FVIActin-F | ATGGCAGAAACCGAGGAC | qRT-PCR reference gene |
| FVIActin-R | AATCAAGGGCAATGTAAG | qRT-PCR reference gene |
| CNL-*FNG.chr5.2146*-F | CTCCACCAGTGCCTGCAA | qRT-PCR |
| CNL-*FNG.chr5.2146*-R | CCACCCAACCAGCTGCTT | qRT-PCR |
| CNL-*FPE.chr7.1432*-F | ATTCCGGCAAGGTCAGGC | qRT-PCR |
| CNL-*FPE.chr7.1432*-R | GCCATCTAGAGGCGTCGG | qRT-PCR |
| CNL-*FVI.chr2.2280*-F | AAGGAGAGGAGCAACGCT | qRT-PCR |
| CNL-*FVI.chr2.2280*-R | ACCATCGGTCTGGCTCTCT | qRT-PCR |
| N-*FNG.chr6.568*-F | GTCTTCGGAATGGGAGGTGT | qRT-PCR |
| N-*FNG.chr6.568*-R | TCAAGTTGGGTGTCTGGGAT | qRT-PCR |
| N-*FNU.ctg119.817*-F | TGAAAGTGGTGGAGGCGG | qRT-PCR |
| N-*FNU.ctg119.817*-R | CTTCTCCTCGTCACCGCC | qRT-PCR |
| NL-*FNG.chr6.2521*-F | GGCCTCAGGTACTAAGGAACG | qRT-PCR |
| NL-*FNG.chr6.2521*-R | CATACAGCCACAAAGCGTCAT | qRT-PCR |
| NL-*FPE.chr2.4140*-F | CAACGCTTCAGCCACTGC | qRT-PCR |
| NL-*FPE.chr2.4140*-R | CCAGCAACTCCACGTCGT | qRT-PCR |
| NL-*FPE.chr3.434*-F | GTTGCCGATCTGGGAGGG | qRT-PCR |
| NL-*FPE.chr3.434*-R | CCAATCGCATGGGGAGCA | qRT-PCR |
| RNL-*FvH4.1g15220.1*-F | TGGACTGGAGGGGCCTTT | qRT-PCR |
| RNL-*FvH4.1g15220.1*-R | CGCATCCTCCTGGAGCAG | qRT-PCR |
| RNL-*FvH4.6g18970.1*-F | GCCACTGATCAGGCCGAT | qRT-PCR |
| RNL-*FvH4.6g18970.1*-R | TTGCGCGCCTTCCATGAT | qRT-PCR |
| TN-*FMA.chr2.2280*-F | GAGGTCTAGACACGCGCC | qRT-PCR |
| TN-*FMA.chr2.2280*-R | CCACCAGGGCATGCTCAA | qRT-PCR |
| TN-*FvH4.3g44370.1*-F | GCGGCTGAGTGAGAGTGG | qRT-PCR |
| TN-*FvH4.3g44370.1*-R | AGCTCCCGTCCACTTCCT | qRT-PCR |
| TNL-*FMA.chr2.2281*-F | TCAAGTGCAGGAGCCAGC | qRT-PCR |
| TNL-*FMA.chr2.2281*-R | GGGCCGCTTCCAGAATCA | qRT-PCR |
| TNL-*FNG.chr7.552*-F | СТАСТССАТТGCCACCACTA | qRT-PCR |
| TNL-*FNG.chr7.552*-R | TACCTGAAGCCAGTTATTCG | qRT-PCR |
| TNL-*FVI.chr2.3162*-F | TCAAGTGCAGGAGCCAGC | qRT-PCR |
| TNL-*FVI.chr2.3162*-R | GCGGATCCCACATGCCTT | qRT-PCR |

**Table S2** Eight pairs of homologous NLRs in strawberries

| class | FDA | FII | FMA | FNG | FPE | FVI | FNU | FVE |
| --- | --- | --- | --- | --- | --- | --- | --- | --- |
| Orthologous gene pairs | 127807 | 126183 | 129899 | 123528 | 116790 | 124432 | 128178 | 127237 |
| paralogous gene pairs | 324 | 446 | 2003 | 329 | 1565 | 198 | 1310 | 89 |
| chr0 gene pairs | 16824 pairs | | | | | | | |
| chr1 gene pairs | 63765 pairs | | | | | | | |
| chr2 gene pairs | 92693 pairs | | | | | | | |
| chr3 gene pairs | 89174 pairs | | | | | | | |
| chr4 gene pairs | 70382 pairs | | | | | | | |
| chr5 gene pairs | 78231 pairs | | | | | | | |
| chr6 gene pairs | 108455 pairs | | | | | | | |
| chr7 gene pairs | 75078 pairs | | | | | | | |

**Table S3** Physicochemical properties of eight strawberry NLR proteins

| IDSequence | Num. of Exon | Num. of Intron | Num. of CDS | Num. of UTR | Num. of Amino Acid | Molecular Weight | Theoretical pI | Instability Index | Aliphatic Index | Grand Average of Hydropathicity |
| --- | --- | --- | --- | --- | --- | --- | --- | --- | --- | --- |
|
| CN-FDA.chr1.06861 | 5 | 4 | 5 | 0 | 558 | 63924.77 | 6.26 | 49.23 | 97.99 | -0.288 |
| CN-FDA.chr2.11532 | 2 | 1 | 2 | 0 | 365 | 40396.75 | 7.97 | 40.92 | 96.9 | -0.127 |
| CN-FDA.chr2.12212 | 1 | 0 | 1 | 0 | 313 | 34694.65 | 5.97 | 37.18 | 78.59 | -0.491 |
| CN-FDA.chr2.12213 | 3 | 2 | 3 | 0 | 499 | 55087.49 | 6.64 | 35.69 | 83.11 | -0.48 |
| CN-FDA.chr2.12217 | 1 | 0 | 1 | 0 | 703 | 78357.73 | 6.76 | 33.6 | 101.48 | -0.184 |
| CN-FDA.chr2.17950 | 4 | 3 | 4 | 0 | 353 | 39538.32 | 9.78 | 42.32 | 94.99 | -0.205 |
| CN-FDA.chr3.05842 | 5 | 4 | 5 | 0 | 449 | 51166.41 | 6.07 | 29.36 | 97.93 | -0.049 |
| CN-FDA.chr5.03686 | 2 | 1 | 2 | 0 | 555 | 63456.46 | 6.95 | 46.97 | 97.1 | -0.266 |
| CN-FDA.chr5.25046 | 2 | 1 | 2 | 0 | 426 | 48725.35 | 8.04 | 39.83 | 92.46 | -0.398 |
| CN-FDA.chr6.13604 | 2 | 1 | 2 | 0 | 374 | 41915.21 | 5.56 | 37.44 | 101.42 | -0.195 |
| CN-FDA.chr6.16615 | 1 | 0 | 1 | 0 | 408 | 45539.38 | 5.34 | 35.7 | 97.67 | -0.137 |
| CN-FDA.chr6.17288 | 7 | 6 | 7 | 0 | 569 | 64114.82 | 7.58 | 42.96 | 97.15 | -0.26 |
| CN-FDA.chr6.17380 | 2 | 1 | 2 | 0 | 942 | 105942.44 | 5.57 | 41.68 | 105.9 | -0.027 |
| CN-FDA.chr6.19192 | 3 | 2 | 3 | 0 | 631 | 73017.79 | 7.65 | 48.83 | 95.04 | -0.306 |
| CN-FDA.chr6.20969 | 1 | 0 | 1 | 0 | 385 | 43784.41 | 8.29 | 33.89 | 85.3 | -0.539 |
| CN-FDA.chr7.00678 | 10 | 9 | 10 | 0 | 900 | 101523.3 | 6.45 | 40.03 | 95.92 | -0.239 |
| CN-FDA.chr7.00892 | 4 | 3 | 4 | 0 | 712 | 80348.85 | 6.13 | 41.5 | 100.29 | -0.185 |
| CN-FDA.chr7.14798 | 23 | 22 | 23 | 0 | 945 | 107217.69 | 6.74 | 42.9 | 88.61 | -0.399 |
| CN-FDA.chr7.20537 | 4 | 3 | 4 | 0 | 250 | 28330.41 | 5.77 | 51.37 | 99.48 | -0.322 |
| CN-FII.chr1.1528 | 8 | 7 | 5 | 5 | 1143 | 128955.8 | 5.06 | 39.02 | 98.54 | -0.162 |
| CN-FII.chr1.1941 | 4 | 3 | 4 | 0 | 270 | 30373.6 | 5.79 | 19.75 | 98.81 | -0.367 |
| CN-FII.chr2.453 | 1 | 0 | 1 | 0 | 199 | 22427.87 | 6.12 | 34.92 | 80.3 | -0.337 |
| CN-FII.chr2.469 | 10 | 9 | 10 | 0 | 940 | 103724.65 | 8.3 | 41.6 | 79.39 | -0.348 |
| CN-FII.chr2.551 | 1 | 0 | 1 | 0 | 347 | 39811.7 | 8.67 | 47.3 | 99.42 | -0.283 |
| CN-FII.chr3.2661 | 1 | 0 | 1 | 0 | 265 | 29813.29 | 5.28 | 31.8 | 103.32 | -0.134 |
| CN-FII.chr3.2924 | 2 | 1 | 2 | 0 | 442 | 50518.7 | 8.35 | 51.57 | 95.32 | -0.298 |
| CN-FII.chr3.950 | 1 | 0 | 1 | 2 | 436 | 50154.75 | 6.77 | 47.88 | 96.54 | -0.263 |
| CN-FII.chr3.955 | 2 | 1 | 2 | 0 | 795 | 90793.23 | 7.86 | 43.41 | 102 | -0.215 |
| CN-FII.chr4.1180 | 3 | 2 | 3 | 0 | 277 | 31827.8 | 5.63 | 32.59 | 107.18 | -0.223 |
| CN-FII.chr4.1429 | 2 | 1 | 2 | 0 | 289 | 32433.08 | 4.98 | 33.41 | 103.46 | -0.194 |
| CN-FII.chr5.2883 | 2 | 1 | 2 | 0 | 226 | 25253.88 | 4.81 | 30.16 | 102.74 | -0.138 |
| CN-FII.chr6.1267 | 3 | 2 | 3 | 0 | 458 | 51450.05 | 5.41 | 39.29 | 95.11 | -0.214 |
| CN-FII.chr6.1890 | 2 | 1 | 1 | 3 | 391 | 44456.24 | 7.16 | 36.96 | 86.5 | -0.499 |
| CN-FII.chr6.198 | 3 | 2 | 1 | 4 | 769 | 85661.57 | 5.33 | 41.42 | 98.09 | -0.166 |
| CN-FII.chr6.2258 | 5 | 4 | 5 | 0 | 509 | 57704.5 | 5.93 | 40.2 | 101.49 | -0.22 |
| CN-FII.chr6.2548 | 6 | 5 | 6 | 0 | 564 | 64161.15 | 6.43 | 40.45 | 100.41 | -0.235 |
| CN-FII.chr6.2623 | 23 | 22 | 23 | 0 | 910 | 103163.53 | 5.13 | 35.62 | 79.64 | -0.613 |
| CN-FII.chr6.2633 | 1 | 0 | 1 | 0 | 248 | 28444.43 | 5.52 | 35.48 | 93.15 | -0.41 |
| CN-FII.chr6.2648 | 2 | 1 | 2 | 0 | 785 | 88205.78 | 5.79 | 38.48 | 104.23 | -0.046 |
| CN-FII.chr6.2656 | 4 | 3 | 4 | 1 | 443 | 50483.21 | 5.84 | 39.73 | 98.98 | -0.242 |
| CN-FII.chr6.2817 | 1 | 0 | 1 | 0 | 346 | 39173.6 | 9.35 | 33.87 | 91.24 | -0.25 |
| IDSequence | Num. of Exon | Num. of Intron | Num. of CDS | Num. of UTR | Num. of Amino Acid | Molecular Weight | Theoretical pI | Instability Index | Aliphatic Index | Grand Average of Hydropathicity |
| CN-FII.chr6.3270.2 | 10 | 9 | 10 | 2 | 748 | 84082.86 | 6 | 37.39 | 97.18 | -0.172 |
| CN-FII.chr7.1012 | 3 | 2 | 3 | 0 | 303 | 35310.66 | 6.89 | 42.73 | 88.18 | -0.402 |
| CN-FII.chr7.1958 | 2 | 1 | 2 | 0 | 319 | 36664.22 | 6.43 | 37.02 | 93.86 | -0.336 |
| CN-FMA.chr0.199 | 2 | 1 | 2 | 0 | 429 | 48847.07 | 6.72 | 43.97 | 91.75 | -0.29 |
| CN-FMA.chr2.312 | 1 | 0 | 1 | 0 | 279 | 31925.38 | 8.44 | 42.55 | 97.03 | -0.16 |
| CN-FMA.chr2.503 | 1 | 0 | 1 | 0 | 401 | 45418 | 7.93 | 44.74 | 100.65 | -0.248 |
| CN-FMA.chr3.1343 | 2 | 1 | 2 | 0 | 247 | 28000.14 | 6.15 | 36.98 | 93.2 | -0.272 |
| CN-FMA.chr3.2162 | 3 | 2 | 3 | 0 | 286 | 32655.19 | 5.87 | 47.29 | 86.85 | -0.382 |
| CN-FMA.chr5.2235 | 2 | 1 | 2 | 0 | 230 | 26723.69 | 8.72 | 62.21 | 82.61 | -0.613 |
| CN-FMA.chr6.1745 | 2 | 1 | 1 | 3 | 392 | 44470.09 | 6.06 | 37.32 | 88.78 | -0.45 |
| CN-FMA.chr6.2632 | 12 | 11 | 12 | 2 | 804 | 90333.79 | 6.34 | 41.15 | 95.07 | -0.286 |
| CN-FMA.chr6.2719 | 22 | 21 | 22 | 0 | 770 | 88479.04 | 6.77 | 35.17 | 80.29 | -0.546 |
| CN-FMA.chr6.2734 | 2 | 1 | 2 | 2 | 1049 | 117434.81 | 6.13 | 41.7 | 104.58 | -0.054 |
| CN-FMA.chr6.4269 | 3 | 2 | 3 | 0 | 405 | 45680.31 | 5.77 | 46.34 | 92.67 | -0.311 |
| CN-FMA.chr7.847 | 5 | 4 | 5 | 0 | 354 | 39082.98 | 5.16 | 33.67 | 108.22 | -0.007 |
| CN-FMA.chr7.865 | 10 | 9 | 10 | 0 | 773 | 87956.72 | 5.98 | 46.44 | 83.58 | -0.366 |
| CN-FNG.chr0.49 | 2 | 1 | 1 | 3 | 241 | 27019.36 | 7.57 | 46.17 | 100.66 | -0.145 |
| CN-FNG.chr1.338 | 2 | 1 | 2 | 0 | 365 | 41551.7 | 8.02 | 52.29 | 96.38 | -0.336 |
| CN-FNG.chr3.2186 | 3 | 2 | 3 | 0 | 156 | 17423.86 | 6.43 | 42.21 | 85.51 | -0.508 |
| CN-FNG.chr3.2311 | 1 | 0 | 1 | 0 | 246 | 28710.89 | 6.4 | 36.66 | 97.48 | -0.352 |
| CN-FNG.chr4.2180 | 1 | 0 | 1 | 0 | 346 | 38627.28 | 5.17 | 37.49 | 93.47 | -0.21 |
| CN-FNG.chr4.2529 | 1 | 0 | 1 | 0 | 296 | 33324.44 | 4.99 | 33.7 | 109.59 | -0.102 |
| CN-FNG.chr5.2228 | 1 | 0 | 1 | 0 | 893 | 103211.31 | 7.63 | 51.49 | 99.29 | -0.275 |
| CN-FNG.chr5.2692 | 5 | 4 | 5 | 0 | 1218 | 137476.18 | 5.82 | 42.67 | 103.83 | -0.098 |
| CN-FNG.chr5.2696 | 2 | 1 | 1 | 3 | 373 | 41701.3 | 5.92 | 45.86 | 103.99 | -0.151 |
| CN-FNG.chr6.1541 | 1 | 0 | 1 | 1 | 384 | 43764.37 | 7.58 | 33.14 | 86.04 | -0.515 |
| CN-FNG.chr6.1594 | 5 | 4 | 5 | 2 | 609 | 69245.2 | 6.12 | 41.1 | 100.97 | -0.104 |
| CN-FNG.chr6.3269 | 1 | 0 | 1 | 0 | 457 | 51467.9 | 5.85 | 35.5 | 97.9 | 0.105 |
| CN-FNG.chr7.813 | 2 | 1 | 2 | 0 | 439 | 49790.09 | 6.66 | 51.55 | 93.9 | -0.333 |
| CN-FNU.ctg104.161 | 1 | 0 | 1 | 0 | 337 | 38118.33 | 6.05 | 41.98 | 96.26 | -0.143 |
| CN-FNU.ctg144.27 | 1 | 0 | 1 | 0 | 226 | 25578.63 | 5.99 | 35.77 | 104.38 | -0.099 |
| CN-FNU.ctg158.39 | 1 | 0 | 1 | 2 | 313 | 35497.74 | 6.57 | 35.69 | 100.29 | -0.297 |
| CN-FNU.ctg161.296 | 1 | 0 | 1 | 0 | 387 | 43535.61 | 6.59 | 45.05 | 102.74 | -0.239 |
| CN-FNU.ctg161.301 | 1 | 0 | 1 | 0 | 331 | 36258.52 | 8.69 | 31.16 | 110.97 | 0.086 |
| CN-FNU.ctg20.194 | 1 | 0 | 1 | 0 | 267 | 30224.84 | 6.17 | 36.84 | 99.21 | -0.049 |
| CN-FNU.ctg53.150 | 1 | 0 | 1 | 2 | 430 | 48503.71 | 6.19 | 44.11 | 91.56 | -0.205 |
| CN-FNU.ctg53.159 | 1 | 0 | 1 | 0 | 216 | 23947.61 | 9.32 | 28.88 | 97.55 | -0.26 |
| CN-FNU.ctg53.173 | 6 | 5 | 6 | 1 | 487 | 54933.75 | 6.27 | 45.23 | 93.1 | -0.382 |
| CN-FNU.ctg54.29 | 6 | 5 | 6 | 0 | 633 | 71316.11 | 6.22 | 40.64 | 97.03 | -0.247 |
| CN-FNU.ctg57.114 | 6 | 5 | 6 | 1 | 503 | 56864.97 | 6.16 | 35.33 | 100.76 | -0.153 |
| CN-FNU.ctg63.62 | 3 | 2 | 2 | 3 | 1049 | 117528.05 | 6 | 42.35 | 105.13 | -0.04 |
| IDSequence | Num. of Exon | Num. of Intron | Num. of CDS | Num. of UTR | Num. of Amino Acid | Molecular Weight | Theoretical pI | Instability Index | Aliphatic Index | Grand Average of Hydropathicity |
| CN-FNU.ctg64.1 | 3 | 2 | 2 | 3 | 1049 | 117528.05 | 6 | 42.35 | 105.13 | -0.04 |
| CN-FNU.ctg65.105 | 2 | 1 | 2 | 0 | 413 | 46925.04 | 5.82 | 38.04 | 100.53 | -0.208 |
| CN-FNU.ctg65.11 | 2 | 1 | 2 | 0 | 482 | 54931.88 | 6.03 | 41.02 | 91.39 | -0.333 |
| CN-FNU.ctg70.1 | 2 | 1 | 1 | 3 | 415 | 46972.71 | 6.49 | 42.05 | 81.28 | -0.582 |
| CN-FNU.ctg71.38 | 2 | 1 | 1 | 3 | 415 | 46972.71 | 6.49 | 42.05 | 81.28 | -0.582 |
| CN-FNU.ctg73.111 | 3 | 2 | 3 | 0 | 616 | 70781.6 | 6.67 | 49.44 | 99.33 | -0.232 |
| CN-FNU.ctg84.31 | 2 | 1 | 2 | 0 | 481 | 54257.57 | 5.49 | 38.95 | 96.24 | -0.219 |
| CN-FPE.chr0.885 | 1 | 0 | 1 | 0 | 417 | 47474.37 | 6.45 | 41.3 | 81.1 | -0.616 |
| CN-FPE.chr1.928 | 2 | 1 | 2 | 0 | 558 | 64316.71 | 6.82 | 42.59 | 94.98 | -0.298 |
| CN-FPE.chr2.3023 | 1 | 0 | 1 | 0 | 381 | 43372.93 | 7.1 | 37.34 | 104.86 | -0.119 |
| CN-FPE.chr4.1090 | 2 | 1 | 2 | 0 | 233 | 25936.5 | 6.15 | 31.2 | 90.39 | -0.297 |
| CN-FPE.chr4.1617 | 1 | 0 | 1 | 0 | 449 | 51280.2 | 6.66 | 34.54 | 100.53 | -0.278 |
| CN-FPE.chr4.2668 | 5 | 4 | 5 | 0 | 829 | 94833.98 | 6.89 | 45.47 | 98.6 | -0.307 |
| CN-FPE.chr4.843 | 5 | 4 | 5 | 0 | 365 | 40753.33 | 6.63 | 33.09 | 101.75 | -0.081 |
| CN-FPE.chr5.3016 | 1 | 0 | 1 | 0 | 372 | 41640.27 | 6.58 | 46.29 | 103.23 | -0.174 |
| CN-FPE.chr6.1521 | 2 | 1 | 2 | 0 | 484 | 55345.72 | 6.04 | 48.2 | 102.48 | -0.188 |
| CN-FPE.chr6.1611 | 2 | 1 | 2 | 0 | 361 | 41034.37 | 6.69 | 31.16 | 101.75 | -0.239 |
| CN-FPE.chr6.1820 | 7 | 6 | 7 | 0 | 559 | 63432.09 | 5.94 | 39.08 | 100.61 | -0.203 |
| CN-FPE.chr6.3007 | 3 | 2 | 3 | 1 | 546 | 61607.48 | 5.93 | 33.38 | 101.56 | -0.135 |
| CN-FPE.chr6.3765 | 2 | 1 | 2 | 0 | 483 | 54807.06 | 5.64 | 35.54 | 99.46 | -0.253 |
| CN-FPE.chr6.447 | 11 | 10 | 11 | 1 | 356 | 41265.68 | 4.52 | 43.39 | 75.31 | -0.846 |
| CN-FPE.chr6.453 | 1 | 0 | 1 | 0 | 438 | 49802.17 | 7.63 | 40.9 | 95.46 | -0.274 |
| CN-FPE.chr6.470 | 3 | 2 | 2 | 3 | 1049 | 117480.95 | 5.93 | 42.09 | 105.13 | -0.037 |
| CN-FPE.chr6.878 | 2 | 1 | 2 | 0 | 622 | 71897.29 | 6.72 | 45.2 | 96.03 | -0.246 |
| CN-FPE.chr7.1432 | 8 | 7 | 8 | 0 | 632 | 71962.75 | 6.42 | 36.94 | 98.62 | -0.231 |
| CN-FPE.chr7.2093 | 2 | 1 | 1 | 3 | 372 | 41640.27 | 6.58 | 46.29 | 103.23 | -0.174 |
| CN-FPE.chr7.728 | 3 | 2 | 3 | 0 | 544 | 62321.48 | 7.99 | 50.14 | 95.85 | -0.271 |
| CN-FvH4.1g02740.1 | 1 | 0 | 1 | 0 | 823 | 91890.6 | 5.58 | 43.33 | 102.93 | -0.077 |
| CN-FvH4.1g18160.1 | 6 | 5 | 4 | 4 | 387 | 43550.54 | 6.09 | 40.91 | 100.41 | -0.064 |
| CN-FvH4.2g05600.1 | 5 | 4 | 5 | 0 | 1012 | 114859.61 | 7.55 | 46.27 | 89.58 | -0.271 |
| CN-FvH4.2g05720.1 | 11 | 10 | 11 | 1 | 1132 | 125509.83 | 8.3 | 45.26 | 82.39 | -0.32 |
| CN-FvH4.2g06550.1 | 4 | 3 | 3 | 2 | 455 | 51349.85 | 8.39 | 47.33 | 97.05 | -0.279 |
| CN-FvH4.2g06640.1 | 4 | 3 | 4 | 0 | 561 | 64283.76 | 5.76 | 42.4 | 112.21 | 0.018 |
| CN-FvH4.2g36850.1 | 3 | 2 | 3 | 2 | 1078 | 121440.75 | 6.04 | 44.22 | 97.02 | -0.204 |
| CN-FvH4.3g16720.1 | 3 | 2 | 3 | 0 | 770 | 87137.35 | 7 | 40.3 | 97.62 | -0.284 |
| CN-FvH4.3g21750.1 | 3 | 2 | 3 | 0 | 432 | 49476.31 | 7.15 | 55.72 | 94.81 | -0.325 |
| CN-FvH4.5g23460.1 | 2 | 1 | 2 | 0 | 426 | 48747.25 | 6.98 | 38.47 | 93.15 | -0.415 |
| CN-FvH4.5g24290.1 | 3 | 2 | 3 | 0 | 401 | 45361.51 | 8.03 | 42.18 | 96.03 | -0.233 |
| CN-FvH4.5g24310.1 | 1 | 0 | 1 | 0 | 232 | 27059.09 | 8.81 | 57.87 | 88.58 | -0.572 |
| CN-FvH4.6g02790.1 | 2 | 1 | 2 | 0 | 589 | 65912.95 | 5.31 | 36.98 | 101.97 | -0.176 |
| CN-FvH4.6g15230.1 | 1 | 0 | 1 | 0 | 374 | 41625.17 | 6.17 | 36.42 | 105.05 | -0.116 |
| IDSequence | Num. of Exon | Num. of Intron | Num. of CDS | Num. of UTR | Num. of Amino Acid | Molecular Weight | Theoretical pI | Instability Index | Aliphatic Index | Grand Average of Hydropathicity |
| CN-FvH4.6g22150.1 | 3 | 2 | 1 | 4 | 392 | 44526.2 | 6.17 | 37.79 | 88.78 | -0.452 |
| CN-FvH4.6g29230.1 | 11 | 10 | 11 | 2 | 780 | 87708.86 | 6.34 | 42.07 | 95.5 | -0.27 |
| CN-FvH4.6g29690.1 | 2 | 1 | 2 | 0 | 369 | 41652.88 | 7.63 | 35.09 | 94.55 | -0.202 |
| CN-FvH4.6g51570.1 | 2 | 1 | 1 | 2 | 883 | 100295.94 | 5.98 | 45.12 | 100.23 | -0.228 |
| CN-FvH4.7g02430.1 | 1 | 0 | 1 | 0 | 317 | 35685.7 | 5.82 | 27.03 | 91.58 | -0.274 |
| CN-FvH4.7g11820.1 | 3 | 2 | 3 | 0 | 367 | 40836.39 | 7.14 | 35.08 | 109.18 | -0.036 |
| CN-FvH4.7g23850.1 | 1 | 0 | 1 | 0 | 404 | 45962.26 | 6.03 | 45.08 | 99.83 | -0.227 |
| CN-FvH4.7g31270.1 | 1 | 0 | 1 | 0 | 528 | 60538.73 | 6.25 | 37.65 | 96.17 | -0.298 |
| CN-FVI.CHR2.1419 | 3 | 2 | 2 | 3 | 1049 | 117580.14 | 6.2 | 41.8 | 104.39 | -0.06 |
| CN-FVI.CHR2.1513 | 1 | 0 | 1 | 2 | 364 | 41177.55 | 6.53 | 36.81 | 100.41 | -0.209 |
| CN-FVI.CHR2.1517 | 2 | 1 | 2 | 0 | 480 | 56145.59 | 5.85 | 50.18 | 95.58 | -0.274 |
| CN-FVI.CHR4.1436 | 1 | 0 | 1 | 2 | 333 | 38452.26 | 6.64 | 34.62 | 94.23 | -0.345 |
| CN-FVI.CHR5.3012 | 2 | 1 | 1 | 3 | 371 | 41541 | 6.1 | 52.82 | 100.62 | -0.24 |
| CN-FVI.CHR6.1776 | 2 | 1 | 2 | 0 | 491 | 55614.12 | 5.93 | 41.07 | 93.16 | -0.271 |
| CN-FVI.CHR6.2188 | 2 | 1 | 1 | 3 | 424 | 48308.38 | 6.66 | 42.39 | 81.16 | -0.616 |
| CN-FVI.CHR6.2635 | 12 | 11 | 12 | 2 | 867 | 97210.46 | 5.58 | 39.79 | 93.52 | -0.255 |
| CN-FVI.CHR7.3759 | 1 | 0 | 1 | 0 | 255 | 28631.2 | 8.84 | 36.8 | 95.65 | -0.179 |
| CNL-FDA.chr1.06545 | 3 | 2 | 3 | 0 | 1219 | 138659.02 | 6.13 | 50.75 | 103.26 | -0.147 |
| CNL-FDA.chr1.22189 | 4 | 3 | 4 | 0 | 638 | 73320.13 | 5.25 | 49.69 | 110.27 | -0.053 |
| CNL-FDA.chr2.12098 | 9 | 8 | 9 | 0 | 1073 | 123313.9 | 8.38 | 41.24 | 99.24 | -0.115 |
| CNL-FDA.chr2.12208 | 3 | 2 | 3 | 0 | 1299 | 146752.72 | 5.77 | 46.82 | 95.65 | -0.239 |
| CNL-FDA.chr2.12209 | 3 | 2 | 3 | 0 | 931 | 105800.85 | 6.05 | 50.74 | 96.52 | -0.239 |
| CNL-FDA.chr2.12218 | 2 | 1 | 2 | 0 | 1289 | 144918.96 | 6.2 | 46.41 | 98.6 | -0.205 |
| CNL-FDA.chr2.17966 | 1 | 0 | 1 | 0 | 433 | 49115.01 | 5.02 | 57.55 | 102.24 | -0.174 |
| CNL-FDA.chr2.19060 | 1 | 0 | 1 | 0 | 781 | 88762.04 | 5.81 | 46.11 | 103.6 | -0.125 |
| CNL-FDA.chr3.05415 | 4 | 3 | 4 | 0 | 885 | 101496.82 | 6.75 | 38.67 | 101.51 | -0.158 |
| CNL-FDA.chr3.05890 | 3 | 2 | 3 | 0 | 503 | 57327.24 | 6.8 | 53.07 | 91.87 | -0.226 |
| CNL-FDA.chr3.05895 | 2 | 1 | 2 | 0 | 879 | 100356.11 | 5.61 | 43.07 | 102.58 | -0.156 |
| CNL-FDA.chr3.09595 | 6 | 5 | 6 | 0 | 1418 | 161733.45 | 7.62 | 44.26 | 102.94 | -0.14 |
| CNL-FDA.chr3.15906 | 5 | 4 | 5 | 0 | 796 | 91058.3 | 7.61 | 47.6 | 102 | -0.263 |
| CNL-FDA.chr3.16699 | 2 | 1 | 2 | 0 | 961 | 110708.4 | 5.91 | 47.34 | 102.63 | -0.182 |
| CNL-FDA.chr3.16968 | 1 | 0 | 1 | 0 | 768 | 87036.71 | 5.97 | 37.02 | 102.57 | -0.26 |
| CNL-FDA.chr3.16978 | 3 | 2 | 3 | 0 | 742 | 83995.37 | 6.95 | 30.28 | 103.65 | -0.234 |
| CNL-FDA.chr3.23062 | 2 | 1 | 2 | 0 | 869 | 98769.41 | 5.95 | 45.66 | 94.89 | -0.214 |
| CNL-FDA.chr3.25684 | 4 | 3 | 4 | 0 | 579 | 65900.99 | 9.04 | 47.74 | 106.91 | -0.119 |
| CNL-FDA.chr4.11333 | 14 | 13 | 14 | 0 | 3061 | 343181.57 | 5.3 | 47.98 | 101.78 | -0.169 |
| CNL-FDA.chr4.24588 | 1 | 0 | 1 | 0 | 810 | 91845.39 | 7.26 | 46.16 | 100.12 | -0.14 |
| CNL-FDA.chr5.03688 | 4 | 3 | 4 | 0 | 971 | 112011.49 | 6.73 | 41.88 | 101.16 | -0.214 |
| CNL-FDA.chr5.07881 | 4 | 3 | 4 | 0 | 1018 | 116354.28 | 7.84 | 45.21 | 103.96 | -0.147 |
| CNL-FDA.chr5.22369 | 7 | 6 | 7 | 0 | 1676 | 189323.3 | 6.05 | 42.98 | 102.73 | -0.12 |
| CNL-FDA.chr5.23812 | 8 | 7 | 8 | 0 | 1764 | 198996.11 | 5.6 | 44.91 | 100.51 | -0.159 |
| CNL-FDA.chr5.24059 | 3 | 2 | 3 | 0 | 915 | 103857.81 | 5.42 | 37.7 | 102.16 | -0.124 |
| IDSequence | Num. of Exon | Num. of Intron | Num. of CDS | Num. of UTR | Num. of Amino Acid | Molecular Weight | Theoretical pI | Instability Index | Aliphatic Index | Grand Average of Hydropathicity |
| CNL-FDA.chr5.24089 | 12 | 11 | 12 | 0 | 1883 | 210580.19 | 5.3 | 48.86 | 101.45 | -0.188 |
| CNL-FDA.chr5.24311 | 13 | 12 | 13 | 0 | 1723 | 195456.62 | 6.21 | 41.59 | 90.85 | -0.293 |
| CNL-FDA.chr5.24966 | 1 | 0 | 1 | 0 | 943 | 107711.4 | 8.62 | 43.66 | 104.31 | -0.212 |
| CNL-FDA.chr5.25044 | 2 | 1 | 2 | 0 | 948 | 107693.6 | 8.83 | 43.57 | 107.94 | -0.134 |
| CNL-FDA.chr5.25047 | 1 | 0 | 1 | 0 | 948 | 108298.13 | 8.68 | 43.38 | 109.05 | -0.16 |
| CNL-FDA.chr5.25049 | 2 | 1 | 2 | 0 | 954 | 109268.93 | 9 | 45.13 | 104.29 | -0.21 |
| CNL-FDA.chr5.25050 | 1 | 0 | 1 | 0 | 952 | 109137.86 | 8.53 | 37.28 | 101.43 | -0.229 |
| CNL-FDA.chr6.02010 | 1 | 0 | 1 | 0 | 902 | 102671.83 | 5.39 | 42.8 | 100.04 | -0.274 |
| CNL-FDA.chr6.02056 | 1 | 0 | 1 | 0 | 946 | 108325.21 | 5.95 | 43.08 | 100.92 | -0.214 |
| CNL-FDA.chr6.02058 | 3 | 2 | 3 | 0 | 920 | 105550.86 | 6.15 | 43.93 | 98.6 | -0.251 |
| CNL-FDA.chr6.02198 | 2 | 1 | 2 | 0 | 1478 | 166714.7 | 5.84 | 44.71 | 102.46 | -0.155 |
| CNL-FDA.chr6.02459 | 3 | 2 | 3 | 0 | 476 | 54285.9 | 6.14 | 42.96 | 100.95 | -0.172 |
| CNL-FDA.chr6.13546 | 4 | 3 | 4 | 0 | 744 | 84432.11 | 5.75 | 50.99 | 99.44 | -0.213 |
| CNL-FDA.chr6.13552 | 4 | 3 | 4 | 0 | 912 | 102527.1 | 5.84 | 44.73 | 99.74 | -0.14 |
| CNL-FDA.chr6.13561 | 3 | 2 | 3 | 0 | 1194 | 135550.54 | 5.86 | 45.59 | 100.9 | -0.184 |
| CNL-FDA.chr6.13872 | 3 | 2 | 3 | 0 | 977 | 112441.95 | 6.12 | 46.54 | 101.89 | -0.271 |
| CNL-FDA.chr6.13908 | 2 | 1 | 2 | 0 | 851 | 96962.09 | 6.07 | 41.99 | 101.56 | -0.188 |
| CNL-FDA.chr6.13967 | 1 | 0 | 1 | 0 | 965 | 110766.92 | 7.71 | 42.99 | 100.34 | -0.278 |
| CNL-FDA.chr6.14104 | 6 | 5 | 6 | 0 | 1768 | 199548.69 | 6.14 | 39.15 | 99.55 | -0.203 |
| CNL-FDA.chr6.17311 | 6 | 5 | 6 | 0 | 751 | 86031.78 | 8.76 | 35.09 | 100.09 | -0.241 |
| CNL-FDA.chr6.26146 | 10 | 9 | 10 | 0 | 1751 | 193987.64 | 8.22 | 46.1 | 88.17 | -0.383 |
| CNL-FDA.chr6.26153 | 6 | 5 | 6 | 0 | 1164 | 131396.65 | 7.51 | 44.16 | 103 | -0.119 |
| CNL-FDA.chr6.26337 | 2 | 1 | 2 | 0 | 1353 | 152464.04 | 7.43 | 51.63 | 103.32 | -0.138 |
| CNL-FDA.chr7.00307 | 14 | 13 | 14 | 0 | 1757 | 198501.56 | 6.46 | 43.06 | 98.86 | -0.189 |
| CNL-FDA.chr7.00809 | 1 | 0 | 1 | 0 | 719 | 82711.59 | 6.2 | 40.67 | 101.2 | -0.267 |
| CNL-FDA.chr7.01119 | 2 | 1 | 2 | 0 | 944 | 108075.02 | 6.11 | 41.84 | 103.09 | -0.207 |
| CNL-FDA.chr7.01192 | 1 | 0 | 1 | 0 | 919 | 104805.72 | 6.25 | 41.96 | 107.61 | -0.123 |
| CNL-FDA.chr7.01250 | 3 | 2 | 3 | 0 | 943 | 109179.17 | 5.98 | 49.62 | 105.12 | -0.293 |
| CNL-FDA.chr7.01259 | 2 | 1 | 2 | 0 | 844 | 97292.37 | 6.44 | 50.18 | 102.43 | -0.278 |
| CNL-FDA.chr7.01379 | 4 | 3 | 4 | 0 | 590 | 68283.5 | 9.24 | 46.22 | 97.88 | -0.302 |
| CNL-FDA.chr7.20574 | 2 | 1 | 2 | 0 | 915 | 103882.7 | 5.33 | 50.51 | 100.44 | -0.133 |
| CNL-FII.chr1.1071 | 1 | 0 | 1 | 0 | 841 | 95421.23 | 6.38 | 43.17 | 99.86 | -0.148 |
| CNL-FII.chr1.1471 | 7 | 6 | 7 | 0 | 781 | 89371.52 | 6.65 | 46.68 | 105.8 | -0.134 |
| CNL-FII.chr2.1235 | 3 | 2 | 1 | 4 | 958 | 111230.67 | 8.96 | 49.36 | 102.91 | -0.171 |
| CNL-FII.chr3.1384 | 1 | 0 | 1 | 0 | 880 | 99733.26 | 6.61 | 42.12 | 101.58 | -0.192 |
| CNL-FII.chr3.1385 | 2 | 1 | 1 | 2 | 932 | 105891.61 | 8.59 | 37.07 | 102.4 | -0.228 |
| CNL-FII.chr3.1811 | 2 | 1 | 2 | 2 | 898 | 102432.46 | 6.32 | 42.26 | 105.39 | -0.047 |
| CNL-FII.chr3.408 | 9 | 8 | 6 | 5 | 1791 | 202950.26 | 5.87 | 41.56 | 98.95 | -0.207 |
| CNL-FII.chr3.77 | 4 | 3 | 2 | 4 | 867 | 98305.17 | 6.94 | 45.3 | 97.14 | -0.203 |
| CNL-FII.chr3.951 | 1 | 0 | 1 | 0 | 921 | 104853.92 | 8.78 | 49.27 | 104.78 | -0.165 |
| CNL-FII.chr3.953 | 1 | 0 | 1 | 0 | 920 | 105059.89 | 7.84 | 47.71 | 102.02 | -0.198 |
| CNL-FII.chr3.954 | 2 | 1 | 1 | 2 | 923 | 105115.83 | 8.56 | 47.33 | 105.58 | -0.072 |
| IDSequence | Num. of Exon | Num. of Intron | Num. of CDS | Num. of UTR | Num. of Amino Acid | Molecular Weight | Theoretical pI | Instability Index | Aliphatic Index | Grand Average of Hydropathicity |
| CNL-FII.chr3.956 | 1 | 0 | 1 | 0 | 919 | 104993.44 | 8.8 | 50.69 | 102.76 | -0.172 |
| CNL-FII.chr4.1300 | 1 | 0 | 1 | 2 | 998 | 113201.98 | 6.27 | 43.31 | 103.03 | -0.158 |
| CNL-FII.chr4.1808 | 3 | 2 | 3 | 0 | 1298 | 148077.02 | 6.7 | 46.79 | 94.46 | -0.313 |
| CNL-FII.chr4.1849.2 | 3 | 2 | 2 | 2 | 926 | 106428.93 | 8.17 | 44.64 | 100.05 | -0.148 |
| CNL-FII.chr4.2076 | 3 | 2 | 2 | 3 | 980 | 110698.86 | 5.85 | 44.03 | 103.53 | -0.094 |
| CNL-FII.chr4.2124 | 1 | 0 | 1 | 1 | 730 | 83962.15 | 6 | 45.91 | 96.36 | -0.248 |
| CNL-FII.chr4.377 | 7 | 6 | 6 | 3 | 1867 | 208395.8 | 5.09 | 49.24 | 101.59 | -0.199 |
| CNL-FII.chr5.2001 | 1 | 0 | 1 | 0 | 894 | 102762.1 | 9 | 36.09 | 106.82 | -0.135 |
| CNL-FII.chr5.2002 | 2 | 1 | 1 | 3 | 955 | 109517.38 | 8.96 | 39.28 | 101.03 | -0.25 |
| CNL-FII.chr5.2003 | 1 | 0 | 1 | 1 | 952 | 109155 | 8.68 | 37.31 | 101.63 | -0.234 |
| CNL-FII.chr5.2004 | 2 | 1 | 2 | 2 | 954 | 109334.98 | 9.04 | 46.82 | 104.29 | -0.215 |
| CNL-FII.chr5.2006 | 1 | 0 | 1 | 0 | 944 | 108135.18 | 8.9 | 43.25 | 109.83 | -0.149 |
| CNL-FII.chr5.2007 | 3 | 2 | 3 | 1 | 817 | 93741.11 | 6.78 | 45.95 | 103.07 | -0.175 |
| CNL-FII.chr5.2008 | 1 | 0 | 1 | 0 | 948 | 107649.68 | 8.72 | 42.98 | 108.67 | -0.118 |
| CNL-FII.chr5.3003 | 3 | 2 | 1 | 4 | 952 | 107626.43 | 6.08 | 34.16 | 102.59 | -0.127 |
| CNL-FII.chr5.3063 | 1 | 0 | 1 | 1 | 984 | 113012.41 | 8.65 | 37.05 | 100.3 | -0.247 |
| CNL-FII.chr6.2256 | 5 | 4 | 1 | 6 | 1151 | 131483.92 | 5.56 | 46.5 | 106.49 | -0.17 |
| CNL-FII.chr6.2546 | 5 | 4 | 1 | 6 | 1151 | 131483.92 | 5.56 | 46.5 | 106.49 | -0.17 |
| CNL-FII.chr6.4055 | 1 | 0 | 1 | 0 | 909 | 102909.45 | 5.77 | 44.87 | 95.76 | -0.171 |
| CNL-FII.chr7.1027 | 5 | 4 | 3 | 3 | 1306 | 147314.59 | 7.17 | 46.05 | 106.13 | -0.032 |
| CNL-FII.chr7.162.3 | 3 | 2 | 2 | 3 | 1449 | 164099.99 | 5.66 | 46.44 | 101.53 | -0.16 |
| CNL-FII.chr7.186 | 3 | 2 | 2 | 2 | 631 | 70872.58 | 8.89 | 42.55 | 89.08 | -0.301 |
| CNL-FII.chr7.206 | 1 | 0 | 1 | 0 | 1359 | 153652.11 | 6.42 | 48.2 | 101.27 | -0.089 |
| CNL-FII.chr7.207 | 4 | 3 | 1 | 5 | 1365 | 153619.53 | 6.34 | 46.16 | 99.02 | -0.149 |
| CNL-FII.chr7.210 | 6 | 5 | 1 | 6 | 1337 | 151052.89 | 7.31 | 43.83 | 101.31 | -0.127 |
| CNL-FII.chr7.257 | 1 | 0 | 1 | 2 | 1445 | 163858.95 | 5.94 | 55.5 | 102.38 | -0.098 |
| CNL-FII.chr7.55 | 1 | 0 | 1 | 1 | 1352 | 153328.15 | 6.85 | 47.46 | 99.63 | -0.153 |
| CNL-FII.chr7.708 | 4 | 3 | 2 | 4 | 962 | 109024.76 | 6.33 | 49.86 | 102.75 | -0.129 |
| CNL-FII.chr7.789 | 6 | 5 | 5 | 3 | 999 | 114054.73 | 5.8 | 49.59 | 100.1 | -0.18 |
| CNL-FII.chr7.998 | 1 | 0 | 1 | 0 | 1060 | 121557.01 | 5.93 | 46.81 | 97.64 | -0.204 |
| CNL-FMA.chr1.1369 | 2 | 1 | 1 | 3 | 841 | 95304.18 | 6.56 | 42.71 | 100.44 | -0.145 |
| CNL-FMA.chr2.328 | 3 | 2 | 3 | 0 | 633 | 71529.75 | 6.34 | 47.31 | 101.5 | -0.122 |
| CNL-FMA.chr3.1344 | 4 | 3 | 4 | 0 | 916 | 104058.55 | 8.44 | 38.29 | 102.27 | -0.22 |
| CNL-FMA.chr3.2744 | 3 | 2 | 3 | 1 | 1054 | 120886.63 | 6.22 | 47.4 | 94.54 | -0.237 |
| CNL-FMA.chr3.2975 | 3 | 2 | 3 | 0 | 987 | 111926.03 | 7.03 | 51.24 | 101.75 | -0.109 |
| CNL-FMA.chr3.75 | 2 | 1 | 2 | 1 | 871 | 98761.65 | 6.61 | 44.43 | 97.03 | -0.206 |
| CNL-FMA.chr4.2118 | 3 | 2 | 2 | 3 | 1004 | 113764.92 | 6.44 | 41.77 | 101.18 | -0.144 |
| CNL-FMA.chr4.2163 | 1 | 0 | 1 | 0 | 762 | 87514.91 | 5.54 | 49.03 | 97.8 | -0.269 |
| CNL-FMA.chr4.679 | 7 | 6 | 7 | 1 | 1723 | 193341.57 | 5.4 | 44.78 | 100.13 | -0.198 |
| CNL-FMA.chr5.1324 | 1 | 0 | 1 | 0 | 948 | 107470.76 | 7.21 | 41.04 | 102.78 | -0.198 |
| CNL-FMA.chr5.1739 | 10 | 9 | 10 | 0 | 1760 | 200093.44 | 7.85 | 44.32 | 94.85 | -0.217 |
| CNL-FMA.chr5.2388 | 6 | 5 | 6 | 0 | 1627 | 183621.79 | 6.09 | 44.83 | 102.65 | -0.092 |
| IDSequence | Num. of Exon | Num. of Intron | Num. of CDS | Num. of UTR | Num. of Amino Acid | Molecular Weight | Theoretical pI | Instability Index | Aliphatic Index | Grand Average of Hydropathicity |
| CNL-FMA.chr6.1184 | 4 | 3 | 2 | 4 | 1315 | 148293.93 | 6.6 | 48.9 | 103.57 | -0.139 |
| CNL-FMA.chr6.1508 | 5 | 4 | 3 | 4 | 1292 | 145531.45 | 6.08 | 44.08 | 102.01 | -0.149 |
| CNL-FMA.chr6.1658 | 8 | 7 | 8 | 1 | 1467 | 166587.07 | 6.44 | 53.1 | 104.31 | -0.139 |
| CNL-FMA.chr6.2166 | 3 | 2 | 3 | 0 | 1155 | 131071.36 | 6.57 | 41.3 | 99.11 | -0.238 |
| CNL-FMA.chr6.2723 | 3 | 2 | 3 | 0 | 1274 | 144124.1 | 5.44 | 49.94 | 94.4 | -0.319 |
| CNL-FMA.chr6.3395 | 1 | 0 | 1 | 0 | 1289 | 147472.85 | 7.59 | 44.12 | 98.84 | -0.247 |
| CNL-FMA.chr7.880 | 1 | 0 | 1 | 0 | 1124 | 128707.45 | 5.91 | 48.09 | 98.51 | -0.202 |
| CNL-FNG.chr0.411 | 5 | 4 | 4 | 3 | 1348 | 153379.15 | 7.46 | 42.23 | 105.39 | -0.1 |
| CNL-FNG.chr1.177 | 3 | 2 | 1 | 4 | 866 | 98522.66 | 7.7 | 36.04 | 102.99 | -0.141 |
| CNL-FNG.chr1.545 | 3 | 2 | 3 | 0 | 936 | 106617.33 | 8.48 | 43.94 | 99.87 | -0.261 |
| CNL-FNG.chr2.1430 | 2 | 1 | 2 | 1 | 1244 | 139134.23 | 5.53 | 42.4 | 101.06 | -0.211 |
| CNL-FNG.chr2.416 | 8 | 7 | 7 | 2 | 1141 | 129337.25 | 8.54 | 43.7 | 99.95 | -0.154 |
| CNL-FNG.chr2.482 | 3 | 2 | 1 | 4 | 977 | 110952.8 | 5.79 | 45.93 | 104.85 | -0.103 |
| CNL-FNG.chr3.1370 | 2 | 1 | 2 | 0 | 794 | 90039.12 | 6.92 | 40.57 | 101.31 | -0.181 |
| CNL-FNG.chr3.1371 | 5 | 4 | 5 | 0 | 908 | 103022.69 | 8.22 | 38.11 | 98.04 | -0.282 |
| CNL-FNG.chr3.2531 | 9 | 8 | 9 | 1 | 1567 | 178050.68 | 8.47 | 48.14 | 99.04 | -0.105 |
| CNL-FNG.chr3.84 | 6 | 5 | 6 | 0 | 1005 | 113546.01 | 5.92 | 40.85 | 93.12 | -0.231 |
| CNL-FNG.chr4.1917 | 4 | 3 | 4 | 1 | 799 | 91949.61 | 8.5 | 43.89 | 102.65 | -0.189 |
| CNL-FNG.chr4.2225 | 1 | 0 | 1 | 1 | 724 | 83307.35 | 5.97 | 45.32 | 97.69 | -0.253 |
| CNL-FNG.chr5.1760 | 5 | 4 | 3 | 3 | 1103 | 124901.6 | 6.46 | 40.56 | 101.6 | -0.203 |
| CNL-FNG.chr5.2089 | 2 | 1 | 1 | 3 | 941 | 108127.01 | 8.47 | 40.47 | 103.26 | -0.201 |
| CNL-FNG.chr5.2145 | 2 | 1 | 1 | 3 | 955 | 109453.24 | 9.01 | 39.96 | 101.95 | -0.246 |
| CNL-FNG.chr5.2146 | 1 | 0 | 1 | 2 | 952 | 109336.13 | 8.64 | 38.12 | 101.32 | -0.24 |
| CNL-FNG.chr5.2147 | 2 | 1 | 2 | 2 | 954 | 109287.85 | 8.94 | 46.49 | 103.97 | -0.218 |
| CNL-FNG.chr5.2149 | 1 | 0 | 1 | 2 | 948 | 108345.23 | 8.5 | 42.58 | 109.98 | -0.139 |
| CNL-FNG.chr5.2150 | 1 | 0 | 1 | 2 | 948 | 107745.63 | 8.83 | 42.67 | 107.94 | -0.14 |
| CNL-FNG.chr5.2227 | 1 | 0 | 1 | 2 | 943 | 107591.27 | 8.98 | 42.39 | 105.12 | -0.218 |
| CNL-FNG.chr5.2999 | 1 | 0 | 1 | 0 | 775 | 88061.82 | 6.19 | 37.1 | 100.85 | -0.15 |
| CNL-FNG.chr5.3271 | 2 | 1 | 2 | 0 | 621 | 70663.22 | 6.29 | 40.78 | 97.33 | -0.105 |
| CNL-FNG.chr5.654 | 6 | 5 | 6 | 0 | 1316 | 147846.64 | 6.34 | 44.76 | 91.16 | -0.194 |
| CNL-FNG.chr5.655 | 1 | 0 | 1 | 0 | 892 | 101034.78 | 6.04 | 43.04 | 96.48 | -0.16 |
| CNL-FNG.chr6.2058 | 3 | 2 | 3 | 2 | 1294 | 146098.33 | 5.42 | 48.78 | 94.06 | -0.298 |
| CNL-FNG.chr6.2406 | 2 | 1 | 2 | 0 | 596 | 67646.25 | 6.06 | 39.11 | 99.93 | -0.216 |
| CNL-FNG.chr6.2420 | 3 | 2 | 3 | 0 | 1338 | 150909.36 | 6.49 | 51.72 | 101.73 | -0.111 |
| CNL-FNG.chr6.2431 | 5 | 4 | 5 | 0 | 787 | 89412.29 | 7.18 | 45.52 | 100.53 | -0.111 |
| CNL-FNG.chr7.204 | 1 | 0 | 1 | 2 | 1445 | 163848.91 | 5.96 | 55.36 | 100.83 | -0.113 |
| CNL-FNG.chr7.951 | 8 | 7 | 3 | 7 | 1306 | 147399.62 | 7.02 | 44.88 | 105.83 | -0.041 |
| CNL-FNU.ctg104.183 | 2 | 1 | 2 | 0 | 473 | 53494.62 | 5.39 | 51.26 | 106.79 | -0.094 |
| CNL-FNU.ctg104.299 | 3 | 2 | 1 | 4 | 948 | 107568.83 | 5.91 | 47.61 | 104.77 | -0.127 |
| CNL-FNU.ctg114.210 | 4 | 3 | 3 | 2 | 1009 | 115682.54 | 6.6 | 50.95 | 98.9 | -0.264 |
| CNL-FNU.ctg129.61 | 4 | 3 | 1 | 4 | 933 | 107396.71 | 6.1 | 42.85 | 106.85 | -0.186 |
| CNL-FNU.ctg132.293 | 4 | 3 | 4 | 1 | 652 | 73562.35 | 6.18 | 38.04 | 105.86 | -0.119 |
| IDSequence | Num. of Exon | Num. of Intron | Num. of CDS | Num. of UTR | Num. of Amino Acid | Molecular Weight | Theoretical pI | Instability Index | Aliphatic Index | Grand Average of Hydropathicity |
| CNL-FNU.ctg134.171 | 2 | 1 | 2 | 1 | 1135 | 128925.05 | 6.03 | 55 | 96.53 | -0.212 |
| CNL-FNU.ctg145.8 | 1 | 0 | 1 | 2 | 1445 | 163809.94 | 5.94 | 54.89 | 101.98 | -0.1 |
| CNL-FNU.ctg146.100 | 5 | 4 | 2 | 5 | 1374 | 156062.31 | 6.14 | 50.33 | 98.76 | -0.167 |
| CNL-FNU.ctg147.19 | 2 | 1 | 1 | 3 | 949 | 107376.94 | 8.36 | 40.38 | 102.88 | -0.186 |
| CNL-FNU.ctg150.57 | 6 | 5 | 6 | 1 | 1599 | 181300.46 | 6.08 | 43.3 | 91.48 | -0.271 |
| CNL-FNU.ctg154.468 | 1 | 0 | 1 | 0 | 868 | 98742.44 | 6.44 | 46.35 | 102.91 | -0.19 |
| CNL-FNU.ctg155.369 | 2 | 1 | 2 | 1 | 706 | 81109.62 | 8.8 | 39.18 | 104.75 | -0.196 |
| CNL-FNU.ctg156.8 | 2 | 1 | 2 | 1 | 706 | 81107.65 | 8.8 | 39.53 | 105.03 | -0.192 |
| CNL-FNU.ctg158.148 | 1 | 0 | 1 | 1 | 943 | 107726.42 | 8.68 | 43.66 | 103.89 | -0.221 |
| CNL-FNU.ctg158.149 | 1 | 0 | 1 | 1 | 892 | 103110.7 | 8.63 | 48.5 | 98.97 | -0.27 |
| CNL-FNU.ctg158.40 | 1 | 0 | 1 | 0 | 955 | 109398.18 | 8.98 | 39.24 | 100.92 | -0.256 |
| CNL-FNU.ctg158.42 | 1 | 0 | 1 | 1 | 952 | 109121.83 | 8.51 | 37.14 | 100.91 | -0.231 |
| CNL-FNU.ctg158.43 | 3 | 2 | 2 | 3 | 954 | 109372.05 | 8.97 | 45.54 | 104.29 | -0.214 |
| CNL-FNU.ctg158.45 | 1 | 0 | 1 | 0 | 877 | 100161.91 | 9.02 | 43.17 | 109.56 | -0.138 |
| CNL-FNU.ctg158.46 | 6 | 5 | 6 | 0 | 800 | 91256.27 | 8.61 | 44.06 | 97.96 | -0.301 |
| CNL-FNU.ctg158.48 | 1 | 0 | 1 | 2 | 784 | 89274.18 | 8.98 | 42.82 | 108.52 | -0.132 |
| CNL-FNU.ctg165.171 | 2 | 1 | 1 | 3 | 930 | 106007.78 | 8.83 | 39 | 101.24 | -0.277 |
| CNL-FNU.ctg169.439 | 2 | 1 | 1 | 3 | 841 | 95400 | 6.25 | 45.16 | 99.05 | -0.17 |
| CNL-FNU.ctg170.24 | 2 | 1 | 1 | 3 | 841 | 95366.04 | 6.38 | 44.06 | 99.05 | -0.163 |
| CNL-FNU.ctg172.27 | 6 | 5 | 6 | 1 | 734 | 83384.36 | 5.53 | 39.33 | 101.69 | -0.159 |
| CNL-FNU.ctg172.55 | 12 | 11 | 12 | 0 | 1297 | 145390.24 | 5.65 | 46.53 | 94.23 | -0.282 |
| CNL-FNU.ctg21.293 | 3 | 2 | 3 | 2 | 991 | 112340.46 | 7.64 | 49.97 | 101.74 | -0.119 |
| CNL-FNU.ctg30.47 | 1 | 0 | 1 | 1 | 888 | 100547.34 | 6.61 | 42.67 | 102.12 | -0.169 |
| CNL-FNU.ctg47.220 | 2 | 1 | 2 | 0 | 871 | 98771.67 | 6.38 | 43.94 | 96.02 | -0.21 |
| CNL-FNU.ctg48.6 | 2 | 1 | 2 | 1 | 865 | 98039.46 | 6.69 | 43.53 | 102.13 | -0.186 |
| CNL-FNU.ctg53.139 | 3 | 2 | 3 | 1 | 1243 | 141006.99 | 6.08 | 44.75 | 101.09 | -0.178 |
| CNL-FNU.ctg53.160 | 4 | 3 | 4 | 0 | 742 | 84950.68 | 5.38 | 52.71 | 98.13 | -0.296 |
| CNL-FNU.ctg53.185 | 4 | 3 | 4 | 0 | 712 | 80713.75 | 6.24 | 41.83 | 95.45 | -0.37 |
| CNL-FNU.ctg53.289 | 3 | 2 | 3 | 0 | 779 | 88226.25 | 5.97 | 40.17 | 95.74 | -0.319 |
| CNL-FNU.ctg53.85 | 3 | 2 | 3 | 1 | 695 | 78994.07 | 6.89 | 41.08 | 97.76 | -0.324 |
| CNL-FNU.ctg64.14.1 | 3 | 2 | 2 | 2 | 980 | 112192.76 | 7.35 | 42.3 | 97.98 | -0.262 |
| CNL-FNU.ctg78.2 | 5 | 4 | 3 | 4 | 1219 | 137362.37 | 6.22 | 48.83 | 103.65 | -0.127 |
| CNL-FNU.ctg81.894 | 1 | 0 | 1 | 0 | 1077 | 122619.26 | 6.11 | 44.74 | 99.08 | -0.217 |
| CNL-FNU.ctg82.313 | 2 | 1 | 2 | 2 | 1002 | 113252.14 | 6.26 | 46.32 | 99.9 | -0.163 |
| CNL-FNU.ctg82.648 | 5 | 4 | 3 | 2 | 1021 | 117441.75 | 6.66 | 47.17 | 99.61 | -0.224 |
| CNL-FNU.ctg84.614 | 4 | 3 | 4 | 0 | 504 | 57232.17 | 8.38 | 47.01 | 98.63 | -0.315 |
| CNL-FPE.chr0.563 | 2 | 1 | 2 | 0 | 931 | 105605.25 | 6.83 | 46.84 | 104.2 | -0.107 |
| CNL-FPE.chr1.1624 | 3 | 2 | 2 | 3 | 901 | 102033.65 | 7.88 | 46.75 | 102.26 | -0.104 |
| CNL-FPE.chr1.2047 | 1 | 0 | 1 | 1 | 949 | 107081.87 | 5.99 | 37.54 | 103.62 | -0.12 |
| CNL-FPE.chr1.871 | 6 | 5 | 6 | 1 | 1269 | 145818.73 | 6.32 | 47.84 | 101.05 | -0.252 |
| CNL-FPE.chr2.3627 | 8 | 7 | 8 | 1 | 1477 | 169601.04 | 7.1 | 50.18 | 100.82 | -0.271 |
| CNL-FPE.chr2.397 | 1 | 0 | 1 | 1 | 618 | 70375.47 | 6.98 | 51.61 | 102.85 | -0.268 |
| IDSequence | Num. of Exon | Num. of Intron | Num. of CDS | Num. of UTR | Num. of Amino Acid | Molecular Weight | Theoretical pI | Instability Index | Aliphatic Index | Grand Average of Hydropathicity |
| CNL-FPE.chr2.767 | 6 | 5 | 6 | 0 | 1015 | 116289.68 | 6.11 | 44.9 | 93.76 | -0.243 |
| CNL-FPE.chr3.1482 | 3 | 2 | 2 | 3 | 998 | 112977.99 | 6.4 | 41.39 | 101.39 | -0.124 |
| CNL-FPE.chr3.2618 | 2 | 1 | 2 | 0 | 939 | 108268.82 | 8.19 | 48.8 | 97.73 | -0.249 |
| CNL-FPE.chr3.4782 | 2 | 1 | 1 | 2 | 633 | 72306.49 | 6.25 | 44.1 | 101.93 | -0.148 |
| CNL-FPE.chr3.535 | 6 | 5 | 6 | 0 | 1783 | 201646.58 | 5.53 | 40.36 | 98.9 | -0.163 |
| CNL-FPE.chr4.1619 | 2 | 1 | 1 | 3 | 955 | 109428.2 | 8.98 | 39.39 | 100.92 | -0.256 |
| CNL-FPE.chr5.124 | 1 | 0 | 1 | 0 | 933 | 106140.89 | 8.97 | 37.67 | 100.71 | -0.259 |
| CNL-FPE.chr5.3277 | 2 | 1 | 2 | 0 | 890 | 101773.94 | 8.72 | 51.22 | 103.02 | -0.215 |
| CNL-FPE.chr6.1273 | 1 | 0 | 1 | 1 | 788 | 89175.14 | 7.01 | 43.42 | 100.77 | -0.126 |
| CNL-FPE.chr6.1639 | 3 | 2 | 3 | 0 | 659 | 75606.6 | 8.2 | 48.37 | 99.36 | -0.235 |
| CNL-FPE.chr6.2574 | 2 | 1 | 2 | 0 | 891 | 101963.19 | 8.6 | 49.28 | 103.02 | -0.209 |
| CNL-FPE.chr7.1015 | 2 | 1 | 2 | 1 | 627 | 72191.66 | 5.68 | 39.07 | 100.88 | -0.046 |
| CNL-FPE.chr7.1124 | 2 | 1 | 2 | 0 | 933 | 107492.49 | 8.04 | 40.36 | 99.19 | -0.22 |
| CNL-FPE.chr7.1571 | 2 | 1 | 2 | 2 | 1391 | 158420.34 | 6.42 | 48.48 | 99.55 | -0.233 |
| CNL-FPE.chr7.1798 | 2 | 1 | 2 | 0 | 885 | 100301.83 | 5.56 | 43.7 | 101.44 | -0.133 |
| CNL-FPE.chr7.237 | 1 | 0 | 1 | 1 | 751 | 85080.96 | 6.73 | 44.89 | 96.95 | -0.19 |
| CNL-FPE.chr7.333 | 3 | 2 | 2 | 2 | 921 | 105257.89 | 8.78 | 49.8 | 98.18 | -0.253 |
| CNL-FPE.chr7.335 | 5 | 4 | 5 | 0 | 985 | 112761.05 | 6.89 | 49.32 | 99.52 | -0.274 |
| CNL-FvH4.1g23030.1 | 3 | 2 | 3 | 0 | 796 | 91227.16 | 5.85 | 40.12 | 97.83 | -0.262 |
| CNL-FvH4.2g00960.1 | 2 | 1 | 1 | 3 | 900 | 103213.89 | 6.12 | 46.89 | 102.32 | -0.094 |
| CNL-FvH4.2g13150.1 | 2 | 1 | 2 | 1 | 924 | 105111.13 | 8.78 | 48.41 | 100.41 | -0.131 |
| CNL-FvH4.2g14930.1 | 9 | 8 | 9 | 1 | 1069 | 124693.94 | 9.16 | 48.11 | 98.51 | -0.277 |
| CNL-FvH4.2g17630.1 | 7 | 6 | 7 | 1 | 399 | 46112.31 | 5.52 | 52.16 | 89.45 | -0.618 |
| CNL-FvH4.2g17640.1 | 1 | 0 | 1 | 0 | 883 | 101171.16 | 6.78 | 43.12 | 100.31 | -0.242 |
| CNL-FvH4.2g36800.1 | 3 | 2 | 2 | 3 | 1330 | 149787.33 | 5.99 | 47.95 | 96.81 | -0.209 |
| CNL-FvH4.2g36810.1 | 3 | 2 | 2 | 3 | 1288 | 145640.19 | 5.41 | 49.47 | 94.09 | -0.274 |
| CNL-FvH4.2g36830 | 2 | 1 | 2 | 2 | 1282 | 145417.52 | 5.56 | 46.82 | 93.6 | -0.324 |
| CNL-FvH4.2g36860 | 2 | 1 | 2 | 0 | 1033 | 116735.57 | 5.65 | 44.58 | 92.21 | -0.296 |
| CNL-FvH4.3g00820.1 | 6 | 5 | 2 | 6 | 867 | 98391.31 | 7.43 | 43.6 | 96.68 | -0.205 |
| CNL-FvH4.3g02290.1 | 2 | 1 | 2 | 1 | 970 | 111885.05 | 6.4 | 48.2 | 101.68 | -0.214 |
| CNL-FvH4.3g04780.1 | 11 | 10 | 8 | 5 | 1759 | 198949.72 | 5.76 | 39.85 | 99.01 | -0.184 |
| CNL-FvH4.3g05070.1 | 9 | 8 | 6 | 5 | 1639 | 185203.55 | 5.7 | 40.62 | 98.08 | -0.185 |
| CNL-FvH4.3g11470.1 | 7 | 6 | 7 | 1 | 1553 | 178282.16 | 8.44 | 53.44 | 107.38 | -0.168 |
| CNL-FvH4.3g11490.1 | 16 | 15 | 16 | 0 | 4656 | 531937.01 | 8.29 | 50.17 | 103.53 | -0.175 |
| CNL-FvH4.3g35820.1 | 15 | 14 | 2 | 15 | 1121 | 128165.84 | 6.97 | 40.45 | 99.47 | -0.155 |
| CNL-FvH4.4g06020 | 7 | 6 | 6 | 3 | 1641 | 183994.16 | 5.4 | 43.86 | 102.05 | -0.17 |
| CNL-FvH4.4g06030 | 6 | 5 | 6 | 0 | 1794 | 201428.04 | 5.3 | 46.31 | 102.73 | -0.184 |
| CNL-FvH4.4g16700.1 | 1 | 0 | 1 | 0 | 1000 | 113540.2 | 6.27 | 43.53 | 102.24 | -0.179 |
| CNL-FvH4.4g22940.1 | 8 | 7 | 8 | 0 | 1861 | 214708.18 | 6.79 | 41.76 | 100.52 | -0.214 |
| CNL-FvH4.4g25750.1 | 3 | 2 | 3 | 2 | 1112 | 125256.73 | 8.3 | 41.66 | 99.5 | -0.114 |
| CNL-FvH4.4g26300.1 | 1 | 0 | 1 | 0 | 759 | 87298.67 | 5.54 | 49.8 | 97.4 | -0.272 |
| CNL-FvH4.4g29310.1 | 6 | 5 | 1 | 7 | 721 | 82567.19 | 6.97 | 45.36 | 98.95 | -0.245 |
| IDSequence | Num. of Exon | Num. of Intron | Num. of CDS | Num. of UTR | Num. of Amino Acid | Molecular Weight | Theoretical pI | Instability Index | Aliphatic Index | Grand Average of Hydropathicity |
| CNL-FvH4.4g29930.1 | 3 | 2 | 2 | 3 | 1111 | 126325.18 | 6.18 | 44.93 | 96.75 | -0.274 |
| CNL-FvH4.4g35200.1 | 6 | 5 | 2 | 6 | 1486 | 167552.28 | 5.42 | 42.24 | 102.82 | -0.172 |
| CNL-FvH4.5g02660.1 | 10 | 9 | 7 | 5 | 1743 | 195486.05 | 5.27 | 48.09 | 99.31 | -0.272 |
| CNL-FvH4.5g05380.1 | 12 | 11 | 7 | 7 | 1751 | 197792.68 | 5.57 | 45.84 | 101.25 | -0.165 |
| CNL-FvH4.5g16110.1 | 2 | 1 | 1 | 3 | 948 | 107470.76 | 7.21 | 41.04 | 102.78 | -0.198 |
| CNL-FvH4.5g16900.1 | 2 | 1 | 1 | 2 | 868 | 98695.18 | 6.28 | 46.05 | 102.47 | -0.193 |
| CNL-FvH4.5g18950.1 | 6 | 5 | 4 | 4 | 1407 | 159707.12 | 5.81 | 45.04 | 104.39 | -0.119 |
| CNL-FvH4.5g19000.1 | 7 | 6 | 6 | 2 | 1378 | 156056.99 | 8.08 | 41.68 | 102.26 | -0.156 |
| CNL-FvH4.5g22710.1 | 1 | 0 | 1 | 0 | 941 | 108122.03 | 8.59 | 41.2 | 103.15 | -0.205 |
| CNL-FvH4.5g23390 | 1 | 0 | 1 | 0 | 939 | 107485.6 | 8.79 | 37.39 | 108.95 | -0.095 |
| CNL-FvH4.5g23400 | 2 | 1 | 1 | 3 | 955 | 109360.2 | 8.98 | 38.84 | 102.45 | -0.241 |
| CNL-FvH4.5g23420 | 1 | 0 | 1 | 0 | 952 | 109199.87 | 8.48 | 37.16 | 101.73 | -0.227 |
| CNL-FvH4.5g23430.1 | 3 | 2 | 2 | 3 | 954 | 109254.81 | 8.9 | 45.55 | 103.97 | -0.209 |
| CNL-FvH4.5g23450 | 1 | 0 | 1 | 0 | 951 | 108501.33 | 8.84 | 41.01 | 108.62 | -0.168 |
| CNL-FvH4.5g23470.1 | 1 | 0 | 1 | 0 | 948 | 107926.92 | 8.62 | 44.31 | 108.77 | -0.125 |
| CNL-FvH4.5g32430.1 | 2 | 1 | 2 | 1 | 941 | 107058.91 | 5.66 | 44.78 | 95.11 | -0.176 |
| CNL-FvH4.5g33290.1 | 5 | 4 | 3 | 3 | 1062 | 120767.75 | 5.71 | 46.16 | 97.98 | -0.245 |
| CNL-FvH4.5g34190.1 | 32 | 31 | 5 | 29 | 827 | 94107.11 | 7.53 | 47.74 | 98.92 | -0.299 |
| CNL-FvH4.5g34680.1 | 3 | 2 | 1 | 4 | 956 | 108219.39 | 5.95 | 38.03 | 101.85 | -0.108 |
| CNL-FvH4.6g02730 | 2 | 1 | 2 | 0 | 1211 | 136061.45 | 5.17 | 43.46 | 103.9 | -0.176 |
| CNL-FvH4.6g02760 | 1 | 0 | 1 | 0 | 1228 | 138916.85 | 5.65 | 44.3 | 101.43 | -0.218 |
| CNL-FvH4.6g02820 | 1 | 0 | 1 | 0 | 1224 | 138008.76 | 5.24 | 43.94 | 102.86 | -0.176 |
| CNL-FvH4.6g12200.1 | 5 | 4 | 2 | 5 | 1314 | 148132.84 | 6.36 | 48.83 | 104.99 | -0.106 |
| CNL-FvH4.6g13450.1 | 11 | 10 | 3 | 10 | 1285 | 145346.64 | 6.05 | 52.04 | 104.3 | -0.142 |
| CNL-FvH4.6g15090.1 | 7 | 6 | 3 | 6 | 1293 | 145710.54 | 6.18 | 42.1 | 101.93 | -0.158 |
| CNL-FvH4.6g15250.1 | 3 | 2 | 3 | 2 | 1367 | 154244.39 | 6.73 | 51.42 | 101.21 | -0.118 |
| CNL-FvH4.6g15340.1 | 4 | 3 | 3 | 3 | 1260 | 144048.11 | 6.23 | 50.21 | 101.16 | -0.195 |
| CNL-FvH4.6g29480.1 | 1 | 0 | 1 | 0 | 962 | 110860.58 | 8.66 | 49.58 | 101.05 | -0.238 |
| CNL-FvH4.6g30120.1 | 9 | 8 | 3 | 8 | 1155 | 131071.36 | 6.57 | 41.3 | 99.11 | -0.238 |
| CNL-FvH4.6g32110.1 | 4 | 3 | 4 | 1 | 916 | 105548.44 | 6.71 | 40.06 | 97.78 | -0.27 |
| CNL-FvH4.6g34770.1 | 2 | 1 | 1 | 3 | 1301 | 148936.58 | 8.23 | 44.45 | 98.22 | -0.256 |
| CNL-FvH4.6g47050.1 | 5 | 4 | 5 | 1 | 1481 | 167358.14 | 6.51 | 47.02 | 102.08 | -0.122 |
| CNL-FvH4.6g47980.1 | 5 | 4 | 3 | 4 | 1204 | 135025.07 | 5.39 | 51.27 | 101.06 | -0.143 |
| CNL-FvH4.6g48170.1 | 4 | 3 | 3 | 2 | 1001 | 113131.74 | 5.54 | 46.56 | 103.93 | -0.139 |
| CNL-FvH4.6g48220.1 | 2 | 1 | 2 | 0 | 905 | 102112.61 | 6.16 | 43.19 | 95.75 | -0.19 |
| CNL-FvH4.6g48310.1 | 5 | 4 | 4 | 3 | 1196 | 135460.62 | 6.11 | 42.55 | 99.92 | -0.179 |
| CNL-FvH4.6g49940.1 | 4 | 3 | 3 | 3 | 985 | 112776.11 | 6.84 | 55.02 | 98.44 | -0.294 |
| CNL-FvH4.6g51140.1 | 1 | 0 | 1 | 0 | 961 | 110584.57 | 5.97 | 47.95 | 99.03 | -0.303 |
| CNL-FvH4.6g51550.1 | 3 | 2 | 2 | 3 | 898 | 102814.02 | 6.24 | 42.66 | 99.61 | -0.227 |
| CNL-FvH4.6g51610.1 | 2 | 1 | 1 | 2 | 884 | 100480.09 | 6.12 | 45.23 | 100.54 | -0.2 |
| CNL-FvH4.6g52150.1 | 1 | 0 | 1 | 0 | 956 | 109092.08 | 8.27 | 38.12 | 101.18 | -0.264 |
| CNL-FvH4.6g53580.1 | 12 | 11 | 10 | 4 | 1912 | 216185.67 | 5.87 | 39.92 | 98.64 | -0.168 |
| IDSequence | Num. of Exon | Num. of Intron | Num. of CDS | Num. of UTR | Num. of Amino Acid | Molecular Weight | Theoretical pI | Instability Index | Aliphatic Index | Grand Average of Hydropathicity |
| CNL-FvH4.7g01950.1 | 2 | 1 | 2 | 0 | 1686 | 190396.96 | 5.91 | 47.22 | 98.52 | -0.212 |
| CNL-FvH4.7g02400.1 | 2 | 1 | 1 | 3 | 729 | 82160.1 | 6.83 | 42.59 | 98.37 | -0.089 |
| CNL-FvH4.7g02450.1 | 4 | 3 | 1 | 5 | 1335 | 150705.37 | 6.59 | 42.9 | 101.02 | -0.121 |
| CNL-FvH4.7g02890.1 | 1 | 0 | 1 | 0 | 1436 | 163006.03 | 6.03 | 56.63 | 102.68 | -0.111 |
| CNL-FvH4.7g08100.1 | 5 | 4 | 2 | 5 | 960 | 108856.84 | 6.45 | 48.85 | 102.96 | -0.106 |
| CNL-FvH4.7g08870.1 | 6 | 5 | 5 | 3 | 999 | 114218.87 | 5.89 | 50.27 | 100.68 | -0.177 |
| CNL-FvH4.7g11380.1 | 2 | 1 | 1 | 3 | 1124 | 128751.46 | 5.87 | 48.27 | 98.34 | -0.207 |
| CNL-FvH4.7g15190.1 | 10 | 9 | 7 | 4 | 731 | 83843.31 | 8.97 | 46.63 | 99.97 | -0.273 |
| CNL-FvH4.7g15240.1 | 3 | 2 | 2 | 3 | 946 | 108723.56 | 8.46 | 46.41 | 98.37 | -0.222 |
| CNL-FvH4.7g20580.1 | 3 | 2 | 3 | 0 | 602 | 69075.68 | 7.63 | 39.6 | 92.89 | -0.387 |
| CNL-FvH4.7g22030.1 | 5 | 4 | 5 | 0 | 1118 | 128207.35 | 6.09 | 44.66 | 103.39 | -0.192 |
| CNL-FvH4.7g22140.1 | 2 | 1 | 2 | 0 | 824 | 94647.83 | 6.24 | 44.93 | 107.26 | -0.225 |
| CNL-FvH4.7g23500.1 | 2 | 1 | 2 | 0 | 985 | 112828.26 | 5.71 | 44.11 | 103.05 | -0.223 |
| CNL-FvH4.7g26370.1 | 1 | 0 | 1 | 0 | 943 | 108545.22 | 5.56 | 44.54 | 103.41 | -0.233 |
| CNL-FvH4.7g27330.1 | 3 | 2 | 3 | 0 | 1443 | 164409.77 | 6.14 | 52.34 | 97.58 | -0.257 |
| CNL-FvH4.7g27890.1 | 2 | 1 | 2 | 1 | 893 | 101549.19 | 7.95 | 42.42 | 94.62 | -0.306 |
| CNL-FvH4.7g28110.1 | 3 | 2 | 1 | 4 | 1392 | 158022.55 | 6.36 | 42.99 | 98.35 | -0.229 |
| CNL-FvH4.7g28390.1 | 11 | 10 | 2 | 10 | 1406 | 159224.95 | 6.05 | 46.85 | 98.43 | -0.229 |
| CNL-FvH4.7g31140.1 | 16 | 15 | 10 | 8 | 2192 | 247200.15 | 5.94 | 40.92 | 100.24 | -0.182 |
| CNL-FvH4.7g31330.1 | 11 | 10 | 11 | 1 | 1149 | 131757.59 | 8.82 | 48.91 | 104.73 | -0.045 |
| CNL-FvH4.7g33440.1 | 4 | 3 | 4 | 1 | 918 | 106207.33 | 8.81 | 45.92 | 94.79 | -0.332 |
| CNL-FVI.CHR1.1117 | 2 | 1 | 1 | 3 | 841 | 95352.16 | 6.47 | 42.9 | 99.63 | -0.148 |
| CNL-FVI.CHR1.1853 | 1 | 0 | 1 | 0 | 867 | 98659.9 | 6.44 | 44.57 | 99.15 | -0.24 |
| CNL-FVI.CHR1.1874 | 2 | 1 | 2 | 2 | 773 | 87648.1 | 5.86 | 42.22 | 100.12 | -0.22 |
| CNL-FVI.CHR1.572 | 2 | 1 | 2 | 1 | 813 | 92586.76 | 7.37 | 39.98 | 96.04 | -0.308 |
| CNL-FVI.CHR2.1431 | 5 | 4 | 5 | 0 | 919 | 104285.28 | 5.27 | 55.06 | 93.75 | -0.357 |
| CNL-FVI.CHR2.1471 | 4 | 3 | 3 | 3 | 951 | 108830.91 | 8.98 | 44.31 | 100.52 | -0.208 |
| CNL-FVI.CHR2.2054 | 2 | 1 | 2 | 1 | 969 | 112675.33 | 9.06 | 49.87 | 102.16 | -0.184 |
| CNL-FVI.CHR2.2280 | 1 | 0 | 1 | 1 | 883 | 101141.88 | 6.85 | 44.44 | 97.76 | -0.282 |
| CNL-FVI.CHR2.3860 | 1 | 0 | 1 | 0 | 895 | 101997.14 | 5.48 | 43.46 | 102.27 | -0.213 |
| CNL-FVI.CHR2.555 | 3 | 2 | 1 | 4 | 893 | 100981.11 | 5.67 | 47.11 | 102.82 | -0.1 |
| CNL-FVI.CHR3.185 | 2 | 1 | 2 | 0 | 781 | 88572.43 | 7.33 | 40.59 | 101.49 | -0.204 |
| CNL-FVI.CHR3.618 | 3 | 2 | 3 | 2 | 991 | 112469.67 | 7.82 | 50.36 | 102.82 | -0.123 |
| CNL-FVI.CHR5.1412 | 1 | 0 | 1 | 1 | 868 | 98893.27 | 6.21 | 47.1 | 101.34 | -0.212 |
| CNL-FVI.CHR5.1617 | 8 | 7 | 4 | 6 | 1389 | 157433.38 | 6.17 | 43.54 | 103.84 | -0.151 |
| CNL-FVI.CHR5.2975 | 3 | 2 | 1 | 4 | 956 | 108318.25 | 5.9 | 38.53 | 101.04 | -0.145 |
| CNL-FVI.CHR5.3016 | 8 | 7 | 7 | 3 | 1695 | 191554.52 | 5.95 | 45.39 | 103.09 | -0.146 |
| CNL-FVI.CHR6.1106 | 3 | 2 | 3 | 0 | 1062 | 119140.82 | 6.25 | 47.11 | 100.11 | -0.161 |
| CNL-FVI.CHR6.1768 | 11 | 10 | 11 | 2 | 1407 | 160149.53 | 6.77 | 48.1 | 100.63 | -0.144 |
| CNL-FVI.CHR6.3417 | 3 | 2 | 3 | 2 | 1176 | 133517.27 | 5.91 | 46.55 | 97.38 | -0.251 |
| CNL-FVI.CHR6.3578 | 4 | 3 | 3 | 3 | 995 | 113951.34 | 6.68 | 52.02 | 95.98 | -0.341 |
| CNL-FVI.CHR7.1021 | 1 | 0 | 1 | 0 | 609 | 70167.78 | 5.96 | 45.14 | 100 | -0.233 |
| IDSequence | Num. of Exon | Num. of Intron | Num. of CDS | Num. of UTR | Num. of Amino Acid | Molecular Weight | Theoretical pI | Instability Index | Aliphatic Index | Grand Average of Hydropathicity |
| CNL-FVI.CHR7.1051 | 6 | 5 | 3 | 5 | 1306 | 147517.69 | 6.92 | 44.96 | 105.61 | -0.046 |
| CNL-FVI.CHR7.1336 | 2 | 1 | 2 | 0 | 937 | 108661.3 | 7.94 | 41.31 | 97.74 | -0.274 |
| CNL-FVI.CHR7.171 | 1 | 0 | 1 | 0 | 1324 | 150171.35 | 6.06 | 47.26 | 101.45 | -0.154 |
| CNL-FVI.CHR7.2604 | 8 | 7 | 7 | 2 | 1036 | 119230 | 8.21 | 41.53 | 106.62 | -0.1 |
| CNL-FVI.CHR7.264 | 1 | 0 | 1 | 2 | 1445 | 163796.77 | 5.81 | 54.5 | 101.78 | -0.1 |
| CNL-FVI.CHR7.2923 | 4 | 3 | 2 | 4 | 866 | 98014.6 | 6.49 | 42.59 | 96.47 | -0.22 |
| CNL-FVI.CHR7.2926 | 4 | 3 | 2 | 4 | 871 | 98784.65 | 6.15 | 45 | 97.8 | -0.189 |
| CNL-FVI.CHR7.3757 | 3 | 2 | 1 | 4 | 922 | 105065.04 | 8.94 | 49.95 | 105.09 | -0.168 |
| CNL-FVI.CHR7.3758 | 3 | 2 | 2 | 2 | 633 | 72799.25 | 7.23 | 48.48 | 102.56 | -0.265 |
| CNL-FVI.CHR7.3762 | 2 | 1 | 2 | 0 | 783 | 90637.72 | 6.87 | 56.2 | 104.42 | -0.142 |
| CNL-FVI.CHR7.810 | 7 | 6 | 7 | 2 | 868 | 98612.7 | 5.68 | 50.38 | 98.93 | -0.197 |
| N-FDA.chr0.1053 | 1 | 0 | 1 | 0 | 83 | 9065.34 | 5.41 | 28.58 | 106.87 | 0.069 |
| N-FDA.chr1.18128 | 4 | 3 | 4 | 0 | 341 | 38341.68 | 8.25 | 36.65 | 98.8 | -0.084 |
| N-FDA.chr1.22180 | 3 | 2 | 3 | 0 | 271 | 30701.9 | 4.84 | 41.32 | 91 | -0.244 |
| N-FDA.chr2.08227 | 1 | 0 | 1 | 0 | 342 | 40354.9 | 9.92 | 46.22 | 92.25 | -0.438 |
| N-FDA.chr2.11387 | 2 | 1 | 2 | 0 | 82 | 9129.43 | 4.47 | 32.63 | 90.37 | -0.124 |
| N-FDA.chr2.11533 | 4 | 3 | 4 | 0 | 237 | 26684.96 | 9.3 | 45.02 | 86.75 | -0.124 |
| N-FDA.chr2.12211 | 1 | 0 | 1 | 0 | 87 | 9393.73 | 5.05 | 37.97 | 89.77 | -0.214 |
| N-FDA.chr3.05877 | 1 | 0 | 1 | 0 | 106 | 12096.84 | 5.61 | 45.71 | 78.21 | -0.558 |
| N-FDA.chr3.05878 | 1 | 0 | 1 | 0 | 106 | 12096.84 | 5.61 | 45.71 | 78.21 | -0.558 |
| N-FDA.chr3.06067 | 1 | 0 | 1 | 0 | 972 | 109069.17 | 8.76 | 43.35 | 84.44 | -0.277 |
| N-FDA.chr3.20666 | 2 | 1 | 2 | 0 | 406 | 45564.49 | 6.29 | 44.38 | 92.51 | -0.254 |
| N-FDA.chr3.20671 | 2 | 1 | 2 | 0 | 367 | 41521.21 | 8.25 | 39.82 | 100.68 | -0.204 |
| N-FDA.chr3.20675 | 2 | 1 | 2 | 0 | 119 | 13028.87 | 5.07 | 22.6 | 87.56 | -0.142 |
| N-FDA.chr3.20742 | 1 | 0 | 1 | 0 | 259 | 29667.24 | 6.26 | 36.54 | 88.88 | -0.373 |
| N-FDA.chr3.20808 | 3 | 2 | 3 | 0 | 546 | 62333.88 | 6.56 | 48.5 | 102.23 | -0.202 |
| N-FDA.chr3.20832 | 1 | 0 | 1 | 0 | 251 | 28858.57 | 8.54 | 46 | 91.31 | -0.323 |
| N-FDA.chr3.20833 | 3 | 2 | 3 | 0 | 394 | 45277.57 | 8.23 | 38.83 | 95.51 | -0.274 |
| N-FDA.chr3.20836 | 2 | 1 | 2 | 0 | 314 | 35687.35 | 6.12 | 43.02 | 90.35 | -0.246 |
| N-FDA.chr3.24469 | 4 | 3 | 4 | 0 | 477 | 55124.66 | 8.62 | 50.33 | 107.65 | -0.2 |
| N-FDA.chr5.07880 | 2 | 1 | 2 | 0 | 190 | 21315.43 | 5.89 | 30.05 | 95.42 | -0.215 |
| N-FDA.chr6.17361 | 2 | 1 | 2 | 0 | 168 | 18504.28 | 8.35 | 43.02 | 84.64 | -0.182 |
| N-FDA.chr7.14603 | 2 | 1 | 2 | 0 | 396 | 44708.43 | 5.77 | 41.36 | 92.07 | -0.252 |
| N-FDA.chr7.22742 | 1 | 0 | 1 | 0 | 194 | 21684.23 | 5.48 | 43.27 | 92.47 | -0.015 |
| N-FII.chr1.1277 | 4 | 3 | 4 | 0 | 336 | 38186.53 | 5.21 | 53.3 | 98.36 | -0.192 |
| N-FII.chr1.1913 | 1 | 0 | 1 | 0 | 262 | 29997.69 | 6.64 | 47.96 | 106.76 | -0.193 |
| N-FII.chr2.1082 | 1 | 0 | 1 | 0 | 197 | 22025.22 | 7.05 | 43.09 | 94.82 | -0.035 |
| N-FII.chr2.2318 | 1 | 0 | 1 | 0 | 94 | 10975.47 | 5.88 | 25.08 | 87.02 | -0.593 |
| N-FII.chr2.645 | 1 | 0 | 1 | 0 | 193 | 21338.66 | 5.65 | 30.8 | 112.54 | 0.064 |
| N-FII.chr2.79 | 2 | 1 | 2 | 0 | 180 | 20544.79 | 5.2 | 43.61 | 92.5 | -0.045 |
| N-FII.chr4.1428 | 2 | 1 | 2 | 0 | 78 | 8594.14 | 9.73 | 38.29 | 101.15 | -0.256 |
| N-FII.chr5.1933 | 2 | 1 | 2 | 0 | 393 | 44367.29 | 6.53 | 40.06 | 94.02 | -0.24 |
| IDSequence | Num. of Exon | Num. of Intron | Num. of CDS | Num. of UTR | Num. of Amino Acid | Molecular Weight | Theoretical pI | Instability Index | Aliphatic Index | Grand Average of Hydropathicity |
| N-FII.chr5.2769 | 1 | 0 | 1 | 0 | 264 | 30022.81 | 8.09 | 35.68 | 99.62 | -0.181 |
| N-FII.chr6.1009 | 2 | 1 | 2 | 0 | 245 | 27472.69 | 8.59 | 27.91 | 95.47 | -0.387 |
| N-FII.chr6.1246 | 2 | 1 | 2 | 0 | 304 | 34394.7 | 6.47 | 41.69 | 98.72 | -0.237 |
| N-FII.chr6.2591 | 4 | 3 | 4 | 0 | 435 | 49951.62 | 6.44 | 52.97 | 94.71 | -0.252 |
| N-FII.chr6.2592 | 1 | 0 | 1 | 0 | 103 | 11579.29 | 6.27 | 32.6 | 92.62 | -0.207 |
| N-FII.chr7.1290 | 4 | 3 | 4 | 0 | 541 | 62152.3 | 6.05 | 39 | 90.44 | -0.363 |
| N-FII.chr7.209 | 1 | 0 | 1 | 0 | 285 | 31862.82 | 7.07 | 26.06 | 100.84 | -0.015 |
| N-FMA.chr1.2322 | 1 | 0 | 1 | 2 | 112 | 12528.5 | 9.57 | 32.74 | 112.14 | -0.101 |
| N-FMA.chr2.1406 | 2 | 1 | 2 | 0 | 164 | 18610.42 | 8.81 | 36.5 | 101.59 | -0.199 |
| N-FMA.chr2.311 | 1 | 0 | 1 | 0 | 83 | 9187.61 | 8.66 | 45.76 | 92.89 | -0.299 |
| N-FMA.chr2.420 | 1 | 0 | 1 | 0 | 210 | 23695.38 | 7.17 | 28 | 116 | -0.031 |
| N-FMA.chr2.489 | 1 | 0 | 1 | 0 | 392 | 44076.79 | 6.08 | 37.75 | 106.12 | -0.121 |
| N-FMA.chr3.2642 | 2 | 1 | 2 | 0 | 151 | 17116.41 | 5.01 | 42.66 | 83.91 | -0.287 |
| N-FMA.chr3.3384 | 1 | 0 | 1 | 2 | 969 | 108779.94 | 8.75 | 43.99 | 84.61 | -0.276 |
| N-FMA.chr4.1879 | 3 | 2 | 3 | 0 | 372 | 42073.04 | 8.96 | 34.86 | 101.13 | -0.193 |
| N-FMA.chr4.2247 | 2 | 1 | 2 | 0 | 196 | 22579.92 | 6.74 | 39.76 | 93.47 | -0.417 |
| N-FMA.chr5.2639 | 1 | 0 | 1 | 0 | 475 | 53510.93 | 7.56 | 38.42 | 96.86 | -0.137 |
| N-FMA.chr6.2154 | 2 | 1 | 2 | 0 | 473 | 53471.56 | 5.97 | 38.39 | 99.53 | -0.203 |
| N-FMA.chr6.2173 | 1 | 0 | 1 | 2 | 153 | 17348.35 | 8.81 | 33.59 | 91.11 | -0.011 |
| N-FNG.chr1.1565 | 1 | 0 | 1 | 0 | 181 | 20305.55 | 9.11 | 41.94 | 93.09 | -0.293 |
| N-FNG.chr1.426 | 2 | 1 | 2 | 0 | 258 | 28696.28 | 6.75 | 42.8 | 107.25 | -0.019 |
| N-FNG.chr2.1151 | 1 | 0 | 1 | 0 | 112 | 12679.53 | 7.02 | 23.87 | 88.66 | -0.271 |
| N-FNG.chr2.1425 | 1 | 0 | 1 | 0 | 105 | 11990.78 | 9.2 | 49.84 | 102.95 | -0.34 |
| N-FNG.chr2.1856 | 2 | 1 | 2 | 0 | 119 | 13180.03 | 4.97 | 49.05 | 76.22 | -0.417 |
| N-FNG.chr3.2988 | 2 | 1 | 2 | 0 | 130 | 14418.44 | 7.76 | 29.75 | 93.69 | -0.178 |
| N-FNG.chr3.3429 | 1 | 0 | 1 | 1 | 970 | 108794.81 | 8.76 | 43.53 | 83.82 | -0.287 |
| N-FNG.chr4.272 | 1 | 0 | 1 | 0 | 107 | 11882.71 | 8.72 | 51.43 | 100.28 | -0.065 |
| N-FNG.chr4.896 | 3 | 2 | 3 | 0 | 151 | 17226.07 | 8.36 | 41.58 | 96.89 | -0.207 |
| N-FNG.chr6.1058 | 1 | 0 | 1 | 0 | 141 | 16041.33 | 5.73 | 36.16 | 94.68 | -0.384 |
| N-FNG.chr6.1059 | 2 | 1 | 2 | 0 | 205 | 23628.27 | 5.96 | 66.26 | 89.8 | -0.33 |
| N-FNG.chr6.568 | 3 | 2 | 3 | 0 | 200 | 22274.7 | 9.09 | 37.01 | 98.9 | -0.161 |
| N-FNU.ctg119.817 | 10 | 9 | 10 | 0 | 600 | 67701.64 | 9.37 | 51.17 | 83.05 | -0.421 |
| N-FNU.ctg119.818 | 2 | 1 | 2 | 1 | 131 | 13895.71 | 4.96 | 40.29 | 76.72 | -0.338 |
| N-FNU.ctg119.819 | 1 | 0 | 1 | 0 | 101 | 11243.13 | 9.75 | 37.49 | 77.33 | -0.221 |
| N-FNU.ctg124.289 | 1 | 0 | 1 | 0 | 314 | 35825.26 | 6.17 | 40.93 | 89.75 | -0.282 |
| N-FNU.ctg155.194 | 1 | 0 | 1 | 0 | 218 | 24705.48 | 5.47 | 32.14 | 96.97 | -0.088 |
| N-FNU.ctg161.240 | 3 | 2 | 2 | 2 | 563 | 63379.88 | 5.97 | 44.68 | 96.59 | -0.16 |
| N-FNU.ctg172.22 | 5 | 4 | 5 | 0 | 437 | 48880.31 | 6.1 | 40.1 | 97.69 | -0.09 |
| N-FNU.ctg172.28 | 6 | 5 | 6 | 0 | 492 | 56178.32 | 5.65 | 45.19 | 90.33 | -0.338 |
| N-FNU.ctg48.4 | 3 | 2 | 3 | 0 | 312 | 36458.51 | 9.9 | 50.48 | 99.68 | -0.422 |
| N-FNU.ctg56.229 | 1 | 0 | 1 | 0 | 144 | 16654.3 | 9.1 | 44.74 | 83.89 | -0.161 |
| N-FNU.ctg57.541 | 1 | 0 | 1 | 0 | 228 | 26227.8 | 8.53 | 67.08 | 107.76 | -0.2 |
| IDSequence | Num. of Exon | Num. of Intron | Num. of CDS | Num. of UTR | Num. of Amino Acid | Molecular Weight | Theoretical pI | Instability Index | Aliphatic Index | Grand Average of Hydropathicity |
| N-FNU.ctg65.16 | 4 | 3 | 4 | 0 | 113 | 12849.99 | 8.51 | 32.15 | 98.41 | -0.225 |
| N-FPE.chr0.2268 | 2 | 1 | 2 | 0 | 357 | 39928.41 | 5.53 | 32.45 | 84.93 | -0.513 |
| N-FPE.chr2.64 | 1 | 0 | 1 | 0 | 402 | 46155.08 | 8.29 | 47.61 | 90.87 | -0.2 |
| N-FPE.chr3.1891 | 3 | 2 | 3 | 0 | 675 | 77373.72 | 8.87 | 48.23 | 103.5 | -0.183 |
| N-FPE.chr3.1892 | 2 | 1 | 2 | 0 | 392 | 44904.67 | 6.29 | 50.34 | 96.68 | -0.319 |
| N-FPE.chr3.1895 | 2 | 1 | 2 | 0 | 656 | 74824.47 | 7.59 | 47.15 | 107.67 | -0.068 |
| N-FPE.chr3.3480 | 1 | 0 | 1 | 0 | 711 | 79683.48 | 6.21 | 51.52 | 76.62 | -0.39 |
| N-FPE.chr5.126 | 2 | 1 | 2 | 0 | 309 | 35038 | 5.17 | 37.22 | 87.48 | -0.297 |
| N-FPE.chr6.5282 | 7 | 6 | 7 | 0 | 304 | 34781.28 | 6.91 | 42.62 | 92.27 | -0.368 |
| N-FPE.chr6.572 | 1 | 0 | 1 | 2 | 969 | 108662.73 | 8.76 | 42.99 | 84.11 | -0.285 |
| N-FvH4.1g12710.1 | 3 | 2 | 3 | 0 | 463 | 52544.3 | 6.54 | 42.1 | 89.87 | -0.279 |
| N-FvH4.2g08360.1 | 3 | 2 | 3 | 1 | 195 | 20951.94 | 6.17 | 46.52 | 105.9 | -0.16 |
| N-FvH4.2g17340.1 | 3 | 2 | 3 | 0 | 262 | 29939.56 | 7.77 | 40.39 | 100.34 | -0.238 |
| N-FvH4.2g17400.1 | 1 | 0 | 1 | 0 | 330 | 36505.22 | 5.04 | 33.38 | 104.91 | -0.072 |
| N-FvH4.3g12820.1 | 2 | 1 | 2 | 2 | 161 | 17819.72 | 9.14 | 41.27 | 74.97 | -0.347 |
| N-FvH4.3g41580.1 | 2 | 1 | 1 | 3 | 969 | 108779.94 | 8.75 | 43.99 | 84.61 | -0.276 |
| N-FvH4.3g44060.1 | 2 | 1 | 2 | 0 | 119 | 13109.88 | 4.92 | 32.28 | 85.97 | -0.151 |
| N-FvH4.4g23400.1 | 2 | 1 | 2 | 0 | 174 | 20275.35 | 8.3 | 37.19 | 91.84 | -0.501 |
| N-FvH4.5g11130.1 | 2 | 1 | 2 | 0 | 214 | 24424.85 | 5.76 | 44.08 | 89.72 | -0.337 |
| N-FvH4.5g21070.1 | 4 | 3 | 4 | 0 | 471 | 53546.55 | 6.04 | 36.34 | 95.77 | -0.202 |
| N-FvH4.5g21530.1 | 2 | 1 | 2 | 0 | 183 | 20748.02 | 9.68 | 48.18 | 85.74 | -0.331 |
| N-FvH4.5g24320.1 | 7 | 6 | 7 | 0 | 794 | 89990.03 | 7.35 | 42.94 | 97.34 | -0.123 |
| N-FvH4.5g38520.1 | 6 | 5 | 6 | 0 | 391 | 44568.12 | 5.63 | 39.78 | 100.36 | -0.285 |
| N-FvH4.6g30050.1 | 2 | 1 | 2 | 0 | 107 | 12111.29 | 9.01 | 42.12 | 88.41 | -0.12 |
| N-FvH4.6g30240.1 | 3 | 2 | 2 | 3 | 473 | 53471.56 | 5.97 | 38.39 | 99.53 | -0.203 |
| N-FvH4.6g50120.1 | 3 | 2 | 3 | 1 | 221 | 24862.54 | 4.78 | 49.51 | 97.51 | -0.076 |
| N-FvH4.7g09280.1 | 1 | 0 | 1 | 0 | 337 | 38231.87 | 6.06 | 52.94 | 98.93 | -0.277 |
| N-FvH4.7g15200.1 | 6 | 5 | 6 | 0 | 736 | 83315.69 | 6.08 | 42.5 | 94.01 | -0.245 |
| N-FVI.CHR1.1875 | 1 | 0 | 1 | 2 | 218 | 24697.48 | 8.75 | 37.58 | 83.58 | -0.279 |
| N-FVI.CHR1.2224 | 2 | 1 | 2 | 0 | 152 | 17162.99 | 9.39 | 35.04 | 92.96 | -0.253 |
| N-FVI.CHR2.1889 | 1 | 0 | 1 | 0 | 117 | 13041.36 | 9.39 | 46.07 | 114.1 | 0.216 |
| N-FVI.CHR2.1890 | 1 | 0 | 1 | 1 | 298 | 34276.19 | 6.01 | 43.57 | 93.79 | -0.264 |
| N-FVI.CHR3.1742 | 2 | 1 | 2 | 0 | 321 | 36893.61 | 7.66 | 28.04 | 99.88 | -0.221 |
| N-FVI.CHR3.2405 | 1 | 0 | 1 | 2 | 969 | 108651.71 | 8.73 | 43.21 | 84.61 | -0.28 |
| N-FVI.CHR4.695 | 2 | 1 | 2 | 0 | 201 | 22350.68 | 8.34 | 49.65 | 96.52 | -0.152 |
| N-FVI.CHR7.1334 | 2 | 1 | 2 | 1 | 465 | 53315.65 | 8.14 | 31.34 | 91.16 | -0.244 |
| N-FVI.CHR7.3760 | 1 | 0 | 1 | 0 | 338 | 39116.91 | 8.37 | 53.03 | 109.53 | -0.075 |
| N-FVI.CHR7.738 | 2 | 1 | 2 | 0 | 266 | 29726.95 | 5.94 | 37.91 | 94.85 | -0.17 |
| NL-FDA.chr1.18083 | 3 | 2 | 3 | 0 | 890 | 101097.75 | 6.03 | 40.23 | 97.48 | -0.19 |
| NL-FDA.chr2.12019 | 5 | 4 | 5 | 0 | 1283 | 145567.43 | 6.41 | 42.29 | 107.09 | -0.1 |
| NL-FDA.chr2.17949 | 2 | 1 | 2 | 0 | 409 | 46066.44 | 6.35 | 39.82 | 106.36 | -0.103 |
| NL-FDA.chr3.05440 | 2 | 1 | 2 | 0 | 803 | 91407.21 | 5.14 | 41.76 | 104.17 | -0.104 |
| IDSequence | Num. of Exon | Num. of Intron | Num. of CDS | Num. of UTR | Num. of Amino Acid | Molecular Weight | Theoretical pI | Instability Index | Aliphatic Index | Grand Average of Hydropathicity |
| NL-FDA.chr3.05618 | 4 | 3 | 4 | 0 | 617 | 69733.43 | 6.69 | 44.99 | 106.63 | 0.017 |
| NL-FDA.chr3.05836 | 2 | 1 | 2 | 0 | 918 | 105204.76 | 6.22 | 38.72 | 100.96 | -0.269 |
| NL-FDA.chr3.06281 | 1 | 0 | 1 | 0 | 897 | 101538.91 | 6.04 | 44.91 | 111.47 | 0.031 |
| NL-FDA.chr3.20676 | 1 | 0 | 1 | 0 | 921 | 104074.98 | 5.84 | 38 | 105.84 | -0.021 |
| NL-FDA.chr3.20746 | 5 | 4 | 5 | 0 | 831 | 94553.03 | 6.22 | 44.06 | 97.11 | -0.215 |
| NL-FDA.chr3.20838 | 3 | 2 | 3 | 0 | 956 | 108129.05 | 6.39 | 41.48 | 98.38 | -0.192 |
| NL-FDA.chr3.21153 | 1 | 0 | 1 | 0 | 687 | 78288.23 | 9.08 | 35.85 | 104.43 | -0.168 |
| NL-FDA.chr4.04655 | 4 | 3 | 4 | 0 | 1164 | 131070.81 | 6.01 | 42.98 | 105.85 | -0.113 |
| NL-FDA.chr4.05233 | 2 | 1 | 2 | 0 | 788 | 89554.28 | 6.87 | 51.61 | 97.83 | -0.238 |
| NL-FDA.chr4.08398 | 1 | 0 | 1 | 0 | 518 | 60131.37 | 7.16 | 45.69 | 96.29 | -0.146 |
| NL-FDA.chr5.03601 | 4 | 3 | 4 | 0 | 442 | 50465.8 | 8.92 | 48.4 | 102.04 | -0.229 |
| NL-FDA.chr5.12862 | 5 | 4 | 5 | 0 | 1138 | 128553.08 | 6.21 | 40.6 | 94.46 | -0.18 |
| NL-FDA.chr5.21327 | 4 | 3 | 4 | 0 | 364 | 40871.75 | 6.47 | 42.96 | 107.72 | 0.002 |
| NL-FDA.chr6.02338 | 4 | 3 | 4 | 0 | 941 | 107199.39 | 8.46 | 45.29 | 96.4 | -0.2 |
| NL-FDA.chr6.26447 | 2 | 1 | 2 | 0 | 1196 | 134849.68 | 6.84 | 51.42 | 104.11 | -0.118 |
| NL-FDA.chr6.26794 | 3 | 2 | 3 | 0 | 1317 | 148666.26 | 5.82 | 43.19 | 106.34 | -0.111 |
| NL-FDA.chr7.00111 | 3 | 2 | 3 | 0 | 533 | 61270.95 | 6.88 | 44.28 | 105.68 | -0.151 |
| NL-FDA.chr7.00705 | 3 | 2 | 3 | 0 | 613 | 68515.31 | 6.94 | 41.84 | 99.35 | -0.187 |
| NL-FDA.chr7.01329 | 4 | 3 | 4 | 0 | 795 | 90566.18 | 5.72 | 41.23 | 93.31 | -0.173 |
| NL-FDA.chr7.14306 | 3 | 2 | 3 | 0 | 842 | 95281.62 | 5.41 | 42.84 | 101.12 | -0.118 |
| NL-FDA.chr7.14797 | 2 | 1 | 2 | 0 | 472 | 54325.25 | 7.99 | 51.95 | 97.69 | -0.166 |
| NL-FDA.chr7.18714 | 2 | 1 | 2 | 0 | 403 | 45748.63 | 5.41 | 25.87 | 99.55 | -0.231 |
| NL-FII.chr1.1268 | 5 | 4 | 4 | 3 | 638 | 73464.31 | 5.45 | 52.11 | 111.35 | -0.055 |
| NL-FII.chr2.2367 | 3 | 2 | 3 | 0 | 758 | 85903.36 | 5.24 | 46.64 | 97.59 | -0.144 |
| NL-FII.chr2.452 | 1 | 0 | 1 | 0 | 314 | 35158.79 | 7 | 35.79 | 102.8 | -0.112 |
| NL-FII.chr3.589 | 9 | 8 | 5 | 6 | 848 | 95683.34 | 4.97 | 42.12 | 98.03 | -0.325 |
| NL-FII.chr5.1996 | 2 | 1 | 2 | 1 | 631 | 72191.8 | 6.12 | 49.25 | 101.92 | -0.092 |
| NL-FII.chr5.3064 | 1 | 0 | 1 | 0 | 820 | 93943.37 | 9.1 | 47.41 | 103.6 | -0.184 |
| NL-FII.chr6.1611 | 2 | 1 | 2 | 0 | 545 | 61669.01 | 8.04 | 44.59 | 102.46 | 0.024 |
| NL-FII.chr6.1892 | 2 | 1 | 1 | 2 | 1297 | 146753.06 | 5.89 | 44.27 | 104.97 | -0.146 |
| NL-FII.chr6.2634 | 3 | 2 | 3 | 1 | 1028 | 116223.41 | 5.42 | 52.03 | 94.42 | -0.293 |
| NL-FMA.chr1.1431 | 1 | 0 | 1 | 0 | 747 | 84672.93 | 7.5 | 43.22 | 98.21 | -0.168 |
| NL-FMA.chr2.856 | 5 | 4 | 5 | 0 | 1047 | 119180.32 | 8.72 | 47.79 | 98.59 | -0.174 |
| NL-FMA.chr4.1335 | 1 | 0 | 1 | 0 | 892 | 101136.96 | 6.22 | 44.85 | 103.89 | -0.145 |
| NL-FMA.chr5.2765 | 5 | 4 | 5 | 0 | 384 | 43931.77 | 5.7 | 48.09 | 103.78 | -0.287 |
| NL-FMA.chr6.1747 | 1 | 0 | 1 | 1 | 1297 | 146582.48 | 5.72 | 42.03 | 105.12 | -0.158 |
| NL-FMA.chr7.904 | 4 | 3 | 4 | 0 | 979 | 111137.87 | 5.82 | 51.9 | 103.37 | -0.048 |
| NL-FNG.chr1.1121 | 1 | 0 | 1 | 0 | 590 | 66847.58 | 6.99 | 40.11 | 103.2 | -0.099 |
| NL-FNG.chr2.1152 | 3 | 2 | 3 | 0 | 293 | 33815.93 | 6.34 | 53.37 | 93.52 | -0.305 |
| NL-FNG.chr2.1426 | 3 | 2 | 3 | 0 | 610 | 69200.3 | 5.67 | 44.35 | 107.34 | 0.045 |
| NL-FNG.chr3.1831 | 6 | 5 | 6 | 0 | 655 | 74826.93 | 7.5 | 40.61 | 99.83 | -0.1 |
| NL-FNG.chr3.2336 | 3 | 2 | 3 | 0 | 506 | 56656.91 | 5.85 | 34.9 | 100.99 | -0.091 |
| IDSequence | Num. of Exon | Num. of Intron | Num. of CDS | Num. of UTR | Num. of Amino Acid | Molecular Weight | Theoretical pI | Instability Index | Aliphatic Index | Grand Average of Hydropathicity |
| NL-FNG.chr3.2989 | 3 | 2 | 3 | 1 | 459 | 52337.24 | 5.95 | 57.23 | 92.79 | -0.205 |
| NL-FNG.chr3.522 | 5 | 4 | 5 | 0 | 900 | 102773.11 | 6.48 | 53.75 | 101.33 | -0.177 |
| NL-FNG.chr4.1397 | 1 | 0 | 1 | 0 | 322 | 36674.72 | 6.83 | 54.08 | 103.82 | -0.053 |
| NL-FNG.chr5.1946 | 4 | 3 | 4 | 0 | 682 | 77167.22 | 6.15 | 34.6 | 106.03 | -0.053 |
| NL-FNG.chr5.800 | 1 | 0 | 1 | 0 | 873 | 98304.83 | 6.58 | 50.56 | 99.47 | -0.108 |
| NL-FNG.chr6.2521 | 1 | 0 | 1 | 0 | 1293 | 146321.69 | 5.92 | 44.07 | 105.38 | -0.146 |
| NL-FNG.chr7.1223 | 7 | 6 | 7 | 0 | 860 | 99036.53 | 7.99 | 47.24 | 101.77 | -0.185 |
| NL-FNG.chr7.723 | 4 | 3 | 4 | 0 | 611 | 69345.28 | 6.49 | 52.8 | 107.69 | -0.078 |
| NL-FNU.ctg131.54 | 4 | 3 | 3 | 2 | 621 | 71505.43 | 9.04 | 47.75 | 102.61 | -0.079 |
| NL-FNU.ctg132.328 | 3 | 2 | 1 | 4 | 931 | 106995.7 | 6.32 | 51.21 | 97.99 | -0.203 |
| NL-FNU.ctg134.34 | 2 | 1 | 2 | 0 | 441 | 50823.86 | 8.36 | 55.63 | 95.94 | -0.247 |
| NL-FNU.ctg144.28 | 1 | 0 | 1 | 0 | 1200 | 135999.77 | 5.88 | 59.04 | 102.33 | -0.098 |
| NL-FNU.ctg155.6 | 9 | 8 | 9 | 0 | 1449 | 163247.51 | 6.15 | 40.02 | 102.42 | -0.206 |
| NL-FNU.ctg158.38 | 2 | 1 | 1 | 2 | 581 | 67021.12 | 9.25 | 36.86 | 114.53 | -0.071 |
| NL-FNU.ctg161.49 | 10 | 9 | 2 | 10 | 861 | 97350.38 | 5.63 | 49.15 | 95.73 | -0.186 |
| NL-FNU.ctg163.12 | 1 | 0 | 1 | 2 | 984 | 112595.87 | 7.61 | 54.23 | 99.14 | -0.132 |
| NL-FNU.ctg169.34 | 5 | 4 | 5 | 0 | 1090 | 120907.89 | 5.91 | 38.57 | 111.1 | 0.033 |
| NL-FNU.ctg72.1 | 3 | 2 | 1 | 4 | 1297 | 146894.93 | 5.57 | 42.94 | 106.17 | -0.142 |
| NL-FNU.ctg81.827 | 7 | 6 | 4 | 4 | 677 | 75961.99 | 8.13 | 47.64 | 96.44 | -0.24 |
| NL-FNU.ctg82.264 | 1 | 0 | 1 | 2 | 477 | 55645.35 | 8.54 | 44.25 | 98.05 | -0.142 |
| NL-FNU.ctg84.211 | 2 | 1 | 2 | 0 | 687 | 77327.66 | 6.14 | 48.64 | 105.95 | -0.098 |
| NL-FPE.chr0.1234 | 2 | 1 | 2 | 1 | 1008 | 115684.74 | 7.53 | 48.24 | 101.31 | -0.228 |
| NL-FPE.chr0.887 | 5 | 4 | 1 | 6 | 1297 | 146819.71 | 5.6 | 42.72 | 105.2 | -0.157 |
| NL-FPE.chr2.1814 | 7 | 6 | 7 | 2 | 1161 | 132043.48 | 6.34 | 43.52 | 91.37 | -0.263 |
| NL-FPE.chr2.4140 | 5 | 4 | 5 | 0 | 530 | 59774.3 | 5.86 | 42.94 | 108.11 | -0.016 |
| NL-FPE.chr3.3367 | 3 | 2 | 3 | 2 | 642 | 73934.27 | 9.25 | 46.4 | 101.56 | -0.204 |
| NL-FPE.chr3.4014 | 1 | 0 | 1 | 0 | 781 | 88334.35 | 6.16 | 47.66 | 105.81 | -0.103 |
| NL-FPE.chr3.434 | 5 | 4 | 5 | 0 | 890 | 100971.96 | 5.92 | 33.93 | 101.61 | -0.148 |
| NL-FPE.chr3.611 | 5 | 4 | 5 | 0 | 702 | 79959.7 | 7.48 | 55.32 | 100.37 | -0.178 |
| NL-FPE.chr7.2369 | 4 | 3 | 4 | 0 | 952 | 108162.59 | 5.68 | 49 | 96.14 | -0.137 |
| NL-FPE.chr7.793 | 2 | 1 | 2 | 0 | 479 | 55168.11 | 6.15 | 42.48 | 110.06 | -0.022 |
| NL-FvH4.1g22800.1 | 2 | 1 | 2 | 0 | 456 | 51783.75 | 5.69 | 49.75 | 102.85 | -0.206 |
| NL-FvH4.3g35890.1 | 5 | 4 | 3 | 3 | 966 | 109825.29 | 6.22 | 37.42 | 101.8 | -0.061 |
| NL-FvH4.3g37980.1 | 2 | 1 | 2 | 0 | 652 | 74671.65 | 6.04 | 48.81 | 99.88 | -0.104 |
| NL-FvH4.4g15190.1 | 5 | 4 | 5 | 0 | 239 | 26705.51 | 4.89 | 39.97 | 86.53 | -0.423 |
| NL-FvH4.4g18270.1 | 10 | 9 | 3 | 9 | 964 | 108369.18 | 6.66 | 47.58 | 102.06 | -0.109 |
| NL-FvH4.4g21900.1 | 5 | 4 | 5 | 0 | 661 | 74885.08 | 6.68 | 40.49 | 96.7 | -0.182 |
| NL-FvH4.4g35430.1 | 6 | 5 | 4 | 4 | 1231 | 137757.33 | 6.39 | 41.51 | 105.78 | -0.116 |
| NL-FvH4.5g33110.1 | 4 | 3 | 4 | 0 | 427 | 48922.53 | 5.95 | 46.23 | 99.7 | -0.281 |
| NL-FvH4.5g38760.1 | 7 | 6 | 7 | 2 | 1394 | 157887.51 | 5.76 | 46.78 | 91.46 | -0.187 |
| NL-FvH4.6g04350.1 | 4 | 3 | 4 | 0 | 941 | 107409.62 | 8.18 | 44.84 | 95.36 | -0.212 |
| NL-FvH4.6g05490.1 | 4 | 3 | 4 | 2 | 934 | 106558.87 | 7.23 | 50.23 | 97.24 | -0.177 |
| IDSequence | Num. of Exon | Num. of Intron | Num. of CDS | Num. of UTR | Num. of Amino Acid | Molecular Weight | Theoretical pI | Instability Index | Aliphatic Index | Grand Average of Hydropathicity |
| NL-FvH4.6g22120.1 | 5 | 4 | 1 | 6 | 1297 | 146507.4 | 5.65 | 41.21 | 105.64 | -0.143 |
| NL-FvH4.7g00850.1 | 1 | 0 | 1 | 0 | 1204 | 136675.01 | 6.59 | 47.81 | 98.11 | -0.15 |
| NL-FVI.CHR2.3163 | 4 | 3 | 4 | 0 | 766 | 86597.54 | 8.65 | 57.88 | 100.13 | -0.271 |
| NL-FVI.CHR2.81 | 9 | 8 | 9 | 0 | 563 | 64126.71 | 5.69 | 44.54 | 93.11 | -0.226 |
| NL-FVI.CHR3.187 | 3 | 2 | 3 | 0 | 818 | 93008.69 | 8.77 | 40.1 | 102.1 | -0.242 |
| NL-FVI.CHR4.1309 | 3 | 2 | 1 | 4 | 564 | 64159.85 | 8.09 | 40.4 | 105.53 | -0.041 |
| NL-FVI.CHR4.1998 | 4 | 3 | 2 | 3 | 687 | 79539.18 | 7.54 | 41.9 | 101.83 | -0.221 |
| NL-FVI.CHR4.2250 | 3 | 2 | 2 | 3 | 779 | 88615.87 | 8.07 | 47.05 | 99.5 | -0.178 |
| NL-FVI.CHR6.1794 | 8 | 7 | 5 | 5 | 1063 | 119553.97 | 7.63 | 45.04 | 102.63 | -0.147 |
| NL-FVI.CHR6.2190 | 3 | 2 | 1 | 3 | 1297 | 146543.62 | 5.91 | 44.19 | 104.07 | -0.171 |
| NL-FVI.CHR7.2360 | 4 | 3 | 4 | 0 | 819 | 92981.6 | 6.11 | 40.42 | 103.79 | -0.141 |
| NL-FVI.CHR7.880 | 6 | 5 | 6 | 2 | 1012 | 114395.01 | 6.11 | 37.26 | 95.6 | -0.223 |
| RN-FDA.chr1.22178 | 8 | 7 | 8 | 0 | 624 | 70842.96 | 6.05 | 35.31 | 96.83 | -0.235 |
| RN-FDA.chr1.22186 | 4 | 3 | 4 | 0 | 388 | 44236.82 | 8.15 | 47.66 | 89.38 | -0.398 |
| RN-FDA.chr6.13755 | 5 | 4 | 5 | 0 | 792 | 90864.85 | 6.55 | 40.81 | 100.52 | -0.194 |
| RN-FII.chr3.1087 | 2 | 1 | 2 | 0 | 193 | 21804.87 | 9.23 | 47.7 | 82.23 | -0.356 |
| RN-FII.chr5.904 | 1 | 0 | 1 | 2 | 647 | 74146.36 | 4.81 | 61.07 | 94.7 | -0.384 |
| RN-FII.chr6.3558 | 6 | 5 | 6 | 0 | 580 | 65474.66 | 6.13 | 39.5 | 101.98 | -0.124 |
| RN-FNG.chr1.1331 | 9 | 8 | 9 | 0 | 1082 | 122461.69 | 5.7 | 42.32 | 99.82 | -0.194 |
| RN-FNG.chr5.1035 | 1 | 0 | 1 | 2 | 645 | 73889.08 | 4.89 | 59.12 | 96.67 | -0.384 |
| RN-FNU.ctg109.400 | 6 | 5 | 6 | 0 | 495 | 55514.73 | 5.86 | 39.12 | 100.04 | -0.214 |
| RN-FNU.ctg172.29 | 2 | 1 | 2 | 0 | 340 | 38818.88 | 7.65 | 36.81 | 93.35 | -0.286 |
| RN-FNU.ctg172.34 | 4 | 3 | 4 | 0 | 257 | 29164.97 | 6.54 | 52.77 | 93 | -0.118 |
| RN-FNU.ctg172.42 | 5 | 4 | 5 | 0 | 443 | 50702.3 | 5.1 | 48.97 | 104.06 | -0.16 |
| RN-FNU.ctg172.45 | 4 | 3 | 4 | 0 | 412 | 45828.47 | 5.23 | 26.72 | 100.41 | -0.299 |
| RN-FNU.ctg38.153 | 5 | 4 | 5 | 0 | 510 | 57172.3 | 8.76 | 44.12 | 87.49 | -0.297 |
| RN-FNU.ctg52.52 | 5 | 4 | 5 | 2 | 795 | 91169.16 | 6 | 44.4 | 97.58 | -0.213 |
| RN-FNU.ctg55.225 | 9 | 8 | 9 | 0 | 586 | 65619.87 | 5.95 | 36.64 | 91.31 | -0.317 |
| RN-FNU.ctg81.619 | 3 | 2 | 3 | 0 | 340 | 38521.46 | 8.72 | 30.39 | 90.03 | -0.356 |
| RN-FPE.chr6.6309 | 4 | 3 | 4 | 0 | 492 | 56393.06 | 6.11 | 41.54 | 92.26 | -0.34 |
| RN-FvH4.1g15140.1 | 4 | 3 | 4 | 0 | 477 | 54723.28 | 6.27 | 37.9 | 94.13 | -0.307 |
| RN-FvH4.1g15200.1 | 5 | 4 | 2 | 5 | 267 | 29995.87 | 8.69 | 40.83 | 94.08 | -0.276 |
| RN-FvH4.1g15240.1 | 7 | 6 | 6 | 3 | 602 | 68027.17 | 6.05 | 35.53 | 95.6 | -0.356 |
| RN-FvH4.6g42370.1 | 7 | 6 | 7 | 0 | 517 | 58995.04 | 6.5 | 40.26 | 100.46 | -0.272 |
| RN-FvH4.6g50330.1 | 6 | 5 | 4 | 3 | 359 | 40482.03 | 5.88 | 42.26 | 97.99 | -0.205 |
| RN-FVI.CHR1.1321 | 6 | 5 | 5 | 3 | 595 | 67028.04 | 5.48 | 40.66 | 99.18 | -0.283 |
| RNL-FDA.chr1.22179 | 5 | 4 | 5 | 0 | 884 | 100688.18 | 5.92 | 48.34 | 98.11 | -0.252 |
| RNL-FDA.chr1.22182 | 5 | 4 | 5 | 0 | 820 | 93685.83 | 5.36 | 42.87 | 108.61 | -0.074 |
| RNL-FDA.chr1.22183 | 7 | 6 | 7 | 0 | 866 | 98439.11 | 5.55 | 47.07 | 103.75 | -0.095 |
| RNL-FDA.chr1.22184 | 5 | 4 | 5 | 0 | 734 | 82850.31 | 8.97 | 41.56 | 100 | -0.155 |
| RNL-FDA.chr1.22187 | 5 | 4 | 5 | 0 | 850 | 96507.47 | 5.87 | 36.66 | 100.65 | -0.22 |
| RNL-FDA.chr1.22190 | 15 | 14 | 15 | 0 | 1507 | 170719.56 | 6.12 | 42.11 | 99.54 | -0.204 |
| IDSequence | Num. of Exon | Num. of Intron | Num. of CDS | Num. of UTR | Num. of Amino Acid | Molecular Weight | Theoretical pI | Instability Index | Aliphatic Index | Grand Average of Hydropathicity |
| RNL-FDA.chr1.22196 | 21 | 20 | 21 | 0 | 1956 | 219725.15 | 6 | 45.21 | 90.14 | -0.353 |
| RNL-FDA.chr1.22201 | 14 | 13 | 14 | 0 | 1237 | 142731 | 6.68 | 50.84 | 96.5 | -0.233 |
| RNL-FDA.chr2.11777 | 5 | 4 | 5 | 0 | 819 | 92692.73 | 6.11 | 49.92 | 105.81 | -0.074 |
| RNL-FDA.chr4.04994 | 4 | 3 | 4 | 0 | 882 | 100448.72 | 7.92 | 39.9 | 102.68 | -0.161 |
| RNL-FDA.chr6.12990 | 5 | 4 | 5 | 0 | 854 | 97664.54 | 6.83 | 39.73 | 102.04 | -0.202 |
| RNL-FDA.chr6.13739 | 15 | 14 | 15 | 0 | 1978 | 226810.66 | 5.33 | 45.52 | 102.29 | -0.22 |
| RNL-FII.chr1.1265 | 5 | 4 | 5 | 0 | 847 | 95826.56 | 5.7 | 39.42 | 107.44 | -0.1 |
| RNL-FII.chr1.1266 | 5 | 4 | 5 | 0 | 879 | 99782.18 | 6.01 | 48.1 | 100.99 | -0.207 |
| RNL-FII.chr1.1267 | 5 | 4 | 5 | 2 | 837 | 94746.82 | 6.03 | 37.99 | 100.73 | -0.184 |
| RNL-FII.chr1.1269 | 6 | 5 | 6 | 2 | 814 | 93010.94 | 5.62 | 46.86 | 101.51 | -0.135 |
| RNL-FII.chr1.1270 | 7 | 6 | 6 | 3 | 893 | 101465.18 | 6.2 | 38.26 | 102.41 | -0.28 |
| RNL-FII.chr2.1682 | 6 | 5 | 5 | 2 | 800 | 90746.31 | 6.91 | 43.94 | 104.54 | -0.275 |
| RNL-FII.chr2.3467 | 5 | 4 | 5 | 2 | 819 | 92553.42 | 5.99 | 49.55 | 106.17 | -0.082 |
| RNL-FMA.chr1.1806 | 12 | 11 | 12 | 0 | 1640 | 187932.06 | 5.55 | 41.26 | 105.38 | -0.114 |
| RNL-FMA.chr1.1807 | 5 | 4 | 5 | 2 | 883 | 100373.26 | 6.37 | 45.07 | 100.99 | -0.204 |
| RNL-FMA.chr1.1808 | 8 | 7 | 5 | 5 | 846 | 95894.38 | 5.59 | 39.95 | 104.68 | -0.129 |
| RNL-FMA.chr1.1810 | 5 | 4 | 5 | 0 | 799 | 90013.79 | 6.01 | 42.71 | 108.54 | -0.022 |
| RNL-FMA.chr2.1492 | 5 | 4 | 5 | 0 | 805 | 92050.64 | 8.46 | 44 | 101.84 | -0.318 |
| RNL-FMA.chr2.3489 | 5 | 4 | 5 | 2 | 820 | 92748.65 | 6.17 | 50.95 | 106.52 | -0.079 |
| RNL-FNG.chr1.1333 | 14 | 13 | 13 | 2 | 1626 | 182846.34 | 5.77 | 47.32 | 94.35 | -0.323 |
| RNL-FNG.chr1.1336 | 6 | 5 | 5 | 3 | 790 | 89607.27 | 8.68 | 43.27 | 102.08 | -0.229 |
| RNL-FNG.chr1.1337 | 6 | 5 | 6 | 2 | 806 | 92029 | 6.32 | 45.46 | 104.84 | -0.065 |
| RNL-FNG.chr1.1344 | 14 | 13 | 14 | 0 | 1153 | 133465.4 | 6.78 | 48.9 | 96.26 | -0.238 |
| RNL-FNG.chr2.1787 | 5 | 4 | 5 | 2 | 793 | 90302.9 | 8.09 | 47.08 | 103.62 | -0.281 |
| RNL-FNG.chr2.3537 | 5 | 4 | 5 | 2 | 813 | 91972.72 | 5.87 | 51.15 | 106.47 | -0.078 |
| RNL-FNG.chr6.3533 | 6 | 5 | 6 | 1 | 946 | 107314.28 | 6.74 | 40.06 | 99.32 | -0.217 |
| RNL-FNG.chr6.4113 | 5 | 4 | 5 | 0 | 875 | 100521.88 | 5.72 | 42.32 | 107.71 | -0.197 |
| RNL-FNG.chr6.4114 | 7 | 6 | 7 | 0 | 1224 | 139629.62 | 5.83 | 43.17 | 109 | -0.123 |
| RNL-FNU.ctg122.114 | 5 | 4 | 5 | 2 | 819 | 92646.56 | 6.27 | 49.07 | 106.15 | -0.09 |
| RNL-FNU.ctg172.65 | 7 | 6 | 7 | 1 | 881 | 101220.72 | 7.77 | 50.03 | 101.95 | -0.154 |
| RNL-FPE.chr0.2431 | 5 | 4 | 5 | 0 | 814 | 92165.01 | 6.08 | 49.93 | 106.57 | -0.08 |
| RNL-FPE.chr5.2037 | 5 | 4 | 5 | 0 | 812 | 91822.78 | 5.3 | 42.41 | 96.28 | -0.242 |
| RNL-FPE.chr5.2061 | 5 | 4 | 5 | 1 | 798 | 90794.27 | 6.56 | 44.69 | 104.06 | -0.278 |
| RNL-FPE.chr6.3526 | 5 | 4 | 5 | 0 | 777 | 89098.62 | 6.19 | 47.36 | 104.23 | -0.089 |
| RNL-FPE.chr6.5624 | 5 | 4 | 5 | 0 | 810 | 91643.83 | 5.28 | 41.39 | 98.68 | -0.184 |
| RNL-FPE.chr6.6304 | 11 | 10 | 11 | 1 | 1334 | 151355.89 | 5.71 | 43.76 | 103.2 | -0.202 |
| RNL-FvH4.1g15090.1 | 6 | 5 | 6 | 2 | 838 | 94329.87 | 6.19 | 45.32 | 107.33 | -0.013 |
| RNL-FvH4.1g15120.1 | 8 | 7 | 8 | 2 | 909 | 102977.4 | 5.67 | 39.39 | 103.01 | -0.139 |
| RNL-FvH4.1g15130.1 | 5 | 4 | 5 | 0 | 883 | 100373.26 | 6.37 | 45.07 | 100.99 | -0.204 |
| RNL-FvH4.1g15160.1 | 7 | 6 | 6 | 3 | 818 | 93353.38 | 5.27 | 43.07 | 108.88 | -0.074 |
| RNL-FvH4.1g15170.1 | 7 | 6 | 6 | 3 | 816 | 93063.84 | 5.35 | 47.85 | 102.7 | -0.129 |
| RNL-FvH4.1g15210.1 | 5 | 4 | 5 | 2 | 855 | 97080.21 | 5.7 | 36.84 | 100.53 | -0.207 |
| IDSequence | Num. of Exon | Num. of Intron | Num. of CDS | Num. of UTR | Num. of Amino Acid | Molecular Weight | Theoretical pI | Instability Index | Aliphatic Index | Grand Average of Hydropathicity |
| RNL-FvH4.1g15220.1 | 6 | 5 | 6 | 1 | 865 | 98540.56 | 5.59 | 48.23 | 109.49 | -0.071 |
| RNL-FvH4.1g15230.1 | 6 | 5 | 6 | 2 | 815 | 93019.83 | 5.46 | 46.86 | 101.5 | -0.126 |
| RNL-FvH4.1g15330.1 | 13 | 12 | 12 | 2 | 1454 | 163765.53 | 5.83 | 46.64 | 95.33 | -0.312 |
| RNL-FvH4.1g15380.1 | 6 | 5 | 5 | 3 | 815 | 93223.59 | 8.57 | 45.46 | 99.6 | -0.263 |
| RNL-FvH4.1g15390.1 | 6 | 5 | 6 | 2 | 813 | 93601.13 | 7.02 | 43.88 | 106.03 | -0.127 |
| RNL-FvH4.1g15500.1 | 13 | 12 | 12 | 3 | 1253 | 144345.07 | 7.52 | 51.55 | 97.77 | -0.202 |
| RNL-FvH4.1g17470.1 | 5 | 4 | 5 | 2 | 780 | 89079.1 | 7.16 | 43.1 | 106.06 | -0.141 |
| RNL-FvH4.2g40720.1 | 5 | 4 | 5 | 2 | 820 | 92742.68 | 6.17 | 50.56 | 107.23 | -0.075 |
| RNL-FvH4.4g31860.1 | 5 | 4 | 5 | 2 | 838 | 95451.73 | 5.87 | 41.45 | 101.58 | -0.195 |
| RNL-FvH4.6g18970.1 | 5 | 4 | 5 | 2 | 789 | 90337.15 | 6.42 | 46.64 | 101.67 | -0.13 |
| RNL-FvH4.6g42380.1 | 4 | 3 | 4 | 2 | 897 | 102358.91 | 6.92 | 41 | 101.93 | -0.154 |
| RNL-FvH4.6g50100.1 | 5 | 4 | 5 | 1 | 852 | 97855.86 | 5 | 45.26 | 107.98 | -0.119 |
| RNL-FvH4.6g50110.1 | 6 | 5 | 6 | 0 | 935 | 106858.46 | 8.03 | 41.75 | 104.97 | -0.183 |
| RNL-FvH4.6g50130.1 | 6 | 5 | 5 | 3 | 906 | 103463.89 | 6.34 | 42.29 | 108.42 | -0.079 |
| RNL-FvH4.6g50140.1 | 5 | 4 | 5 | 2 | 955 | 109418.19 | 5.69 | 42.36 | 103.16 | -0.188 |
| RNL-FvH4.6g50310.1 | 11 | 10 | 11 | 2 | 1465 | 168271.1 | 8.17 | 47.71 | 97.43 | -0.269 |
| RNL-FVI.CHR1.1325 | 14 | 13 | 13 | 3 | 1626 | 182575.01 | 5.67 | 45.58 | 95.92 | -0.302 |
| RNL-FVI.CHR1.1341 | 14 | 13 | 14 | 2 | 1236 | 142420.72 | 6.68 | 53.11 | 95.4 | -0.226 |
| RNL-FVI.CHR2.4287 | 5 | 4 | 5 | 2 | 821 | 92923.88 | 6.11 | 50.34 | 104.59 | -0.102 |
| RNL-FVI.CHR6.3601 | 6 | 5 | 5 | 3 | 803 | 90165.28 | 7.98 | 40.18 | 103.33 | -0.242 |
| TN-FDA.chr2.08147 | 2 | 1 | 2 | 0 | 423 | 47567.06 | 6.54 | 41.86 | 92.1 | -0.168 |
| TN-FDA.chr2.12371 | 2 | 1 | 2 | 0 | 551 | 63535.73 | 6.24 | 41.22 | 97.6 | -0.245 |
| TN-FDA.chr3.05844 | 3 | 2 | 3 | 0 | 631 | 71664.58 | 7.25 | 46.58 | 92.42 | -0.198 |
| TN-FDA.chr3.05876 | 2 | 1 | 2 | 0 | 293 | 33421.29 | 6.46 | 39.41 | 81.84 | -0.309 |
| TN-FDA.chr3.09422 | 2 | 1 | 2 | 0 | 536 | 61301.01 | 5.87 | 45.61 | 97.95 | -0.199 |
| TN-FDA.chr3.09564 | 4 | 3 | 4 | 0 | 346 | 39221.16 | 9 | 41.38 | 92.46 | -0.31 |
| TN-FDA.chr3.15822 | 2 | 1 | 2 | 0 | 512 | 57518.8 | 5.78 | 42.53 | 99.96 | -0.156 |
| TN-FDA.chr3.20717 | 6 | 5 | 6 | 0 | 949 | 107919.78 | 5.61 | 45.88 | 91.17 | -0.202 |
| TN-FDA.chr3.20720 | 3 | 2 | 3 | 0 | 622 | 71025.79 | 7.3 | 39.14 | 96.35 | -0.251 |
| TN-FDA.chr3.20724 | 3 | 2 | 3 | 0 | 554 | 63074.96 | 6.06 | 38.43 | 93.92 | -0.303 |
| TN-FDA.chr3.20745 | 4 | 3 | 4 | 0 | 346 | 39352.99 | 5.85 | 41.13 | 86.27 | -0.442 |
| TN-FDA.chr5.03486 | 2 | 1 | 2 | 0 | 463 | 52729.93 | 6.26 | 50.72 | 92.61 | -0.309 |
| TN-FDA.chr5.23634 | 2 | 1 | 2 | 0 | 541 | 61987.6 | 6.3 | 42.98 | 93.48 | -0.343 |
| TN-FDA.chr6.02329 | 2 | 1 | 2 | 0 | 318 | 36677.39 | 6.84 | 36.25 | 95.6 | -0.147 |
| TN-FDA.chr6.02460 | 2 | 1 | 2 | 0 | 279 | 32152.05 | 6.84 | 38.36 | 94.98 | -0.207 |
| TN-FDA.chr6.26557 | 3 | 2 | 3 | 0 | 854 | 96294.45 | 8.04 | 41.65 | 93.78 | -0.252 |
| TN-FDA.chr7.00118 | 2 | 1 | 2 | 0 | 507 | 58767.33 | 5.61 | 44.28 | 93.98 | -0.208 |
| TN-FDA.chr7.00120 | 2 | 1 | 2 | 0 | 533 | 61106.83 | 5.34 | 44.01 | 95.07 | -0.197 |
| TN-FDA.chr7.00191 | 2 | 1 | 2 | 0 | 316 | 36535.98 | 5.4 | 41.57 | 94.97 | -0.103 |
| TN-FDA.chr7.00192 | 3 | 2 | 3 | 0 | 550 | 63287.52 | 5.4 | 45.21 | 95.67 | -0.18 |
| TN-FDA.chr7.02893 | 2 | 1 | 2 | 0 | 435 | 49594.09 | 8.28 | 39.85 | 93.68 | -0.283 |
| TN-FDA.chr7.21088 | 2 | 1 | 2 | 0 | 565 | 64330.93 | 9.06 | 43.06 | 93.43 | -0.268 |
| IDSequence | Num. of Exon | Num. of Intron | Num. of CDS | Num. of UTR | Num. of Amino Acid | Molecular Weight | Theoretical pI | Instability Index | Aliphatic Index | Grand Average of Hydropathicity |
| TN-FII.chr1.2339 | 2 | 1 | 2 | 2 | 410 | 46987.15 | 6.36 | 36.67 | 85.56 | -0.382 |
| TN-FII.chr2.1653 | 2 | 1 | 2 | 0 | 366 | 41376.31 | 7.75 | 32.87 | 93.74 | -0.295 |
| TN-FII.chr2.560 | 5 | 4 | 4 | 3 | 711 | 81547.15 | 5.74 | 39.37 | 104.32 | -0.031 |
| TN-FII.chr2.633 | 3 | 2 | 3 | 0 | 637 | 72542.13 | 6.46 | 44.28 | 94.66 | -0.284 |
| TN-FII.chr3.1974 | 3 | 2 | 3 | 0 | 392 | 44477.58 | 5.81 | 43.59 | 83.49 | -0.341 |
| TN-FII.chr5.2853 | 2 | 1 | 2 | 0 | 410 | 46560.16 | 7.15 | 44.99 | 91.32 | -0.272 |
| TN-FII.chr5.2971 | 6 | 5 | 5 | 2 | 645 | 73875.89 | 6 | 50.56 | 101.97 | -0.161 |
| TN-FII.chr6.4140 | 7 | 6 | 7 | 0 | 434 | 48896.18 | 8.53 | 41.9 | 97.72 | -0.244 |
| TN-FII.chr7.888 | 3 | 2 | 3 | 0 | 430 | 48578.4 | 5.91 | 52.43 | 92.88 | -0.331 |
| TN-FII.chr7.937 | 3 | 2 | 2 | 2 | 196 | 21950.53 | 9.3 | 41 | 97.45 | -0.046 |
| TN-FII.chr7.977 | 2 | 1 | 2 | 2 | 392 | 44414.97 | 6.63 | 39.25 | 91.28 | -0.284 |
| TN-FMA.chr0.347 | 2 | 1 | 2 | 0 | 367 | 42017.82 | 8.19 | 38.62 | 90.33 | -0.375 |
| TN-FMA.chr3.2485 | 2 | 1 | 2 | 0 | 327 | 37496.74 | 5.11 | 36.88 | 92.97 | -0.222 |
| TN-FMA.chr3.3317 | 2 | 1 | 2 | 0 | 578 | 65953.76 | 5.77 | 47.53 | 90.43 | -0.288 |
| TN-FMA.chr5.2917 | 4 | 3 | 4 | 0 | 650 | 73365.41 | 6.02 | 50.95 | 83.37 | -0.464 |
| TN-FNG.chr1.1802 | 2 | 1 | 2 | 1 | 410 | 47127.45 | 6.58 | 35.77 | 86.76 | -0.385 |
| TN-FNG.chr2.1155 | 2 | 1 | 2 | 0 | 440 | 50351.96 | 6.26 | 36.35 | 100.11 | -0.148 |
| TN-FNG.chr3.1991 | 2 | 1 | 2 | 0 | 168 | 18916.91 | 6.37 | 38.69 | 98.57 | -0.182 |
| TN-FNG.chr3.2312 | 2 | 1 | 2 | 0 | 291 | 33527.81 | 8.85 | 42 | 93.78 | -0.185 |
| TN-FNG.chr3.2354 | 3 | 2 | 3 | 0 | 589 | 67860.83 | 5.94 | 33.8 | 94.67 | -0.151 |
| TN-FNG.chr3.760 | 2 | 1 | 2 | 0 | 385 | 42350.57 | 6.99 | 35.34 | 92.26 | -0.144 |
| TN-FNG.chr4.253 | 2 | 1 | 2 | 2 | 578 | 65405.45 | 5.77 | 43.93 | 95.28 | -0.21 |
| TN-FNG.chr7.1408 | 2 | 1 | 2 | 0 | 291 | 33145.1 | 6.55 | 43.95 | 98.8 | -0.261 |
| TN-FNG.chr7.573 | 4 | 3 | 3 | 2 | 532 | 60684.42 | 9.03 | 50.65 | 101.47 | -0.213 |
| TN-FNU.ctg10.27 | 3 | 2 | 3 | 0 | 436 | 50039.46 | 8.56 | 34.35 | 107.52 | -0.15 |
| TN-FNU.ctg173.411 | 2 | 1 | 2 | 0 | 387 | 44324.32 | 6.23 | 39.23 | 87.39 | -0.358 |
| TN-FNU.ctg173.413 | 2 | 1 | 2 | 0 | 367 | 42231.66 | 8.5 | 35.41 | 100.05 | -0.21 |
| TN-FNU.ctg177.55 | 3 | 2 | 2 | 3 | 410 | 47218.54 | 6.4 | 36.01 | 85.56 | -0.395 |
| TN-FNU.ctg8.251 | 3 | 2 | 3 | 1 | 352 | 40600.88 | 6.36 | 37.46 | 100.2 | -0.232 |
| TN-FNU.ctg84.632 | 3 | 2 | 3 | 0 | 442 | 49405.4 | 6.08 | 40.12 | 92.85 | -0.188 |
| TN-FPE.chr1.445 | 3 | 2 | 3 | 0 | 228 | 25515.5 | 8.73 | 34.83 | 103.42 | -0.05 |
| TN-FPE.chr4.1012 | 2 | 1 | 2 | 0 | 401 | 45191.24 | 5.7 | 33.57 | 102.09 | -0.134 |
| TN-FPE.chr5.245 | 4 | 3 | 4 | 0 | 500 | 57072.67 | 6.87 | 46.18 | 95.72 | -0.25 |
| TN-FPE.chr5.3159 | 2 | 1 | 2 | 0 | 556 | 62790.73 | 5.52 | 39.94 | 97.68 | -0.197 |
| TN-FPE.chr6.5875 | 5 | 4 | 2 | 5 | 438 | 50024.39 | 7.02 | 45.75 | 98.11 | -0.234 |
| TN-FPE.chr7.406 | 3 | 2 | 3 | 0 | 187 | 21232.7 | 5.69 | 40.19 | 80.86 | -0.431 |
| TN-FvH4.1g22520.1 | 5 | 4 | 5 | 0 | 534 | 61131.4 | 6.95 | 43.59 | 94.72 | -0.195 |
| TN-FvH4.1g27020.1 | 2 | 1 | 2 | 2 | 410 | 47099.26 | 6.32 | 37.09 | 85.07 | -0.4 |
| TN-FvH4.2g20690.1 | 2 | 1 | 2 | 0 | 268 | 30254.36 | 6.2 | 36.43 | 86.6 | -0.375 |
| TN-FvH4.3g09290.1 | 9 | 8 | 2 | 9 | 382 | 43126.37 | 7.25 | 37.58 | 95.92 | -0.249 |
| TN-FvH4.3g35840.1 | 3 | 2 | 3 | 0 | 305 | 34614.44 | 6.99 | 47.87 | 81.25 | -0.452 |
| TN-FvH4.3g45630.1 | 5 | 4 | 2 | 5 | 548 | 62430.33 | 6.47 | 47.65 | 84.01 | -0.416 |
| IDSequence | Num. of Exon | Num. of Intron | Num. of CDS | Num. of UTR | Num. of Amino Acid | Molecular Weight | Theoretical pI | Instability Index | Aliphatic Index | Grand Average of Hydropathicity |
| TN-FvH4.5g07190.1 | 3 | 2 | 3 | 0 | 347 | 40341.62 | 6.02 | 34.2 | 87.03 | -0.432 |
| TN-FvH4.5g32990.1 | 6 | 5 | 6 | 0 | 560 | 63730.27 | 9.12 | 44.65 | 92.61 | -0.296 |
| TN-FvH4.5g34230.1 | 2 | 1 | 2 | 0 | 413 | 46907.68 | 6.88 | 42.54 | 96.03 | -0.166 |
| TN-FvH4.5g34240.1 | 2 | 1 | 2 | 0 | 288 | 32338.56 | 5.84 | 52.46 | 87.36 | -0.263 |
| TN-FvH4.6g01300.1 | 3 | 2 | 3 | 1 | 344 | 39234.01 | 8.56 | 43.51 | 83.9 | -0.452 |
| TN-FvH4.6g39860.1 | 3 | 2 | 2 | 3 | 660 | 75586.61 | 6.8 | 46.26 | 88.18 | -0.295 |
| TN-FvH4.6g49490.1 | 10 | 9 | 10 | 2 | 826 | 92716.61 | 8 | 36.44 | 98.75 | -0.158 |
| TN-FvH4.6g49500.1 | 5 | 4 | 5 | 2 | 417 | 47497.79 | 8 | 41.21 | 104.7 | -0.164 |
| TN-FvH4.7g11160.1 | 13 | 12 | 2 | 13 | 336 | 37690.02 | 6.27 | 38.16 | 88.15 | -0.286 |
| TN-FvH4.7g11580.1 | 4 | 3 | 4 | 0 | 335 | 37710.02 | 8.04 | 50.96 | 82.36 | -0.467 |
| TN-FvH4.7g28520.1 | 3 | 2 | 3 | 0 | 315 | 35767.84 | 6.39 | 50.82 | 87.87 | -0.375 |
| TN-FvH4.7g32720.1 | 2 | 1 | 2 | 0 | 493 | 56506.7 | 5.67 | 37.5 | 94.69 | -0.215 |
| TN-FVI.CHR1.2577 | 4 | 3 | 2 | 3 | 323 | 37330.26 | 6.06 | 36.55 | 88.92 | -0.427 |
| TN-FVI.CHR4.939 | 7 | 6 | 7 | 0 | 543 | 62662.17 | 7.93 | 50.66 | 92.21 | -0.316 |
| TN-FVI.CHR5.1806 | 2 | 1 | 2 | 0 | 451 | 51337.5 | 5.79 | 38.78 | 91.62 | -0.334 |
| TN-FVI.CHR5.2927 | 7 | 6 | 7 | 1 | 845 | 96371.23 | 6.29 | 58.04 | 95.72 | -0.269 |
| TN-FVI.CHR7.995 | 3 | 2 | 3 | 0 | 513 | 58685.15 | 8.79 | 48.44 | 85.69 | -0.42 |
| TNL-FDA.chr1.04275 | 11 | 10 | 11 | 0 | 947 | 107466.85 | 6.41 | 43.27 | 99.33 | -0.139 |
| TNL-FDA.chr1.07161 | 5 | 4 | 5 | 0 | 1115 | 126313.04 | 6.99 | 42.45 | 96.41 | -0.207 |
| TNL-FDA.chr1.15663 | 5 | 4 | 5 | 0 | 1303 | 145221 | 5.73 | 38.57 | 110.22 | -0.014 |
| TNL-FDA.chr1.17976 | 4 | 3 | 4 | 0 | 729 | 83298.15 | 6.83 | 47.29 | 100.88 | -0.16 |
| TNL-FDA.chr1.17978 | 4 | 3 | 4 | 0 | 847 | 96181.29 | 6.58 | 42.73 | 104.79 | -0.109 |
| TNL-FDA.chr1.17980 | 5 | 4 | 5 | 0 | 1363 | 153295.15 | 6.22 | 45.48 | 106.61 | -0.096 |
| TNL-FDA.chr1.17981 | 8 | 7 | 8 | 0 | 1879 | 213537.13 | 6.33 | 46.9 | 97.58 | -0.145 |
| TNL-FDA.chr2.10226 | 11 | 10 | 11 | 0 | 947 | 107186.37 | 6.08 | 46.49 | 97.97 | -0.224 |
| TNL-FDA.chr2.12354 | 4 | 3 | 4 | 0 | 747 | 84773.72 | 6.35 | 38.15 | 101.38 | -0.229 |
| TNL-FDA.chr2.12488 | 6 | 5 | 6 | 0 | 935 | 106489.22 | 8.05 | 44.84 | 102.53 | -0.207 |
| TNL-FDA.chr2.20071 | 7 | 6 | 7 | 0 | 1001 | 113561.19 | 6.13 | 47.4 | 97.07 | -0.228 |
| TNL-FDA.chr2.20072 | 6 | 5 | 6 | 0 | 1216 | 137706.73 | 6.55 | 55.99 | 97.78 | -0.247 |
| TNL-FDA.chr2.20138 | 5 | 4 | 5 | 0 | 1172 | 133912.83 | 6.81 | 45.37 | 94.56 | -0.301 |
| TNL-FDA.chr3.05636 | 16 | 15 | 16 | 0 | 1519 | 172500.8 | 6.69 | 41.09 | 98.49 | -0.135 |
| TNL-FDA.chr3.05639 | 2 | 1 | 2 | 0 | 992 | 112079.68 | 6.53 | 46.48 | 101.7 | -0.072 |
| TNL-FDA.chr3.05837 | 13 | 12 | 13 | 0 | 3047 | 345497.44 | 6.17 | 43.14 | 93.65 | -0.216 |
| TNL-FDA.chr3.05838 | 4 | 3 | 4 | 0 | 1153 | 131584.75 | 6.14 | 47.12 | 95.08 | -0.207 |
| TNL-FDA.chr3.05839 | 11 | 10 | 11 | 0 | 1280 | 146379.55 | 5.83 | 44.83 | 91.8 | -0.175 |
| TNL-FDA.chr3.05883 | 3 | 2 | 3 | 0 | 1028 | 117752.13 | 5.91 | 46.73 | 101.54 | -0.158 |
| TNL-FDA.chr3.05887 | 3 | 2 | 3 | 0 | 1072 | 122162.46 | 6.99 | 45.69 | 100.04 | -0.132 |
| TNL-FDA.chr3.05894 | 3 | 2 | 3 | 0 | 1117 | 128705 | 6.19 | 38.61 | 100.53 | -0.162 |
| TNL-FDA.chr3.09399 | 4 | 3 | 4 | 0 | 801 | 89768.17 | 6.62 | 31.22 | 112.38 | -0.014 |
| TNL-FDA.chr3.09421 | 5 | 4 | 5 | 0 | 1084 | 121781.92 | 6.75 | 43.33 | 91.55 | -0.263 |
| TNL-FDA.chr3.09474 | 4 | 3 | 4 | 0 | 1112 | 127415.92 | 7.09 | 44.37 | 97.15 | -0.165 |
| TNL-FDA.chr3.09523 | 5 | 4 | 5 | 0 | 1165 | 129940.21 | 6.25 | 42.61 | 105.66 | -0.044 |
| IDSequence | Num. of Exon | Num. of Intron | Num. of CDS | Num. of UTR | Num. of Amino Acid | Molecular Weight | Theoretical pI | Instability Index | Aliphatic Index | Grand Average of Hydropathicity |
| TNL-FDA.chr3.20669 | 5 | 4 | 5 | 0 | 1178 | 134623.88 | 6.87 | 46.95 | 94.38 | -0.296 |
| TNL-FDA.chr3.20670 | 5 | 4 | 5 | 0 | 2208 | 250701.69 | 5.25 | 43.83 | 99.62 | -0.114 |
| TNL-FDA.chr3.20674 | 3 | 2 | 3 | 0 | 1252 | 141870.86 | 4.98 | 45.6 | 99.07 | -0.162 |
| TNL-FDA.chr3.20677 | 3 | 2 | 3 | 0 | 1241 | 141307.03 | 5.84 | 50.22 | 91.56 | -0.368 |
| TNL-FDA.chr3.20678 | 3 | 2 | 3 | 0 | 1119 | 125663.36 | 6.57 | 39.21 | 100.42 | -0.067 |
| TNL-FDA.chr3.20719 | 5 | 4 | 5 | 0 | 1016 | 116494.48 | 6.22 | 45.16 | 95.51 | -0.167 |
| TNL-FDA.chr3.20725 | 6 | 5 | 6 | 0 | 1060 | 120930.38 | 5.92 | 47.99 | 91.55 | -0.233 |
| TNL-FDA.chr3.20747 | 5 | 4 | 5 | 0 | 1199 | 137035.62 | 5.87 | 47.45 | 98.84 | -0.142 |
| TNL-FDA.chr4.10670 | 5 | 4 | 5 | 0 | 1364 | 152719.03 | 6.28 | 41.71 | 107.3 | -0.018 |
| TNL-FDA.chr4.10758 | 5 | 4 | 5 | 0 | 1032 | 117267.98 | 4.9 | 40.05 | 93.22 | -0.198 |
| TNL-FDA.chr5.03487 | 7 | 6 | 7 | 0 | 973 | 110940.15 | 8.32 | 40.37 | 100.63 | -0.114 |
| TNL-FDA.chr5.03488 | 4 | 3 | 4 | 0 | 683 | 78305.93 | 7.93 | 44.76 | 97.17 | -0.253 |
| TNL-FDA.chr5.03613 | 6 | 5 | 6 | 0 | 1102 | 124950.38 | 8.32 | 45.13 | 99.56 | -0.123 |
| TNL-FDA.chr5.03707 | 8 | 7 | 8 | 0 | 1192 | 135071.86 | 4.92 | 41.61 | 92.84 | -0.328 |
| TNL-FDA.chr5.12803 | 9 | 8 | 9 | 0 | 1673 | 189783.89 | 6.33 | 44.83 | 97.85 | -0.094 |
| TNL-FDA.chr5.12815 | 9 | 8 | 9 | 0 | 1573 | 178174.48 | 8.1 | 42.88 | 97.59 | -0.22 |
| TNL-FDA.chr5.23994 | 5 | 4 | 5 | 0 | 839 | 95353.86 | 6.78 | 48.16 | 94.65 | -0.264 |
| TNL-FDA.chr5.25282 | 5 | 4 | 5 | 0 | 1275 | 144154.65 | 6.26 | 41.82 | 93.96 | -0.297 |
| TNL-FDA.chr5.25283 | 8 | 7 | 8 | 0 | 830 | 94208.84 | 5.9 | 39.69 | 97.12 | -0.174 |
| TNL-FDA.chr6.02345 | 7 | 6 | 7 | 0 | 1195 | 136392.62 | 6.48 | 43.66 | 95.81 | -0.215 |
| TNL-FDA.chr6.02361 | 5 | 4 | 5 | 0 | 1158 | 132500.46 | 7.94 | 45.75 | 95.26 | -0.178 |
| TNL-FDA.chr6.02439 | 4 | 3 | 4 | 0 | 1163 | 132063.66 | 5.93 | 44.41 | 103 | -0.037 |
| TNL-FDA.chr6.13989 | 5 | 4 | 5 | 0 | 2250 | 256560.59 | 6.48 | 47.16 | 100.14 | -0.176 |
| TNL-FDA.chr6.16370 | 9 | 8 | 9 | 0 | 1308 | 148284.34 | 5.57 | 45.02 | 98.13 | -0.15 |
| TNL-FDA.chr7.00085 | 4 | 3 | 4 | 0 | 1265 | 143794.38 | 6.26 | 48.07 | 99.94 | -0.128 |
| TNL-FDA.chr7.00110 | 5 | 4 | 5 | 0 | 791 | 90708.34 | 5.91 | 43.66 | 100.18 | -0.186 |
| TNL-FDA.chr7.00114 | 5 | 4 | 5 | 0 | 868 | 99175.83 | 6 | 46.41 | 96.59 | -0.167 |
| TNL-FDA.chr7.00159 | 5 | 4 | 5 | 0 | 1093 | 124058.58 | 5.78 | 42.92 | 98.01 | -0.135 |
| TNL-FDA.chr7.00184 | 6 | 5 | 6 | 0 | 994 | 113720.43 | 5.76 | 46.07 | 95.12 | -0.255 |
| TNL-FDA.chr7.00189 | 5 | 4 | 5 | 0 | 1109 | 126056.59 | 5.49 | 43.53 | 96.4 | -0.143 |
| TNL-FDA.chr7.00190 | 4 | 3 | 4 | 0 | 1103 | 125716.5 | 6 | 44.04 | 95.26 | -0.197 |
| TNL-FDA.chr7.00274 | 3 | 2 | 3 | 0 | 1827 | 208294.33 | 5.54 | 47.32 | 97.82 | -0.186 |
| TNL-FDA.chr7.00582 | 7 | 6 | 7 | 0 | 1332 | 151519.69 | 6.81 | 48.32 | 90.59 | -0.276 |
| TNL-FDA.chr7.00835 | 8 | 7 | 8 | 0 | 1337 | 151714.77 | 6.08 | 42.99 | 90.63 | -0.284 |
| TNL-FDA.chr7.01326 | 4 | 3 | 4 | 0 | 1118 | 127673.31 | 5.74 | 40.79 | 93 | -0.231 |
| TNL-FDA.chr7.01333 | 6 | 5 | 6 | 0 | 1113 | 126005.81 | 5.83 | 38.36 | 97 | -0.174 |
| TNL-FDA.chr7.01621 | 5 | 4 | 5 | 0 | 1240 | 141428.34 | 7.18 | 40.62 | 97.94 | -0.172 |
| TNL-FDA.chr7.02890 | 5 | 4 | 5 | 0 | 1178 | 132394.23 | 5.17 | 42.07 | 91.51 | -0.249 |
| TNL-FDA.chr7.02892 | 9 | 8 | 9 | 0 | 1996 | 226324.3 | 6.54 | 44.66 | 98.47 | -0.177 |
| TNL-FDA.chr7.14713 | 5 | 4 | 5 | 0 | 1200 | 135513.06 | 6.18 | 41.77 | 97.66 | -0.14 |
| TNL-FII.chr1.1393 | 4 | 3 | 4 | 1 | 1049 | 119031.14 | 6.01 | 46.74 | 101.12 | -0.154 |
| TNL-FII.chr1.1394 | 10 | 9 | 10 | 0 | 2492 | 281374.79 | 5.89 | 47.8 | 104.65 | -0.105 |
| IDSequence | Num. of Exon | Num. of Intron | Num. of CDS | Num. of UTR | Num. of Amino Acid | Molecular Weight | Theoretical pI | Instability Index | Aliphatic Index | Grand Average of Hydropathicity |
| TNL-FII.chr1.757 | 5 | 4 | 5 | 2 | 1376 | 153879.4 | 5.48 | 38.67 | 108.05 | -0.046 |
| TNL-FII.chr2.1158 | 7 | 6 | 7 | 2 | 1235 | 140994.47 | 5.98 | 44.47 | 92.92 | -0.221 |
| TNL-FII.chr2.1183 | 8 | 7 | 8 | 0 | 710 | 81281.55 | 5.52 | 47.69 | 92.68 | -0.305 |
| TNL-FII.chr2.172 | 8 | 7 | 8 | 2 | 1648 | 187059.34 | 6.58 | 50.24 | 94.93 | -0.187 |
| TNL-FII.chr2.1754 | 5 | 4 | 5 | 0 | 1117 | 126993.71 | 5.87 | 40.2 | 96.06 | -0.185 |
| TNL-FII.chr2.49 | 5 | 4 | 5 | 1 | 1189 | 135025.95 | 6.8 | 42.44 | 94.75 | -0.236 |
| TNL-FII.chr2.55 | 8 | 7 | 6 | 4 | 1770 | 200826.73 | 6.39 | 40.29 | 102.97 | -0.141 |
| TNL-FII.chr2.708 | 5 | 4 | 5 | 1 | 480 | 54439.83 | 6.01 | 40.16 | 94.65 | -0.195 |
| TNL-FII.chr2.77 | 5 | 4 | 4 | 3 | 1204 | 136649.66 | 6.38 | 39.14 | 93.47 | -0.228 |
| TNL-FII.chr3.1313 | 10 | 9 | 10 | 0 | 2247 | 250890.09 | 5.14 | 47.83 | 105.47 | -0.103 |
| TNL-FII.chr3.1814 | 3 | 2 | 3 | 2 | 1353 | 154674.09 | 6.87 | 42.6 | 96.47 | -0.201 |
| TNL-FII.chr3.1830 | 5 | 4 | 5 | 1 | 1225 | 138635.38 | 5.6 | 43.69 | 94.51 | -0.14 |
| TNL-FII.chr3.1831 | 6 | 5 | 5 | 3 | 1230 | 139740 | 6.45 | 41.35 | 92.15 | -0.157 |
| TNL-FII.chr3.3419 | 6 | 5 | 6 | 0 | 1647 | 184601.69 | 6.8 | 39.97 | 104.45 | -0.059 |
| TNL-FII.chr3.3603 | 5 | 4 | 5 | 2 | 1329 | 151070.86 | 5.38 | 44.5 | 92.45 | -0.273 |
| TNL-FII.chr3.3626 | 7 | 6 | 7 | 2 | 1523 | 173762.95 | 5.5 | 47.14 | 95.18 | -0.207 |
| TNL-FII.chr3.744.2 | 5 | 4 | 4 | 3 | 1410 | 157041.07 | 5.86 | 39.51 | 104.44 | -0.027 |
| TNL-FII.chr4.2875 | 8 | 7 | 8 | 0 | 1297 | 145554.19 | 5.85 | 42.71 | 100.93 | -0.215 |
| TNL-FII.chr4.2876 | 32 | 31 | 32 | 0 | 2651 | 298742.88 | 7.29 | 43.03 | 96.11 | -0.146 |
| TNL-FII.chr5.1800 | 6 | 5 | 6 | 0 | 809 | 91554.41 | 6.38 | 39.82 | 99.04 | -0.163 |
| TNL-FII.chr5.2851 | 5 | 4 | 5 | 1 | 1188 | 134095.76 | 8.23 | 42.15 | 98.66 | -0.111 |
| TNL-FII.chr5.2900 | 5 | 4 | 5 | 0 | 744 | 84558.75 | 7.19 | 41.8 | 98.9 | -0.162 |
| TNL-FII.chr5.2969 | 5 | 4 | 5 | 1 | 1074 | 121822.3 | 6.87 | 47.12 | 92.28 | -0.25 |
| TNL-FII.chr5.2970 | 6 | 5 | 6 | 2 | 1074 | 122204.34 | 8.74 | 42.13 | 100.16 | -0.102 |
| TNL-FII.chr5.302.2 | 5 | 4 | 5 | 2 | 1118 | 128231.21 | 6.76 | 46.8 | 93.6 | -0.263 |
| TNL-FII.chr5.3330 | 4 | 3 | 4 | 1 | 1042 | 117701.02 | 5.83 | 44.37 | 99.88 | -0.039 |
| TNL-FII.chr5.3343 | 7 | 6 | 7 | 0 | 971 | 109547.24 | 7.1 | 42.8 | 98.97 | -0.244 |
| TNL-FII.chr5.3370 | 5 | 4 | 5 | 0 | 1178 | 133503.15 | 6.26 | 46.53 | 93.57 | -0.249 |
| TNL-FII.chr5.3371 | 6 | 5 | 5 | 3 | 1200 | 136086.3 | 5.76 | 44.73 | 93.41 | -0.155 |
| TNL-FII.chr5.609 | 5 | 4 | 5 | 2 | 1136 | 130860.78 | 6.24 | 42.25 | 95.62 | -0.219 |
| TNL-FII.chr6.136 | 9 | 8 | 9 | 2 | 1478 | 167445.76 | 5.78 | 48.07 | 83.03 | -0.343 |
| TNL-FII.chr6.2998 | 7 | 6 | 7 | 0 | 1220 | 137875.39 | 6.23 | 50.01 | 102.15 | -0.116 |
| TNL-FII.chr6.322 | 6 | 5 | 6 | 0 | 1189 | 136036.39 | 8.29 | 44.8 | 95.39 | -0.23 |
| TNL-FII.chr7.1056 | 6 | 5 | 6 | 1 | 1162 | 131601.5 | 5.93 | 41.08 | 95.28 | -0.148 |
| TNL-FII.chr7.1130 | 17 | 16 | 17 | 0 | 1583 | 178921.06 | 7.17 | 54.12 | 95.36 | -0.186 |
| TNL-FII.chr7.2338 | 13 | 12 | 11 | 3 | 2061 | 233129.78 | 6.22 | 46.52 | 94.39 | -0.203 |
| TNL-FII.chr7.790 | 5 | 4 | 5 | 0 | 1159 | 132310.45 | 6.14 | 48.08 | 99.8 | -0.199 |
| TNL-FII.chr7.857 | 7 | 6 | 7 | 0 | 835 | 94652.31 | 8.39 | 42.31 | 97.78 | -0.216 |
| TNL-FII.chr7.921 | 4 | 3 | 4 | 0 | 705 | 80758.91 | 8.41 | 43 | 101.8 | -0.148 |
| TNL-FII.chr7.923 | 9 | 8 | 7 | 4 | 1196 | 134508.62 | 6.74 | 50.69 | 94.48 | -0.17 |
| TNL-FII.chr7.936 | 6 | 5 | 6 | 0 | 1209 | 137316.19 | 6.08 | 43.21 | 96.77 | -0.158 |
| TNL-FMA.chr0.553 | 7 | 6 | 7 | 0 | 871 | 98961.73 | 8.16 | 42.64 | 100.38 | -0.135 |
| IDSequence | Num. of Exon | Num. of Intron | Num. of CDS | Num. of UTR | Num. of Amino Acid | Molecular Weight | Theoretical pI | Instability Index | Aliphatic Index | Grand Average of Hydropathicity |
| TNL-FMA.chr0.554 | 4 | 3 | 4 | 0 | 815 | 92238.11 | 6.68 | 42.1 | 101.17 | -0.182 |
| TNL-FMA.chr0.565 | 11 | 10 | 11 | 0 | 1916 | 218218.67 | 7.83 | 44.56 | 101.56 | -0.155 |
| TNL-FMA.chr1.1780 | 5 | 4 | 5 | 0 | 1148 | 130790.29 | 6 | 38.69 | 94.68 | -0.193 |
| TNL-FMA.chr2.1405 | 6 | 5 | 6 | 2 | 1202 | 136273.15 | 6.94 | 46.73 | 98.92 | -0.159 |
| TNL-FMA.chr2.2218 | 7 | 6 | 7 | 0 | 1194 | 135942.46 | 6.18 | 48.91 | 92.87 | -0.325 |
| TNL-FMA.chr2.2280 | 6 | 5 | 6 | 0 | 1354 | 152670.49 | 6.62 | 53.48 | 97.61 | -0.255 |
| TNL-FMA.chr2.2281 | 8 | 7 | 6 | 4 | 1215 | 137289.99 | 5.3 | 47.17 | 96.03 | -0.169 |
| TNL-FMA.chr2.857 | 7 | 6 | 6 | 2 | 1118 | 127515.76 | 5.92 | 46.81 | 92.85 | -0.268 |
| TNL-FMA.chr3.3484 | 3 | 2 | 3 | 1 | 1124 | 127344.53 | 6.41 | 43.53 | 95.65 | -0.167 |
| TNL-FMA.chr4.2945 | 4 | 3 | 4 | 1 | 828 | 93048.57 | 8.41 | 41.51 | 103.35 | -0.163 |
| TNL-FMA.chr4.2946 | 5 | 4 | 5 | 1 | 1403 | 157630.94 | 6.04 | 43.84 | 102.74 | -0.157 |
| TNL-FMA.chr4.57 | 2 | 1 | 2 | 2 | 180 | 20594.32 | 7.01 | 29.87 | 79.11 | -0.431 |
| TNL-FMA.chr5.2425 | 6 | 5 | 5 | 3 | 1222 | 138858.28 | 7.95 | 49.65 | 97.56 | -0.16 |
| TNL-FMA.chr5.2877 | 5 | 4 | 5 | 0 | 1164 | 132251.88 | 5.87 | 45.89 | 93.18 | -0.206 |
| TNL-FMA.chr5.364 | 4 | 3 | 4 | 0 | 1077 | 123393.71 | 7.08 | 44.65 | 95.99 | -0.179 |
| TNL-FNG.chr1.1006 | 5 | 4 | 5 | 0 | 1208 | 137599.04 | 6.47 | 41.75 | 93.9 | -0.135 |
| TNL-FNG.chr1.1446 | 4 | 3 | 4 | 0 | 1043 | 118799.06 | 5.98 | 53.98 | 94.53 | -0.253 |
| TNL-FNG.chr2.105 | 8 | 7 | 8 | 0 | 1602 | 181933.55 | 6.24 | 49.68 | 95.28 | -0.165 |
| TNL-FNG.chr2.1702 | 5 | 4 | 5 | 0 | 1132 | 128786.6 | 6.27 | 37.95 | 93.32 | -0.238 |
| TNL-FNG.chr2.1729 | 5 | 4 | 5 | 2 | 1148 | 128839.67 | 6.76 | 39.97 | 96.69 | -0.13 |
| TNL-FNG.chr2.2347 | 6 | 5 | 6 | 0 | 970 | 110180.55 | 6.38 | 42.5 | 96.26 | -0.261 |
| TNL-FNG.chr2.2392 | 14 | 13 | 13 | 2 | 2189 | 248382.82 | 6.2 | 51.81 | 97.78 | -0.228 |
| TNL-FNG.chr3.2334 | 3 | 2 | 3 | 2 | 1362 | 155357.9 | 6.94 | 42.79 | 97.33 | -0.189 |
| TNL-FNG.chr3.2352 | 6 | 5 | 6 | 2 | 1145 | 128888.8 | 5.8 | 44.46 | 96.16 | -0.095 |
| TNL-FNG.chr3.3168 | 2 | 1 | 2 | 0 | 522 | 59506.67 | 6.28 | 43.64 | 92.28 | -0.298 |
| TNL-FNG.chr3.3322 | 5 | 4 | 4 | 3 | 1635 | 182888.82 | 5.22 | 43.12 | 87.7 | -0.326 |
| TNL-FNG.chr3.3728 | 6 | 5 | 6 | 2 | 1768 | 200414.21 | 6.77 | 40.16 | 102.26 | -0.138 |
| TNL-FNG.chr4.271 | 3 | 2 | 3 | 0 | 224 | 25478.87 | 5.48 | 49.28 | 85.67 | -0.273 |
| TNL-FNG.chr4.3018 | 5 | 4 | 5 | 1 | 1431 | 160491.31 | 6.09 | 45.71 | 103.25 | -0.12 |
| TNL-FNG.chr5.112 | 20 | 19 | 18 | 3 | 2530 | 284737.78 | 6.09 | 38.98 | 88.65 | -0.268 |
| TNL-FNG.chr5.2639 | 7 | 6 | 5 | 4 | 1222 | 138648.08 | 7.94 | 48.97 | 97.32 | -0.159 |
| TNL-FNG.chr5.3204 | 8 | 7 | 8 | 0 | 708 | 78974.99 | 8.26 | 44.57 | 99.84 | -0.133 |
| TNL-FNG.chr6.2320 | 5 | 4 | 5 | 0 | 582 | 66401.25 | 8.48 | 40.43 | 92.94 | -0.288 |
| TNL-FNG.chr6.244 | 10 | 9 | 10 | 0 | 1416 | 160291.3 | 6.83 | 49.08 | 93.91 | -0.221 |
| TNL-FNG.chr7.2518 | 4 | 3 | 4 | 0 | 1676 | 186915.04 | 6.65 | 43.19 | 96.36 | -0.057 |
| TNL-FNG.chr7.267 | 4 | 3 | 4 | 0 | 590 | 67281.2 | 6.4 | 37.3 | 97.92 | -0.188 |
| TNL-FNG.chr7.552 | 5 | 4 | 5 | 2 | 1166 | 131301.02 | 7.18 | 49.51 | 95.32 | -0.165 |
| TNL-FNG.chr7.927 | 6 | 5 | 6 | 2 | 1801 | 204647.81 | 6.46 | 40.69 | 100.14 | -0.161 |
| TNL-FNU.ctg101.157 | 8 | 7 | 8 | 2 | 1649 | 187110.55 | 6.59 | 50.01 | 95.52 | -0.181 |
| TNL-FNU.ctg101.35 | 6 | 5 | 6 | 0 | 939 | 105914.07 | 6.79 | 37.14 | 102.63 | -0.142 |
| TNL-FNU.ctg105.95 | 4 | 3 | 4 | 2 | 1113 | 126662.93 | 6.18 | 41.37 | 92.96 | -0.232 |
| TNL-FNU.ctg109.139 | 4 | 3 | 4 | 0 | 867 | 98605.2 | 6.84 | 46 | 103.63 | -0.113 |
| IDSequence | Num. of Exon | Num. of Intron | Num. of CDS | Num. of UTR | Num. of Amino Acid | Molecular Weight | Theoretical pI | Instability Index | Aliphatic Index | Grand Average of Hydropathicity |
| TNL-FNU.ctg114.235 | 6 | 5 | 5 | 3 | 1091 | 124324.86 | 6.9 | 44.26 | 96.31 | -0.271 |
| TNL-FNU.ctg129.394 | 6 | 5 | 6 | 1 | 1222 | 138513.48 | 5.84 | 40.57 | 97.94 | -0.215 |
| TNL-FNU.ctg132.96 | 3 | 2 | 3 | 1 | 1105 | 125921.98 | 5.42 | 40.48 | 96.24 | -0.148 |
| TNL-FNU.ctg134.120 | 7 | 6 | 7 | 1 | 913 | 103871.92 | 8.95 | 53.9 | 96.16 | -0.283 |
| TNL-FNU.ctg134.16 | 9 | 8 | 9 | 2 | 1462 | 165499.14 | 5.81 | 45.08 | 79.04 | -0.394 |
| TNL-FNU.ctg134.31 | 6 | 5 | 6 | 2 | 1567 | 177762.84 | 7.24 | 40.5 | 99.79 | -0.183 |
| TNL-FNU.ctg135.24 | 7 | 6 | 7 | 2 | 1330 | 150900.44 | 6.41 | 43.2 | 89.29 | -0.305 |
| TNL-FNU.ctg147.20.1 | 3 | 2 | 2 | 3 | 1055 | 119823.25 | 5.36 | 37.65 | 96.99 | -0.173 |
| TNL-FNU.ctg160.481 | 5 | 4 | 5 | 2 | 1090 | 123086.57 | 8.77 | 42.51 | 100.39 | -0.106 |
| TNL-FNU.ctg160.483 | 5 | 4 | 5 | 0 | 1172 | 131783.39 | 6.8 | 45.82 | 95.63 | -0.178 |
| TNL-FNU.ctg162.30 | 3 | 2 | 3 | 0 | 1354 | 154353.8 | 6.21 | 51.15 | 95.72 | -0.256 |
| TNL-FNU.ctg172.194 | 6 | 5 | 6 | 1 | 1000 | 114020.55 | 5.97 | 52.81 | 96.37 | -0.263 |
| TNL-FNU.ctg172.195 | 12 | 11 | 9 | 5 | 2296 | 260530 | 6.25 | 44.89 | 96.74 | -0.189 |
| TNL-FNU.ctg172.196 | 9 | 8 | 9 | 2 | 2293 | 261484.62 | 6.07 | 45.81 | 96.55 | -0.176 |
| TNL-FNU.ctg173.410 | 7 | 6 | 6 | 2 | 1003 | 113335.05 | 8.11 | 45.23 | 97.55 | -0.178 |
| TNL-FNU.ctg3.21 | 5 | 4 | 5 | 0 | 796 | 90837.49 | 6.51 | 45.34 | 95.46 | -0.233 |
| TNL-FNU.ctg3.22 | 6 | 5 | 6 | 0 | 1046 | 119145.99 | 5.8 | 46.68 | 94.37 | -0.197 |
| TNL-FNU.ctg3.25 | 5 | 4 | 5 | 0 | 1072 | 122415 | 6.81 | 48.27 | 93.62 | -0.21 |
| TNL-FNU.ctg3.73 | 6 | 5 | 3 | 5 | 1241 | 141402.43 | 5.95 | 49.79 | 91.96 | -0.368 |
| TNL-FNU.ctg3.75 | 5 | 4 | 5 | 1 | 1328 | 151292.6 | 5.67 | 40.94 | 93.73 | -0.272 |
| TNL-FNU.ctg3.77 | 6 | 5 | 6 | 1 | 2201 | 250156.34 | 5.22 | 43.01 | 100.2 | -0.105 |
| TNL-FNU.ctg32.58 | 5 | 4 | 4 | 3 | 1099 | 124394.83 | 5.68 | 44.8 | 98.18 | -0.123 |
| TNL-FNU.ctg56.77 | 9 | 8 | 9 | 1 | 1325 | 150745.2 | 5.66 | 46.92 | 96.42 | -0.172 |
| TNL-FNU.ctg8.247 | 4 | 3 | 4 | 0 | 1111 | 126678.26 | 6.78 | 43.66 | 94.83 | -0.184 |
| TNL-FNU.ctg81.211 | 5 | 4 | 5 | 0 | 1419 | 159319.89 | 6.84 | 45.63 | 103.23 | -0.156 |
| TNL-FNU.ctg81.212 | 5 | 4 | 5 | 0 | 1395 | 156499.67 | 6.58 | 43.31 | 101.87 | -0.165 |
| TNL-FNU.ctg81.216 | 5 | 4 | 5 | 0 | 1304 | 146208.43 | 6.41 | 42.72 | 105.91 | -0.092 |
| TNL-FPE.chr0.1078 | 8 | 7 | 8 | 2 | 1648 | 187099.31 | 6.48 | 49.47 | 94.45 | -0.19 |
| TNL-FPE.chr1.1274 | 5 | 4 | 2 | 5 | 1036 | 118408.95 | 6.09 | 37.15 | 100.44 | -0.166 |
| TNL-FPE.chr2.3663 | 7 | 6 | 7 | 0 | 990 | 112590.47 | 7.55 | 45.42 | 102.96 | -0.156 |
| TNL-FPE.chr2.91 | 5 | 4 | 5 | 0 | 1190 | 134764.92 | 6.51 | 41.99 | 96.32 | -0.203 |
| TNL-FPE.chr2.959 | 6 | 5 | 6 | 2 | 1189 | 134404.82 | 7.36 | 43.44 | 98.43 | -0.203 |
| TNL-FPE.chr3.3124 | 6 | 5 | 6 | 0 | 1084 | 123716.24 | 7.68 | 46.6 | 97.2 | -0.156 |
| TNL-FPE.chr3.552 | 5 | 4 | 5 | 0 | 1104 | 125121.28 | 8.2 | 40.46 | 100.88 | -0.062 |
| TNL-FPE.chr5.1409 | 6 | 5 | 6 | 0 | 714 | 81171.86 | 5.9 | 45.88 | 95.01 | -0.312 |
| TNL-FPE.chr5.1953 | 4 | 3 | 4 | 0 | 361 | 40555 | 5.77 | 55.44 | 88.09 | -0.216 |
| TNL-FPE.chr5.2900 | 4 | 3 | 4 | 1 | 1134 | 128004.42 | 5.84 | 41.41 | 102.47 | -0.083 |
| TNL-FPE.chr6.1904 | 5 | 4 | 5 | 1 | 1341 | 149634.2 | 5.23 | 37.4 | 106.37 | -0.049 |
| TNL-FPE.chr6.5657 | 5 | 4 | 4 | 3 | 1205 | 136841.98 | 6.34 | 39.87 | 95.41 | -0.221 |
| TNL-FPE.chr7.2368 | 8 | 7 | 7 | 3 | 1331 | 151411.55 | 6.89 | 47.82 | 90.36 | -0.29 |
| TNL-FvH4.1g01230.1 | 4 | 3 | 3 | 2 | 1087 | 123568.3 | 6.11 | 48.29 | 96.63 | -0.228 |
| TNL-FvH4.1g07020.1 | 5 | 4 | 5 | 2 | 1147 | 129918.85 | 8 | 44.27 | 96.87 | -0.196 |
| IDSequence | Num. of Exon | Num. of Intron | Num. of CDS | Num. of UTR | Num. of Amino Acid | Molecular Weight | Theoretical pI | Instability Index | Aliphatic Index | Grand Average of Hydropathicity |
| TNL-FvH4.1g11570.1 | 6 | 5 | 6 | 2 | 1203 | 136456.39 | 6.73 | 42.66 | 94.07 | -0.177 |
| TNL-FvH4.1g11580.1 | 5 | 4 | 5 | 0 | 1207 | 137862.44 | 6.8 | 42.93 | 94.63 | -0.154 |
| TNL-FvH4.1g15650.1 | 6 | 5 | 6 | 0 | 1225 | 139510.53 | 6.05 | 39.64 | 93.5 | -0.174 |
| TNL-FvH4.1g16600.1 | 10 | 9 | 10 | 0 | 1143 | 129315.47 | 6.18 | 43.98 | 99.57 | -0.129 |
| TNL-FvH4.1g16610.1 | 6 | 5 | 6 | 0 | 954 | 108750.18 | 5.65 | 56.33 | 94.91 | -0.277 |
| TNL-FvH4.1g16620.1 | 9 | 8 | 9 | 2 | 2295 | 260437 | 6.31 | 44.44 | 97.76 | -0.176 |
| TNL-FvH4.1g16640.1 | 15 | 14 | 9 | 8 | 2278 | 258868.75 | 6.25 | 45.76 | 94.03 | -0.205 |
| TNL-FvH4.1g16650.1 | 8 | 7 | 8 | 0 | 1868 | 211745.47 | 6.1 | 46.98 | 96.35 | -0.16 |
| TNL-FvH4.1g17740.1 | 14 | 13 | 6 | 9 | 1196 | 135607.97 | 5.9 | 38.46 | 93.74 | -0.224 |
| TNL-FvH4.1g22370.1 | 9 | 8 | 9 | 0 | 875 | 99455.94 | 6.6 | 42.16 | 97.81 | -0.191 |
| TNL-FvH4.1g22380.1 | 7 | 6 | 7 | 0 | 999 | 112826.62 | 6.33 | 41.61 | 99.7 | -0.206 |
| TNL-FvH4.1g22390.1 | 5 | 4 | 5 | 0 | 1067 | 122140.06 | 6.26 | 45.86 | 100.82 | -0.105 |
| TNL-FvH4.1g22540.1 | 8 | 7 | 8 | 0 | 1126 | 128934.77 | 7.36 | 48.84 | 102.01 | -0.141 |
| TNL-FvH4.2g00540.1 | 9 | 8 | 5 | 6 | 1129 | 128234.37 | 6.2 | 45.34 | 98.92 | -0.185 |
| TNL-FvH4.2g00550.1 | 6 | 5 | 6 | 2 | 1304 | 148179.4 | 5.89 | 48.05 | 94.85 | -0.145 |
| TNL-FvH4.2g02050.1 | 9 | 8 | 8 | 3 | 1648 | 187021.22 | 6.39 | 49.49 | 95.16 | -0.177 |
| TNL-FvH4.2g14040.1 | 7 | 6 | 7 | 2 | 949 | 108628.05 | 6.24 | 45.71 | 92.28 | -0.191 |
| TNL-FvH4.2g14320.1 | 14 | 13 | 13 | 2 | 2057 | 234220.23 | 6.44 | 47.16 | 100.26 | -0.172 |
| TNL-FvH4.2g17330.1 | 6 | 5 | 6 | 1 | 1202 | 136273.15 | 6.94 | 46.73 | 98.92 | -0.159 |
| TNL-FvH4.2g20310.1 | 5 | 4 | 5 | 2 | 1206 | 135917.77 | 7.04 | 40.71 | 95.84 | -0.137 |
| TNL-FvH4.2g27070.1 | 6 | 5 | 5 | 3 | 1173 | 133783.57 | 6.91 | 46.99 | 94.38 | -0.296 |
| TNL-FvH4.2g27370.1 | 6 | 5 | 6 | 1 | 1112 | 127022.15 | 7.91 | 47.91 | 92.32 | -0.327 |
| TNL-FvH4.2g27720.1 | 17 | 16 | 7 | 12 | 1250 | 141306.82 | 5.48 | 45.41 | 97.08 | -0.173 |
| TNL-FvH4.2g38620.1 | 7 | 6 | 6 | 3 | 1769 | 200592.68 | 6.53 | 38.52 | 103.45 | -0.128 |
| TNL-FvH4.3g05740.1 | 7 | 6 | 5 | 4 | 1060 | 120422.43 | 5.68 | 46.58 | 95.6 | -0.203 |
| TNL-FvH4.3g10020.1 | 6 | 5 | 6 | 1 | 1367 | 153536.32 | 6.06 | 36.39 | 106.9 | -0.055 |
| TNL-FvH4.3g15720.1 | 5 | 4 | 5 | 0 | 1011 | 113106.33 | 6.06 | 48.63 | 101.27 | -0.16 |
| TNL-FvH4.3g33030.1 | 9 | 8 | 9 | 0 | 1362 | 154238.6 | 6.38 | 43.76 | 94.26 | -0.104 |
| TNL-FvH4.3g36130.1 | 9 | 8 | 9 | 0 | 865 | 98832.88 | 8.8 | 39.84 | 96.44 | -0.219 |
| TNL-FvH4.3g38670.1 | 3 | 2 | 3 | 0 | 1160 | 132059.32 | 6.51 | 47.67 | 97.04 | -0.171 |
| TNL-FvH4.3g42940.1 | 3 | 2 | 3 | 2 | 1131 | 128221.55 | 6.55 | 44.09 | 95.48 | -0.163 |
| TNL-FvH4.3g43390.1 | 4 | 3 | 3 | 3 | 1238 | 141795.16 | 7.08 | 42.58 | 95.92 | -0.203 |
| TNL-FvH4.3g43610.1 | 2 | 1 | 2 | 0 | 1092 | 125343.67 | 5.99 | 39.18 | 97.2 | -0.206 |
| TNL-FvH4.3g43630.1 | 9 | 8 | 6 | 4 | 2224 | 254157.72 | 5.77 | 42.87 | 99.62 | -0.153 |
| TNL-FvH4.3g43650.1 | 2 | 1 | 2 | 1 | 1081 | 123094.99 | 6.35 | 46.23 | 97.11 | -0.202 |
| TNL-FvH4.3g43660.1 | 13 | 12 | 13 | 0 | 2100 | 238612.16 | 6.19 | 46.48 | 98.18 | -0.185 |
| TNL-FvH4.3g43850.1 | 16 | 15 | 2 | 16 | 1130 | 129137.2 | 5.36 | 45.01 | 94.72 | -0.274 |
| TNL-FvH4.3g43860.1 | 2 | 1 | 2 | 0 | 1122 | 127716.05 | 5.77 | 48.04 | 96.17 | -0.182 |
| TNL-FvH4.3g43880.1 | 13 | 12 | 12 | 2 | 1733 | 196722.09 | 5.69 | 45.05 | 92.36 | -0.208 |
| TNL-FvH4.3g43890.1 | 14 | 13 | 2 | 14 | 1122 | 127398.81 | 5.59 | 44.89 | 96.69 | -0.165 |
| TNL-FvH4.3g43940.1 | 2 | 1 | 2 | 1 | 1128 | 128447.32 | 6.44 | 49.25 | 96.84 | -0.198 |
| TNL-FvH4.3g43950.1 | 11 | 10 | 3 | 10 | 1131 | 129116.87 | 5.4 | 39.78 | 99.12 | -0.149 |
| IDSequence | Num. of Exon | Num. of Intron | Num. of CDS | Num. of UTR | Num. of Amino Acid | Molecular Weight | Theoretical pI | Instability Index | Aliphatic Index | Grand Average of Hydropathicity |
| TNL-FvH4.3g43960.1 | 5 | 4 | 5 | 0 | 1108 | 127167.78 | 6.17 | 39.01 | 96.94 | -0.225 |
| TNL-FvH4.3g44000.1 | 13 | 12 | 13 | 1 | 2525 | 287861.47 | 5.66 | 46.88 | 95.35 | -0.198 |
| TNL-FvH4.3g44030.1 | 5 | 4 | 4 | 3 | 1223 | 138495.67 | 5.94 | 45.03 | 98.59 | -0.232 |
| TNL-FvH4.3g44050.1 | 15 | 14 | 12 | 5 | 1849 | 209155.31 | 5.33 | 42.5 | 97.08 | -0.147 |
| TNL-FvH4.3g44070.1 | 5 | 4 | 5 | 0 | 1334 | 152011.74 | 5.61 | 41.93 | 94.63 | -0.247 |
| TNL-FvH4.3g44370.1 | 8 | 7 | 6 | 4 | 1046 | 119204.9 | 5.75 | 45.51 | 93.89 | -0.222 |
| TNL-FvH4.3g44390.1 | 14 | 13 | 13 | 2 | 1915 | 218727.42 | 7.71 | 44.44 | 95.35 | -0.21 |
| TNL-FvH4.3g44570.1 | 3 | 2 | 3 | 0 | 1125 | 127643.35 | 5.99 | 42.05 | 98.08 | -0.139 |
| TNL-FvH4.3g44580.1 | 7 | 6 | 7 | 2 | 1078 | 122757.46 | 8.81 | 41.38 | 93.36 | -0.275 |
| TNL-FvH4.3g44610.1 | 6 | 5 | 4 | 4 | 1074 | 121722.91 | 6.35 | 36.75 | 95.13 | -0.223 |
| TNL-FvH4.3g44670.1 | 3 | 2 | 3 | 2 | 1132 | 128826.06 | 6.53 | 48.06 | 97.47 | -0.219 |
| TNL-FvH4.3g44680.1 | 12 | 11 | 5 | 9 | 1178 | 133396.55 | 5.88 | 41.42 | 97.22 | -0.188 |
| TNL-FvH4.3g45170.1 | 2 | 1 | 2 | 0 | 1067 | 121641.47 | 5.87 | 49.3 | 98.91 | -0.216 |
| TNL-FvH4.3g45380.1 | 7 | 6 | 7 | 0 | 925 | 105123.85 | 8.22 | 51.46 | 94.99 | -0.179 |
| TNL-FvH4.3g45680.1 | 4 | 3 | 3 | 3 | 1110 | 125878.23 | 5.95 | 43.39 | 98.78 | -0.188 |
| TNL-FvH4.3g45690.1 | 6 | 5 | 5 | 3 | 1151 | 130483.05 | 7.23 | 37.5 | 98.64 | -0.16 |
| TNL-FvH4.3g45700.1 | 2 | 1 | 2 | 1 | 1106 | 126010.4 | 6.9 | 40.95 | 95.01 | -0.252 |
| TNL-FvH4.3g45730.1 | 5 | 4 | 3 | 4 | 1118 | 127453.9 | 6.29 | 44.07 | 95.89 | -0.247 |
| TNL-FvH4.4g00790.1 | 3 | 2 | 3 | 0 | 247 | 27868.32 | 5.52 | 50.59 | 80.93 | -0.323 |
| TNL-FvH4.4g13420.1 | 6 | 5 | 5 | 3 | 1354 | 151476.46 | 6.64 | 41.73 | 105.95 | -0.036 |
| TNL-FvH4.4g35420.1 | 6 | 5 | 5 | 3 | 1403 | 157523.8 | 6.38 | 44.74 | 100.94 | -0.187 |
| TNL-FvH4.5g01270.1 | 46 | 45 | 46 | 1 | 4812 | 540892.64 | 6.37 | 37.38 | 87.56 | -0.303 |
| TNL-FvH4.5g02970.1 | 8 | 7 | 8 | 1 | 1682 | 190780.36 | 5.15 | 45.29 | 90.87 | -0.336 |
| TNL-FvH4.5g03630.1 | 5 | 4 | 4 | 3 | 838 | 95361.38 | 7.02 | 40.66 | 96.04 | -0.188 |
| TNL-FvH4.5g07310.1 | 5 | 4 | 5 | 2 | 1137 | 130894.85 | 6.04 | 40.9 | 95.8 | -0.213 |
| TNL-FvH4.5g16070.1 | 3 | 2 | 2 | 3 | 1065 | 121348.75 | 5.33 | 39.74 | 94.42 | -0.235 |
| TNL-FvH4.5g27640.1 | 4 | 3 | 3 | 3 | 1164 | 131927.19 | 6.1 | 42.03 | 100.83 | -0.093 |
| TNL-FvH4.5g32050.1 | 5 | 4 | 5 | 0 | 1207 | 137420.26 | 5.74 | 45.69 | 95.34 | -0.227 |
| TNL-FvH4.5g32970.1 | 5 | 4 | 5 | 1 | 1186 | 133889.86 | 8.6 | 43.67 | 99.25 | -0.12 |
| TNL-FvH4.5g34210.1 | 6 | 5 | 6 | 0 | 1034 | 117236.22 | 7.56 | 45.95 | 93.11 | -0.239 |
| TNL-FvH4.5g35770.1 | 6 | 5 | 5 | 3 | 1222 | 138704.07 | 7.95 | 49.46 | 97.48 | -0.161 |
| TNL-FvH4.5g38540.1 | 8 | 7 | 7 | 3 | 1502 | 170360.01 | 6.06 | 47.22 | 93.15 | -0.188 |
| TNL-FvH4.5g38600.1 | 10 | 9 | 9 | 3 | 1545 | 175233 | 5.39 | 46.47 | 96.87 | -0.128 |
| TNL-FvH4.5g38680.1 | 8 | 7 | 8 | 2 | 1679 | 191248.73 | 6.48 | 43.27 | 97.53 | -0.107 |
| TNL-FvH4.5g38870.1 | 6 | 5 | 6 | 1 | 1284 | 145083.06 | 7.21 | 42.23 | 96.48 | -0.236 |
| TNL-FvH4.6g01690.1 | 9 | 8 | 9 | 2 | 1475 | 167120.93 | 6.1 | 45.5 | 83.39 | -0.325 |
| TNL-FvH4.6g03250.1 | 7 | 6 | 7 | 1 | 1329 | 151048.61 | 7.05 | 46.87 | 93.36 | -0.268 |
| TNL-FvH4.6g04250.1 | 7 | 6 | 6 | 3 | 1142 | 130016.23 | 6.52 | 47.59 | 93.44 | -0.175 |
| TNL-FvH4.6g04730.1 | 5 | 4 | 4 | 2 | 1125 | 128097.05 | 6.58 | 46.6 | 91.31 | -0.23 |
| TNL-FvH4.6g05610.1 | 6 | 5 | 6 | 1 | 1207 | 137428.51 | 7.01 | 48.63 | 93.75 | -0.126 |
| TNL-FvH4.6g34410.1 | 8 | 7 | 5 | 5 | 1285 | 146656 | 6.12 | 46.79 | 97.19 | -0.202 |
| TNL-FvH4.7g04050.1 | 6 | 5 | 6 | 0 | 1050 | 118620.33 | 7.61 | 40.55 | 97.33 | -0.084 |
| IDSequence | Num. of Exon | Num. of Intron | Num. of CDS | Num. of UTR | Num. of Amino Acid | Molecular Weight | Theoretical pI | Instability Index | Aliphatic Index | Grand Average of Hydropathicity |
| TNL-FvH4.7g06680.1 | 9 | 8 | 4 | 7 | 1160 | 132974.63 | 6.12 | 38.41 | 97.94 | -0.196 |
| TNL-FvH4.7g09800.1 | 7 | 6 | 7 | 2 | 1235 | 139547.18 | 6.28 | 37.67 | 91.75 | -0.287 |
| TNL-FvH4.7g10520.1 | 8 | 7 | 8 | 2 | 1217 | 137113.24 | 6.85 | 50.79 | 91.97 | -0.221 |
| TNL-FvH4.7g10750.1 | 10 | 9 | 10 | 1 | 1299 | 148043.54 | 7.7 | 44.97 | 94.8 | -0.228 |
| TNL-FvH4.7g11550.1 | 13 | 12 | 13 | 1 | 1593 | 181446.9 | 5.99 | 44.8 | 79.82 | -0.426 |
| TNL-FvH4.7g12160.1 | 6 | 5 | 6 | 2 | 1214 | 137282.77 | 6.2 | 40.84 | 96.34 | -0.1 |
| TNL-FvH4.7g13090.1 | 11 | 10 | 11 | 2 | 1309 | 148041.96 | 6.68 | 50.98 | 97.51 | -0.132 |
| TNL-FvH4.7g13640.1 | 13 | 12 | 3 | 12 | 1105 | 125770.86 | 5.44 | 39.84 | 95.35 | -0.141 |
| TNL-FvH4.7g17700.1 | 5 | 4 | 5 | 2 | 1236 | 140643.36 | 7.06 | 39.27 | 96.84 | -0.169 |
| TNL-FvH4.7g21060.1 | 5 | 4 | 5 | 0 | 1136 | 128486.5 | 5.65 | 37.44 | 95.55 | -0.167 |
| TNL-FvH4.7g21070.1 | 7 | 6 | 5 | 4 | 1145 | 130542.39 | 5.31 | 43.5 | 93.28 | -0.188 |
| TNL-FvH4.7g21180.1 | 14 | 13 | 12 | 3 | 1871 | 212019.45 | 5.88 | 42.38 | 96.51 | -0.087 |
| TNL-FvH4.7g21230.1 | 5 | 4 | 5 | 0 | 1152 | 131731.08 | 5.72 | 42.32 | 94.71 | -0.212 |
| TNL-FvH4.7g28510.1 | 7 | 6 | 7 | 1 | 1964 | 219237.51 | 6.46 | 43.68 | 92.96 | -0.154 |
| TNL-FvH4.7g28540.1 | 7 | 6 | 5 | 4 | 1327 | 150651.47 | 6.11 | 46.58 | 96.16 | -0.181 |
| TNL-FvH4.7g28550.1 | 9 | 8 | 9 | 1 | 1368 | 155360.28 | 6.62 | 46.62 | 91.62 | -0.245 |
| TNL-FvH4.7g31110.1 | 2 | 1 | 2 | 2 | 1102 | 125689.83 | 7 | 44.51 | 98.16 | -0.124 |
| TNL-FvH4.7g31510.1 | 10 | 9 | 5 | 6 | 2240 | 254115.51 | 5.86 | 46.26 | 98.36 | -0.182 |
| TNL-FvH4.7g32440.1 | 6 | 5 | 6 | 0 | 912 | 104156.69 | 6.07 | 37.93 | 95.01 | -0.205 |
| TNL-FvH4.7g32460.1 | 5 | 4 | 5 | 0 | 1098 | 125272.88 | 5.69 | 46.42 | 95.94 | -0.179 |
| TNL-FvH4.7g32470.1 | 10 | 9 | 9 | 2 | 1362 | 155448.86 | 5.44 | 47.02 | 98.41 | -0.16 |
| TNL-FvH4.7g32480.1 | 11 | 10 | 7 | 6 | 1018 | 115614.54 | 5.99 | 42.75 | 98.43 | -0.188 |
| TNL-FvH4.7g32740.1 | 6 | 5 | 5 | 3 | 1114 | 127240.14 | 5.92 | 41.6 | 96.25 | -0.164 |
| TNL-FvH4.7g32760.1 | 6 | 5 | 6 | 2 | 1065 | 120522.49 | 5.62 | 41.15 | 100.39 | -0.129 |
| TNL-FvH4.7g33140.1 | 5 | 4 | 5 | 2 | 1082 | 123817.33 | 6.42 | 44.4 | 94.85 | -0.232 |
| TNL-FvH4.7g33190.1 | 4 | 3 | 4 | 0 | 786 | 90357.83 | 6.34 | 42.89 | 97.94 | -0.217 |
| TNL-FVI.CHR1.1015 | 5 | 4 | 5 | 2 | 1244 | 140880.35 | 6.23 | 40.66 | 92.37 | -0.166 |
| TNL-FVI.CHR1.1444 | 10 | 9 | 10 | 2 | 2256 | 257728.31 | 6.13 | 47.83 | 95.12 | -0.19 |
| TNL-FVI.CHR1.592 | 6 | 5 | 5 | 3 | 1159 | 131021.59 | 6.61 | 41.24 | 96.79 | -0.173 |
| TNL-FVI.CHR2.1052 | 7 | 6 | 5 | 3 | 1084 | 123893.26 | 6.34 | 47.8 | 99.59 | -0.184 |
| TNL-FVI.CHR2.166 | 8 | 7 | 8 | 2 | 1649 | 187255.58 | 6.52 | 49.77 | 94.69 | -0.184 |
| TNL-FVI.CHR2.2260 | 6 | 5 | 6 | 2 | 1197 | 135589.46 | 8.06 | 44.72 | 97.68 | -0.187 |
| TNL-FVI.CHR2.2512 | 5 | 4 | 5 | 1 | 934 | 105552.21 | 8.55 | 40.79 | 97.34 | -0.162 |
| TNL-FVI.CHR2.3162 | 9 | 8 | 6 | 5 | 1190 | 135037.72 | 5.64 | 48.35 | 95.34 | -0.221 |
| TNL-FVI.CHR2.4091 | 7 | 6 | 6 | 3 | 1769 | 200631.6 | 6.4 | 40.11 | 103.29 | -0.133 |
| TNL-FVI.CHR3.118 | 5 | 4 | 4 | 2 | 1072 | 118415.92 | 6.25 | 48.05 | 104.5 | -0.077 |
| TNL-FVI.CHR3.2623 | 7 | 6 | 5 | 4 | 1100 | 124855.11 | 5.94 | 47.34 | 92.4 | -0.221 |
| TNL-FVI.CHR4.3081 | 6 | 5 | 5 | 3 | 1385 | 155183.58 | 6.26 | 45.05 | 105.76 | -0.074 |
| TNL-FVI.CHR4.3082 | 7 | 6 | 7 | 0 | 1293 | 144930.13 | 6.38 | 42.44 | 101.99 | -0.167 |
| TNL-FVI.CHR4.3083 | 5 | 4 | 5 | 2 | 1432 | 160560.35 | 6.33 | 42.81 | 103.72 | -0.12 |
| TNL-FVI.CHR5.2738 | 8 | 7 | 8 | 0 | 788 | 89587.62 | 5.84 | 47.82 | 97.34 | -0.273 |
| TNL-FVI.CHR5.3055 | 6 | 5 | 5 | 3 | 1222 | 138750.15 | 8.12 | 49.58 | 97.41 | -0.163 |
| IDSequence | Num. of Exon | Num. of Intron | Num. of CDS | Num. of UTR | Num. of Amino Acid | Molecular Weight | Theoretical pI | Instability Index | Aliphatic Index | Grand Average of Hydropathicity |
| TNL-FVI.CHR5.3285 | 8 | 7 | 8 | 2 | 1676 | 190695.91 | 7.03 | 45.18 | 94.69 | -0.117 |
| TNL-FVI.CHR5.808 | 5 | 4 | 5 | 1 | 746 | 85899.88 | 8.77 | 53.4 | 94.42 | -0.285 |
| TNL-FVI.CHR6.256 | 6 | 5 | 5 | 3 | 1233 | 140458.6 | 7.94 | 48.05 | 93.45 | -0.279 |
| TNL-FVI.CHR7.1039 | 10 | 9 | 10 | 1 | 1584 | 179387.8 | 6.3 | 46.15 | 82.62 | -0.341 |
| TNL-FVI.CHR7.2164 | 8 | 7 | 8 | 2 | 1348 | 152721.05 | 6.16 | 43.43 | 91.19 | -0.27 |
| TNL-FVI.CHR7.2362 | 9 | 8 | 7 | 4 | 1332 | 151569.82 | 6.8 | 46.75 | 90.07 | -0.279 |
| TNL-FVI.CHR7.993 | 5 | 4 | 5 | 1 | 1059 | 120396.94 | 5.65 | 47.46 | 99.38 | -0.223 |

**Table S4a** List of NLR gene family paralogous gene pairs

| Segmental duplication gene pairs | | | | Tandem duplication gene pairs | | | |
| --- | --- | --- | --- | --- | --- | --- | --- |
| ID of sequence 1 | ID of sequence 2 | ID of sequence 1 ID of sequence 2 | | ID of sequence 1 ID of sequence 2 | | ID of sequence 1 | ID of sequence 2 |
| TNL-FvH4.7g32740.1 | TNL-FvH4.7g32460.1 | N-FII.chr7.209 | CNL-FII.chr7.210 | TNL-FDA.chr7.00190 | TN-FDA.chr7.00191 | TNL-FVI.CHR4.3082 | TNL-FVI.CHR4.3083 |
| TNL-FvH4.7g32440.1 | TNL-FvH4.7g32460.1 | N-FII.chr5.1933 | CNL-FII.chr5.2003 | TNL-FDA.chr7.00189 | TNL-FDA.chr7.00184 | TNL-FVI.CHR4.3081 | TNL-FVI.CHR4.3082 |
| TNL-FvH4.7g21180.1 | TNL-FvH4.7g21070.1 | N-FII.chr5.1933 | CNL-FII.chr5.3063 | TNL-FDA.chr7.00189 | TNL-FDA.chr7.00190 | TNL-FVI.CHR2.3162 | NL-FVI.CHR2.3163 |
| TNL-FvH4.6g04730.1 | TNL-FvH4.6g05610.1 | N-FDA.chr3.20836 | NL-FDA.chr3.20838 | TNL-FDA.chr7.00189 | TNL-FDA.chr7.00159 | TNL-FvH4.7g33190.1 | TNL-FvH4.7g32760.1 |
| TNL-FvH4.6g04250.1 | NL-FvH4.6g05490.1 | N-FDA.chr3.20836 | TN-FDA.chr3.05844 | TNL-FDA.chr7.00184 | TNL-FDA.chr7.00190 | TNL-FvH4.7g33190.1 | TNL-FvH4.7g33140.1 |
| TNL-FvH4.5g38870.1 | TNL-FvH4.1g15650.1 | N-FDA.chr3.20833 | N-FDA.chr3.20832 | TNL-FDA.chr7.00184 | TNL-FDA.chr7.00159 | TNL-FvH4.7g33190.1 | TNL-FvH4.7g32470.1 |
| TNL-FvH4.5g02970.1 | TNL-FvH4.5g01270.1 | N-FDA.chr3.20833 | NL-FDA.chr3.20838 | TNL-FDA.chr7.00114 | TNL-FDA.chr7.00190 | TNL-FvH4.7g33190.1 | TNL-FvH4.7g32740.1 |
| TNL-FvH4.3g44570.1 | TNL-FvH4.1g17740.1 | N-FDA.chr3.20832 | N-FDA.chr3.20836 | TNL-FDA.chr7.00114 | TNL-FDA.chr7.00159 | TNL-FvH4.7g33190.1 | TNL-FvH4.7g32460.1 |
| TNL-FvH4.3g44370.1 | TNL-FvH4.3g44390.1 | N-FDA.chr3.20832 | NL-FDA.chr3.20838 | TNL-FDA.chr7.00114 | TNL-FDA.chr7.00184 | TNL-FvH4.7g33140.1 | TNL-FvH4.7g32480.1 |
| TNL-FvH4.3g44070.1 | TNL-FvH4.3g44000.1 | N-FDA.chr3.20832 | TN-FDA.chr3.05844 | TNL-FDA.chr7.00114 | TNL-FDA.chr7.00189 | TNL-FvH4.7g33140.1 | TNL-FvH4.7g32740.1 |
| TNL-FvH4.3g43890.1 | TNL-FvH4.3g43850.1 | N-FDA.chr3.20742 | N-FDA.chr3.20833 | TNL-FDA.chr7.00110 | TNL-FDA.chr7.00189 | TNL-FvH4.7g33140.1 | TNL-FvH4.7g32760.1 |
| TNL-FvH4.3g43880.1 | TNL-FvH4.3g44000.1 | N-FDA.chr3.20742 | NL-FDA.chr3.20838 | TNL-FDA.chr7.00110 | TNL-FDA.chr7.00190 | TNL-FvH4.7g33140.1 | TNL-FvH4.7g32440.1 |
| TNL-FvH4.3g43860.1 | TNL-FvH4.3g43940.1 | N-FDA.chr3.20671 | TNL-FDA.chr3.20670 | TNL-FDA.chr7.00110 | TNL-FDA.chr7.00159 | TNL-FvH4.7g33140.1 | TNL-FvH4.7g32460.1 |
| TNL-FvH4.3g43610.1 | TNL-FvH4.3g43390.1 | N-FDA.chr3.20666 | NL-FDA.chr3.20676 | TNL-FDA.chr6.02361 | TNL-FDA.chr6.02345 | TNL-FvH4.7g32760.1 | TNL-FvH4.7g32740.1 |
| TNL-FvH4.3g10020.1 | TNL-FvH4.4g13420.1 | N-FDA.chr3.20666 | TNL-FDA.chr3.05837 | TNL-FDA.chr5.25282 | TNL-FDA.chr5.25283 | TNL-FvH4.7g32760.1 | TNL-FvH4.7g32480.1 |
| TNL-FvH4.2g00550.1 | TNL-FvH4.7g33190.1 | N-FDA.chr3.05878 | N-FDA.chr3.05877 | TNL-FDA.chr5.03488 | TNL-FDA.chr5.03487 | TNL-FvH4.7g32760.1 | TNL-FvH4.7g32460.1 |
| TNL-FvH4.1g22390.1 | TNL-FvH4.1g22540.1 | N-FDA.chr3.05878 | N-FDA.chr3.20666 | TNL-FDA.chr5.03487 | TN-FDA.chr5.03486 | TNL-FvH4.7g32760.1 | TNL-FvH4.7g32440.1 |
| TNL-FNU.ctg3.21 | TNL-FNU.ctg3.25 | N-FDA.chr3.05878 | NL-FDA.chr3.20676 | TNL-FDA.chr3.20747 | NL-FDA.chr3.20746 | TNL-FvH4.7g32480.1 | TNL-FvH4.7g32460.1 |
| TNL-FNU.ctg162.30 | NL-FNU.ctg163.12 | N-FDA.chr3.05878 | TNL-FDA.chr3.05837 | TNL-FDA.chr3.20725 | TN-FDA.chr3.20724 | TNL-FvH4.7g32470.1 | TNL-FvH4.7g32480.1 |
| TNL-FNG.chr4.271 | TN-FNG.chr4.253 | N-FDA.chr3.05877 | N-FDA.chr3.20666 | TNL-FDA.chr3.20678 | TNL-FDA.chr3.20677 | TNL-FvH4.7g32460.1 | TNL-FvH4.7g32470.1 |
| TNL-FNG.chr3.3322 | TNL-FNG.chr5.112 | N-FDA.chr3.05877 | NL-FDA.chr3.20676 | TNL-FDA.chr3.20670 | TNL-FDA.chr3.20669 | TNL-FvH4.7g28540.1 | TNL-FvH4.7g28550.1 |
| TNL-FMA.chr1.1780 | TNL-FMA.chr5.2877 | N-FDA.chr3.05877 | TNL-FDA.chr3.05837 | TNL-FDA.chr3.20670 | TNL-FDA.chr3.20678 | TNL-FvH4.7g28510.1 | TN-FvH4.7g28520.1 |
| TNL-FMA.chr0.554 | TNL-FMA.chr0.565 | N-FDA.chr2.12211 | CN-FDA.chr2.12213 | TNL-FDA.chr3.09523 | TNL-FDA.chr1.15663 | TNL-FvH4.7g21060.1 | TNL-FvH4.7g21070.1 |
| TNL-FII.chr3.744.2 | TNL-FII.chr3.3419 | N-FDA.chr2.12211 | CN-FDA.chr2.12217 | TNL-FDA.chr3.09523 | TNL-FDA.chr4.10670 | TNL-FvH4.6g04250.1 | TNL-FvH4.6g05610.1 |
| TNL-FII.chr3.3626 | TNL-FII.chr3.3603 | N-FDA.chr2.12211 | CNL-FDA.chr2.12209 | TNL-FDA.chr3.05839 | TNL-FDA.chr3.05838 | TNL-FvH4.6g04250.1 | TNL-FvH4.6g04730.1 |
| TNL-FII.chr3.1830 | TNL-FII.chr7.1056 | N-FDA.chr2.12211 | CNL-FDA.chr2.12218 | TNL-FDA.chr3.05837 | TNL-FDA.chr3.05839 | TNL-FvH4.6g04250.1 | NL-FvH4.6g04350.1 |
| TNL-FII.chr1.757 | TNL-FII.chr3.3419 | N-FDA.chr1.22180 | RNL-FDA.chr1.22179 | TNL-FDA.chr3.05837 | TNL-FDA.chr3.05838 | TNL-FvH4.4g35420.1 | NL-FvH4.4g35430.1 |
| TNL-FDA.chr6.02361 | NL-FDA.chr6.02338 | CNL-FVI.CHR7.3758 | CN-FVI.CHR7.3759 | TNL-FDA.chr2.20071 | TNL-FDA.chr2.20072 | TNL-FvH4.3g45730.1 | TNL-FvH4.3g45170.1 |
| TNL-FDA.chr3.09474 | TNL-FDA.chr5.23994 | CNL-FVI.CHR7.3757 | CNL-FVI.CHR7.3758 | TNL-FDA.chr1.17980 | TNL-FDA.chr1.17981 | TNL-FvH4.3g45730.1 | TNL-FvH4.3g45690.1 |
| TNL-FDA.chr3.09399 | TNL-FDA.chr1.15663 | CNL-FVI.CHR1.1874 | N-FVI.CHR1.1875 | TN-FvH4.7g32720.1 | TNL-FvH4.7g33140.1 | TNL-FvH4.3g45730.1 | TNL-FvH4.3g45700.1 |
| TNL-FDA.chr3.05639 | TNL-FDA.chr6.02439 | CNL-FvH4.7g28390 | CNL-FvH4.7g27330.1 | TN-FvH4.7g32720.1 | TNL-FvH4.7g32480.1 | TNL-FvH4.3g45730.1 | TNL-FvH4.1g17740.1 |
| TNL-FDA.chr1.17978 | TNL-FDA.chr1.17976 | CNL-FvH4.7g28390 | CNL-FvH4.7g28110.1 | TN-FvH4.7g32720.1 | TNL-FvH4.7g32760.1 | TNL-FvH4.3g45730.1 | TNL-FvH4.3g44570.1 |
| TNL-FDA.chr1.15663 | TNL-FDA.chr4.10670 | CNL-FvH4.7g27890 | CNL-FvH4.7g27330.1 | TN-FvH4.7g32720.1 | TNL-FvH4.7g32440.1 | TNL-FvH4.3g45700.1 | TNL-FvH4.1g17740.1 |
| TNL-FDA.chr1.07161 | TNL-FDA.chr1.04275 | CNL-FvH4.7g27890 | CNL-FvH4.7g28110.1 | TN-FvH4.7g32720.1 | TNL-FvH4.7g32460.1 | TNL-FvH4.3g45700.1 | TNL-FvH4.3g44570.1 |
| TN-FvH4.6g01300.1 | TN-FvH4.3g45630.1 | CNL-FvH4.7g15190 | N-FvH4.7g15200.1 | TN-FvH4.6g49490.1 | TN-FvH4.6g49500.1 | TNL-FvH4.3g45690.1 | TNL-FvH4.1g17740.1 |
| TN-FvH4.5g34240.1 | TNL-FvH4.3g45380.1 | CNL-FvH4.6g53580 | CNL-FvH4.3g05070.1 | TN-FvH4.6g01300.1 | TNL-FvH4.3g45700.1 | TNL-FvH4.3g45690.1 | TNL-FvH4.3g44570.1 |
| TN-FvH4.3g45630.1 | TNL-FvH4.3g44610.1 | CNL-FvH4.6g51610 | CN-FvH4.6g51570.1 | TN-FvH4.6g01300.1 | TNL-FvH4.3g44570.1 | TNL-FvH4.3g45690.1 | TNL-FvH4.3g44670.1 |
| TN-FvH4.3g09290.1 | TNL-FvH4.5g07310.1 | CNL-FvH4.6g51610 | CNL-FvH4.2g17640.1 | TN-FvH4.6g01300.1 | TNL-FvH4.3g45680.1 | TNL-FvH4.3g45690.1 | TNL-FvH4.3g45700.1 |
| TN-FvH4.3g09290.1 | TNL-FvH4.5g03630.1 | CNL-FvH4.6g51550 | CN-FvH4.6g51570.1 | TN-FvH4.6g01300.1 | TNL-FvH4.3g43950.1 | TNL-FvH4.3g45680.1 | TNL-FvH4.3g45700.1 |
| TN-FPE.chr5.245 | TNL-FPE.chr5.1409 | CNL-FvH4.6g51550 | CNL-FvH4.2g17640.1 | TN-FvH4.6g01300.1 | TNL-FvH4.3g43960.1 | TNL-FvH4.3g45680.1 | TNL-FvH4.3g45730.1 |

| Segmental duplication gene pairs | | | Tandem duplication gene pairs | | |
| --- | --- | --- | --- | --- | --- |
| ID of sequence 1 | ID of sequence 2 | ID of sequence 1 ID of sequence 2 | ID of sequence 1 ID of sequence 2 | ID of sequence 1 | ID of sequence 2 |

| TN-FPE.chr4.1012 | TNL-FPE.chr6.1904 | CNL-FvH4.6g51140 | CNL-FvH4.7g26370.1 | TN-FvH4.6g01300.1 | TNL-FvH4.3g43890.1 | TNL-FvH4.3g45680.1 | TNL-FvH4.3g44670.1 |
| --- | --- | --- | --- | --- | --- | --- | --- |

| TN-FNG.chr3.2354 | TNL-FNG.chr3.2352 | CNL-FvH4.6g47980 | CNL-FvH4.6g47050.1 | TN-FvH4.6g01300.1 | TNL-FvH4.1g17740.1 | TNL-FvH4.3g45680.1 | TNL-FvH4.3g44680.1 |
| --- | --- | --- | --- | --- | --- | --- | --- |
| TN-FNG.chr3.1991 | TNL-FNG.chr3.3322 | CNL-FvH4.6g02820 | CN-FvH4.1g02740.1 | TN-FvH4.5g34230.1 | TN-FvH4.5g34240.1 | TNL-FvH4.3g45680.1 | TNL-FvH4.3g45690.1 |
| TN-FII.chr5.2853 | TNL-FII.chr5.2851 | CNL-FvH4.6g02820 | CN-FvH4.6g02790.1 | TN-FvH4.3g45630.1 | TNL-FvH4.3g45680.1 | TNL-FvH4.3g45680.1 | TNL-FvH4.3g44570.1 |
| TN-FDA.chr7.02893 | TNL-FDA.chr7.02890 | CNL-FvH4.6g02820 | CNL-FvH4.4g35200.1 | TN-FvH4.3g45630.1 | TNL-FvH4.3g45690.1 | TNL-FvH4.3g45680.1 | TNL-FvH4.1g17740.1 |
| TN-FDA.chr6.26557 | TNL-FDA.chr3.09399 | CNL-FvH4.6g02820 | CNL-FvH4.6g02730 | TN-FvH4.3g45630.1 | TNL-FvH4.3g45700.1 | TNL-FvH4.3g45170.1 | TNL-FvH4.3g45680.1 |
| TN-FDA.chr6.02460 | TN-FDA.chr6.02329 | CNL-FvH4.6g02820 | CNL-FvH4.6g02760 | TN-FvH4.3g45630.1 | TNL-FvH4.3g45730.1 | TNL-FvH4.3g45170.1 | TNL-FvH4.3g45700.1 |
| TN-FDA.chr3.20745 | N-FDA.chr3.20742 | CNL-FvH4.6g02760 | CN-FvH4.1g02740.1 | TN-FvH4.3g45630.1 | TNL-FvH4.3g44570.1 | TNL-FvH4.3g45170.1 | TNL-FvH4.3g44570.1 |
| TN-FDA.chr3.05876 | TNL-FDA.chr3.05837 | CNL-FvH4.6g02760 | CNL-FvH4.4g35200.1 | TN-FvH4.3g45630.1 | TNL-FvH4.3g44680.1 | TNL-FvH4.3g44680.1 | TNL-FvH4.3g45730.1 |
| RNL-FvH4.6g42380.1 | RNL-FvH4.4g31860.1 | CNL-FvH4.6g02730 | CN-FvH4.1g02740.1 | TN-FvH4.3g45630.1 | TNL-FvH4.1g17740.1 | TNL-FvH4.3g44680.1 | TNL-FvH4.3g45700.1 |
| RNL-FPE.chr5.2037 | RNL-FPE.chr6.5624 | CNL-FvH4.6g02730 | CN-FvH4.6g02790.1 | TN-FvH4.3g45630.1 | TNL-FvH4.3g43950.1 | TNL-FvH4.3g44680.1 | TNL-FvH4.3g45690.1 |
| RNL-FPE.chr0.2431 | NL-FPE.chr2.4140 | CNL-FvH4.6g02730 | CNL-FvH4.4g35200.1 | TN-FvH4.1g22520.1 | TNL-FvH4.1g07020.1 | TNL-FvH4.3g44680.1 | TNL-FvH4.3g45170.1 |
| RNL-FDA.chr4.04994 | RNL-FDA.chr6.12990 | CNL-FvH4.6g02730 | CNL-FvH4.6g02760 | TN-FvH4.1g22520.1 | TNL-FvH4.1g22370.1 | TNL-FvH4.3g44680.1 | TNL-FvH4.3g44570.1 |
| RN-FvH4.6g50330.1 | RNL-FvH4.6g50310.1 | CNL-FvH4.5g23450 | CN-FvH4.5g23460.1 | TN-FNG.chr3.1991 | TNL-FNG.chr5.112 | TNL-FvH4.3g44680.1 | TNL-FvH4.1g17740.1 |
| RN-FNU.ctg81.619 | RN-FNU.ctg55.225 | CNL-FvH4.5g23450 | CNL-FvH4.5g23470.1 | TN-FDA.chr7.02893 | TNL-FDA.chr7.02892 | TNL-FvH4.3g44680.1 | TNL-FvH4.3g44670.1 |
| RN-FNU.ctg38.153 | RN-FNU.ctg52.52 | CNL-FvH4.5g23420 | CNL-FvH4.5g23430.1 | TN-FDA.chr7.00192 | TNL-FDA.chr7.00190 | TNL-FvH4.3g44670.1 | TNL-FvH4.3g45730.1 |
| NL-FvH4.4g15190.1 | CNL-FvH4.1g23030.1 | CNL-FvH4.5g23400 | CNL-FvH4.5g23390 | TN-FDA.chr7.00192 | TNL-FDA.chr7.00189 | TNL-FvH4.3g44670.1 | TNL-FvH4.3g45700.1 |
| NL-FvH4.3g35890.1 | TNL-FvH4.5g27640.1 | CNL-FvH4.5g23390 | CNL-FvH4.5g23420 | TN-FDA.chr7.00192 | TNL-FDA.chr7.00159 | TNL-FvH4.3g44670.1 | TNL-FvH4.3g45170.1 |
| NL-FPE.chr0.1234 | CNL-FPE.chr1.871 | CNL-FvH4.4g06030 | CNL-FvH4.5g02660.1 | TN-FDA.chr7.00191 | TNL-FDA.chr7.00189 | TNL-FvH4.3g44670.1 | TNL-FvH4.3g44570.1 |
| NL-FNU.ctg132.328 | NL-FNU.ctg134.34 | CNL-FvH4.4g06020 | CNL-FvH4.4g06030 | TN-FDA.chr7.00191 | TN-FDA.chr7.00192 | TNL-FvH4.3g44610.1 | TNL-FvH4.3g45730.1 |
| NL-FDA.chr7.18714 | NL-FDA.chr7.14306 | CNL-FvH4.4g06020 | CNL-FvH4.5g02660.1 | TN-FDA.chr7.00191 | TNL-FDA.chr7.00159 | TNL-FvH4.3g44610.1 | TNL-FvH4.3g45700.1 |
| NL-FDA.chr7.00705 | CN-FDA.chr7.00678 | CNL-FvH4.2g36860 | CNL-FvH4.2g36810.1 | TN-FDA.chr7.00191 | TN-FDA.chr7.00120 | TNL-FvH4.3g44610.1 | TNL-FvH4.3g44680.1 |
| NL-FDA.chr6.02338 | TNL-FDA.chr6.02345 | CNL-FvH4.2g36830 | CNL-FvH4.2g36810.1 | TN-FDA.chr7.00120 | TNL-FDA.chr7.00189 | TNL-FvH4.3g44610.1 | TNL-FvH4.3g45680.1 |
| NL-FDA.chr3.20838 | TNL-FDA.chr3.20747 | CNL-FvH4.2g36830 | CNL-FvH4.2g36860 | TN-FDA.chr7.00120 | TNL-FDA.chr7.00190 | TNL-FvH4.3g44610.1 | TNL-FvH4.3g45690.1 |
| NL-FDA.chr3.20676 | TNL-FDA.chr3.05837 | CNL-FvH4.2g36800 | CNL-FvH4.2g36810.1 | TN-FDA.chr7.00120 | TNL-FDA.chr7.00159 | TNL-FvH4.3g44610.1 | TNL-FvH4.3g44570.1 |
| NL-FDA.chr3.05440 | CNL-FDA.chr5.24059 | CNL-FvH4.2g17630 | CNL-FvH4.2g17640.1 | TN-FDA.chr7.00120 | TN-FDA.chr7.00192 | TNL-FvH4.3g44610.1 | TNL-FvH4.1g17740.1 |
| N-FvH4.7g09280.1 | CNL-FvH4.6g49940.1 | CNL-FPE.chr7.333 | CNL-FPE.chr7.335 | TN-FDA.chr7.00118 | TNL-FDA.chr7.00190 | TNL-FvH4.3g43960.1 | TNL-FvH4.3g43850.1 |
| N-FvH4.3g44060.1 | TN-FvH4.6g01300.1 | CNL-FPE.chr2.3627 | CNL-FPE.chr7.333 | TN-FDA.chr7.00118 | TNL-FDA.chr7.00110 | TNL-FvH4.3g43950.1 | TNL-FvH4.3g43890.1 |
| N-FvH4.3g12820.1 | RN-FvH4.6g50330.1 | CNL-FPE.chr2.3627 | CNL-FPE.chr7.335 | TN-FDA.chr7.00118 | TNL-FDA.chr7.00189 | TNL-FvH4.3g43950.1 | TNL-FvH4.3g43960.1 |
| N-FvH4.2g17400.1 | CNL-FvH4.6g02820 | CNL-FPE.chr1.871 | CNL-FPE.chr2.3627 | TN-FDA.chr7.00118 | TN-FDA.chr7.00120 | TNL-FvH4.3g43950.1 | TNL-FvH4.3g43850.1 |
| N-FPE.chr3.3480 | N-FPE.chr6.572 | CNL-FPE.chr1.871 | CNL-FPE.chr7.333 | TN-FDA.chr6.26557 | TNL-FDA.chr1.15663 | TNL-FvH4.3g43940.1 | TNL-FvH4.3g44000.1 |
| N-FPE.chr0.2268 | CN-FPE.chr0.885 | CNL-FPE.chr1.871 | CNL-FPE.chr7.335 | TN-FDA.chr6.26557 | TNL-FDA.chr4.10670 | TNL-FvH4.3g43940.1 | TNL-FvH4.3g43880.1 |
| N-FNU.ctg48.4 | CNL-FNU.ctg165.171 | CNL-FNU.ctg84.614 | CNL-FNU.ctg53.139 | TN-FDA.chr6.02460 | TNL-FDA.chr6.02345 | TNL-FvH4.3g43860.1 | TNL-FvH4.3g44000.1 |
| N-FNG.chr2.1856 | RNL-FNG.chr2.1787 | CNL-FNU.ctg84.614 | CNL-FNU.ctg53.185 | TN-FDA.chr6.02460 | TNL-FDA.chr6.02361 | TNL-FvH4.3g43860.1 | TNL-FvH4.3g43880.1 |
| N-FMA.chr6.2173 | CN-FMA.chr6.2719 | CNL-FNU.ctg84.614 | CNL-FNU.ctg53.289 | TN-FDA.chr6.02329 | TNL-FDA.chr6.02345 | TNL-FvH4.3g43650.1 | TNL-FvH4.3g43660.1 |
| N-FMA.chr6.2154 | CN-FMA.chr6.2734 | CNL-FNU.ctg84.614 | CNL-FNU.ctg53.85 | TN-FDA.chr6.02329 | TNL-FDA.chr6.02361 | TNL-FvH4.3g43650.1 | TNL-FvH4.3g43610.1 |
| N-FMA.chr2.489 | N-FMA.chr4.1879 | CNL-FNU.ctg53.85 | CN-FNU.ctg54.29 | TN-FDA.chr3.20745 | NL-FDA.chr3.20838 | TNL-FvH4.3g43630.1 | TNL-FvH4.3g43610.1 |
| N-FMA.chr2.311 | CNL-FMA.chr2.328 | CNL-FNU.ctg53.85 | CNL-FNU.ctg53.139 | TN-FDA.chr3.20724 | TN-FDA.chr3.20717 | TNL-FvH4.3g43630.1 | TNL-FvH4.3g43390.1 |
| N-FII.chr6.1246 | CN-FII.chr4.1429 | CNL-FNU.ctg53.85 | CNL-FNU.ctg53.185 | TN-FDA.chr3.20724 | TNL-FDA.chr3.20719 | TNL-FvH4.3g42940.1 | TNL-FvH4.3g45170.1 |
| N-FII.chr2.645 | TNL-FII.chr5.609 | CNL-FNU.ctg53.85 | CNL-FNU.ctg53.289 | TN-FDA.chr3.20720 | TNL-FDA.chr3.20719 | TNL-FvH4.3g42940.1 | TNL-FvH4.3g44670.1 |
| N-FDA.chr7.22742 | TNL-FDA.chr7.00274 | CNL-FNU.ctg53.185 | CNL-FNU.ctg53.139 | TN-FDA.chr3.20720 | TN-FDA.chr3.20717 | TNL-FvH4.2g17330.1 | N-FvH4.2g17340.1 |
| N-FDA.chr7.22742 | TNL-FDA.chr6.13989 | CNL-FNU.ctg53.185 | CNL-FNU.ctg53.289 | TN-FDA.chr3.20720 | TN-FDA.chr3.20724 | TNL-FvH4.2g00540.1 | TNL-FvH4.2g00550.1 |
| Segmental duplication gene pairs | | | | Tandem duplication gene pairs | | | |
| ID of sequence 1 | ID of sequence 2 | ID of sequence 1 ID of sequence 2 | | ID of sequence 1 ID of sequence 2 | | ID of sequence 1 | ID of sequence 2 |
| N-FDA.chr3.24469 | RN-FDA.chr6.13755 | CNL-FNU.ctg53.160 | CNL-FNU.ctg53.185 | RNL-FvH4.6g50130.1 | RNL-FvH4.6g50140.1 | TNL-FvH4.1g22380.1 | TNL-FvH4.1g07020.1 |
| N-FDA.chr3.20832 | CN-FDA.chr3.05842 | CNL-FNU.ctg53.160 | CNL-FNU.ctg53.289 | RNL-FvH4.6g50110.1 | N-FvH4.6g50120.1 | TNL-FvH4.1g22380.1 | TNL-FvH4.1g22390.1 |
| N-FDA.chr3.20808 | TNL-FDA.chr5.03488 | CNL-FNU.ctg53.160 | CNL-FNU.ctg53.85 | RNL-FvH4.6g50110.1 | RNL-FvH4.6g50130.1 | TNL-FvH4.1g22370.1 | TNL-FvH4.1g22380.1 |
| N-FDA.chr3.20675 | NL-FDA.chr3.20838 | CNL-FNU.ctg48.6 | N-FNU.ctg48.4 | RNL-FvH4.6g50100.1 | RNL-FvH4.6g50110.1 | TNL-FvH4.1g16640.1 | TNL-FvH4.1g16650.1 |
| N-FDA.chr2.11533 | CNL-FDA.chr6.02198 | CNL-FNU.ctg172.27 | N-FNU.ctg172.28 | RNL-FvH4.6g50100.1 | RNL-FvH4.1g17470.1 | TNL-FvH4.1g16620.1 | TNL-FvH4.1g16640.1 |
| CNL-FvH4.7g28390.1 | CNL-FvH4.7g27890.1 | CNL-FNU.ctg158.45 | CNL-FNU.ctg158.148 | RNL-FvH4.1g15230.1 | RN-FvH4.1g15240.1 | TNL-FvH4.1g16610.1 | TNL-FvH4.1g16620.1 |
| CNL-FvH4.7g28110.1 | CNL-FvH4.7g27330.1 | CNL-FNU.ctg158.45 | CNL-FNU.ctg158.46 | RNL-FvH4.1g15230.1 | RNL-FvH4.1g15170.1 | TNL-FvH4.1g16600.1 | TNL-FvH4.1g16610.1 |
| CNL-FvH4.7g23500.1 | CNL-FvH4.7g26370.1 | CNL-FNU.ctg158.42 | CNL-FNU.ctg158.43 | RNL-FvH4.1g15220.1 | RNL-FvH4.1g15230.1 | TNL-FvH4.1g11570.1 | TNL-FvH4.1g11580.1 |
| CNL-FvH4.6g53580.1 | CNL-FvH4.3g04780.1 | CNL-FNU.ctg158.14 | CNL-FNU.ctg158.149 | RNL-FvH4.1g15210.1 | RNL-FvH4.1g15220.1 | TNL-FPE.chr7.2368 | NL-FPE.chr7.2369 |
| CNL-FvH4.6g51550.1 | CNL-FvH4.6g51610.1 | CNL-FNG.chr5.654 | CNL-FNG.chr5.655 | RNL-FvH4.1g15160.1 | RNL-FvH4.1g15220.1 | TNL-FNU.ctg81.216 | TNL-FNU.ctg81.211 |
| CNL-FvH4.6g51140.1 | CNL-FvH4.7g23500.1 | CNL-FNG.chr5.2227 | CN-FNG.chr5.2228 | RNL-FvH4.1g15160.1 | RNL-FvH4.1g15170.1 | TNL-FNU.ctg81.216 | TNL-FNU.ctg81.212 |
| CNL-FvH4.6g48310.1 | CNL-FvH4.6g47050.1 | CNL-FNG.chr5.2150 | CNL-FNG.chr5.2227 | RNL-FvH4.1g15130.1 | RN-FvH4.1g15140.1 | TNL-FNU.ctg81.212 | TNL-FNU.ctg81.211 |
| CNL-FvH4.6g48170.1 | CNL-FvH4.6g47050.1 | CNL-FNG.chr5.2149 | CNL-FNG.chr5.2150 | RNL-FvH4.1g15120.1 | RNL-FvH4.1g15130.1 | TNL-FNU.ctg3.75 | TNL-FNU.ctg3.73 |
| CNL-FvH4.6g47980.1 | CNL-FvH4.6g48170.1 | CNL-FNG.chr5.2146 | CNL-FNG.chr5.2147 | RNL-FNG.chr6.4113 | RNL-FNG.chr6.4114 | TNL-FNU.ctg3.22 | TNL-FNU.ctg3.21 |
| CNL-FvH4.6g15090.1 | NL-FvH4.4g18270.1 | CNL-FNG.chr5.2145 | CNL-FNG.chr5.2146 | RNL-FNG.chr1.1336 | RNL-FNG.chr1.1337 | TNL-FNU.ctg173.410 | TN-FNU.ctg173.411 |
| CNL-FvH4.5g23420 | CNL-FvH4.5g22710.1 | CNL-FNG.chr3.1370 | CNL-FNG.chr3.1371 | RNL-FMA.chr1.1807 | RNL-FMA.chr1.1808 | TNL-FNU.ctg172.195 | TNL-FNU.ctg172.196 |
| CNL-FvH4.5g18950.1 | CNL-FvH4.5g19000.1 | CNL-FII.chr7.206 | CNL-FII.chr7.207 | RNL-FMA.chr1.1806 | RNL-FMA.chr1.1807 | TNL-FNU.ctg172.194 | TNL-FNU.ctg172.195 |
| CNL-FvH4.4g35200.1 | CN-FvH4.1g02740.1 | CNL-FII.chr5.3063 | NL-FII.chr5.3064 | RNL-FII.chr1.1270 | RNL-FII.chr1.1269 | TNL-FMA.chr4.2945 | TNL-FMA.chr4.2946 |
| CNL-FvH4.4g29930.1 | CNL-FvH4.4g29310.1 | CNL-FII.chr5.2007 | CNL-FII.chr5.2008 | RNL-FII.chr1.1267 | NL-FII.chr1.1268 | TNL-FMA.chr2.2280 | TNL-FMA.chr2.2281 |
| CNL-FvH4.3g04780.1 | CNL-FvH4.3g05070.1 | CNL-FII.chr5.2006 | CNL-FII.chr5.2007 | RNL-FII.chr1.1266 | RNL-FII.chr1.1267 | TNL-FMA.chr0.553 | TNL-FMA.chr0.554 |
| CNL-FPE.chr5.3277 | CNL-FPE.chr6.2574 | CNL-FII.chr5.2004 | NL-FII.chr5.3064 | RNL-FII.chr1.1265 | RNL-FII.chr1.1266 | TNL-FII.chr7.936 | TN-FII.chr7.937 |
| CNL-FNU.ctg84.614 | CNL-FNU.ctg53.160 | CNL-FII.chr5.2003 | CNL-FII.chr5.2001 | RNL-FDA.chr1.22190 | CNL-FDA.chr1.22189 | TNL-FII.chr5.3370 | TNL-FII.chr5.3371 |
| CNL-FNU.ctg53.139 | CNL-FNU.ctg53.289 | CNL-FII.chr5.2003 | CNL-FII.chr5.2004 | RNL-FDA.chr1.22190 | RNL-FDA.chr1.22183 | TNL-FII.chr5.2970 | TN-FII.chr5.2971 |
| CNL-FNU.ctg48.6 | CNL-FNU.ctg30.47 | CNL-FII.chr5.2003 | CNL-FII.chr5.3063 | RNL-FDA.chr1.22187 | RN-FDA.chr1.22186 | TNL-FII.chr5.2969 | TNL-FII.chr5.2970 |
| CNL-FNU.ctg169.439 | CNL-FNU.ctg170.24 | CNL-FII.chr5.2002 | CNL-FII.chr5.2003 | RNL-FDA.chr1.22184 | RNL-FDA.chr1.22183 | TNL-FII.chr4.2875 | TNL-FII.chr4.2876 |
| CNL-FNU.ctg158.48 | CNL-FNU.ctg158.148 | CNL-FII.chr5.2001 | CNL-FII.chr5.2002 | RNL-FDA.chr1.22183 | RNL-FDA.chr1.22182 | TNL-FII.chr3.1830 | TNL-FII.chr3.1831 |
| CNL-FNU.ctg156.8 | CNL-FNU.ctg155.369 | CNL-FII.chr5.2001 | CNL-FII.chr5.3063 | RNL-FDA.chr1.22179 | RN-FDA.chr1.22178 | TNL-FII.chr1.1393 | TNL-FII.chr1.1394 |
| CNL-FNU.ctg145.8 | NL-FNU.ctg144.28 | CNL-FII.chr3.956 | CNL-FII.chr3.951 | RN-FvH4.6g42370.1 | RNL-FvH4.6g42380.1 | TNL-FDA.chr7.00190 | TNL-FDA.chr7.00159 |
| CNL-FNG.chr5.2146 | CNL-FNG.chr5.2089 | CNL-FII.chr3.956 | CNL-FII.chr3.954 | RN-FvH4.1g15200.1 | RNL-FvH4.1g15210.1 | N-FvH4.2g17400.1 | CNL-FvH4.6g02760 |
| CNL-FNG.chr1.545 | CNL-FNG.chr3.1371 | CNL-FII.chr3.954 | CN-FII.chr3.955 | RN-FvH4.1g15200.1 | RNL-FvH4.1g15130.1 | N-FPE.chr3.1891 | N-FPE.chr3.1892 |
| CNL-FMA.chr1.1369 | NL-FMA.chr1.1431 | CNL-FII.chr3.954 | CNL-FII.chr3.951 | RN-FDA.chr1.22186 | RNL-FDA.chr1.22179 | N-FNU.ctg172.28 | RN-FNU.ctg172.29 |
| CNL-FII.chr7.186 | CNL-FII.chr7.207 | CNL-FII.chr3.953 | CNL-FII.chr3.951 | NL-FvH4.6g05490.1 | TNL-FvH4.6g04730.1 | N-FNU.ctg119.819 | N-FNU.ctg119.817 |
| CNL-FII.chr6.2256 | CNL-FII.chr6.2546 | CNL-FII.chr3.953 | CNL-FII.chr3.954 | NL-FvH4.6g05490.1 | TNL-FvH4.6g05610.1 | N-FNU.ctg119.818 | N-FNU.ctg119.819 |
| CNL-FDA.chr7.01259 | CNL-FDA.chr7.01250 | CNL-FII.chr3.1384 | CNL-FII.chr3.1385 | NL-FvH4.6g05490.1 | NL-FvH4.6g04350.1 | N-FNU.ctg119.817 | N-FNU.ctg119.818 |
| CNL-FDA.chr7.00809 | CNL-FDA.chr7.01119 | CNL-FDA.chr6.13872 | CNL-FDA.chr7.01119 | NL-FvH4.6g04350.1 | TNL-FvH4.6g05610.1 | N-FMA.chr2.1406 | TNL-FMA.chr2.1405 |
| CNL-FDA.chr6.26146 | CNL-FDA.chr6.26337 | CNL-FDA.chr6.02058 | CNL-FDA.chr6.02056 | NL-FvH4.6g04350.1 | TNL-FvH4.6g04730.1 | CN-FDA.chr5.25046 | CNL-FDA.chr5.25047 |
| CNL-FDA.chr6.14104 | CNL-FDA.chr7.00307 | CNL-FDA.chr5.25049 | CNL-FDA.chr5.25050 | NL-FPE.chr0.1234 | CNL-FPE.chr7.335 | CN-FDA.chr3.05842 | N-FDA.chr3.20836 |
| CNL-FDA.chr6.13908 | CNL-FDA.chr6.02010 | CNL-FDA.chr5.25047 | CNL-FDA.chr5.25044 | NL-FPE.chr0.1234 | CNL-FPE.chr7.333 | CN-FDA.chr3.05842 | TN-FDA.chr3.05844 |
| CNL-FDA.chr6.13872 | CNL-FDA.chr7.00809 | CNL-FDA.chr3.16968 | CNL-FDA.chr7.00307 | NL-FPE.chr0.1234 | CNL-FPE.chr2.3627 | CN-FDA.chr2.12217 | CNL-FDA.chr2.12218 |
| CNL-FDA.chr6.02459 | TNL-FDA.chr6.02345 | CNL-FDA.chr3.05895 | TNL-FDA.chr3.05894 | NL-FMA.chr2.856 | TNL-FMA.chr2.857 | CN-FDA.chr2.12212 | CN-FDA.chr2.12213 |
| CNL-FDA.chr5.25044 | CNL-FDA.chr5.24966 | CNL-FDA.chr3.05890 | TNL-FDA.chr3.05894 | NL-FII.chr1.1268 | RNL-FII.chr1.1269 | CN-FDA.chr2.12212 | CNL-FDA.chr2.12208 |
| CNL-FDA.chr5.24966 | CNL-FDA.chr5.25047 | CNL-FDA.chr3.05890 | TNL-FDA.chr3.05887 | NL-FDA.chr7.00705 | CN-FDA.chr7.00892 | CN-FII.chr3.950 | CNL-FII.chr3.953 |
| Segmental duplication gene pairs | | | | Tandem duplication gene pairs | | | |
| ID of sequence 1 | ID of sequence 2 | ID of sequence 1 ID of sequence 2 | | ID of sequence 1 ID of sequence 2 | | ID of sequence 1 | ID of sequence 2 |
| CNL-FDA.chr5.23812 | CNL-FDA.chr7.00307 | CNL-FDA.chr2.12208 | CNL-FDA.chr2.12209 | NL-FDA.chr7.00111 | TNL-FDA.chr7.00184 | CN-FII.chr3.950 | CNL-FII.chr3.954 |
| CNL-FDA.chr4.11333 | CNL-FDA.chr5.24089 | CNL-FDA.chr2.12098 | TNL-FDA.chr5.23994 | NL-FDA.chr7.00111 | TNL-FDA.chr7.00189 | CN-FII.chr3.950 | CNL-FII.chr3.956 |
| CNL-FDA.chr3.16978 | CNL-FDA.chr7.00307 | CNL-FDA.chr1.22189 | RNL-FDA.chr1.22182 | NL-FDA.chr7.00111 | TNL-FDA.chr7.00190 |  |  |
| CNL-FDA.chr3.16968 | CNL-FDA.chr3.16978 | CN-FvH4.6g02790.1 | CN-FvH4.1g02740.1 | NL-FDA.chr7.00111 | TNL-FDA.chr7.00159 |  |  |
| CNL-FDA.chr3.16968 | CNL-FDA.chr6.14104 | CN-FvH4.6g02790.1 | CNL-FvH4.4g35200.1 | NL-FDA.chr7.00111 | TNL-FDA.chr7.00110 |  |  |
| CNL-FDA.chr3.05890 | TNL-FDA.chr3.05887 | CN-FvH4.6g02790.1 | CNL-FvH4.6g02760 | NL-FDA.chr3.20746 | NL-FDA.chr3.20838 |  |  |
| CNL-FDA.chr2.12098 | TNL-FDA.chr3.09474 | CN-FvH4.5g23460.1 | CNL-FvH4.5g23470.1 | NL-FDA.chr1.18083 | NL-FDA.chr3.20838 |  |  |
| CN-FvH4.7g23850.1 | CNL-FvH4.7g22030.1 | CN-FvH4.2g36850.1 | CNL-FvH4.2g36860 | NL-FDA.chr1.18083 | TNL-FDA.chr3.20747 |  |  |
| CN-FvH4.6g51570.1 | CNL-FvH4.2g17640.1 | CN-FPE.chr4.1617 | CNL-FPE.chr4.1619 | N-FvH4.6g50120.1 | RNL-FvH4.6g50140.1 |  |  |
| CN-FvH4.6g15230.1 | CNL-FvH4.6g15250.1 | CN-FPE.chr2.3023 | CNL-FPE.chr6.2574 | N-FvH4.6g50120.1 | RNL-FvH4.1g17470.1 |  |  |
| CN-FvH4.6g02790.1 | N-FvH4.2g17400.1 | CN-FNU.ctg54.29 | CNL-FNU.ctg53.139 | N-FvH4.6g50120.1 | RNL-FvH4.6g50100.1 |  |  |
| CN-FPE.chr5.3016 | CN-FPE.chr7.2093 | CN-FNU.ctg54.29 | CNL-FNU.ctg53.185 | N-FvH4.6g50120.1 | RNL-FvH4.6g50130.1 |  |  |
| CN-FPE.chr4.1090 | CNL-FPE.chr4.1619 | CN-FNU.ctg54.29 | CNL-FNU.ctg53.289 | N-FvH4.5g21530.1 | RNL-FvH4.1g15220.1 |  |  |
| CN-FPE.chr2.3023 | CNL-FPE.chr5.3277 | CN-FNU.ctg53.159 | CN-FNU.ctg54.29 | N-FvH4.5g21530.1 | RNL-FvH4.1g15160.1 |  |  |
| CN-FNU.ctg71.38 | CN-FNU.ctg70.1 | CN-FNU.ctg53.159 | CNL-FNU.ctg53.185 | N-FvH4.3g44060.1 | TNL-FvH4.3g45680.1 |  |  |
| CN-FNU.ctg64.1 | CN-FNU.ctg63.62 | CN-FNU.ctg53.159 | CNL-FNU.ctg53.289 | N-FvH4.3g44060.1 | TNL-FvH4.1g17740.1 |  |  |
| CN-FNU.ctg53.159 | CN-FNU.ctg53.173 | CN-FNU.ctg53.159 | CNL-FNU.ctg53.85 | N-FvH4.3g12820.1 | RNL-FvH4.6g50310.1 |  |  |
| CN-FNU.ctg144.27 | CNL-FNU.ctg145.8 | CN-FNU.ctg158.39 | CNL-FNU.ctg158.40 | N-FvH4.2g17400.1 | CN-FvH4.1g02740.1 |  |  |
| CN-FNG.chr0.49 | CN-FNG.chr5.2696 | CN-FMA.chr3.1343 | CNL-FMA.chr3.1344 | N-FvH4.2g17400.1 | CNL-FvH4.4g35200.1 |  |  |
| CN-FMA.chr2.312 | CNL-FMA.chr2.328 | CN-FII.chr3.955 | CNL-FII.chr3.951 | N-FvH4.2g17400.1 | CNL-FvH4.6g02730 |  |  |
| CN-FII.chr6.2258 | CN-FII.chr6.2548 | CN-FII.chr3.955 | CNL-FII.chr3.956 | N-FPE.chr3.1892 | N-FPE.chr3.1895 |  |  |
| CN-FDA.chr7.00678 | CN-FDA.chr7.00892 | CN-FII.chr3.950 | CN-FII.chr3.955 |  |  |  |  |
| CN-FDA.chr6.13604 | CNL-FDA.chr6.13561 | CN-FII.chr3.950 | CNL-FII.chr3.951 |  |  |  |  |
| CN-FDA.chr2.11532 | CNL-FDA.chr6.02198 |  |  |  |  |  |  |

**Table S4b** List of NLR gene family orthologous gene pairs

| Orthologous gene pairsof TNL | | | | | Orthologous gene pairs of non-TNL | | | | | | | | | | | | | | |
| --- | --- | --- | --- | --- | --- | --- | --- | --- | --- | --- | --- | --- | --- | --- | --- | --- | --- | --- | --- |
| ID of sequence 1 | ID of sequence 2 | | | | ID of sequence 1 | ID of sequence 2 | ID of sequence 1 | | | | ID of sequence 2 | | | | | | | | |
| *TN-FDA.chr2.08147* | *TNL-FII.chr2.1158* | | | | *CN-FDA.chr1.06861* | *CN-FNG.chr1.338* | *CNL-FvH4.5g18950.1* | | | | *CNL-FMA.chr5.1739* | | | | | | | | |
| *TN-FDA.chr2.08147* | *TNL-FMA.chr5.2877* | | | | *CN-FDA.chr1.06861* | *CNL-FNU.ctg114.210* | *CNL-FvH4.4g29310.1* | | | | *NL-FDA.chr4.05233* | | | | | | | | |
| *TN-FDA.chr2.08147* | *TNL-FvH4.2g14040.1* | | | | *CN-FDA.chr1.06861* | *CNL-FPE.chr1.871* | *CNL-FvH4.4g29930.1* | | | | *CNL-FNU.ctg81.894* | | | | | | | | |
| *TN-FDA.chr2.12371* | *TNL-FII.chr2.77* | | | | *CN-FDA.chr1.06861* | *CNL-FvH4.6g49940.1* | *CNL-FvH4.4g29930.1* | | | | *CNL-FPE.chr3.4782* | | | | | | | | |
| *TN-FDA.chr2.12371* | *TNL-FMA.chr0.565* | | | | *CN-FDA.chr2.11532* | *CN-FII.chr6.198* | *CNL-FvH4.4g29930.1* | | | | *CNL-FMA.chr7.880* | | | | | | | | |
| *TN-FDA.chr2.12371* | *TNL-FNU.ctg101.35* | | | | *CN-FDA.chr2.11532* | *CN-FvH4.1g02740.1* | *CNL-FvH4.4g35200.1* | | | | *CN-FII.chr6.198* | | | | | | | | |
| *TN-FDA.chr2.12371* | *TNL-FPE.chr6.5657* | | | | *CN-FDA.chr2.11532* | *CNL-FNG.chr2.1430* | *CNL-FvH4.4g35200.1* | | | | *CNL-FNG.chr2.1430* | | | | | | | | |
| *TN-FDA.chr2.12371* | *TNL-FvH4.1g22540.1* | | | | *CN-FDA.chr2.12212* | *CNL-FvH4.2g36810.1* | *CNL-FvH4.5g02660.1* | | | | *CNL-FII.chr4.377* | | | | | | | | |
| *TN-FDA.chr3.05844* | *TNL-FvH4.3g43950.1* | | | | *CN-FDA.chr2.12213* | *CNL-FvH4.2g36860.1* | *CNL-FvH4.5g02660.1* | | | | *CNL-FMA.chr4.679* | | | | | | | | |
| *TN-FDA.chr3.05876* | *TNL-FII.chr3.3603* | | | | *CN-FDA.chr2.12217* | *CNL-FvH4.2g36830.1* | *CNL-FvH4.5g02660.1* | | | | *CNL-FNG.chr0.411* | | | | | | | | |
| *TN-FDA.chr3.05876* | *TNL-FNG.chr5.112* | | | | *CN-FDA.chr2.17950* | *CN-FII.chr2.453* | *CNL-FvH4.5g02660.1* | | | | *CNL-FDA.chr5.24089* | | | | | | | | |
| *TN-FDA.chr3.05876* | *TNL-FNU.ctg3.75* | | | | *CN-FDA.chr2.17950* | *CN-FMA.chr2.312* | *CNL-FvH4.5g05380.1* | | | | *CNL-FII.chr3.408* | | | | | | | | |
| *TN-FDA.chr3.05876* | *TNL-FvH4.3g44070.1* | | | | *CN-FDA.chr2.17950* | *CN-FNU.ctg104.161* | *CNL-FvH4.5g05380.1* | | | | *CNL-FPE.chr3.535* | | | | | | | | |
| *TN-FDA.chr3.09422* | *TNL-FII.chr3.3419* | | | | *CN-FDA.chr2.17950* | *CN-FvH4.2g05600.1* | *CNL-FvH4.5g16110.1* | | | | *CNL-FII.chr5.3003* | | | | | | | | |
| *TN-FDA.chr3.09422* | *TNL-FMA.chr1.1780* | | | | *CN-FDA.chr2.17950* | *CNL-FNG.chr2.416* | *CNL-FvH4.5g16110.1* | | | | *CNL-FNU.ctg147.19* | | | | | | | | |
| *TN-FDA.chr3.09422* | *TNL-FNU.ctg105.95* | | | | *CN-FDA.chr2.17950* | *CNL-FPE.chr7.1798* | *CNL-FvH4.5g16110.1* | | | | *CNL-FPE.chr1.2047* | | | | | | | | |
| *TN-FDA.chr3.09422* | *TNL-FvH4.3g44580.1* | | | | *CN-FDA.chr5.03686* | *CNL-FNG.chr5.3271* | *CNL-FvH4.5g16900.1* | | | | *CNL-FDA.chr6.02010* | | | | | | | | |
| *TN-FDA.chr3.09564* | *TN-FvH4.7g11580.1* | | | | *CN-FDA.chr5.03686* | *CNL-FvH4.5g32430.1* | *CNL-FvH4.5g16900.1* | | | | *CNL-FNU.ctg154.468* | | | | | | | | |
| *TN-FDA.chr3.09564* | *TNL-FII.chr6.136* | | | | *CN-FDA.chr5.25046* | *CN-FvH4.5g23460.1* | *CNL-FvH4.5g18950.1* | | | | *NL-FNU.ctg155.6* | | | | | | | | |
| *TN-FDA.chr3.09564* | *TNL-FNU.ctg129.394* | | | | *CN-FDA.chr5.25046* | *CNL-FII.chr5.2007* | *CNL-FvH4.5g19000.1* | | | | *CNL-FDA.chr5.07881* | | | | | | | | |
| *TN-FDA.chr3.15822* | *TNL-FII.chr3.1313* | | | | *CN-FDA.chr5.25046* | *CNL-FNG.chr5.2149* | *CNL-FvH4.5g19000.1* | | | | *CNL-FNG.chr5.1760* | | | | | | | | |
| *TN-FDA.chr3.15822* | *TNL-FMA.chr2.2218* | | | | *CN-FDA.chr5.25046* | *CNL-FNU.ctg158.46* | *CNL-FvH4.5g22710.1* | | | | *CNL-FDA.chr5.25050* | | | | | | | | |
| *TN-FDA.chr3.15822* | *TNL-FNU.ctg32.58* | | | | *CN-FDA.chr6.13604* | *CNL-FNU.ctg53.85* | *CNL-FvH4.5g22710.1* | | | | *CNL-FII.chr5.3063* | | | | | | | | |
| *TN-FDA.chr3.15822* | *TNL-FPE.chr5.1409* | | | | *CN-FDA.chr6.13604* | *CNL-FvH4.6g47980.1* | *CNL-FvH4.5g22710.1* | | | | *CNL-FNU.ctg158.42* | | | | | | | | |
| *TN-FDA.chr3.15822* | *TNL-FvH4.3g15720.1* | | | | *CN-FDA.chr6.16615* | *CN-FII.chr6.3270.2* | *CNL-FvH4.5g23390.1* | | | | *CNL-FDA.chr5.25050* | | | | | | | | |
| *TN-FDA.chr3.20717* | *TNL-FNU.ctg3.22* | | | | *CN-FDA.chr6.16615* | *CN-FMA.chr6.2632* | *CNL-FvH4.5g23390.1* | | | | *CNL-FNG.chr5.2146* | | | | | | | | |
| *TN-FDA.chr3.20717* | *TNL-FvH4.3g44370.1* | | | | *CN-FDA.chr6.16615* | *CN-FNG.chr6.3269* | *CNL-FvH4.5g23390.1* | | | | *CNL-FNU.ctg158.42* | | | | | | | | |
| *TN-FDA.chr3.20720* | *TNL-FNU.ctg3.21* | | | | *CN-FDA.chr6.16615* | *CN-FNU.ctg57.114* | *CNL-FvH4.5g23400.1* | | | | *CNL-FDA.chr5.25050* | | | | | | | | |
| *TN-FDA.chr3.20720* | *TNL-FvH4.3g44390.1* | | | | *CN-FDA.chr6.16615* | *CN-FPE.chr6.3007* | *CNL-FvH4.5g23400.1* | | | | *CNL-FNU.ctg158.40* | | | | | | | | |
| *TN-FDA.chr3.20724* | *TNL-FNU.ctg3.21* | | | | *CN-FDA.chr6.16615* | *CN-FvH4.6g29230.1* | *CNL-FvH4.5g23420.1* | | | | *CNL-FNU.ctg158.42* | | | | | | | | |
| *TN-FDA.chr3.20724* | *TNL-FvH4.3g44390.1* | | | | *CN-FDA.chr6.17288* | *CN-FII.chr6.2548* | *CNL-FvH4.5g23430.1* | | | | *CNL-FNU.ctg158.43* | | | | | | | | |
| *TN-FDA.chr3.20745* | *TNL-FvH4.3g44610.1* | | | | *CN-FDA.chr6.17288* | *CN-FMA.chr6.2632* | *CNL-FvH4.5g23450.1* | | | | *CNL-FNU.ctg158.45* | | | | | | | | |
| *TN-FDA.chr5.03486* | *TN-FII.chr5.2971* | | | | *CN-FDA.chr6.17288* | *CN-FNG.chr6.1594* | *CNL-FvH4.5g23470.1* | | | | *CNL-FNU.ctg158.48* | | | | | | | | |
| *TN-FDA.chr5.03486* | *TNL-FvH4.3g45380.1* | | | | *CN-FDA.chr6.17288* | *CN-FNU.ctg65.105* | *CNL-FvH4.5g32430.1* | | | | *CNL-FDA.chr3.16699* | | | | | | | | |
| *TN-FDA.chr5.23634* | *TNL-FII.chr5.609* | | | | *CN-FDA.chr6.17288* | *CN-FPE.chr6.1820* | *CNL-FvH4.5g32430.1* | | | | *NL-FNU.ctg161.49* | | | | | | | | |
| *TN-FDA.chr5.23634* | *TNL-FMA.chr5.364* | | | | *CN-FDA.chr6.17288* | *CN-FvH4.6g29230.1* | *CNL-FvH4.5g33290.1* | | | | *CNL-FDA.chr3.16699* | | | | | | | | |
| *TN-FDA.chr5.23634* | *TNL-FvH4.5g07310.1* | | | | *CN-FDA.chr6.17380* | *CN-FII.chr6.2648* | *CNL-FvH4.5g33290.1* | | | | *CNL-FNG.chr5.3271* | | | | | | | | |
| *TN-FDA.chr6.02329* | *TN-FNG.chr3.2312* | | | | *CN-FDA.chr6.17380* | *CN-FMA.chr6.2734* | *CNL-FvH4.5g33290.1* | | | | *NL-FNU.ctg161.49* | | | | | | | | |
| *TN-FDA.chr6.02329* | *TNL-FII.chr6.322* | | | | *CN-FDA.chr6.17380* | *CN-FNU.ctg64.1* | *CNL-FvH4.5g34680.1* | | | | *CNL-FNU.ctg82.313* | | | | | | | | |
| *TN-FDA.chr6.02329* | *TNL-FvH4.6g04250.1* | | | | *CN-FDA.chr6.17380* | *CN-FPE.chr6.470* | *CNL-FvH4.6g02730.1* | | | | *CN-FII.chr6.198* | | | | | | | | |
| Orthologous gene pairsof TNL | | | | | Orthologous gene pairs of non-TNL | | | | | | | | | | | | | | |
| ID of sequence 1 | ID of sequence 2 | | | | ID of sequence 1 | ID of sequence 2 | ID of sequence 1 | | | | | | | | | | ID of sequence 2 | | |
| *TN-FDA.chr6.02460* | *TN-FNG.chr3.2312* | | | | *CN-FDA.chr6.17380* | *N-FvH4.6g30240.1* | *CNL-FvH4.6g02730.1* | | | | *CNL-FDA.chr6.02198* | | | | | | | | |
| *TN-FDA.chr6.02460* | *TNL-FII.chr6.322* | | | | *CN-FDA.chr6.19192* | *CNL-FII.chr4.1808* | *CNL-FvH4.6g02730.1* | | | | *CNL-FNG.chr2.1430* | | | | | | | | |
| *TN-FDA.chr6.02460* | *TNL-FvH4.6g04730.1* | | | | *CN-FDA.chr6.19192* | *CNL-FMA.chr6.3395* | *CNL-FvH4.6g02760.1* | | | | *CNL-FDA.chr6.02198* | | | | | | | | |
| *TN-FDA.chr6.26557* | *TNL-FII.chr1.757* | | | | *CN-FDA.chr6.19192* | *CNL-FPE.chr2.397* | *CNL-FvH4.6g02760.1* | | | | *CNL-FNG.chr2.1430* | | | | | | | | |
| *TN-FDA.chr6.26557* | *TNL-FPE.chr6.1904* | | | | *CN-FDA.chr6.19192* | *CNL-FvH4.6g34770.1* | *CNL-FvH4.6g02820.1* | | | | *CN-FII.chr6.198* | | | | | | | | |
| *TN-FDA.chr6.26557* | *TNL-FvH4.3g10020.1* | | | | *CN-FDA.chr6.19192* | *N-FNU.ctg57.541* | *CNL-FvH4.6g02820.1* | | | | *CNL-FDA.chr6.02198* | | | | | | | | |
| *TN-FDA.chr7.00118* | *TNL-FvH4.7g33190.1* | | | | *CN-FDA.chr6.20969* | *CN-FII.chr6.1890* | *CNL-FvH4.6g12200.1* | | | | *CNL-FNU.ctg78.2* | | | | | | | | |
| *TN-FDA.chr7.00120* | *TNL-FvH4.7g32760.1* | | | | *CN-FDA.chr6.20969* | *CN-FMA.chr6.1745* | *CNL-FvH4.6g13450.1* | | | | *CNL-FNU.ctg78.2* | | | | | | | | |
| *TN-FDA.chr7.00191* | *TNL-FvH4.7g32470.1* | | | | *CN-FDA.chr6.20969* | *CN-FNG.chr6.1541* | *CNL-FvH4.6g15090.1* | | | | *CNL-FNU.ctg78.2* | | | | | | | | |
| *TN-FDA.chr7.00192* | *TNL-FvH4.7g32760.1* | | | | *CN-FDA.chr6.20969* | *CN-FNU.ctg71.38* | *CNL-FvH4.6g15090.1* | | | | *NL-FDA.chr6.26447* | | | | | | | | |
| *TN-FDA.chr7.02893* | *TN-FII.chr7.977* | | | | *CN-FDA.chr6.20969* | *CN-FPE.chr0.885* | *CNL-FvH4.6g15250.1* | | | | *CNL-FMA.chr6.1184* | | | | | | | | |
| *TN-FDA.chr7.02893* | *TN-FvH4.7g11160.1* | | | | *CN-FDA.chr6.20969* | *CN-FvH4.6g22150.1* | *CNL-FvH4.6g15340.1* | | | | *CNL-FMA.chr6.1658* | | | | | | | | |
| *TN-FDA.chr7.02893* | *TNL-FNU.ctg129.394* | | | | *CN-FDA.chr7.00678* | *CNL-FPE.chr7.1571* | *CNL-FvH4.6g15340.1* | | | | *CNL-FNU.ctg78.2* | | | | | | | | |
| *TN-FDA.chr7.21088* | *TNL-FII.chr7.857* | | | | *CN-FDA.chr7.00678* | *CNL-FvH4.7g28390.1* | *CNL-FvH4.6g29480.1* | | | | *CNL-FDA.chr6.13967* | | | | | | | | |
| *TN-FDA.chr7.21088* | *TNL-FMA.chr2.1405* | | | | *CN-FDA.chr7.00892* | *CNL-FPE.chr7.1571* | *CNL-FvH4.6g30120.1* | | | | *CNL-FNU.ctg64.14.1* | | | | | | | | |
| *TN-FDA.chr7.21088* | *TNL-FNU.ctg135.24* | | | | *CN-FDA.chr7.00892* | *CNL-FvH4.7g27330.1* | *CNL-FvH4.6g32110.1* | | | | *CNL-FDA.chr3.16699* | | | | | | | | |
| *TN-FDA.chr7.21088* | *TNL-FvH4.7g09800.1* | | | | *CN-FDA.chr7.20537* | *CNL-FNU.ctg114.210* | *CNL-FvH4.6g34770.1* | | | | *CN-FNG.chr5.2228* | | | | | | | | |
| *TN-FII.chr1.2339* | *TN-FNG.chr1.1802* | | | | *CN-FDA.chr7.20537* | *CNL-FPE.chr2.3627* | *CNL-FvH4.6g34770.1* | | | | *N-FNU.ctg57.541* | | | | | | | | |
| *TN-FII.chr1.2339* | *TN-FNU.ctg177.55* | | | | *CN-FDA.chr7.20537* | *CNL-FvH4.6g49940.1* | *CNL-FvH4.6g47050.1* | | | | *CNL-FDA.chr6.13561* | | | | | | | | |
| *TN-FII.chr1.2339* | *TN-FvH4.1g27020.1* | | | | *CN-FII.chr1.1528* | *CNL-FDA.chr3.09595* | *CNL-FvH4.6g47050.1* | | | | *CNL-FNU.ctg53.139* | | | | | | | | |
| *TN-FII.chr2.1653* | *TN-FDA.chr7.00120* | | | | *CN-FII.chr1.1528* | *CNL-FMA.chr4.679* | *CNL-FvH4.6g47980.1* | | | | *CNL-FDA.chr6.13561* | | | | | | | | |
| *TN-FII.chr2.1653* | *TNL-FvH4.7g32460.1* | | | | *CN-FII.chr1.1528* | *CNL-FNG.chr0.411* | *CNL-FvH4.6g47980.1* | | | | *CNL-FNU.ctg53.139* | | | | | | | | |
| *TN-FII.chr2.560* | *TNL-FDA.chr3.20725* | | | | *CN-FII.chr1.1528* | *CNL-FvH4.5g18950.1* | *CNL-FvH4.6g48170.1* | | | | *CNL-FDA.chr6.13561* | | | | | | | | |
| *TN-FII.chr2.560* | *TNL-FMA.chr1.1780* | | | | *CN-FII.chr1.1941* | *CN-FPE.chr7.1432* | *CNL-FvH4.6g48170.1* | | | | *CNL-FNU.ctg53.139* | | | | | | | | |
| *TN-FII.chr2.633* | *TNL-FDA.chr3.20725* | | | | *CN-FII.chr1.1941* | *CNL-FDA.chr6.02010* | *CNL-FvH4.6g48220.1* | | | | *CN-FNU.ctg53.150* | | | | | | | | |
| *TN-FII.chr2.633* | *TNL-FMA.chr5.2877* | | | | *CN-FII.chr1.1941* | *CNL-FvH4.1g23030.1* | *CNL-FvH4.6g48310.1* | | | | *CNL-FNU.ctg53.139* | | | | | | | | |
| *TN-FII.chr2.633* | *TNL-FNU.ctg105.95* | | | | *CN-FII.chr2.453* | *CN-FMA.chr2.312* | *CNL-FvH4.6g49940.1* | | | | *CNL-FNU.ctg114.210* | | | | | | | | |
| *TN-FII.chr2.633* | *TNL-FvH4.3g44390.1* | | | | *CN-FII.chr2.453* | *CN-FNU.ctg104.161* | *CNL-FvH4.6g51140.1* | | | | *CNL-FNU.ctg129.61* | | | | | | | | |
| *TN-FII.chr3.1974* | *TNL-FDA.chr3.05837* | | | | *CN-FII.chr2.453* | *CN-FvH4.2g05600.1* | *CNL-FvH4.6g51550.1* | | | | *CNL-FDA.chr6.13908* | | | | | | | | |
| *TN-FII.chr3.1974* | *TNL-FNG.chr5.112* | | | | *CN-FII.chr2.453* | *CNL-FNG.chr2.416* | *CNL-FvH4.6g51610.1* | | | | *CNL-FDA.chr6.13908* | | | | | | | | |
| *TN-FII.chr3.1974* | *TNL-FNU.ctg3.75* | | | | *CN-FII.chr2.453* | *CNL-FPE.chr7.1798* | *CNL-FvH4.6g53580.1* | | | | *CNL-FII.chr3.408* | | | | | | | | |
| *TN-FII.chr3.1974* | *TNL-FvH4.5g01270.1* | | | | *CN-FII.chr2.469* | *CN-FvH4.2g05720.1* | *CNL-FvH4.6g53580.1* | | | | *CNL-FPE.chr3.535* | | | | | | | | |
| *TN-FII.chr5.2853* | *TN-FvH4.5g32990.1* | | | | *CN-FII.chr2.551* | *CN-FMA.chr2.503* | *CNL-FvH4.7g01950.1* | | | | *CNL-FNG.chr7.204* | | | | | | | | |
| *TN-FII.chr5.2853* | *TNL-FDA.chr5.25282* | | | | *CN-FII.chr2.551* | *CN-FPE.chr7.728* | *CNL-FvH4.7g01950.1* | | | | *CNL-FNU.ctg146.100* | | | | | | | | |
| *TN-FII.chr5.2853* | *TNL-FNU.ctg160.483* | | | | *CN-FII.chr2.551* | *CN-FvH4.2g06550.1* | *CNL-FvH4.7g02400.1* | | | | *CNL-FPE.chr7.1571* | | | | | | | | |
| *TN-FII.chr5.2971* | *TNL-FDA.chr5.03488* | | | | *CN-FII.chr2.551* | *CNL-FNG.chr2.482* | *CNL-FvH4.7g02450.1* | | | | *CNL-FPE.chr7.1571* | | | | | | | | |
| *TN-FII.chr5.2971* | *TNL-FvH4.3g45380.1* | | | | *CN-FII.chr2.551* | *CNL-FNU.ctg104.299* | *CNL-FvH4.7g02890.1* | | | | *CNL-FNU.ctg145.8* | | | | | | | | |
| *TN-FII.chr6.4140* | *TN-FvH4.6g49490.1* | | | | *CN-FII.chr3.2661* | *CN-FNG.chr4.2529* | *CNL-FvH4.7g08100.1* | | | | *CNL-FNU.ctg114.210* | | | | | | | | |
| *TN-FII.chr6.4140* | *TNL-FNU.ctg160.483* | | | | *CN-FII.chr3.2661* | *CN-FNU.ctg20.194* | *CNL-FvH4.7g08870.1* | | | | *CNL-FNU.ctg114.210* | | | | | | | | |
| *TN-FII.chr7.888* | *TNL-FvH4.1g01230.1* | | | | *CN-FII.chr3.2661* | *CNL-FMA.chr3.2744* | *CNL-FvH4.7g08870.1* | | | | *NL-FPE.chr0.1234* | | | | | | | | |
| *TN-FII.chr7.937* | *TN-FDA.chr7.21088* | | | | *CN-FII.chr3.2661* | *CNL-FPE.chr7.1015* | *CNL-FvH4.7g11380.1* | | | | *NL-FNU.ctg132.328* | | | | | | | | |
| *TN-FII.chr7.937* | *TN-FNG.chr7.573* | | | | *CN-FII.chr3.2661* | *CNL-FvH4.4g29930.1* | *CNL-FvH4.7g15190.1* | | | | *CNL-FII.chr7.789* | | | | | | | | |
| Orthologous gene pairsof TNL | | | | | Orthologous gene pairs of non-TNL | | | | | | | | | | | | | | |
| ID of sequence 1 | ID of sequence 2 | | | | ID of sequence 1 | ID of sequence 2 | ID of sequence 1 | | | | | | | ID of sequence 2 | | | | | |
| *TN-FII.chr7.937* | *TNL-FNU.ctg135.24* | | | | *CN-FII.chr3.2924* | *CN-FvH4.3g21750.1* | *CNL-FvH4.7g15190.1* | | | | *CNL-FNU.ctg114.210* | | | | | | | | |
| *TN-FII.chr7.937* | *TNL-FPE.chr2.3663* | | | | *CN-FII.chr3.2924* | *CNL-FMA.chr3.2975* | *CNL-FvH4.7g15190.1* | | | | *CNL-FPE.chr7.1124* | | | | | | | | |
| *TN-FII.chr7.977* | *TN-FvH4.7g11160.1* | | | | *CN-FII.chr3.2924* | *CNL-FNU.ctg21.293* | *CNL-FvH4.7g15240.1* | | | | *NL-FNU.ctg131.54* | | | | | | | | |
| *TN-FII.chr7.977* | *TNL-FNU.ctg129.394* | | | | *CN-FII.chr3.2924* | *CNL-FPE.chr1.1624* | *CNL-FvH4.7g20580.1* | | | | *CNL-FNU.ctg129.61* | | | | | | | | |
| *TN-FMA.chr0.347* | *TN-FPE.chr5.3159* | | | | *CN-FII.chr3.950* | *CNL-FvH4.3g11490.1* | *CNL-FvH4.7g22030.1* | | | | *CNL-FNU.ctg129.61* | | | | | | | | |
| *TN-FMA.chr0.347* | *TNL-FDA.chr3.20725* | | | | *CN-FII.chr3.955* | *CNL-FvH4.3g11490.1* | *CNL-FvH4.7g22140.1* | | | | *CNL-FDA.chr7.01250* | | | | | | | | |
| *TN-FMA.chr0.347* | *TNL-FII.chr2.1158* | | | | *CN-FII.chr3.955* | *N-FPE.chr3.1891* | *CNL-FvH4.7g22140.1* | | | | *CNL-FNU.ctg129.61* | | | | | | | | |
| *TN-FMA.chr0.347* | *TNL-FNU.ctg105.95* | | | | *CN-FII.chr4.1180* | *CN-FPE.chr7.1432* | *CNL-FvH4.7g23500.1* | | | | *CNL-FNU.ctg129.61* | | | | | | | | |
| *TN-FMA.chr0.347* | *TNL-FvH4.3g44390.1* | | | | *CN-FII.chr4.1180* | *CNL-FDA.chr6.02010* | *CNL-FvH4.7g26370.1* | | | | *CNL-FDA.chr7.01119* | | | | | | | | |
| *TN-FMA.chr3.2485* | *TNL-FDA.chr7.01326* | | | | *CN-FII.chr4.1180* | *CNL-FvH4.1g23030.1* | *CNL-FvH4.7g26370.1* | | | | *CNL-FNU.ctg129.61* | | | | | | | | |
| *TN-FMA.chr3.2485* | *TNL-FII.chr2.77* | | | | *CN-FII.chr4.1429* | *CN-FNU.ctg84.31* | *CNL-FvH4.7g27330.1* | | | | *CNL-FII.chr7.55* | | | | | | | | |
| *TN-FMA.chr3.2485* | *TNL-FPE.chr6.5657* | | | | *CN-FII.chr4.1429* | *CN-FPE.chr4.843* | *CNL-FvH4.7g27890.1* | | | | *CNL-FII.chr7.55* | | | | | | | | |
| *TN-FMA.chr3.2485* | *TNL-FvH4.3g33030.1* | | | | *CN-FII.chr4.1429* | *CNL-FMA.chr6.1508* | *CNL-FvH4.7g27890.1* | | | | *CNL-FPE.chr7.1571* | | | | | | | | |
| *TN-FMA.chr3.3317* | *TNL-FDA.chr3.05639* | | | | *CN-FII.chr4.1429* | *CNL-FNG.chr6.2406* | *CNL-FvH4.7g28110.1* | | | | *CN-FDA.chr7.00892* | | | | | | | | |
| *TN-FMA.chr3.3317* | *TNL-FNG.chr3.3168* | | | | *CN-FII.chr4.1429* | *CNL-FvH4.6g15090.1* | *CNL-FvH4.7g28110.1* | | | | *CNL-FII.chr7.55* | | | | | | | | |
| *TN-FMA.chr3.3317* | *TNL-FNU.ctg132.96* | | | | *CN-FII.chr5.2883* | *CN-FDA.chr5.03686* | *CNL-FvH4.7g28110.1* | | | | *CNL-FPE.chr7.1571* | | | | | | | | |
| *TN-FMA.chr3.3317* | *TNL-FvH4.3g38670.1* | | | | *CN-FII.chr5.2883* | *CNL-FNG.chr5.3271* | *CNL-FvH4.7g28390.1* | | | | *CN-FDA.chr7.00892* | | | | | | | | |
| *TN-FMA.chr5.2917* | *TNL-FDA.chr5.12803* | | | | *CN-FII.chr5.2883* | *CNL-FvH4.5g32430.1* | *CNL-FvH4.7g28390.1* | | | | *CNL-FII.chr7.55* | | | | | | | | |
| *TN-FMA.chr5.2917* | *TNL-FII.chr5.3330* | | | | *CN-FII.chr6.1267* | *CNL-FDA.chr6.26153* | *CNL-FvH4.7g28390.1* | | | | *CNL-FPE.chr7.1571* | | | | | | | | |
| *TN-FMA.chr5.2917* | *TNL-FNG.chr2.105* | | | | *CN-FII.chr6.1267* | *CNL-FMA.chr6.1508* | *CNL-FvH4.7g31140.1* | | | | *CNL-FII.chr3.408* | | | | | | | | |
| *TN-FMA.chr5.2917* | *TNL-FPE.chr0.1078* | | | | *CN-FII.chr6.1267* | *CNL-FNG.chr6.2420* | *CNL-FvH4.7g31140.1* | | | | *CNL-FPE.chr3.535* | | | | | | | | |
| *TN-FMA.chr5.2917* | *TNL-FvH4.5g38540.1* | | | | *CN-FII.chr6.1267* | *CNL-FvH4.6g15250.1* | *CNL-FvH4.7g33440.1* | | | | *CNL-FII.chr4.1808* | | | | | | | | |
| *TN-FNG.chr1.1802* | *TN-FNU.ctg177.55* | | | | *CN-FII.chr6.1890* | *CN-FMA.chr6.1745* | *CNL-FvH4.7g33440.1* | | | | *CNL-FNU.ctg82.648* | | | | | | | | |
| *TN-FNG.chr1.1802* | *TN-FvH4.1g27020.1* | | | | *CN-FII.chr6.1890* | *CN-FNG.chr6.1541* | *CNL-FVI.CHR1.1117* | | | | *CNL-FII.chr1.1071* | | | | | | | | |
| *TN-FNG.chr2.1155* | *TNL-FDA.chr6.02345* | | | | *CN-FII.chr6.1890* | *CN-FNU.ctg71.38* | *CNL-FVI.CHR1.1117* | | | | *CNL-FMA.chr1.1369* | | | | | | | | |
| *TN-FNG.chr2.1155* | *TNL-FII.chr6.322* | | | | *CN-FII.chr6.1890* | *CN-FPE.chr0.885* | *CNL-FVI.CHR1.1117* | | | | *CNL-FNU.ctg170.24* | | | | | | | | |
| *TN-FNG.chr2.1155* | *TNL-FMA.chr2.857* | | | | *CN-FII.chr6.1890* | *CN-FvH4.6g22150.1* | *CNL-FVI.CHR1.1117* | | | | *CNL-FPE.chr6.1273* | | | | | | | | |
| *TN-FNG.chr2.1155* | *TNL-FNU.ctg8.247* | | | | *CN-FII.chr6.198* | *CNL-FNG.chr2.1430* | *CNL-FVI.CHR1.1117* | | | | *N-FvH4.1g12710.1* | | | | | | | | |
| *TN-FNG.chr2.1155* | *TNL-FPE.chr3.3124* | | | | *CN-FII.chr6.198* | *CNL-FvH4.6g02760.1* | *CNL-FVI.CHR1.1117* | | | | *NL-FNG.chr1.1121* | | | | | | | | |
| *TN-FNG.chr2.1155* | *TNL-FvH4.2g14320.1* | | | | *CN-FII.chr6.2258* | *CN-FDA.chr6.17288* | *CNL-FVI.CHR1.1853* | | | | *CNL-FDA.chr6.02010* | | | | | | | | |
| *TN-FNG.chr3.1991* | *TN-FII.chr3.1974* | | | | *CN-FII.chr6.2258* | *CN-FMA.chr6.2632* | *CNL-FVI.CHR1.1853* | | | | *CNL-FvH4.6g51610.1* | | | | | | | | |
| *TN-FNG.chr3.1991* | *TNL-FDA.chr3.20678* | | | | *CN-FII.chr6.2258* | *CN-FNG.chr6.1594* | *CNL-FVI.CHR1.1874* | | | | *CNL-FDA.chr6.02010* | | | | | | | | |
| *TN-FNG.chr3.1991* | *TNL-FvH4.5g01270.1* | | | | *CN-FII.chr6.2258* | *CN-FNU.ctg65.105* | *CNL-FVI.CHR1.1874* | | | | *CNL-FvH4.1g23030.1* | | | | | | | | |
| *TN-FNG.chr3.2312* | *TN-FNU.ctg8.251* | | | | *CN-FII.chr6.2258* | *CN-FPE.chr6.1820* | *CNL-FVI.CHR1.572* | | | | *CN-FvH4.3g16720.1* | | | | | | | | |
| *TN-FNG.chr3.2312* | *TNL-FII.chr6.322* | | | | *CN-FII.chr6.2258* | *CN-FvH4.6g29230.1* | *CNL-FVI.CHR1.572* | | | | *CNL-FII.chr3.1385* | | | | | | | | |
| *TN-FNG.chr3.2312* | *TNL-FvH4.6g04250.1* | | | | *CN-FII.chr6.2548* | *CN-FMA.chr6.2632* | *CNL-FVI.CHR1.572* | | | | *CNL-FMA.chr3.1344* | | | | | | | | |
| *TN-FNG.chr3.2354* | *TNL-FII.chr3.1831* | | | | *CN-FII.chr6.2548* | *CN-FNG.chr6.1594* | *CNL-FVI.CHR1.572* | | | | *CNL-FNG.chr1.545* | | | | | | | | |
| *TN-FNG.chr3.2354* | *TNL-FvH4.7g12160.1* | | | | *CN-FII.chr6.2548* | *CN-FNU.ctg65.105* | *CNL-FVI.CHR1.572* | | | | *CNL-FNU.ctg165.171* | | | | | | | | |
| *TN-FNG.chr3.760* | *TN-FNU.ctg84.632* | | | | *CN-FII.chr6.2548* | *CN-FPE.chr6.1820* | *CNL-FVI.CHR1.572* | | | | *CNL-FPE.chr5.124* | | | | | | | | |
| *TN-FNG.chr3.760* | *TNL-FDA.chr3.09523* | | | | *CN-FII.chr6.2548* | *CN-FvH4.6g29230.1* | *CNL-FVI.CHR1.572* | | | | *NL-FDA.chr3.21153* | | | | | | | | |
| *TN-FNG.chr3.760* | *TNL-FII.chr3.744.2* | | | | *CN-FII.chr6.2623* | *CN-FMA.chr6.2719* | *CNL-FVI.CHR2.1431* | | | | *CNL-FMA.chr6.2723* | | | | | | | | |
| *TN-FNG.chr3.760* | *TNL-FPE.chr6.1904* | | | | *CN-FII.chr6.2633* | *CN-FNU.ctg65.11* | *CNL-FVI.CHR2.1431* | | | | *CNL-FNG.chr6.2058* | | | | | | | | |
| Orthologous gene pairsof TNL | | | | | Orthologous gene pairs of non-TNL | | | | | | | | | | | | | | |
| ID of sequence 1 | ID of sequence 2 | | | | ID of sequence 1 | ID of sequence 2 | ID of sequence 1 | | | | | | | | | ID of sequence 2 | | | |
| *TN-FNG.chr3.760* | *TNL-FvH4.3g10020.1* | | | | *CN-FII.chr6.2633* | *CN-FPE.chr6.453* | *CNL-FVI.CHR2.1431* | | | | *CNL-FNU.ctg64.14.1* | | | | | | | | |
| *TN-FNG.chr4.253* | *TNL-FDA.chr3.09523* | | | | *CN-FII.chr6.2633* | *CNL-FDA.chr5.03688* | *CNL-FVI.CHR2.1431* | | | | *CNL-FvH4.6g30120.1* | | | | | | | | |
| *TN-FNG.chr4.253* | *TNL-FII.chr2.1158* | | | | *CN-FII.chr6.2633* | *CNL-FMA.chr6.2723* | *CNL-FVI.CHR2.1431* | | | | *NL-FII.chr6.2634* | | | | | | | | |
| *TN-FNG.chr4.253* | *TNL-FNU.ctg105.95* | | | | *CN-FII.chr6.2633* | *CNL-FNG.chr6.2058* | *CNL-FVI.CHR2.1471* | | | | *CNL-FII.chr3.951* | | | | | | | | |
| *TN-FNG.chr4.253* | *TNL-FvH4.3g44370.1* | | | | *CN-FII.chr6.2633* | *CNL-FvH4.6g30120.1* | *CNL-FVI.CHR2.1471* | | | | *CNL-FPE.chr6.2574* | | | | | | | | |
| *TN-FNG.chr7.1408* | *TNL-FDA.chr7.01621* | | | | *CN-FII.chr6.2648* | *CN-FMA.chr6.2734* | *CNL-FVI.CHR2.1471* | | | | *CNL-FvH4.3g11470.1* | | | | | | | | |
| *TN-FNG.chr7.1408* | *TNL-FPE.chr7.2368* | | | | *CN-FII.chr6.2648* | *CN-FNU.ctg64.1* | *CNL-FVI.CHR2.2054* | | | | *CNL-FII.chr2.1235* | | | | | | | | |
| *TN-FNG.chr7.1408* | *TNL-FvH4.7g17700.1* | | | | *CN-FII.chr6.2648* | *CN-FPE.chr6.470* | *CNL-FVI.CHR2.2054* | | | | *CNL-FvH4.2g14930.1* | | | | | | | | |
| *TN-FNG.chr7.573* | *TNL-FII.chr7.936* | | | | *CN-FII.chr6.2648* | *N-FvH4.6g30240.1* | *CNL-FVI.CHR2.2280* | | | | *CNL-FDA.chr6.13908* | | | | | | | | |
| *TN-FNG.chr7.573* | *TNL-FMA.chr2.1405* | | | | *CN-FII.chr6.2656* | *CNL-FDA.chr3.16699* | *CNL-FVI.CHR2.2280* | | | | *CNL-FvH4.2g17640.1* | | | | | | | | |
| *TN-FNG.chr7.573* | *TNL-FNU.ctg109.139* | | | | *CN-FII.chr6.2656* | *CNL-FvH4.6g29480.1* | *CNL-FVI.CHR2.3860* | | | | *CNL-FDA.chr6.02010* | | | | | | | | |
| *TN-FNG.chr7.573* | *TNL-FPE.chr2.3663* | | | | *CN-FII.chr6.2817* | *CNL-FDA.chr3.16699* | *CNL-FVI.CHR2.3860* | | | | *CNL-FvH4.1g23030.1* | | | | | | | | |
| *TN-FNG.chr7.573* | *TNL-FvH4.7g10750.1* | | | | *CN-FII.chr6.2817* | *CNL-FvH4.6g32110.1* | *CNL-FVI.CHR2.555* | | | | *CN-FII.chr2.551* | | | | | | | | |
| *TN-FNU.ctg10.27* | *TNL-FDA.chr2.12354* | | | | *CN-FII.chr6.3270.2* | *CN-FMA.chr6.2632* | *CNL-FVI.CHR2.555* | | | | *CN-FMA.chr2.503* | | | | | | | | |
| *TN-FNU.ctg10.27* | *TNL-FII.chr2.49* | | | | *CN-FII.chr6.3270.2* | *CN-FNG.chr6.3269* | *CNL-FVI.CHR2.555* | | | | *CN-FPE.chr7.728* | | | | | | | | |
| *TN-FNU.ctg10.27* | *TNL-FPE.chr2.91* | | | | *CN-FII.chr6.3270.2* | *CN-FNU.ctg57.114* | *CNL-FVI.CHR2.555* | | | | *CN-FvH4.2g06550.1* | | | | | | | | |
| *TN-FNU.ctg173.411* | *TNL-FDA.chr1.07161* | | | | *CN-FII.chr6.3270.2* | *CN-FPE.chr6.3007* | *CNL-FVI.CHR2.555* | | | | *CNL-FDA.chr2.19060* | | | | | | | | |
| *TN-FNU.ctg173.411* | *TNL-FMA.chr0.565* | | | | *CN-FII.chr6.3270.2* | *CN-FvH4.6g29230.1* | *CNL-FVI.CHR2.555* | | | | *CNL-FNG.chr2.482* | | | | | | | | |
| *TN-FNU.ctg173.411* | *TNL-FvH4.1g07020.1* | | | | *CN-FII.chr7.1958* | *CNL-FDA.chr7.01192* | *CNL-FVI.CHR2.555* | | | | *CNL-FNU.ctg104.299* | | | | | | | | |
| *TN-FNU.ctg173.413* | *TNL-FDA.chr1.07161* | | | | *CN-FII.chr7.1958* | *CNL-FNU.ctg129.61* | *CNL-FVI.CHR3.185* | | | | *CNL-FII.chr3.1384* | | | | | | | | |
| *TN-FNU.ctg173.413* | *TNL-FMA.chr0.565* | | | | *CN-FII.chr7.1958* | *CNL-FvH4.6g32110.1* | *CNL-FVI.CHR3.185* | | | | *CNL-FMA.chr3.1344* | | | | | | | | |
| *TN-FNU.ctg173.413* | *TNL-FvH4.1g22390.1* | | | | *CN-FMA.chr0.199* | *CNL-FII.chr7.55* | *CNL-FVI.CHR3.185* | | | | *CNL-FNG.chr3.1370* | | | | | | | | |
| *TN-FNU.ctg8.251* | *TNL-FDA.chr6.02361* | | | | *CN-FMA.chr0.199* | *CNL-FPE.chr7.237* | *CNL-FVI.CHR3.185* | | | | *CNL-FNU.ctg30.47* | | | | | | | | |
| *TN-FNU.ctg8.251* | *TNL-FII.chr6.322* | | | | *CN-FMA.chr0.199* | *NL-FvH4.7g00850.1* | *CNL-FVI.CHR3.185* | | | | *CNL-FPE.chr5.124* | | | | | | | | |
| *TN-FNU.ctg8.251* | *TNL-FPE.chr3.3124* | | | | *CN-FMA.chr2.312* | *CN-FNU.ctg104.161* | *CNL-FVI.CHR3.618* | | | | *CN-FII.chr3.2924* | | | | | | | | |
| *TN-FNU.ctg8.251* | *TNL-FvH4.3g36130.1* | | | | *CN-FMA.chr2.312* | *CN-FvH4.2g05600.1* | *CNL-FVI.CHR3.618* | | | | *CN-FvH4.3g21750.1* | | | | | | | | |
| *TN-FNU.ctg84.632* | *TNL-FDA.chr3.09523* | | | | *CN-FMA.chr2.312* | *CNL-FNG.chr2.416* | *CNL-FVI.CHR3.618* | | | | *CNL-FMA.chr3.2975* | | | | | | | | |
| *TN-FNU.ctg84.632* | *TNL-FII.chr3.744.2* | | | | *CN-FMA.chr2.312* | *CNL-FPE.chr7.1798* | *CNL-FVI.CHR3.618* | | | | *CNL-FNG.chr3.2531* | | | | | | | | |
| *TN-FNU.ctg84.632* | *TNL-FPE.chr6.1904* | | | | *CN-FMA.chr2.503* | *CN-FPE.chr7.728* | *CNL-FVI.CHR3.618* | | | | *CNL-FNU.ctg21.293* | | | | | | | | |
| *TN-FNU.ctg84.632* | *TNL-FvH4.4g13420.1* | | | | *CN-FMA.chr2.503* | *CN-FvH4.2g06550.1* | *CNL-FVI.CHR3.618* | | | | *CNL-FPE.chr1.1624* | | | | | | | | |
| *TN-FPE.chr1.445* | *TN-FDA.chr3.09422* | | | | *CN-FMA.chr2.503* | *CNL-FNG.chr2.482* | *CNL-FVI.CHR5.1412* | | | | *CNL-FDA.chr6.02010* | | | | | | | | |
| *TN-FPE.chr1.445* | *TN-FNG.chr4.253* | | | | *CN-FMA.chr2.503* | *CNL-FNU.ctg104.299* | *CNL-FVI.CHR5.1412* | | | | *CNL-FNU.ctg154.468* | | | | | | | | |
| *TN-FPE.chr1.445* | *TNL-FII.chr1.757* | | | | *CN-FMA.chr3.1343* | *CN-FvH4.3g16720.1* | *CNL-FVI.CHR5.1412* | | | | *CNL-FvH4.5g16900.1* | | | | | | | | |
| *TN-FPE.chr1.445* | *TNL-FvH4.7g04050.1* | | | | *CN-FMA.chr3.1343* | *CNL-FII.chr3.1384* | *CNL-FVI.CHR5.1617* | | | | *CNL-FDA.chr5.07881* | | | | | | | | |
| *TN-FPE.chr4.1012* | *TNL-FDA.chr4.10670* | | | | *CN-FMA.chr3.1343* | *CNL-FNG.chr3.1370* | *CNL-FVI.CHR5.1617* | | | | *CNL-FMA.chr5.1739* | | | | | | | | |
| *TN-FPE.chr4.1012* | *TNL-FII.chr3.3419* | | | | *CN-FMA.chr3.1343* | *CNL-FNU.ctg48.6* | *CNL-FVI.CHR5.1617* | | | | *CNL-FNG.chr5.1760* | | | | | | | | |
| *TN-FPE.chr4.1012* | *TNL-FvH4.4g13420.1* | | | | *CN-FMA.chr3.1343* | *N-FPE.chr5.126* | *CNL-FVI.CHR5.1617* | | | | *CNL-FvH4.5g18950.1* | | | | | | | | |
| *TN-FPE.chr5.245* | *TNL-FDA.chr2.20138* | | | | *CN-FMA.chr3.2162* | *NL-FNU.ctg155.6* | *CNL-FVI.CHR5.1617* | | | | *NL-FNU.ctg155.6* | | | | | | | | |
| *TN-FPE.chr5.245* | *TNL-FMA.chr2.2218* | | | | *CN-FMA.chr5.2235* | *CN-FNG.chr5.2228* | *CNL-FVI.CHR5.2975* | | | | *CNL-FII.chr5.3003* | | | | | | | | |
| *TN-FPE.chr5.245* | *TNL-FNG.chr2.2347* | | | | *CN-FMA.chr5.2235* | *CN-FPE.chr1.928* | *CNL-FVI.CHR5.2975* | | | | *CNL-FNG.chr5.2999* | | | | | | | | |
| *TN-FPE.chr5.245* | *TNL-FNU.ctg114.235* | | | | *CN-FMA.chr5.2235* | *CN-FvH4.5g24310.1* | *CNL-FVI.CHR5.2975* | | | | *CNL-FNU.ctg82.313* | | | | | | | | |
| *TN-FPE.chr5.245* | *TNL-FvH4.2g27070.1* | | | | *CN-FMA.chr5.2235* | *CNL-FII.chr4.1808* | *CNL-FVI.CHR5.2975* | | | | *CNL-FPE.chr1.2047* | | | | | | | | |
| Orthologous gene pairsof TNL | | | | | Orthologous gene pairs of non-TNL | | | | | | | | | | | | | | |
| ID of sequence 1 | ID of sequence 2 | | | | ID of sequence 1 | ID of sequence 2 | ID of sequence 1 | | | | | | | | ID of sequence 2 | | | | |
| *TN-FPE.chr5.3159* | *TNL-FDA.chr5.12815* | | | | *CN-FMA.chr5.2235* | *CNL-FNU.ctg158.149* | *CNL-FVI.CHR5.2975* | | | | *CNL-FvH4.5g34680.1* | | | | | | | | |
| *TN-FPE.chr5.3159* | *TNL-FII.chr5.3371* | | | | *CN-FMA.chr6.1745* | *CN-FNG.chr6.1541* | *CNL-FVI.CHR5.2975* | | | | *N-FMA.chr5.2639* | | | | | | | | |
| *TN-FPE.chr5.3159* | *TNL-FvH4.1g15650.1* | | | | *CN-FMA.chr6.1745* | *CN-FNU.ctg71.38* | *CNL-FVI.CHR5.3016* | | | | *CN-FNG.chr5.2692* | | | | | | | | |
| *TN-FPE.chr6.5875* | *TNL-FII.chr7.1130* | | | | *CN-FMA.chr6.1745* | *CN-FPE.chr0.885* | *CNL-FVI.CHR5.3016* | | | | *CNL-FDA.chr5.22369* | | | | | | | | |
| *TN-FPE.chr6.5875* | *TNL-FMA.chr5.2425* | | | | *CN-FMA.chr6.1745* | *CN-FvH4.6g22150.1* | *CNL-FVI.CHR5.3016* | | | | *CNL-FMA.chr5.2388* | | | | | | | | |
| *TN-FPE.chr6.5875* | *TNL-FNG.chr6.244* | | | | *CN-FMA.chr6.2632* | *CN-FNG.chr6.1594* | *CNL-FVI.CHR5.3016* | | | | *CNL-FvH4.7g31140.1* | | | | | | | | |
| *TN-FPE.chr6.5875* | *TNL-FvH4.7g13090.1* | | | | *CN-FMA.chr6.2632* | *CN-FNU.ctg65.105* | *CNL-FVI.CHR6.1106* | | | | *CNL-FDA.chr6.26146* | | | | | | | | |
| *TN-FPE.chr7.406* | *TNL-FDA.chr3.20725* | | | | *CN-FMA.chr6.2632* | *CN-FPE.chr6.1820* | *CNL-FVI.CHR6.1106* | | | | *CNL-FMA.chr6.1184* | | | | | | | | |
| *TN-FPE.chr7.406* | *TNL-FII.chr2.1158* | | | | *CN-FMA.chr6.2632* | *CN-FvH4.6g29230.1* | *CNL-FVI.CHR6.1106* | | | | *CNL-FvH4.6g15340.1* | | | | | | | | |
| *TN-FPE.chr7.406* | *TNL-FNU.ctg105.95* | | | | *CN-FMA.chr6.2734* | *CN-FNU.ctg64.1* | *CNL-FVI.CHR6.1768* | | | | *CNL-FDA.chr6.26146* | | | | | | | | |
| *TN-FPE.chr7.406* | *TNL-FvH4.3g44390.1* | | | | *CN-FMA.chr6.2734* | *CN-FPE.chr6.470* | *CNL-FVI.CHR6.1768* | | | | *CNL-FMA.chr6.1658* | | | | | | | | |
| *TN-FvH4.1g22520.1* | *TNL-FDA.chr1.07161* | | | | *CN-FMA.chr6.2734* | *N-FvH4.6g30240.1* | *CNL-FVI.CHR6.1768* | | | | *CNL-FNU.ctg78.2* | | | | | | | | |
| *TN-FvH4.1g22520.1* | *TNL-FMA.chr0.553* | | | | *CN-FMA.chr6.4269* | *CN-FNU.ctg53.150* | *CNL-FVI.CHR6.1768* | | | | *CNL-FvH4.6g15340.1* | | | | | | | | |
| *TN-FvH4.1g22520.1* | *TNL-FNU.ctg173.410* | | | | *CN-FMA.chr6.4269* | *CNL-FNG.chr5.655* | *CNL-FVI.CHR6.3417* | | | | *CNL-FDA.chr6.13561* | | | | | | | | |
| *TN-FvH4.1g27020.1* | *TN-FNU.ctg177.55* | | | | *CN-FMA.chr6.4269* | *CNL-FvH4.6g48220.1* | *CNL-FVI.CHR6.3417* | | | | *CNL-FNU.ctg53.139* | | | | | | | | |
| *TN-FvH4.2g20690.1* | *TN-FNU.ctg177.55* | | | | *CN-FMA.chr7.847* | *CN-FDA.chr7.14798* | *CNL-FVI.CHR6.3417* | | | | *CNL-FvH4.6g48310.1* | | | | | | | | |
| *TN-FvH4.2g20690.1* | *TNL-FII.chr2.1754* | | | | *CN-FMA.chr7.847* | *CN-FPE.chr6.3765* | *CNL-FVI.CHR6.3578* | | | | *CNL-FNU.ctg114.210* | | | | | | | | |
| *TN-FvH4.2g20690.1* | *TNL-FNG.chr2.1702* | | | | *CN-FMA.chr7.847* | *CN-FvH4.7g11820.1* | *CNL-FVI.CHR6.3578* | | | | *CNL-FPE.chr7.335* | | | | | | | | |
| *TN-FvH4.3g09290.1* | *TN-FPE.chr5.3159* | | | | *CN-FMA.chr7.847* | *CNL-FII.chr7.1027* | *CNL-FVI.CHR6.3578* | | | | *CNL-FvH4.6g49940.1* | | | | | | | | |
| *TN-FvH4.3g09290.1* | *TNL-FDA.chr3.09474* | | | | *CN-FMA.chr7.847* | *CNL-FNG.chr7.951* | *CNL-FVI.CHR7.1021* | | | | *CNL-FII.chr7.998* | | | | | | | | |
| *TN-FvH4.3g09290.1* | *TNL-FII.chr5.302.2* | | | | *CN-FMA.chr7.847* | *CNL-FNU.ctg132.293* | *CNL-FVI.CHR7.1021* | | | | *CNL-FMA.chr7.880* | | | | | | | | |
| *TN-FvH4.3g09290.1* | *TNL-FMA.chr5.364* | | | | *CN-FNG.chr0.49* | *CN-FII.chr6.2258* | *CNL-FVI.CHR7.1021* | | | | *CNL-FPE.chr3.4782* | | | | | | | | |
| *TN-FvH4.3g35840.1* | *TN-FMA.chr3.3317* | | | | *CN-FNG.chr0.49* | *CN-FNU.ctg161.296* | *CNL-FVI.CHR7.1021* | | | | *CNL-FvH4.7g11380.1* | | | | | | | | |
| *TN-FvH4.3g35840.1* | *TNL-FDA.chr3.05636* | | | | *CN-FNG.chr0.49* | *CN-FPE.chr5.3016* | *CNL-FVI.CHR7.1021* | | | | *NL-FNU.ctg132.328* | | | | | | | | |
| *TN-FvH4.3g35840.1* | *TNL-FNG.chr7.2518* | | | | *CN-FNG.chr1.338* | *CNL-FNU.ctg114.210* | *CNL-FVI.CHR7.1051* | | | | *CN-FPE.chr6.3765* | | | | | | | | |
| *TN-FvH4.3g35840.1* | *TNL-FNU.ctg132.96* | | | | *CN-FNG.chr1.338* | *CNL-FPE.chr1.871* | *CNL-FVI.CHR7.1051* | | | | *CNL-FII.chr7.1027* | | | | | | | | |
| *TN-FvH4.5g07190.1* | *TN-FDA.chr5.23634* | | | | *CN-FNG.chr1.338* | *CNL-FvH4.6g49940.1* | *CNL-FVI.CHR7.1051* | | | | *CNL-FMA.chr6.1184* | | | | | | | | |
| *TN-FvH4.5g07190.1* | *TNL-FII.chr5.609* | | | | *CN-FNG.chr3.2186* | *CNL-FDA.chr4.11333* | *CNL-FVI.CHR7.1051* | | | | *CNL-FNG.chr7.951* | | | | | | | | |
| *TN-FvH4.5g07190.1* | *TNL-FMA.chr5.364* | | | | *CN-FNG.chr3.2186* | *CNL-FII.chr4.377* | *CNL-FVI.CHR7.1051* | | | | *CNL-FNU.ctg132.293* | | | | | | | | |
| *TN-FvH4.5g32990.1* | *TNL-FDA.chr5.03488* | | | | *CN-FNG.chr3.2186* | *CNL-FvH4.4g06030.1* | *CNL-FVI.CHR7.1051* | | | | *CNL-FvH4.6g12200.1* | | | | | | | | |
| *TN-FvH4.5g32990.1* | *TNL-FNU.ctg160.483* | | | | *CN-FNG.chr3.2311* | *NL-FDA.chr6.02338* | *CNL-FVI.CHR7.1336* | | | | *CNL-FII.chr7.789* | | | | | | | | |
| *TN-FvH4.5g34230.1* | *TNL-FDA.chr5.03487* | | | | *CN-FNG.chr4.2180* | *CNL-FII.chr4.2076* | *CNL-FVI.CHR7.1336* | | | | *CNL-FNU.ctg114.210* | | | | | | | | |
| *TN-FvH4.5g34230.1* | *TNL-FII.chr5.2970* | | | | *CN-FNG.chr4.2180* | *CNL-FMA.chr4.2118* | *CNL-FVI.CHR7.1336* | | | | *CNL-FPE.chr7.1124* | | | | | | | | |
| *TN-FvH4.5g34240.1* | *TN-FDA.chr5.03486* | | | | *CN-FNG.chr4.2180* | *CNL-FNU.ctg82.313* | *CNL-FVI.CHR7.1336* | | | | *CNL-FvH4.7g15240.1* | | | | | | | | |
| *TN-FvH4.5g34240.1* | *TNL-FII.chr5.2969* | | | | *CN-FNG.chr4.2180* | *CNL-FPE.chr3.1482* | *CNL-FVI.CHR7.171* | | | | *CNL-FII.chr7.162.3* | | | | | | | | |
| *TN-FvH4.6g01300.1* | *TN-FDA.chr3.05844* | | | | *CN-FNG.chr4.2180* | *CNL-FvH4.4g25750.1* | *CNL-FVI.CHR7.171* | | | | *CNL-FNG.chr7.204* | | | | | | | | |
| *TN-FvH4.6g39860.1* | *TNL-FNU.ctg162.30* | | | | *CN-FNG.chr4.2529* | *CNL-FII.chr7.998* | *CNL-FVI.CHR7.171* | | | | *CNL-FNU.ctg146.100* | | | | | | | | |
| *TN-FvH4.6g49490.1* | *TN-FDA.chr2.08147* | | | | *CN-FNG.chr4.2529* | *CNL-FMA.chr7.880* | *CNL-FVI.CHR7.171* | | | | *CNL-FvH4.7g01950.1* | | | | | | | | |
| *TN-FvH4.6g49490.1* | *TNL-FNU.ctg160.481* | | | | *CN-FNG.chr4.2529* | *CNL-FNU.ctg81.894* | *CNL-FVI.CHR7.2604* | | | | *CN-FvH4.7g31270.1* | | | | | | | | |
| *TN-FvH4.6g49500.1* | *TN-FDA.chr2.08147* | | | | *CN-FNG.chr4.2529* | *CNL-FPE.chr3.4782* | *CNL-FVI.CHR7.2604* | | | | *CNL-FDA.chr6.02010* | | | | | | | | |
| *TN-FvH4.6g49500.1* | *TN-FII.chr6.4140* | | | | *CN-FNG.chr4.2529* | *CNL-FvH4.4g29930.1* | *CNL-FVI.CHR7.264* | | | | *CNL-FII.chr7.257* | | | | | | | | |
| *TN-FvH4.7g11160.1* | *TNL-FNU.ctg129.394* | | | | *CN-FNG.chr5.2228* | *CNL-FII.chr4.1808* | *CNL-FVI.CHR7.264* | | | | *CNL-FNG.chr7.204* | | | | | | | | |
| Orthologous gene pairsof TNL | | | | | Orthologous gene pairs of non-TNL | | | | | | | | | | | | | | |
| ID of sequence 1 | ID of sequence 2 | | | | ID of sequence 1 | ID of sequence 2 | ID of sequence 1 | | | | | | | | ID of sequence 2 | | | | |
| *TN-FvH4.7g11580.1* | *TNL-FII.chr6.136* | | | | *CN-FNG.chr5.2228* | *CNL-FNU.ctg158.149* | *CNL-FVI.CHR7.264* | | | | *CNL-FNU.ctg145.8* | | | | | | | | |
| *TN-FvH4.7g11580.1* | *TNL-FNU.ctg129.394* | | | | *CN-FNG.chr5.2228* | *CNL-FPE.chr7.1124* | *CNL-FVI.CHR7.264* | | | | *CNL-FPE.chr6.1639* | | | | | | | | |
| *TN-FvH4.7g28520.1* | *TN-FII.chr7.977* | | | | *CN-FNG.chr5.2228* | *N-FvH4.5g24320.1* | *CNL-FVI.CHR7.264* | | | | *CNL-FvH4.7g02890.1* | | | | | | | | |
| *TN-FvH4.7g28520.1* | *TNL-FDA.chr7.01621* | | | | *CN-FNG.chr5.2692* | *CN-FNU.ctg161.301* | *CNL-FVI.CHR7.2923* | | | | *CNL-FDA.chr3.23062* | | | | | | | | |
| *TN-FvH4.7g32720.1* | *TN-FDA.chr7.00120* | | | | *CN-FNG.chr5.2692* | *CNL-FvH4.4g06020.1* | *CNL-FVI.CHR7.2923* | | | | *CNL-FII.chr3.77* | | | | | | | | |
| *TN-FVI.CHR1.2577* | *TN-FII.chr1.2339* | | | | *CN-FNG.chr5.2696* | *CN-FII.chr6.2548* | *CNL-FVI.CHR7.2923* | | | | *CNL-FMA.chr3.75* | | | | | | | | |
| *TN-FVI.CHR1.2577* | *TN-FNG.chr1.1802* | | | | *CN-FNG.chr5.2696* | *CN-FNU.ctg161.296* | *CNL-FVI.CHR7.2923* | | | | *CNL-FNG.chr3.84* | | | | | | | | |
| *TN-FVI.CHR1.2577* | *TN-FNU.ctg177.55* | | | | *CN-FNG.chr5.2696* | *CN-FPE.chr5.3016* | *CNL-FVI.CHR7.2923* | | | | *CNL-FNU.ctg47.220* | | | | | | | | |
| *TN-FVI.CHR1.2577* | *TN-FvH4.1g27020.1* | | | | *CN-FNG.chr5.2696* | *CN-FvH4.6g29230.1* | *CNL-FVI.CHR7.2923* | | | | *CNL-FvH4.3g00820.1* | | | | | | | | |
| *TN-FVI.CHR4.939* | *TNL-FDA.chr1.17976* | | | | *CN-FNG.chr6.1541* | *CN-FNU.ctg71.38* | *CNL-FVI.CHR7.2926* | | | | *CNL-FDA.chr3.23062* | | | | | | | | |
| *TN-FVI.CHR4.939* | *TNL-FII.chr1.1393* | | | | *CN-FNG.chr6.1541* | *CN-FPE.chr0.885* | *CNL-FVI.CHR7.2926* | | | | *CNL-FII.chr3.77* | | | | | | | | |
| *TN-FVI.CHR4.939* | *TNL-FNG.chr1.1446* | | | | *CN-FNG.chr6.1541* | *CN-FvH4.6g22150.1* | *CNL-FVI.CHR7.2926* | | | | *CNL-FMA.chr3.75* | | | | | | | | |
| *TN-FVI.CHR4.939* | *TNL-FNU.ctg172.194* | | | | *CN-FNG.chr6.1594* | *CN-FNU.ctg65.105* | *CNL-FVI.CHR7.2926* | | | | *CNL-FNG.chr3.84* | | | | | | | | |
| *TN-FVI.CHR4.939* | *TNL-FvH4.1g16600.1* | | | | *CN-FNG.chr6.1594* | *CN-FPE.chr6.1820* | *CNL-FVI.CHR7.2926* | | | | *CNL-FNU.ctg47.220* | | | | | | | | |
| *TN-FVI.CHR5.1806* | *TNL-FDA.chr5.25283* | | | | *CN-FNG.chr6.1594* | *CN-FvH4.6g29230.1* | *CNL-FVI.CHR7.2926* | | | | *CNL-FvH4.3g00820.1* | | | | | | | | |
| *TN-FVI.CHR5.1806* | *TNL-FII.chr5.1800* | | | | *CN-FNG.chr6.3269* | *CN-FMA.chr6.2632* | *CNL-FVI.CHR7.3757* | | | | *CNL-FII.chr3.951* | | | | | | | | |
| *TN-FVI.CHR5.1806* | *TNL-FNU.ctg160.483* | | | | *CN-FNG.chr6.3269* | *CN-FNU.ctg57.114* | *CNL-FVI.CHR7.3757* | | | | *CNL-FvH4.3g11470.1* | | | | | | | | |
| *TN-FVI.CHR5.1806* | *TNL-FvH4.5g32970.1* | | | | *CN-FNG.chr6.3269* | *CN-FPE.chr6.3007* | *CNL-FVI.CHR7.3757* | | | | *N-FPE.chr3.1891* | | | | | | | | |
| *TN-FVI.CHR5.2927* | *TN-FII.chr5.2971* | | | | *CN-FNG.chr6.3269* | *CN-FvH4.6g29230.1* | *CNL-FVI.CHR7.3758* | | | | *CNL-FDA.chr3.15906* | | | | | | | | |
| *TN-FVI.CHR5.2927* | *TNL-FDA.chr5.03488* | | | | *CN-FNG.chr7.813* | *CN-FPE.chr4.2668* | *CNL-FVI.CHR7.3758* | | | | *CNL-FII.chr3.953* | | | | | | | | |
| *TN-FVI.CHR5.2927* | *TNL-FvH4.3g45380.1* | | | | *CN-FNG.chr7.813* | *CNL-FII.chr7.789* | *CNL-FVI.CHR7.3758* | | | | *CNL-FvH4.3g11490.1* | | | | | | | | |
| *TN-FVI.CHR7.995* | *TN-FII.chr7.977* | | | | *CN-FNG.chr7.813* | *CNL-FNU.ctg114.210* | *CNL-FVI.CHR7.3762* | | | | *CNL-FDA.chr3.15906* | | | | | | | | |
| *TN-FVI.CHR7.995* | *TNL-FDA.chr7.02890* | | | | *CN-FNG.chr7.813* | *CNL-FvH4.7g08870.1* | *CNL-FVI.CHR7.3762* | | | | *CNL-FII.chr3.954* | | | | | | | | |
| *TN-FVI.CHR7.995* | *TNL-FNU.ctg129.394* | | | | *CN-FNU.ctg104.161* | *CNL-FNG.chr2.416* | *CNL-FVI.CHR7.3762* | | | | *CNL-FvH4.3g11490.1* | | | | | | | | |
| *TNL-FDA.chr1.04275* | *TNL-FMA.chr0.565* | | | | *CN-FNU.ctg104.161* | *CNL-FPE.chr7.1798* | *CNL-FVI.CHR7.810* | | | | *CN-FNG.chr7.813* | | | | | | | | |
| *TNL-FDA.chr1.04275* | *TNL-FNU.ctg173.410* | | | | *CN-FNU.ctg144.27* | *CNL-FII.chr7.257* | *CNL-FVI.CHR7.810* | | | | *CNL-FII.chr7.789* | | | | | | | | |
| *TNL-FDA.chr1.04275* | *TNL-FvH4.1g22380.1* | | | | *CN-FNU.ctg144.27* | *CNL-FNG.chr7.204* | *CNL-FVI.CHR7.810* | | | | *CNL-FNU.ctg114.210* | | | | | | | | |
| *TNL-FDA.chr1.07161* | *TNL-FMA.chr0.565* | | | | *CN-FNU.ctg144.27* | *CNL-FPE.chr6.1639* | *CNL-FVI.CHR7.810* | | | | *CNL-FvH4.7g08870.1* | | | | | | | | |
| *TNL-FDA.chr1.07161* | *TNL-FNU.ctg173.410* | | | | *CN-FNU.ctg144.27* | *CNL-FvH4.7g02890.1* | *CNL-FVI.CHR7.810* | | | | *NL-FPE.chr0.1234* | | | | | | | | |
| *TNL-FDA.chr1.07161* | *TNL-FvH4.1g07020.1* | | | | *CN-FNU.ctg158.39* | *CN-FPE.chr4.1617* | *N-FDA.chr1.18128* | | | | *CN-FvH4.1g18160.1* | | | | | | | | |
| *TNL-FDA.chr1.15663* | *TNL-FII.chr1.757* | | | | *CN-FNU.ctg158.39* | *CNL-FDA.chr5.25050* | *N-FDA.chr1.18128* | | | | *CNL-FNG.chr5.1760* | | | | | | | | |
| *TNL-FDA.chr1.15663* | *TNL-FPE.chr6.1904* | | | | *CN-FNU.ctg158.39* | *CNL-FII.chr5.2001* | *N-FDA.chr1.18128* | | | | *NL-FNU.ctg155.6* | | | | | | | | |
| *TNL-FDA.chr1.15663* | *TNL-FvH4.3g10020.1* | | | | *CN-FNU.ctg158.39* | *CNL-FNG.chr5.2145* | *N-FDA.chr1.22180* | | | | *RN-FPE.chr6.6309* | | | | | | | | |
| *TNL-FDA.chr1.17976* | *TNL-FII.chr1.1393* | | | | *CN-FNU.ctg158.39* | *CNL-FvH4.5g23390.1* | *N-FDA.chr1.22180* | | | | *RN-FvH4.1g15140.1* | | | | | | | | |
| *TNL-FDA.chr1.17976* | *TNL-FNG.chr1.1446* | | | | *CN-FNU.ctg161.296* | *CN-FII.chr6.2548* | *N-FDA.chr1.22180* | | | | *RNL-FII.chr1.1269* | | | | | | | | |
| *TNL-FDA.chr1.17976* | *TNL-FNU.ctg172.194* | | | | *CN-FNU.ctg161.296* | *CN-FMA.chr6.2632* | *N-FDA.chr1.22180* | | | | *RNL-FMA.chr1.1806* | | | | | | | | |
| *TNL-FDA.chr1.17976* | *TNL-FvH4.1g16600.1* | | | | *CN-FNU.ctg161.296* | *CN-FvH4.6g29230.1* | *N-FDA.chr2.08227* | | | | *CNL-FII.chr2.1235* | | | | | | | | |
| *TNL-FDA.chr1.17978* | *TNL-FII.chr1.1393* | | | | *CN-FNU.ctg161.301* | *CNL-FDA.chr5.22369* | *N-FDA.chr2.08227* | | | | *CNL-FvH4.2g14930.1* | | | | | | | | |
| *TNL-FDA.chr1.17978* | *TNL-FNG.chr1.1446* | | | | *CN-FNU.ctg161.301* | *CNL-FMA.chr5.2388* | *N-FDA.chr2.11387* | | | | *RN-FNU.ctg109.400* | | | | | | | | |
| *TNL-FDA.chr1.17978* | *TNL-FNU.ctg172.194* | | | | *CN-FNU.ctg161.301* | *CNL-FvH4.4g06030.1* | *N-FDA.chr2.11387* | | | | *RNL-FII.chr2.1682* | | | | | | | | |
| *TNL-FDA.chr1.17978* | *TNL-FvH4.1g16600.1* | | | | *CN-FNU.ctg20.194* | *CNL-FMA.chr3.2744* | *N-FDA.chr2.11387* | | | | *RNL-FMA.chr2.1492* | | | | | | | | |
| *TNL-FDA.chr1.17980* | *TNL-FII.chr1.1394* | | | | *CN-FNU.ctg20.194* | *CNL-FPE.chr7.1015* | *N-FDA.chr2.11387* | | | | *RNL-FNG.chr2.1787* | | | | | | | | |
| Orthologous gene pairsof TNL | | | | | Orthologous gene pairs of non-TNL | | | | | | | | | | | | | | |
| ID of sequence 1 | ID of sequence 2 | | | | ID of sequence 1 | ID of sequence 2 | ID of sequence 1 | | | | | | | | ID of sequence 2 | | | | |
| *TNL-FDA.chr1.17980* | *TNL-FNU.ctg172.195* | | | | *CN-FNU.ctg20.194* | *CNL-FvH4.7g11380.1* | | *N-FDA.chr2.11387* | | | | *RNL-FPE.chr5.2061* | | | | | | | | |
| *TNL-FDA.chr1.17980* | *TNL-FvH4.1g16620.1* | | | | *CN-FNU.ctg53.159* | *CN-FDA.chr6.13604* | | *N-FDA.chr2.11533* | | | | *CN-FII.chr6.198* | | | | | | | | |
| *TNL-FDA.chr1.17981* | *TNL-FII.chr1.1394* | | | | *CN-FNU.ctg53.159* | *CNL-FvH4.6g47980.1* | | *N-FDA.chr2.11533* | | | | *CNL-FNG.chr2.1430* | | | | | | | | |
| *TNL-FDA.chr1.17981* | *TNL-FNU.ctg172.196* | | | | *CN-FNU.ctg53.173* | *CNL-FDA.chr6.13561* | | *N-FDA.chr2.11533* | | | | *CNL-FvH4.4g35200.1* | | | | | | | | |
| *TNL-FDA.chr1.17981* | *TNL-FvH4.1g16650.1* | | | | *CN-FNU.ctg53.173* | *CNL-FvH4.6g47980.1* | | *N-FDA.chr2.12211* | | | | *CNL-FvH4.2g36810.1* | | | | | | | | |
| *TNL-FDA.chr2.10226* | *TNL-FII.chr2.708* | | | | *CN-FNU.ctg54.29* | *CNL-FDA.chr6.13561* | | *N-FDA.chr3.06067* | | | | *N-FMA.chr3.3384* | | | | | | | | |
| *TNL-FDA.chr2.10226* | *TNL-FMA.chr5.2877* | | | | *CN-FNU.ctg54.29* | *CNL-FvH4.6g47050.1* | | *N-FDA.chr3.06067* | | | | *N-FNG.chr3.3429* | | | | | | | | |
| *TNL-FDA.chr2.10226* | *TNL-FNU.ctg105.95* | | | | *CN-FNU.ctg57.114* | *CN-FMA.chr6.2632* | | *N-FDA.chr3.06067* | | | | *N-FPE.chr6.572* | | | | | | | | |
| *TNL-FDA.chr2.10226* | *TNL-FvH4.5g07310.1* | | | | *CN-FNU.ctg57.114* | *CN-FvH4.6g29230.1* | | *N-FDA.chr3.06067* | | | | *N-FvH4.3g41580.1* | | | | | | | | |
| *TNL-FDA.chr2.12354* | *TNL-FII.chr2.49* | | | | *CN-FNU.ctg63.62* | *CN-FDA.chr6.17380* | | *N-FDA.chr3.20671* | | | | *CNL-FII.chr3.1811* | | | | | | | | |
| *TNL-FDA.chr2.12354* | *TNL-FPE.chr2.91* | | | | *CN-FNU.ctg63.62* | *CN-FII.chr6.2648* | | *N-FDA.chr3.20675* | | | | *N-FvH4.3g44060.1* | | | | | | | | |
| *TNL-FDA.chr2.12354* | *TNL-FvH4.7g33140.1* | | | | *CN-FNU.ctg63.62* | *CN-FMA.chr6.2734* | | *N-FDA.chr3.24469* | | | | *NL-FII.chr1.1268* | | | | | | | | |
| *TNL-FDA.chr2.12488* | *TNL-FII.chr2.172* | | | | *CN-FNU.ctg63.62* | *CN-FPE.chr6.470* | | *N-FDA.chr3.24469* | | | | *RN-FNU.ctg52.52* | | | | | | | | |
| *TNL-FDA.chr2.12488* | *TNL-FNG.chr2.105* | | | | *CN-FNU.ctg63.62* | *N-FvH4.6g30240.1* | | *N-FDA.chr3.24469* | | | | *RNL-FvH4.6g50310.1* | | | | | | | | |
| *TNL-FDA.chr2.12488* | *TNL-FPE.chr0.1078* | | | | *CN-FNU.ctg65.11* | *CNL-FvH4.6g30120.1* | | *N-FDA.chr5.07880* | | | | *CNL-FMA.chr5.1739* | | | | | | | | |
| *TNL-FDA.chr2.12488* | *TNL-FvH4.2g02050.1* | | | | *CN-FNU.ctg70.1* | *CN-FDA.chr6.20969* | | *N-FDA.chr5.07880* | | | | *CNL-FNG.chr5.1760* | | | | | | | | |
| *TNL-FDA.chr2.12488* | *TNL-FNU.ctg101.157* | | | | *CN-FNU.ctg70.1* | *CN-FII.chr6.1890* | | *N-FDA.chr5.07880* | | | | *CNL-FvH4.5g18950.1* | | | | | | | | |
| *TNL-FDA.chr2.20071* | *TNL-FMA.chr2.2281* | | | | *CN-FNU.ctg70.1* | *CN-FMA.chr6.1745* | | *N-FDA.chr5.07880* | | | | *NL-FNU.ctg155.6* | | | | | | | | |
| *TNL-FDA.chr2.20071* | *TNL-FNG.chr2.2392* | | | | *CN-FNU.ctg70.1* | *CN-FNG.chr6.1541* | | *N-FDA.chr6.17361* | | | | *CN-FII.chr6.2623* | | | | | | | | |
| *TNL-FDA.chr2.20071* | *TNL-FvH4.2g27720.1* | | | | *CN-FNU.ctg70.1* | *CN-FPE.chr0.885* | | *N-FDA.chr6.17361* | | | | *CN-FPE.chr6.447* | | | | | | | | |
| *TNL-FDA.chr2.20072* | *TNL-FMA.chr2.2280* | | | | *CN-FNU.ctg70.1* | *CN-FvH4.6g22150.1* | | *N-FDA.chr6.17361* | | | | *N-FMA.chr6.2173* | | | | | | | | |
| *TNL-FDA.chr2.20072* | *TNL-FNG.chr2.2392* | | | | *CN-FNU.ctg73.111* | *CNL-FII.chr4.1808* | | *N-FDA.chr6.17361* | | | | *N-FNU.ctg65.16* | | | | | | | | |
| *TNL-FDA.chr2.20072* | *TNL-FvH4.2g27720.1* | | | | *CN-FNU.ctg73.111* | *CNL-FvH4.4g22940.1* | | *N-FDA.chr6.17361* | | | | *N-FvH4.6g30050.1* | | | | | | | | |
| *TNL-FDA.chr2.20138* | *TNL-FMA.chr2.2218* | | | | *CN-FNU.ctg84.31* | *CNL-FMA.chr6.1508* | | *N-FDA.chr7.14603* | | | | *CNL-FII.chr3.1811* | | | | | | | | |
| *TNL-FDA.chr2.20138* | *TNL-FNG.chr2.2347* | | | | *CN-FNU.ctg84.31* | *CNL-FvH4.6g15090.1* | | *N-FDA.chr7.22742* | | | | *CNL-FII.chr3.1811* | | | | | | | | |
| *TNL-FDA.chr2.20138* | *TNL-FNU.ctg114.235* | | | | *CN-FPE.chr0.885* | *CN-FNU.ctg71.38* | | *N-FII.chr1.1277* | | | | *CNL-FNU.ctg172.55* | | | | | | | | |
| *TNL-FDA.chr2.20138* | *TNL-FPE.chr5.1409* | | | | *CN-FPE.chr0.885* | *CN-FvH4.6g22150.1* | | *N-FII.chr1.1277* | | | | *RNL-FDA.chr1.22196* | | | | | | | | |
| *TNL-FDA.chr2.20138* | *TNL-FvH4.2g27070.1* | | | | *CN-FPE.chr1.928* | *CNL-FNU.ctg82.648* | | *N-FII.chr1.1277* | | | | *RNL-FMA.chr1.1806* | | | | | | | | |
| *TNL-FDA.chr3.05636* | *TNL-FII.chr3.1814_FII.chr3.1815* | | | | *CN-FPE.chr1.928* | *CNL-FvH4.4g22940.1* | | *N-FII.chr1.1277* | | | | *RNL-FNG.chr1.1333* | | | | | | | | |
| *TNL-FDA.chr3.05636* | *TNL-FNG.chr3.2334* | | | | *CN-FPE.chr2.3023* | *CN-FvH4.6g29690.1* | | *N-FII.chr1.1277* | | | | *RNL-FvH4.1g15330.1* | | | | | | | | |
| *TNL-FDA.chr3.05639* | *TNL-FII.chr3.3626* | | | | *CN-FPE.chr2.3023* | *CNL-FII.chr3.951* | | *N-FII.chr2.1082* | | | | *CNL-FvH4.2g13150.1* | | | | | | | | |
| *TNL-FDA.chr3.05639* | *TNL-FNG.chr7.2518* | | | | *CN-FPE.chr4.1090* | *CNL-FII.chr5.2002* | | *N-FII.chr2.1082* | | | | *N-FDA.chr2.08227* | | | | | | | | |
| *TNL-FDA.chr3.05639* | *TNL-FPE.chr5.2900* | | | | *CN-FPE.chr4.1090* | *CNL-FNG.chr5.2145* | | *N-FII.chr2.1082* | | | | *N-FPE.chr2.64* | | | | | | | | |
| *TNL-FDA.chr3.05639* | *TNL-FvH4.5g27640.1* | | | | *CN-FPE.chr4.1090* | *CNL-FNU.ctg158.40* | | *N-FII.chr2.645* | | | | *N-FMA.chr2.489* | | | | | | | | |
| *TNL-FDA.chr3.05837* | *TNL-FNU.ctg3.75* | | | | *CN-FPE.chr4.1090* | *CNL-FvH4.5g23400.1* | | *N-FII.chr2.79* | | | | *CNL-FDA.chr3.15906* | | | | | | | | |
| *TNL-FDA.chr3.05837* | *TNL-FvH4.3g44000.1* | | | | *CN-FPE.chr4.1617* | *CNL-FDA.chr5.25050* | | *N-FII.chr2.79* | | | | *CNL-FvH4.2g00960.1* | | | | | | | | |
| *TNL-FDA.chr3.05838* | *TNL-FvH4.3g43940.1* | | | | *CN-FPE.chr4.1617* | *CNL-FII.chr5.2001* | | *N-FII.chr4.1428* | | | | *CN-FNU.ctg84.31* | | | | | | | | |
| *TNL-FDA.chr3.05839* | *TNL-FvH4.3g43860.1* | | | | *CN-FPE.chr4.1617* | *CNL-FNG.chr5.2145* | | *N-FII.chr4.1428* | | | | *CNL-FMA.chr6.1508* | | | | | | | | |
| *TNL-FDA.chr3.05883* | *TNL-FvH4.3g43660.1* | | | | *CN-FPE.chr4.1617* | *CNL-FNU.ctg158.40* | | *N-FII.chr4.1428* | | | | *NL-FDA.chr6.26447* | | | | | | | | |
| *TNL-FDA.chr3.05887* | *TNL-FvH4.3g43650.1* | | | | *CN-FPE.chr4.1617* | *CNL-FvH4.5g23390.1* | | *N-FII.chr4.1428* | | | | *NL-FvH4.4g18270.1* | | | | | | | | |
| *TNL-FDA.chr3.05894* | *TNL-FvH4.3g43630.1* | | | | *CN-FPE.chr4.2668* | *CNL-FNU.ctg114.210* | | *N-FII.chr5.1933* | | | | *CNL-FDA.chr5.25050* | | | | | | | | |
| *TNL-FDA.chr3.09399* | *TNL-FII.chr1.757* | | | | *CN-FPE.chr4.2668* | *CNL-FvH4.6g49940.1* | | *N-FII.chr5.1933* | | | | *CNL-FNG.chr5.2089* | | | | | | | | |
| Orthologous gene pairsof TNL | | | | | Orthologous gene pairs of non-TNL | | | | | | | | | | | | | | | |
| ID of sequence 1 | ID of sequence 2 | | | | ID of sequence 1 | ID of sequence 2 | | ID of sequence 1 | | | | | | | | | ID of sequence 2 | | | |
| *TNL-FDA.chr3.09399* | *TNL-FPE.chr6.1904* | | | | *CN-FPE.chr4.843* | *CN-FNU.ctg84.31* | *N-FII.chr5.1933* | | | | | *CNL-FNU.ctg158.42* | | | | | | | | |
| *TNL-FDA.chr3.09399* | *TNL-FvH4.3g10020.1* | | | | *CN-FPE.chr4.843* | *CNL-FMA.chr6.1508* | *N-FII.chr5.1933* | | | | | *CNL-FvH4.5g22710.1* | | | | | | | | |
| *TNL-FDA.chr3.09421 TNL-FDA.chr3.09421 TNL-FDA.chr3.09421 TNL-FDA.chr3.09421 TNL-FDA.chr3.09421 TNL-FDA.chr3.09474 TNL-FDA.chr3.09474 TNL-FDA.chr3.09474 TNL-FDA.chr3.09523 TNL-FDA.chr3.09523 TNL-FDA.chr3.09523 TNL-FDA.chr3.20669 TNL-FDA.chr3.20669 TNL-FDA.chr3.20669 TNL-FDA.chr3.20670 TNL-FDA.chr3.20670 TNL-FDA.chr3.20674 TNL-FDA.chr3.20674 TNL-FDA.chr3.20674 TNL-FDA.chr3.20677 TNL-FDA.chr3.20677 TNL-FDA.chr3.20677* | *TNL-FII.chr5.302.2 TNL-FMA.chr5.364 TNL-FNU.ctg105.95 TNL-FPE.chr3.552 TNL-FvH4.5g07310.1 TNL-FII.chr5.302.2 TNL-FMA.chr5.364 TNL-FvH4.5g07310.1 TNL-FII.chr3.744.2 TNL-FPE.chr6.1904 TNL-FvH4.4g13420.1 TNL-FNG.chr7.2518 TNL-FNU.ctg3.73 TNL-FvH4.3g44030.1 TNL-FNU.ctg3.77 TNL-FvH4.3g44000.1 TNL-FNG.chr7.2518 TNL-FNU.ctg3.77 TNL-FvH4.3g44050.1 TNL-FNG.chr7.2518 TNL-FNU.ctg3.73 TNL-FvH4.3g44030.1* | | | | *CN-FPE.chr4.843 CN-FPE.chr4.843 CN-FPE.chr5.3016 CN-FPE.chr5.3016 CN-FPE.chr5.3016 CN-FPE.chr5.3016 CN-FPE.chr6.1521 CN-FPE.chr6.1521 CN-FPE.chr6.1521 CN-FPE.chr6.1611 CN-FPE.chr6.1611 CN-FPE.chr6.1611 CN-FPE.chr6.1611 CN-FPE.chr6.1820 CN-FPE.chr6.1820 CN-FPE.chr6.3007 CN-FPE.chr6.3007 CN-FPE.chr6.3007 CN-FPE.chr6.3765 CN-FPE.chr6.3765 CN-FPE.chr6.3765 CN-FPE.chr6.447* | *CNL-FNG.chr6.2406 CNL-FvH4.6g15090.1 CN-FII.chr6.2548 CN-FMA.chr6.2632 CN-FNU.ctg161.296 CN-FvH4.6g29230.1 CN-FNU.ctg73.111 CNL-FII.chr4.1808 CNL-FvH4.4g22940.1 CNL-FDA.chr6.26146 CNL-FMA.chr6.1658 CNL-FNU.ctg78.2 CNL-FvH4.6g15340.1 CN-FNU.ctg65.105 CN-FvH4.6g29230.1 CN-FMA.chr6.2632 CN-FNU.ctg57.114 CN-FvH4.6g29230.1 CNL-FMA.chr6.1184 CNL-FNU.ctg132.293 CNL-FvH4.6g12200.1 CN-FII.chr6.2623* | *N-FII.chr6.1009 N-FII.chr6.1009 N-FII.chr6.1009 N-FII.chr6.1009 N-FII.chr6.1009 N-FII.chr6.1246 N-FII.chr6.1246 N-FII.chr6.1246 N-FII.chr6.1246 N-FII.chr6.1246 N-FII.chr6.2591 N-FII.chr6.2591 N-FII.chr6.2592 N-FII.chr6.2592 N-FII.chr6.2592 N-FII.chr7.1290 N-FII.chr7.1290 N-FII.chr7.209 N-FII.chr7.209 N-FII.chr7.209 N-FMA.chr2.1406 N-FMA.chr2.1406* | | | | | *CN-FPE.chr6.1611 CNL-FDA.chr6.26146 CNL-FMA.chr6.1658 CNL-FNU.ctg78.2 CNL-FvH4.6g15340.1 CN-FNU.ctg84.31 CNL-FMA.chr6.1508 CNL-FNG.chr6.2406 CNL-FvH4.6g15090.1 NL-FDA.chr6.26447 CNL-FPE.chr6.2574 CNL-FvH4.3g11470.1 CN-FPE.chr2.3023 CN-FvH4.6g29690.1 N-FDA.chr2.08227 CNL-FPE.chr7.1124 CNL-FvH4.7g15240.1 CN-FMA.chr0.199 CNL-FPE.chr7.237 CNL-FvH4.7g02400.1 N-FNG.chr2.1425 N-FvH4.2g17340.1* | | | | | | | | |
| *TNL-FDA.chr3.20678* | *TNL-FNG.chr7.2518* | | | | *CN-FPE.chr6.447* | *CN-FMA.chr6.2719* | *N-FMA.chr2.311* | | | | | *CN-FvH4.2g05600.1* | | | | | | | | |
| *TNL-FDA.chr3.20678* | *TNL-FNU.ctg3.77* | | | | *CN-FPE.chr6.447* | *N-FNU.ctg65.16* | *N-FMA.chr2.311* | | | | | *CNL-FNG.chr2.416* | | | | | | | | |
| *TNL-FDA.chr3.20678* | *TNL-FvH4.3g44050.1* | | | | *CN-FPE.chr6.453* | *CN-FNU.ctg65.11* | *N-FMA.chr2.311* | | | | | *CNL-FNU.ctg104.183* | | | | | | | | |
| *TNL-FDA.chr3.20719* | *TNL-FNU.ctg3.22* | | | | *CN-FPE.chr6.453* | *CNL-FvH4.6g30120.1* | *N-FMA.chr2.311* | | | | | *CNL-FPE.chr7.1798* | | | | | | | | |
| *TNL-FDA.chr3.20719* | *TNL-FvH4.3g44390.1* | | | | *CN-FPE.chr6.470* | *CN-FNU.ctg64.1* | *N-FMA.chr2.311* | | | | | *NL-FDA.chr2.17949* | | | | | | | | |
| *TNL-FDA.chr3.20725* | *TNL-FNU.ctg105.95* | | | | *CN-FPE.chr6.470* | *N-FvH4.6g30240.1* | *N-FMA.chr2.311* | | | | | *NL-FII.chr2.452* | | | | | | | | |
| *TNL-FDA.chr3.20725* | *TNL-FvH4.3g44390.1* | | | | *CN-FPE.chr6.878* | *CNL-FNG.chr4.1917* | *N-FMA.chr3.3384* | | | | | *N-FNG.chr3.3429* | | | | | | | | |
| *TNL-FDA.chr3.20747* | *TNL-FMA.chr3.3484* | | | | *CN-FPE.chr6.878* | *CNL-FNU.ctg82.648* | *N-FMA.chr3.3384* | | | | | *N-FPE.chr6.572* | | | | | | | | |
| *TNL-FDA.chr3.20747* | *TNL-FvH4.3g44670.1* | | | | *CN-FPE.chr6.878* | *CNL-FvH4.4g22940.1* | *N-FMA.chr3.3384* | | | | | *N-FvH4.3g41580.1* | | | | | | | | |
| *TNL-FDA.chr4.10670* | *TNL-FII.chr3.3419* | | | | *CN-FPE.chr7.1432* | *CNL-FDA.chr6.02010* | *N-FMA.chr4.1879* | | | | | *NL-FvH4.4g21900.1* | | | | | | | | |
| *TNL-FDA.chr4.10670* | *TNL-FPE.chr6.1904* | | | | *CN-FPE.chr7.1432* | *CNL-FvH4.6g51610.1* | *N-FMA.chr4.2247* | | | | | *CN-FPE.chr6.878* | | | | | | | | |
| *TNL-FDA.chr4.10670* | *TNL-FvH4.4g13420.1* | | | | *CN-FPE.chr7.2093* | *CN-FII.chr6.2548* | *N-FMA.chr4.2247* | | | | | *CNL-FII.chr4.1849.2* | | | | | | | | |
| *TNL-FDA.chr4.10758* | *TNL-FNU.ctg3.77* | | | | *CN-FPE.chr7.2093* | *CN-FMA.chr6.2632* | *N-FMA.chr4.2247* | | | | | *CNL-FNG.chr4.1917* | | | | | | | | |
| *TNL-FDA.chr4.10758* | *TNL-FvH4.3g43960.1* | | | | *CN-FPE.chr7.2093* | *CN-FNG.chr5.2696* | *N-FMA.chr4.2247* | | | | | *CNL-FNU.ctg82.648* | | | | | | | | |
| *TNL-FDA.chr5.03487* | *TNL-FII.chr5.2970* | | | | *CN-FPE.chr7.2093* | *CN-FNU.ctg161.296* | *N-FMA.chr4.2247* | | | | | *N-FvH4.4g23400.1* | | | | | | | | |
| *TNL-FDA.chr5.03487* | *TNL-FNU.ctg160.483* | | | | *CN-FPE.chr7.2093* | *CN-FvH4.6g29230.1* | *N-FMA.chr5.2639* | | | | | *CNL-FII.chr5.3003* | | | | | | | | |
| *TNL-FDA.chr5.03487* | *TNL-FvH4.5g34210.1* | | | | *CN-FPE.chr7.728* | *CN-FvH4.2g06550.1* | *N-FMA.chr5.2639* | | | | | *CNL-FNG.chr5.2999* | | | | | | | | |
| *TNL-FDA.chr5.03488* | *TNL-FII.chr5.2969* | | | | *CN-FPE.chr7.728* | *CNL-FNU.ctg104.299* | *N-FMA.chr5.2639* | | | | | *CNL-FPE.chr1.2047* | | | | | | | | |
| Orthologous gene pairsof TNL | | | | | Orthologous gene pairs of non-TNL | | | | | | | | | | | | | | |
| ID of sequence 1 | ID of sequence 2 | | | | ID of sequence 1 | ID of sequence 2 | ID of sequence 1 | | | | | | | | ID of sequence 2 | | | | |
| *TNL-FDA.chr5.03488* | *TNL-FvH4.5g34210.1* | | | | *CN-FvH4.1g02740.1* | *CN-FII.chr6.198* | *N-FMA.chr5.2639* | | | | | *CNL-FvH4.5g34680.1* | | | | | | | | |
| *TNL-FDA.chr5.03613* | *TNL-FII.chr5.2851* | | | | *CN-FvH4.1g02740.1* | *CNL-FDA.chr6.02198* | *N-FMA.chr5.2639* | | | | | *N-FNU.ctg161.240* | | | | | | | | |
| *TNL-FDA.chr5.03613* | *TNL-FNU.ctg160.481* | | | | *CN-FvH4.1g02740.1* | *CNL-FNG.chr2.1430* | *N-FMA.chr6.2154* | | | | | *CN-FDA.chr6.17380* | | | | | | | | |
| *TNL-FDA.chr5.03613* | *TNL-FvH4.5g32970.1* | | | | *CN-FvH4.1g18160.1* | *CNL-FNG.chr5.1760* | *N-FMA.chr6.2154* | | | | | *CN-FII.chr6.2648* | | | | | | | | |
| *TNL-FDA.chr5.03707* | *TNL-FII.chr5.2851* | | | | *CN-FvH4.1g18160.1* | *NL-FNU.ctg155.6* | *N-FMA.chr6.2154* | | | | | *CN-FNU.ctg64.1* | | | | | | | | |
| *TNL-FDA.chr5.03707* | *TNL-FNU.ctg160.483* | | | | *CN-FvH4.2g05600.1* | *CN-FNU.ctg104.161* | *N-FMA.chr6.2154* | | | | | *CN-FPE.chr6.470* | | | | | | | | |
| *TNL-FDA.chr5.03707* | *TNL-FvH4.5g32050.1* | | | | *CN-FvH4.2g06550.1* | *CNL-FNU.ctg104.299* | *N-FMA.chr6.2154* | | | | | *N-FvH4.6g30240.1* | | | | | | | | |
| *TNL-FDA.chr5.12803* | *TNL-FII.chr5.3330* | | | | *CN-FvH4.2g06640.1* | *CNL-FPE.chr2.767* | *N-FMA.chr6.2173* | | | | | *CN-FII.chr6.2623* | | | | | | | | |
| *TNL-FDA.chr5.12803* | *TNL-FNG.chr2.105* | | | | *CN-FvH4.3g16720.1* | *CNL-FNU.ctg165.171* | *N-FMA.chr6.2173* | | | | | *CN-FPE.chr6.447* | | | | | | | | |
| *TNL-FDA.chr5.12803* | *TNL-FvH4.5g38680.1* | | | | *CN-FvH4.3g21750.1* | *CNL-FNG.chr3.2531* | *N-FMA.chr6.2173* | | | | | *N-FNU.ctg65.16* | | | | | | | | |
| *TNL-FDA.chr5.12815* | *TNL-FII.chr5.3343* | | | | *CN-FvH4.3g21750.1* | *CNL-FNU.ctg21.293* | *N-FMA.chr6.2173* | | | | | *N-FvH4.6g30050.1* | | | | | | | | |
| *TNL-FDA.chr5.12815* | *TNL-FMA.chr5.2877* | | | | *CN-FvH4.5g23460.1* | *CNL-FII.chr5.2007* | *N-FNG.chr1.1565* | | | | | *CN-FII.chr1.1528* | | | | | | | | |
| *TNL-FDA.chr5.12815* | *TNL-FvH4.5g38870.1* | | | | *CN-FvH4.5g23460.1* | *CNL-FNG.chr5.2149* | *N-FNG.chr1.1565* | | | | | *CNL-FMA.chr4.679* | | | | | | | | |
| *TNL-FDA.chr5.23994* | *TNL-FII.chr5.302.2* | | | | *CN-FvH4.5g23460.1* | *CNL-FNU.ctg158.46* | *N-FNG.chr1.1565* | | | | | *CNL-FvH4.4g06020.1* | | | | | | | | |
| *TNL-FDA.chr5.23994* | *TNL-FMA.chr5.364* | | | | *CN-FvH4.5g24290.1* | *CNL-FDA.chr5.24966* | *N-FNG.chr1.1565* | | | | | *N-FDA.chr1.18128* | | | | | | | | |
| *TNL-FDA.chr5.23994* | *TNL-FvH4.5g03630.1* | | | | *CN-FvH4.5g24290.1* | *CNL-FII.chr5.2008* | *N-FNG.chr1.426* | | | | | *CNL-FII.chr3.1385* | | | | | | | | |
| *TNL-FDA.chr5.25282* | *TNL-FII.chr5.2851* | | | | *CN-FvH4.5g24290.1* | *CNL-FNG.chr5.2227* | *N-FNG.chr1.426* | | | | | *CNL-FNU.ctg30.47* | | | | | | | | |
| *TNL-FDA.chr5.25282* | *TNL-FNU.ctg160.483* | | | | *CN-FvH4.5g24290.1* | *CNL-FNU.ctg158.148* | *N-FNG.chr1.426* | | | | | *CNL-FPE.chr5.124* | | | | | | | | |
| *TNL-FDA.chr5.25282* | *TNL-FvH4.5g32970.1* | | | | *CN-FvH4.5g24310.1* | *CN-FNG.chr5.2228* | *N-FNG.chr2.1425* | | | | | *N-FvH4.2g17340.1* | | | | | | | | |
| *TNL-FDA.chr5.25283* | *TNL-FII.chr5.1800* | | | | *CN-FvH4.5g24310.1* | *CNL-FII.chr4.1808* | *N-FNG.chr2.1856* | | | | | *N-FDA.chr2.11387* | | | | | | | | |
| *TNL-FDA.chr5.25283* | *TNL-FNU.ctg160.481* | | | | *CN-FvH4.5g24310.1* | *CNL-FNU.ctg158.149* | *N-FNG.chr2.1856* | | | | | *RN-FNU.ctg109.400* | | | | | | | | |
| *TNL-FDA.chr5.25283* | *TNL-FvH4.5g32970.1* | | | | *CN-FvH4.5g24310.1* | *CNL-FPE.chr3.2618* | *N-FNG.chr2.1856* | | | | | *RNL-FII.chr2.1682* | | | | | | | | |
| *TNL-FDA.chr6.02345* | *TNL-FII.chr6.322* | | | | *CN-FvH4.6g02790.1* | *CN-FII.chr6.198* | *N-FNG.chr2.1856* | | | | | *RNL-FMA.chr2.1492* | | | | | | | | |
| *TNL-FDA.chr6.02345* | *TNL-FNU.ctg8.247* | | | | *CN-FvH4.6g02790.1* | *CNL-FDA.chr6.02198* | *N-FNG.chr2.1856* | | | | | *RNL-FPE.chr5.2061* | | | | | | | | |
| *TNL-FDA.chr6.02345* | *TNL-FvH4.6g05610.1* | | | | *CN-FvH4.6g02790.1* | *CNL-FNG.chr2.1430* | *N-FNG.chr3.2988* | | | | | *CN-FII.chr3.2661* | | | | | | | | |
| *TNL-FDA.chr6.02361* | *TNL-FII.chr6.322* | | | | *CN-FvH4.6g15230.1* | *CN-FII.chr6.1267* | *N-FNG.chr3.2988* | | | | | *CN-FNU.ctg20.194* | | | | | | | | |
| *TNL-FDA.chr6.02361* | *TNL-FNU.ctg8.247* | | | | *CN-FvH4.6g15230.1* | *CNL-FDA.chr6.26153* | *N-FNG.chr3.2988* | | | | | *CNL-FDA.chr1.06545* | | | | | | | | |
| *TNL-FDA.chr6.02361* | *TNL-FvH4.6g04730.1* | | | | *CN-FvH4.6g15230.1* | *CNL-FMA.chr6.1184* | *N-FNG.chr3.2988* | | | | | *CNL-FPE.chr7.1015* | | | | | | | | |
| *TNL-FDA.chr6.02439* | *TNL-FII.chr3.3626* | | | | *CN-FvH4.6g15230.1* | *CNL-FNG.chr6.2420* | *N-FNG.chr3.3429* | | | | | *N-FPE.chr6.572* | | | | | | | | |
| *TNL-FDA.chr6.02439* | *TNL-FNG.chr7.2518* | | | | *CN-FvH4.6g15230.1* | *CNL-FNU.ctg78.2* | *N-FNG.chr3.3429* | | | | | *N-FvH4.3g41580.1* | | | | | | | | |
| *TNL-FDA.chr6.02439* | *TNL-FNU.ctg3.75* | | | | *CN-FvH4.6g22150.1* | *CN-FNU.ctg71.38* | *N-FNG.chr4.896* | | | | | *CNL-FII.chr4.1808* | | | | | | | | |
| *TNL-FDA.chr6.02439* | *TNL-FPE.chr5.2900* | | | | *CN-FvH4.6g29230.1* | *CN-FNU.ctg65.105* | *N-FNG.chr4.896* | | | | | *CNL-FNU.ctg82.648* | | | | | | | | |
| *TNL-FDA.chr6.02439* | *TNL-FvH4.5g27640.1* | | | | *CN-FvH4.6g29690.1* | *CNL-FII.chr3.951* | *N-FNG.chr4.896* | | | | | *CNL-FvH4.7g33440.1* | | | | | | | | |
| *TNL-FDA.chr6.13989* | *TNL-FNU.ctg3.77* | | | | *CN-FvH4.6g29690.1* | *CNL-FPE.chr5.3277* | *N-FNG.chr4.896* | | | | | *NL-FPE.chr3.3367* | | | | | | | | |
| *TNL-FDA.chr6.13989* | *TNL-FvH4.7g31510.1* | | | | *CN-FvH4.7g02430.1* | *CN-FMA.chr0.199* | *N-FNG.chr6.1058* | | | | | *CN-FPE.chr6.1611* | | | | | | | | |
| *TNL-FDA.chr6.16370* | *TNL-FII.chr1.1394* | | | | *CN-FvH4.7g02430.1* | *CNL-FII.chr7.207* | *N-FNG.chr6.1058* | | | | | *CNL-FDA.chr6.26337* | | | | | | | | |
| *TNL-FDA.chr6.16370* | *TNL-FNU.ctg56.77* | | | | *CN-FvH4.7g02430.1* | *CNL-FPE.chr7.237* | *N-FNG.chr6.1058* | | | | | *CNL-FMA.chr6.1658* | | | | | | | | |
| *TNL-FDA.chr6.16370* | *TNL-FvH4.1g16620.1* | | | | *CN-FvH4.7g11820.1* | *CN-FPE.chr6.3765* | *N-FNG.chr6.1058* | | | | | *CNL-FvH4.6g13450.1* | | | | | | | | |
| *TNL-FDA.chr7.00085* | *TNL-FvH4.3g43650.1* | | | | *CN-FvH4.7g11820.1* | *CNL-FII.chr7.1027* | *N-FNG.chr6.1058* | | | | | *N-FII.chr6.1009* | | | | | | | | |
| *TNL-FDA.chr7.00110* | *TNL-FvH4.7g33190.1* | | | | *CN-FvH4.7g11820.1* | *CNL-FNG.chr7.951* | *N-FNG.chr6.1059* | | | | | *CNL-FDA.chr6.26337* | | | | | | | | |
| *TNL-FDA.chr7.00114* | *TNL-FvH4.7g32740.1* | | | | *CN-FvH4.7g11820.1* | *CNL-FNU.ctg132.293* | *N-FNG.chr6.1059* | | | | | *CNL-FMA.chr6.1658* | | | | | | | | |
| *TNL-FDA.chr7.00159* | *TNL-FvH4.7g32460.1* | | | | *CN-FvH4.7g23850.1* | *CNL-FDA.chr7.01192* | *N-FNG.chr6.1059* | | | | | *CNL-FNU.ctg78.2* | | | | | | | | |
| Orthologous gene pairsof TNL | | | | | Orthologous gene pairs of non-TNL | | | | | | | | | | | | | | |
| ID of sequence 1 | ID of sequence 2 | | | | ID of sequence 1 | ID of sequence 2 | ID of sequence 1 | | | | | | | | ID of sequence 2 | | | | |
| *TNL-FDA.chr7.00184* | *TNL-FvH4.7g32460.1* | | | | *CN-FvH4.7g23850.1* | *CNL-FNU.ctg129.61* | *N-FNG.chr6.1059* | | | | | *CNL-FvH4.6g13450.1* | | | | | | | | |
| *TNL-FDA.chr7.00189* | *TNL-FvH4.7g32460.1* | | | | *CN-FvH4.7g31270.1* | *CNL-FDA.chr6.02010* | *N-FNU.ctg119.817* | | | | | *CN-FDA.chr2.12212* | | | | | | | | |
| *TNL-FDA.chr7.00190* | *TNL-FvH4.7g32740.1* | | | | *CN-FVI.CHR2.1419* | *CN-FDA.chr6.17380* | *N-FNU.ctg119.817* | | | | | *CNL-FvH4.2g36810.1* | | | | | | | | |
| *TNL-FDA.chr7.00274* | *TNL-FvH4.7g31510.1* | | | | *CN-FVI.CHR2.1419* | *CN-FII.chr6.2648* | *N-FNU.ctg119.818* | | | | | *CNL-FDA.chr2.12218* | | | | | | | | |
| *TNL-FDA.chr7.00582* | *TNL-FII.chr7.2338* | | | | *CN-FVI.CHR2.1419* | *CN-FMA.chr6.2734* | *N-FNU.ctg119.818* | | | | | *CNL-FvH4.2g36800.1* | | | | | | | | |
| *TNL-FDA.chr7.00582* | *TNL-FPE.chr7.2368* | | | | *CN-FVI.CHR2.1419* | *CN-FNU.ctg64.1* | *N-FNU.ctg119.819* | | | | | *CN-FDA.chr2.12213* | | | | | | | | |
| *TNL-FDA.chr7.00582* | *TNL-FvH4.7g28550.1* | | | | *CN-FVI.CHR2.1419* | *CN-FPE.chr6.470* | *N-FNU.ctg119.819* | | | | | *CNL-FvH4.2g36860.1* | | | | | | | | |
| *TNL-FDA.chr7.00835* | *TNL-FII.chr7.2338* | | | | *CN-FVI.CHR2.1419* | *N-FvH4.6g30240.1* | *N-FNU.ctg124.289* | | | | | *CN-FvH4.7g31270.1* | | | | | | | | |
| *TNL-FDA.chr7.00835* | *TNL-FPE.chr7.2368* | | | | *CN-FVI.CHR2.1513* | *CN-FDA.chr6.17288* | *N-FNU.ctg124.289* | | | | | *CNL-FDA.chr6.02010* | | | | | | | | |
| *TNL-FDA.chr7.00835* | *TNL-FvH4.7g28550.1* | | | | *CN-FVI.CHR2.1513* | *CN-FII.chr6.2548* | *N-FNU.ctg155.194* | | | | | *N-FvH4.5g21070.1* | | | | | | | | |
| *TNL-FDA.chr7.01326* | *TNL-FvH4.7g21230.1* | | | | *CN-FVI.CHR2.1513* | *CN-FMA.chr6.2632* | *N-FNU.ctg155.194* | | | | | *NL-FNG.chr5.1946* | | | | | | | | |
| *TNL-FDA.chr7.01333* | *TNL-FvH4.7g21060.1* | | | | *CN-FVI.CHR2.1513* | *CN-FNG.chr6.1594* | *N-FNU.ctg161.240* | | | | | *CNL-FII.chr5.3003* | | | | | | | | |
| *TNL-FDA.chr7.01621* | *TNL-FPE.chr7.2368* | | | | *CN-FVI.CHR2.1513* | *CN-FNU.ctg65.105* | *N-FNU.ctg161.240* | | | | | *CNL-FNG.chr5.2999* | | | | | | | | |
| *TNL-FDA.chr7.01621* | *TNL-FvH4.7g17700.1* | | | | *CN-FVI.CHR2.1513* | *CN-FPE.chr6.1820* | *N-FNU.ctg161.240* | | | | | *CNL-FvH4.5g34680.1* | | | | | | | | |
| *TNL-FDA.chr7.02890* | *TNL-FNU.ctg129.394* | | | | *CN-FVI.CHR2.1513* | *CN-FvH4.6g29230.1* | *N-FNU.ctg172.22* | | | | | *RNL-FDA.chr1.22182* | | | | | | | | |
| *TNL-FDA.chr7.02890* | *TNL-FPE.chr7.2368* | | | | *CN-FVI.CHR2.1517* | *CNL-FDA.chr3.16699* | *N-FNU.ctg172.22* | | | | | *RNL-FII.chr1.1265* | | | | | | | | |
| *TNL-FDA.chr7.02890* | *TNL-FvH4.7g28550.1* | | | | *CN-FVI.CHR2.1517* | *CNL-FII.chr6.2256* | *N-FNU.ctg48.4* | | | | | *CNL-FII.chr3.1385* | | | | | | | | |
| *TNL-FDA.chr7.02892* | *TNL-FII.chr7.2338* | | | | *CN-FVI.CHR2.1517* | *CNL-FvH4.6g29480.1* | *N-FNU.ctg48.4* | | | | | *CNL-FMA.chr3.1344* | | | | | | | | |
| *TNL-FDA.chr7.02892* | *TNL-FPE.chr7.2368* | | | | *CN-FVI.CHR4.1436* | *CNL-FDA.chr4.24588* | *N-FNU.ctg48.4* | | | | | *CNL-FNG.chr3.1371* | | | | | | | | |
| *TNL-FDA.chr7.02892* | *TNL-FvH4.7g17700.1* | | | | *CN-FVI.CHR4.1436* | *CNL-FII.chr4.1300* | *N-FNU.ctg48.4* | | | | | *CNL-FPE.chr5.124* | | | | | | | | |
| *TNL-FDA.chr7.14713* | *TNL-FNG.chr7.2518* | | | | *CN-FVI.CHR4.1436* | *CNL-FNU.ctg154.468* | *N-FNU.ctg48.4* | | | | | *NL-FDA.chr3.21153* | | | | | | | | |
| *TNL-FDA.chr7.14713* | *TNL-FNU.ctg132.96* | | | | *CN-FVI.CHR4.1436* | *CNL-FvH4.4g16700.1* | *N-FNU.ctg56.229* | | | | | *CNL-FDA.chr1.06545* | | | | | | | | |
| *TNL-FDA.chr7.14713* | *TNL-FvH4.7g06680.1* | | | | *CN-FVI.CHR4.1436* | *NL-FMA.chr4.1335* | *N-FNU.ctg56.229* | | | | | *CNL-FII.chr7.998* | | | | | | | | |
| *TNL-FII.chr1.1393* | *TNL-FNG.chr1.1446* | | | | *CN-FVI.CHR4.1436* | *NL-FPE.chr3.4014* | *N-FNU.ctg56.229* | | | | | *CNL-FMA.chr7.880* | | | | | | | | |
| *TNL-FII.chr1.1393* | *TNL-FNU.ctg172.194* | | | | *CN-FVI.CHR5.3012* | *CN-FII.chr6.2548* | *N-FNU.ctg56.229* | | | | | *CNL-FPE.chr7.1015* | | | | | | | | |
| *TNL-FII.chr1.1393* | *TNL-FvH4.1g16600.1* | | | | *CN-FVI.CHR5.3012* | *CN-FMA.chr6.2632* | *N-FNU.ctg57.541* | | | | | *CN-FII.chr3.955* | | | | | | | | |
| *TNL-FII.chr1.1394* | *TNL-FNG.chr1.1446* | | | | *CN-FVI.CHR5.3012* | *CN-FNG.chr5.2696* | *N-FNU.ctg65.16* | | | | | *CN-FII.chr6.2623* | | | | | | | | |
| *TNL-FII.chr1.1394* | *TNL-FNU.ctg172.196* | | | | *CN-FVI.CHR5.3012* | *CN-FNU.ctg161.296* | *N-FNU.ctg65.16* | | | | | *CN-FMA.chr6.2719* | | | | | | | | |
| *TNL-FII.chr1.1394* | *TNL-FvH4.1g16650.1* | | | | *CN-FVI.CHR5.3012* | *CN-FPE.chr5.3016* | *N-FPE.chr0.2268* | | | | | *CN-FDA.chr6.20969* | | | | | | | | |
| *TNL-FII.chr1.757* | *TNL-FPE.chr6.1904* | | | | *CN-FVI.CHR5.3012* | *CN-FvH4.6g29230.1* | *N-FPE.chr0.2268* | | | | | *CN-FII.chr6.1890* | | | | | | | | |
| *TNL-FII.chr1.757* | *TNL-FvH4.3g10020.1* | | | | *CN-FVI.CHR6.1776* | *CN-FII.chr6.1267* | *N-FPE.chr0.2268* | | | | | *CN-FMA.chr6.1745* | | | | | | | | |
| *TNL-FII.chr2.1158* | *TNL-FvH4.2g14040.1* | | | | *CN-FVI.CHR6.1776* | *CNL-FDA.chr6.26153* | *N-FPE.chr0.2268* | | | | | *CN-FNG.chr6.1541* | | | | | | | | |
| *TNL-FII.chr2.1183* | *TNL-FDA.chr6.02345* | | | | *CN-FVI.CHR6.1776* | *CNL-FMA.chr6.1184* | *N-FPE.chr0.2268* | | | | | *CN-FNU.ctg71.38* | | | | | | | | |
| *TNL-FII.chr2.1183* | *TNL-FMA.chr2.857* | | | | *CN-FVI.CHR6.1776* | *CNL-FNG.chr6.2420* | *N-FPE.chr0.2268* | | | | | *CN-FvH4.6g22150.1* | | | | | | | | |
| *TNL-FII.chr2.1183* | *TNL-FNU.ctg8.247* | | | | *CN-FVI.CHR6.1776* | *CNL-FNU.ctg78.2* | *N-FPE.chr2.64* | | | | | *CNL-FII.chr3.951* | | | | | | | | |
| *TNL-FII.chr2.1183* | *TNL-FPE.chr3.3124* | | | | *CN-FVI.CHR6.1776* | *CNL-FvH4.6g15250.1* | *N-FPE.chr2.64* | | | | | *CNL-FvH4.2g13150.1* | | | | | | | | |
| *TNL-FII.chr2.1183* | *TNL-FvH4.2g14320.1* | | | | *CN-FVI.CHR6.2188* | *CN-FDA.chr6.20969* | *N-FPE.chr3.1891* | | | | | *CNL-FvH4.3g11470.1* | | | | | | | | |
| *TNL-FII.chr2.172* | *TNL-FNG.chr2.105* | | | | *CN-FVI.CHR6.2188* | *CN-FII.chr6.1890* | *N-FPE.chr3.1892* | | | | | *CNL-FII.chr3.953* | | | | | | | | |
| *TNL-FII.chr2.172* | *TNL-FPE.chr0.1078* | | | | *CN-FVI.CHR6.2188* | *CN-FMA.chr6.1745* | *N-FPE.chr3.1892* | | | | | *CNL-FvH4.3g11490.1* | | | | | | | | |
| *TNL-FII.chr2.172* | *TNL-FvH4.2g02050.1* | | | | *CN-FVI.CHR6.2188* | *CN-FNG.chr6.1541* | *N-FPE.chr3.1895* | | | | | *CNL-FvH4.3g11490.1* | | | | | | | | |
| *TNL-FII.chr2.172* | *TNL-FNU.ctg101.157* | | | | *CN-FVI.CHR6.2188* | *CN-FNU.ctg71.38* | *N-FPE.chr3.3480* | | | | | *N-FDA.chr3.06067* | | | | | | | | |
| *TNL-FII.chr2.1754* | *TNL-FNG.chr2.1702* | | | | *CN-FVI.CHR6.2188* | *CN-FPE.chr0.885* | *N-FPE.chr3.3480* | | | | | *N-FMA.chr3.3384* | | | | | | | | |
| Orthologous gene pairsof TNL | | | | | Orthologous gene pairs of non-TNL | | | | | | | | | | | | | | |
| ID of sequence 1 | ID of sequence 2 | | | | ID of sequence 1 | ID of sequence 2 | ID of sequence 1 | | | | | | | | ID of sequence 2 | | | | |
| *TNL-FII.chr2.1754* | *TNL-FNU.ctg135.24* | | | | *CN-FVI.CHR6.2188* | *CN-FvH4.6g22150.1* | *N-FPE.chr3.3480* | | | | | *N-FNG.chr3.3429* | | | | | | | | |
| *TNL-FII.chr2.1754* | *TNL-FvH4.2g20310.1* | | | | *CN-FVI.CHR6.2635* | *CN-FDA.chr6.16615* | *N-FPE.chr3.3480* | | | | | *N-FvH4.3g41580.1* | | | | | | | | |
| *TNL-FII.chr2.49* | *TNL-FNG.chr2.105* | | | | *CN-FVI.CHR6.2635* | *CN-FII.chr6.3270.2* | *N-FPE.chr5.126* | | | | | *CN-FvH4.3g16720.1* | | | | | | | | |
| *TNL-FII.chr2.49* | *TNL-FPE.chr2.91* | | | | *CN-FVI.CHR6.2635* | *CN-FMA.chr6.2632* | *N-FPE.chr5.126* | | | | | *CNL-FII.chr3.1384* | | | | | | | | |
| *TNL-FII.chr2.49* | *TNL-FvH4.7g12160.1* | | | | *CN-FVI.CHR6.2635* | *CN-FNG.chr6.3269* | *N-FPE.chr5.126* | | | | | *CNL-FNG.chr3.1370* | | | | | | | | |
| *TNL-FII.chr2.55* | *TNL-FNG.chr3.3728* | | | | *CN-FVI.CHR6.2635* | *CN-FNU.ctg57.114* | *N-FPE.chr5.126* | | | | | *CNL-FNU.ctg48.6* | | | | | | | | |
| *TNL-FII.chr2.55* | *TNL-FNU.ctg134.31* | | | | *CN-FVI.CHR6.2635* | *CN-FPE.chr6.3007* | *N-FPE.chr6.5282* | | | | | *RN-FDA.chr6.13755* | | | | | | | | |
| *TNL-FII.chr2.55* | *TNL-FvH4.2g38620.1* | | | | *CN-FVI.CHR6.2635* | *CN-FvH4.6g29230.1* | *N-FPE.chr6.5282* | | | | | *RN-FNU.ctg52.52* | | | | | | | | |
| *TNL-FII.chr2.708* | *TNL-FPE.chr3.552* | | | | *CN-FVI.CHR7.3759* | *CNL-FII.chr3.954* | *N-FPE.chr6.5282* | | | | | *RNL-FvH4.6g50310.1* | | | | | | | | |
| *TNL-FII.chr2.708* | *TNL-FvH4.5g38870.1* | | | | *CN-FVI.CHR7.3759* | *CNL-FvH4.3g11490.1* | *N-FPE.chr6.572* | | | | | *N-FvH4.3g41580.1* | | | | | | | | |
| *TNL-FII.chr2.77* | *TNL-FNU.ctg101.35* | | | | *CNL-FDA.chr1.06545* | *CNL-FMA.chr7.880* | *N-FvH4.1g12710.1* | | | | | *CNL-FDA.chr3.23062* | | | | | | | | |
| *TNL-FII.chr2.77* | *TNL-FPE.chr6.5657* | | | | *CNL-FDA.chr1.22189* | *NL-FII.chr1.1268* | *N-FvH4.1g12710.1* | | | | | *CNL-FNU.ctg170.24* | | | | | | | | |
| *TNL-FII.chr2.77* | *TNL-FvH4.1g22390.1* | | | | *CNL-FDA.chr1.22189* | *RNL-FMA.chr1.1806* | *N-FvH4.2g08360.1* | | | | | *N-FMA.chr2.489* | | | | | | | | |
| *TNL-FII.chr3.1313* | *TNL-FMA.chr2.2281* | | | | *CNL-FDA.chr1.22189* | *RNL-FvH4.1g15220.1* | *N-FvH4.2g17400.1* | | | | | *CN-FII.chr6.198* | | | | | | | | |
| *TNL-FII.chr3.1313* | *TNL-FNG.chr2.2392* | | | | *CNL-FDA.chr2.12208* | *CN-FvH4.2g36850.1* | *N-FvH4.2g17400.1* | | | | | *CNL-FDA.chr6.02198* | | | | | | | | |
| *TNL-FII.chr3.1313* | *TNL-FNU.ctg32.58* | | | | *CNL-FDA.chr2.12209* | *CNL-FvH4.2g36810.1* | *N-FvH4.2g17400.1* | | | | | *CNL-FNG.chr2.1430* | | | | | | | | |
| *TNL-FII.chr3.1313* | *TNL-FvH4.3g15720.1* | | | | *CNL-FDA.chr2.12218* | *CNL-FvH4.2g36800.1* | *N-FvH4.3g12820.1* | | | | | *N-FPE.chr6.5282* | | | | | | | | |
| *TNL-FII.chr3.1814* | *TNL-FNG.chr3.2334* | | | | *CNL-FDA.chr2.17966* | *CNL-FII.chr3.1811* | *N-FvH4.3g12820.1* | | | | | *RN-FDA.chr6.13755* | | | | | | | | |
| *TNL-FII.chr3.1830* | *TNL-FNG.chr3.2352* | | | | *CNL-FDA.chr2.17966* | *CNL-FMA.chr2.328* | *N-FvH4.3g12820.1* | | | | | *RN-FII.chr3.1087* | | | | | | | | |
| *TNL-FII.chr3.1830* | *TNL-FvH4.7g12160.1* | | | | *CNL-FDA.chr2.17966* | *CNL-FNG.chr2.416* | *N-FvH4.3g12820.1* | | | | | *RN-FNU.ctg52.52* | | | | | | | | |
| *TNL-FII.chr3.1831* | *TNL-FDA.chr7.00159* | | | | *CNL-FDA.chr2.17966* | *CNL-FNU.ctg104.183* | *N-FvH4.4g23400.1* | | | | | *CN-FPE.chr6.878* | | | | | | | | |
| *TNL-FII.chr3.1831* | *TNL-FNG.chr3.2352* | | | | *CNL-FDA.chr2.19060* | *CN-FII.chr2.551* | *N-FvH4.4g23400.1* | | | | | *CNL-FII.chr4.1849.2* | | | | | | | | |
| *TNL-FII.chr3.1831* | *TNL-FvH4.7g12160.1* | | | | *CNL-FDA.chr2.19060* | *CN-FMA.chr2.503* | *N-FvH4.4g23400.1* | | | | | *CNL-FNG.chr4.1917* | | | | | | | | |
| *TNL-FII.chr3.3419* | *TNL-FPE.chr6.1904* | | | | *CNL-FDA.chr2.19060* | *CN-FPE.chr7.728* | *N-FvH4.4g23400.1* | | | | | *CNL-FNU.ctg82.648* | | | | | | | | |
| *TNL-FII.chr3.3419* | *TNL-FvH4.4g13420.1* | | | | *CNL-FDA.chr2.19060* | *CN-FvH4.2g06550.1* | *N-FvH4.5g11130.1* | | | | | *N-FNU.ctg172.28* | | | | | | | | |
| *TNL-FII.chr3.3603* | *TNL-FDA.chr3.05837* | | | | *CNL-FDA.chr2.19060* | *CNL-FNG.chr2.482* | *N-FvH4.5g11130.1* | | | | | *RNL-FDA.chr1.22179* | | | | | | | | |
| *TNL-FII.chr3.3603* | *TNL-FNG.chr3.3322* | | | | *CNL-FDA.chr2.19060* | *CNL-FNU.ctg104.299* | *N-FvH4.5g11130.1* | | | | | *RNL-FMA.chr1.1807* | | | | | | | | |
| *TNL-FII.chr3.3603* | *TNL-FNU.ctg3.75* | | | | *CNL-FDA.chr3.05415* | *CNL-FNU.ctg129.61* | *N-FvH4.5g21530.1* | | | | | *NL-FII.chr1.1268* | | | | | | | | |
| *TNL-FII.chr3.3603* | *TNL-FvH4.3g44070.1* | | | | *CNL-FDA.chr3.05415* | *CNL-FvH4.7g22030.1* | *N-FvH4.5g21530.1* | | | | | *RN-FNG.chr1.1331* | | | | | | | | |
| *TNL-FII.chr3.3626* | *TNL-FDA.chr3.05837* | | | | *CNL-FDA.chr3.09595* | *CNL-FII.chr3.408* | *N-FvH4.5g21530.1* | | | | | *RNL-FDA.chr1.22182* | | | | | | | | |
| *TNL-FII.chr3.3626* | *TNL-FNG.chr3.3322* | | | | *CNL-FDA.chr3.09595* | *CNL-FNG.chr0.411* | *N-FvH4.5g21530.1* | | | | | *RNL-FMA.chr1.1806* | | | | | | | | |
| *TNL-FII.chr3.3626* | *TNL-FNU.ctg3.75* | | | | *CNL-FDA.chr3.09595* | *CNL-FPE.chr3.535* | *N-FvH4.5g24320.1* | | | | | *CNL-FII.chr7.789* | | | | | | | | |
| *TNL-FII.chr3.3626* | *TNL-FvH4.3g44000.1* | | | | *CNL-FDA.chr3.09595* | *CNL-FvH4.7g31140.1* | *N-FvH4.5g24320.1* | | | | | *CNL-FNU.ctg158.149* | | | | | | | | |
| *TNL-FII.chr3.744.2* | *TNL-FPE.chr6.1904* | | | | *CNL-FDA.chr3.15906* | *CNL-FII.chr3.953* | *N-FvH4.5g24320.1* | | | | | *CNL-FPE.chr7.1124* | | | | | | | | |
| *TNL-FII.chr3.744.2* | *TNL-FvH4.4g13420.1* | | | | *CNL-FDA.chr3.15906* | *CNL-FvH4.3g11490.1* | *N-FvH4.6g30050.1* | | | | | *CN-FII.chr6.2623* | | | | | | | | |
| *TNL-FII.chr4.2875* | *TNL-FMA.chr4.2946* | | | | *CNL-FDA.chr3.16699* | *CNL-FvH4.3g02290.1* | *N-FvH4.6g30050.1* | | | | | *CN-FPE.chr6.447* | | | | | | | | |
| *TNL-FII.chr4.2875* | *TNL-FNG.chr4.3018* | | | | *CNL-FDA.chr3.16968* | *CNL-FII.chr3.408* | *N-FvH4.6g30050.1* | | | | | *N-FNU.ctg65.16* | | | | | | | | |
| *TNL-FII.chr4.2875* | *TNL-FNU.ctg81.212* | | | | *CNL-FDA.chr3.16968* | *CNL-FPE.chr3.535* | *N-FvH4.6g30240.1* | | | | | *CN-FNU.ctg64.1* | | | | | | | | |
| *TNL-FII.chr4.2875* | *TNL-FvH4.4g35420.1* | | | | *CNL-FDA.chr3.16968* | *CNL-FvH4.3g04780.1* | *N-FvH4.6g50120.1* | | | | | *CNL-FII.chr1.1471* | | | | | | | | |
| *TNL-FII.chr4.2876* | *TNL-FNG.chr4.3018* | | | | *CNL-FDA.chr3.16978* | *CNL-FII.chr3.408* | *N-FvH4.6g50120.1* | | | | | *RNL-FDA.chr6.13739* | | | | | | | | |
| *TNL-FII.chr4.2876* | *TNL-FNU.ctg81.211* | | | | *CNL-FDA.chr3.16978* | *CNL-FPE.chr3.535* | *N-FvH4.6g50120.1* | | | | | *RNL-FNG.chr6.4113* | | | | | | | | |
| *TNL-FII.chr5.1800* | *TNL-FNU.ctg160.483* | | | | *CNL-FDA.chr3.16978* | *CNL-FvH4.3g04780.1* | *N-FvH4.7g09280.1* | | | | | *CNL-FNU.ctg114.210* | | | | | | | | |
| Orthologous gene pairsof TNL | | | | | Orthologous gene pairs of non-TNL | | | | | | | | | | | | | | |
| ID of sequence 1 | ID of sequence 2 | | | | ID of sequence 1 | ID of sequence 2 | ID of sequence 1 | | | | | | | | ID of sequence 2 | | | | |
| *TNL-FII.chr5.1800* | *TNL-FvH4.5g32970.1* | | | | *CNL-FDA.chr3.23062* | *CNL-FII.chr3.77* | *N-FvH4.7g09280.1* | | | | | *CNL-FPE.chr7.335* | | | | | | | | |
| *TNL-FII.chr5.2851* | *TNL-FNU.ctg160.481* | | | | *CNL-FDA.chr3.23062* | *CNL-FMA.chr3.75* | *N-FvH4.7g09280.1* | | | | | *CN-FDA.chr1.06861* | | | | | | | | |
| *TNL-FII.chr5.2851* | *TNL-FvH4.5g32970.1* | | | | *CNL-FDA.chr3.23062* | *CNL-FNG.chr3.84* | *N-FvH4.7g15200.1* | | | | | *CNL-FII.chr7.789* | | | | | | | | |
| *TNL-FII.chr5.2900* | *TNL-FMA.chr1.1780* | | | | *CNL-FDA.chr3.23062* | *CNL-FNU.ctg47.220* | *N-FvH4.7g15200.1* | | | | | *CNL-FPE.chr7.1124* | | | | | | | | |
| *TNL-FII.chr5.2900* | *TNL-FvH4.1g15650.1* | | | | *CNL-FDA.chr3.23062* | *CNL-FvH4.3g00820.1* | *N-FVI.CHR1.1875* | | | | | *CN-FII.chr1.1941* | | | | | | | | |
| *TNL-FII.chr5.2969* | *TNL-FvH4.5g34210.1* | | | | *CNL-FDA.chr3.25684* | *CNL-FII.chr3.954* | *N-FVI.CHR1.1875* | | | | | *CNL-FDA.chr6.02010* | | | | | | | | |
| *TNL-FII.chr5.2970* | *TNL-FNU.ctg160.483* | | | | *CNL-FDA.chr3.25684* | *CNL-FvH4.3g11490.1* | *N-FVI.CHR1.1875* | | | | | *CNL-FvH4.1g23030.1* | | | | | | | | |
| *TNL-FII.chr5.2970* | *TNL-FvH4.5g34210.1* | | | | *CNL-FDA.chr3.25684* | *N-FPE.chr3.1895* | *N-FVI.CHR1.2224* | | | | | *N-FvH4.5g21530.1* | | | | | | | | |
| *TNL-FII.chr5.302.2* | *TNL-FMA.chr5.364* | | | | *CNL-FDA.chr4.11333* | *CNL-FII.chr4.377* | *N-FVI.CHR1.2224* | | | | | *NL-FII.chr1.1268* | | | | | | | | |
| *TNL-FII.chr5.302.2* | *TNL-FvH4.5g03630.1* | | | | *CNL-FDA.chr4.11333* | *CNL-FMA.chr4.679* | *N-FVI.CHR1.2224* | | | | | *RN-FNG.chr1.1331* | | | | | | | | |
| *TNL-FII.chr5.3330* | *TNL-FNG.chr2.105* | | | | *CNL-FDA.chr4.11333* | *CNL-FNG.chr0.411* | *N-FVI.CHR1.2224* | | | | | *RNL-FDA.chr1.22182* | | | | | | | | |
| *TNL-FII.chr5.3330* | *TNL-FvH4.5g38680.1* | | | | *CNL-FDA.chr4.11333* | *CNL-FvH4.4g06030.1* | *N-FVI.CHR1.2224* | | | | | *RNL-FMA.chr1.1806* | | | | | | | | |
| *TNL-FII.chr5.3343* | *TNL-FMA.chr5.2877* | | | | *CNL-FDA.chr4.24588* | *CNL-FII.chr4.1300* | *N-FVI.CHR2.1889* | | | | | *CNL-FII.chr3.951* | | | | | | | | |
| *TNL-FII.chr5.3343* | *TNL-FvH4.5g38870.1* | | | | *CNL-FDA.chr4.24588* | *CNL-FvH4.4g16700.1* | *N-FVI.CHR2.1889* | | | | | *CNL-FvH4.2g13150.1* | | | | | | | | |
| *TNL-FII.chr5.3370* | *TNL-FDA.chr5.12815* | | | | *CNL-FDA.chr4.24588* | *NL-FMA.chr4.1335* | *N-FVI.CHR2.1889* | | | | | *N-FPE.chr2.64* | | | | | | | | |
| *TNL-FII.chr5.3370* | *TNL-FMA.chr1.1780* | | | | *CNL-FDA.chr4.24588* | *NL-FNG.chr4.1397* | *N-FVI.CHR2.1890* | | | | | *CNL-FvH4.2g13150.1* | | | | | | | | |
| *TNL-FII.chr5.3370* | *TNL-FvH4.1g15650.1* | | | | *CNL-FDA.chr4.24588* | *NL-FNU.ctg84.211* | *N-FVI.CHR2.1890* | | | | | *N-FII.chr2.1082* | | | | | | | | |
| *TNL-FII.chr5.3371* | *TNL-FMA.chr5.2877* | | | | *CNL-FDA.chr4.24588* | *NL-FPE.chr3.4014* | *N-FVI.CHR2.1890* | | | | | *N-FPE.chr2.64* | | | | | | | | |
| *TNL-FII.chr5.3371* | *TNL-FvH4.1g15650.1* | | | | *CNL-FDA.chr5.03688* | *CNL-FvH4.3g02290.1* | *N-FVI.CHR3.1742* | | | | | *CNL-FPE.chr2.767* | | | | | | | | |
| *TNL-FII.chr5.609* | *TNL-FDA.chr3.09474* | | | | *CNL-FDA.chr5.07881* | *CNL-FII.chr3.408* | *N-FVI.CHR3.1742* | | | | | *NL-FNG.chr3.1831* | | | | | | | | |
| *TNL-FII.chr5.609* | *TNL-FMA.chr5.364* | | | | *CNL-FDA.chr5.07881* | *CNL-FMA.chr5.1739* | *N-FVI.CHR3.2405* | | | | | *N-FDA.chr3.06067* | | | | | | | | |
| *TNL-FII.chr5.609* | *TNL-FvH4.5g07310.1* | | | | *CNL-FDA.chr5.07881* | *CNL-FNG.chr5.1760* | *N-FVI.CHR3.2405* | | | | | *N-FMA.chr3.3384* | | | | | | | | |
| *TNL-FII.chr6.136* | *TNL-FDA.chr7.00582* | | | | *CNL-FDA.chr5.07881* | *CNL-FvH4.5g18950.1* | *N-FVI.CHR3.2405* | | | | | *N-FNG.chr3.3429* | | | | | | | | |
| *TNL-FII.chr6.136* | *TNL-FNU.ctg134.16* | | | | *CNL-FDA.chr5.22369* | *CN-FNG.chr5.2692* | *N-FVI.CHR3.2405* | | | | | *N-FPE.chr6.572* | | | | | | | | |
| *TNL-FII.chr6.136* | *TNL-FPE.chr7.2368* | | | | *CNL-FDA.chr5.22369* | *CNL-FMA.chr5.2388* | *N-FVI.CHR3.2405* | | | | | *N-FvH4.3g41580.1* | | | | | | | | |
| *TNL-FII.chr6.136* | *TNL-FvH4.6g01690.1* | | | | *CNL-FDA.chr5.22369* | *CNL-FvH4.7g31140.1* | *N-FVI.CHR7.1334* | | | | | *CNL-FII.chr7.789* | | | | | | | | |
| *TNL-FII.chr6.2998* | *TNL-FMA.chr5.2425* | | | | *CNL-FDA.chr5.23812* | *CNL-FII.chr3.408* | *N-FVI.CHR7.1334* | | | | | *CNL-FNU.ctg114.210* | | | | | | | | |
| *TNL-FII.chr6.2998* | *TNL-FNG.chr5.2639* | | | | *CNL-FDA.chr5.23812* | *CNL-FPE.chr3.535* | *N-FVI.CHR7.1334* | | | | | *CNL-FPE.chr7.1124* | | | | | | | | |
| *TNL-FII.chr6.2998* | *TNL-FvH4.6g34410.1* | | | | *CNL-FDA.chr5.23812* | *CNL-FvH4.5g05380.1* | *N-FVI.CHR7.1334* | | | | | *CNL-FvH4.7g15240.1* | | | | | | | | |
| *TNL-FII.chr6.322* | *TNL-FvH4.6g05610.1* | | | | *CNL-FDA.chr5.24089* | *CNL-FII.chr4.377* | *N-FVI.CHR7.3760* | | | | | *CNL-FII.chr3.954* | | | | | | | | |
| *TNL-FII.chr7.1056* | *TNL-FDA.chr7.00159* | | | | *CNL-FDA.chr5.24089* | *CNL-FMA.chr4.679* | *N-FVI.CHR7.3760* | | | | | *CNL-FvH4.3g11490.1* | | | | | | | | |
| *TNL-FII.chr7.1056* | *TNL-FNG.chr3.2352* | | | | *CNL-FDA.chr5.24089* | *CNL-FvH4.4g06030.1* | *N-FVI.CHR7.3760* | | | | | *N-FPE.chr3.1895* | | | | | | | | |
| *TNL-FII.chr7.1056* | *TNL-FvH4.7g12160.1* | | | | *CNL-FDA.chr5.24311* | *CNL-FNG.chr0.411* | *N-FVI.CHR7.738* | | | | | *CN-FNG.chr7.813* | | | | | | | | |
| *TNL-FII.chr7.1130* | *TNL-FNG.chr5.2639* | | | | *CNL-FDA.chr5.24311* | *CNL-FNU.ctg150.57* | *N-FVI.CHR7.738* | | | | | *CNL-FII.chr7.708* | | | | | | | | |
| *TNL-FII.chr7.1130* | *TNL-FvH4.7g13090.1* | | | | *CNL-FDA.chr5.24966* | *CNL-FII.chr5.2008* | *N-FVI.CHR7.738* | | | | | *CNL-FPE.chr0.563* | | | | | | | | |
| *TNL-FII.chr7.2338* | *TNL-FPE.chr7.2368* | | | | *CNL-FDA.chr5.24966* | *CNL-FNG.chr5.2227* | *N-FVI.CHR7.738* | | | | | *CNL-FvH4.7g08100.1* | | | | | | | | |
| *TNL-FII.chr7.2338* | *TNL-FvH4.7g28550.1* | | | | *CNL-FDA.chr5.24966* | *CNL-FNU.ctg158.148* | *NL-FDA.chr2.17949* | | | | | *CNL-FMA.chr2.328* | | | | | | | | |
| *TNL-FII.chr7.790* | *TNL-FNG.chr3.2334* | | | | *CNL-FDA.chr5.24966* | *CNL-FvH4.5g23470.1* | *NL-FDA.chr2.17949* | | | | | *CNL-FNG.chr2.416* | | | | | | | | |
| *TNL-FII.chr7.790* | *TNL-FvH4.7g31110.1* | | | | *CNL-FDA.chr5.25044* | *CNL-FII.chr5.2008* | *NL-FDA.chr2.17949* | | | | | *CNL-FNU.ctg104.183* | | | | | | | | |
| *TNL-FII.chr7.857* | *TNL-FMA.chr2.1405* | | | | *CNL-FDA.chr5.25044* | *CNL-FNG.chr5.2150* | *NL-FDA.chr2.17949* | | | | | *CNL-FPE.chr7.1798* | | | | | | | | |
| *TNL-FII.chr7.857* | *TNL-FNU.ctg135.24* | | | | *CNL-FDA.chr5.25044* | *CNL-FNU.ctg158.48* | *NL-FDA.chr2.17949* | | | | | *NL-FII.chr2.452* | | | | | | | | |
| *TNL-FII.chr7.857* | *TNL-FvH4.7g09800.1* | | | | *CNL-FDA.chr5.25044* | *CNL-FvH4.5g23470.1* | *NL-FDA.chr3.06281* | | | | | *CNL-FII.chr3.1811* | | | | | | | | |
| Orthologous gene pairsof TNL | | | | | Orthologous gene pairs of non-TNL | | | | | | | | | | | | | | |
| ID of sequence 1 | ID of sequence 2 | | | | ID of sequence 1 | ID of sequence 2 | ID of sequence 1 | | | | | | | | ID of sequence 2 | | | | |
| *TNL-FII.chr7.921* | *TNL-FMA.chr2.1405* | | | | *CNL-FDA.chr5.25047* | *CNL-FII.chr5.2006* | *NL-FDA.chr3.21153* | | | | | *CN-FvH4.3g16720.1* | | | | | | | | |
| *TNL-FII.chr7.921* | *TNL-FNG.chr7.552* | | | | *CNL-FDA.chr5.25047* | *CNL-FNG.chr5.2149* | *NL-FDA.chr3.21153* | | | | | *CNL-FII.chr3.1385* | | | | | | | | |
| *TNL-FII.chr7.921* | *TNL-FPE.chr2.3663* | | | | *CNL-FDA.chr5.25047* | *CNL-FNU.ctg158.45* | *NL-FDA.chr3.21153* | | | | | *CNL-FMA.chr3.1344* | | | | | | | | |
| *TNL-FII.chr7.921* | *TNL-FvH4.7g10750.1* | | | | *CNL-FDA.chr5.25047* | *CNL-FvH4.5g23450.1* | *NL-FDA.chr3.21153* | | | | | *CNL-FNG.chr1.545* | | | | | | | | |
| *TNL-FII.chr7.923* | *TNL-FMA.chr2.1405* | | | | *CNL-FDA.chr5.25049* | *CNL-FII.chr5.2004* | *NL-FDA.chr3.21153* | | | | | *CNL-FNU.ctg165.171* | | | | | | | | |
| *TNL-FII.chr7.923* | *TNL-FNG.chr7.552* | | | | *CNL-FDA.chr5.25049* | *CNL-FNG.chr5.2147* | *NL-FDA.chr3.21153* | | | | | *CNL-FPE.chr5.124* | | | | | | | | |
| *TNL-FII.chr7.923* | *TNL-FNU.ctg134.120* | | | | *CNL-FDA.chr5.25049* | *CNL-FNU.ctg158.43* | *NL-FDA.chr4.04655* | | | | | *NL-FvH4.4g35430.1* | | | | | | | | |
| *TNL-FII.chr7.923* | *TNL-FvH4.7g10520.1* | | | | *CNL-FDA.chr5.25049* | *CNL-FvH4.5g23430.1* | *NL-FDA.chr4.05233* | | | | | *CNL-FMA.chr7.880* | | | | | | | | |
| *TNL-FII.chr7.936* | *TNL-FMA.chr2.1405* | | | | *CNL-FDA.chr5.25050* | *CNL-FII.chr5.3063* | *NL-FDA.chr4.05233* | | | | | *CNL-FNU.ctg81.894* | | | | | | | | |
| *TNL-FII.chr7.936* | *TNL-FNG.chr7.552* | | | | *CNL-FDA.chr5.25050* | *CNL-FNG.chr5.2146* | *NL-FDA.chr4.05233* | | | | | *CNL-FvH4.4g29930.1* | | | | | | | | |
| *TNL-FII.chr7.936* | *TNL-FPE.chr2.3663* | | | | *CNL-FDA.chr5.25050* | *CNL-FNU.ctg158.42* | *NL-FDA.chr4.08398* | | | | | *CNL-FII.chr4.2124* | | | | | | | | |
| *TNL-FII.chr7.936* | *TNL-FvH4.7g10750.1* | | | | *CNL-FDA.chr5.25050* | *CNL-FvH4.5g23420.1* | *NL-FDA.chr4.08398* | | | | | *CNL-FMA.chr4.2163* | | | | | | | | |
| *TNL-FMA.chr0.553* | *TNL-FDA.chr1.07161* | | | | *CNL-FDA.chr6.02010* | *CNL-FvH4.6g51610.1* | *NL-FDA.chr4.08398* | | | | | *CNL-FNG.chr4.2225* | | | | | | | | |
| *TNL-FMA.chr0.553* | *TNL-FNU.ctg173.410* | | | | *CNL-FDA.chr6.02056* | *CNL-FNU.ctg129.61* | *NL-FDA.chr4.08398* | | | | | *CNL-FvH4.4g26300.1* | | | | | | | | |
| *TNL-FMA.chr0.553* | *TNL-FvH4.1g22370.1* | | | | *CNL-FDA.chr6.02056* | *CNL-FvH4.3g02290.1* | *NL-FDA.chr4.08398* | | | | | *NL-FNU.ctg82.264* | | | | | | | | |
| *TNL-FMA.chr0.554* | *TNL-FDA.chr1.07161* | | | | *CNL-FDA.chr6.02058* | *CNL-FNU.ctg129.61* | *NL-FDA.chr5.03601* | | | | | *NL-FMA.chr5.2765* | | | | | | | | |
| *TNL-FMA.chr0.554* | *TNL-FvH4.1g22380.1* | | | | *CNL-FDA.chr6.02058* | *CNL-FvH4.7g22030.1* | *NL-FDA.chr5.03601* | | | | | *NL-FNU.ctg161.49* | | | | | | | | |
| *TNL-FMA.chr0.565* | *TNL-FvH4.1g22540.1* | | | | *CNL-FDA.chr6.02198* | *CN-FII.chr6.198* | *NL-FDA.chr5.03601* | | | | | *NL-FvH4.5g33110.1* | | | | | | | | |
| *TNL-FMA.chr1.1780* | *TN-FPE.chr5.3159* | | | | *CNL-FDA.chr6.02198* | *CNL-FNG.chr2.1430* | *NL-FDA.chr5.21327* | | | | | *CNL-FII.chr3.1811* | | | | | | | | |
| *TNL-FMA.chr1.1780* | *TNL-FII.chr5.3371* | | | | *CNL-FDA.chr6.02198* | *CNL-FvH4.4g35200.1* | *NL-FDA.chr6.02338* | | | | | *NL-FvH4.6g04350.1* | | | | | | | | |
| *TNL-FMA.chr1.1780* | *TNL-FvH4.1g15650.1* | | | | *CNL-FDA.chr6.13546* | *CNL-FNU.ctg53.160* | *NL-FDA.chr6.26447* | | | | | *CNL-FMA.chr6.1184* | | | | | | | | |
| *TNL-FMA.chr2.1405* | *TNL-FNU.ctg109.139* | | | | *CNL-FDA.chr6.13546* | *CNL-FvH4.6g48310.1* | *NL-FDA.chr6.26447* | | | | | *CNL-FNU.ctg78.2* | | | | | | | | |
| *TNL-FMA.chr2.1405* | *TNL-FPE.chr2.959* | | | | *CNL-FDA.chr6.13552* | *CN-FMA.chr6.4269* | *NL-FDA.chr6.26447* | | | | | *CNL-FvH4.6g12200.1* | | | | | | | | |
| *TNL-FMA.chr2.1405* | *TNL-FvH4.2g17330.1* | | | | *CNL-FDA.chr6.13552* | *CN-FNU.ctg53.150* | *NL-FDA.chr6.26794* | | | | | *NL-FII.chr6.1892* | | | | | | | | |
| *TNL-FMA.chr2.2218* | *TNL-FNG.chr2.2347* | | | | *CNL-FDA.chr6.13552* | *CNL-FII.chr6.4055* | *NL-FDA.chr6.26794* | | | | | *NL-FMA.chr6.1747* | | | | | | | | |
| *TNL-FMA.chr2.2218* | *TNL-FNU.ctg114.235* | | | | *CNL-FDA.chr6.13552* | *CNL-FNG.chr5.655* | *NL-FDA.chr6.26794* | | | | | *NL-FNG.chr6.2521* | | | | | | | | |
| *TNL-FMA.chr2.2218* | *TNL-FPE.chr5.1409* | | | | *CNL-FDA.chr6.13552* | *CNL-FvH4.6g48220.1* | *NL-FDA.chr6.26794* | | | | | *NL-FNU.ctg72.1* | | | | | | | | |
| *TNL-FMA.chr2.2218* | *TNL-FvH4.2g27070.1* | | | | *CNL-FDA.chr6.13561* | *CNL-FNU.ctg53.139* | *NL-FDA.chr6.26794* | | | | | *NL-FPE.chr0.887* | | | | | | | | |
| *TNL-FMA.chr2.2280* | *TNL-FNG.chr2.2392* | | | | *CNL-FDA.chr6.13561* | *CNL-FvH4.6g48310.1* | *NL-FDA.chr6.26794* | | | | | *NL-FvH4.6g22120.1* | | | | | | | | |
| *TNL-FMA.chr2.2280* | *TNL-FvH4.2g27720.1* | | | | *CNL-FDA.chr6.13872* | *CNL-FNU.ctg129.61* | *NL-FDA.chr7.00705* | | | | | *CNL-FPE.chr7.1571* | | | | | | | | |
| *TNL-FMA.chr2.2281* | *TNL-FNG.chr2.2392* | | | | *CNL-FDA.chr6.13872* | *CNL-FvH4.6g51140.1* | *NL-FDA.chr7.00705* | | | | | *CNL-FvH4.7g27330.1* | | | | | | | | |
| *TNL-FMA.chr2.2281* | *TNL-FvH4.2g27720.1* | | | | *CNL-FDA.chr6.13908* | *CN-FvH4.6g51570.1* | *NL-FDA.chr7.14306* | | | | | *CNL-FII.chr7.210* | | | | | | | | |
| *TNL-FMA.chr2.857* | *TNL-FDA.chr6.02345* | | | | *CNL-FDA.chr6.13967* | *CNL-FvH4.6g52150.1* | *NL-FDA.chr7.14306* | | | | | *CNL-FvH4.7g02450.1* | | | | | | | | |
| *TNL-FMA.chr2.857* | *TNL-FNU.ctg8.247* | | | | *CNL-FDA.chr6.14104* | *CNL-FII.chr3.408* | *NL-FDA.chr7.14797* | | | | | *CN-FPE.chr6.3765* | | | | | | | | |
| *TNL-FMA.chr2.857* | *TNL-FPE.chr3.3124* | | | | *CNL-FDA.chr6.14104* | *CNL-FPE.chr3.535* | *NL-FDA.chr7.14797* | | | | | *CNL-FII.chr7.1027* | | | | | | | | |
| *TNL-FMA.chr2.857* | *TNL-FvH4.2g14320.1* | | | | *CNL-FDA.chr6.14104* | *CNL-FvH4.6g53580.1* | *NL-FDA.chr7.14797* | | | | | *CNL-FMA.chr6.1184* | | | | | | | | |
| *TNL-FMA.chr3.3484* | *TNL-FvH4.3g42940.1* | | | | *CNL-FDA.chr6.17311* | *CNL-FvH4.6g52150.1* | *NL-FDA.chr7.14797* | | | | | *CNL-FNG.chr7.951* | | | | | | | | |
| *TNL-FMA.chr4.2945* | *TNL-FII.chr4.2876* | | | | *CNL-FDA.chr6.26146* | *CNL-FMA.chr6.1184* | *NL-FDA.chr7.14797* | | | | | *CNL-FvH4.6g15250.1* | | | | | | | | |
| *TNL-FMA.chr4.2945* | *TNL-FNG.chr4.3018* | | | | *CNL-FDA.chr6.26146* | *CNL-FvH4.6g15340.1* | *NL-FDA.chr7.18714* | | | | | *CNL-FII.chr7.210* | | | | | | | | |
| *TNL-FMA.chr4.2945* | *TNL-FNU.ctg81.211* | | | | *CNL-FDA.chr6.26153* | *CNL-FII.chr7.1027* | *NL-FDA.chr7.18714* | | | | | *CNL-FvH4.7g02450.1* | | | | | | | | |
| *TNL-FMA.chr4.2946* | *TNL-FNG.chr4.3018* | | | | *CNL-FDA.chr6.26153* | *CNL-FMA.chr6.1508* | *NL-FII.chr1.1268* | | | | | *RNL-FMA.chr1.1806* | | | | | | | | |
| *TNL-FMA.chr4.2946* | *TNL-FNU.ctg81.212* | | | | *CNL-FDA.chr6.26153* | *CNL-FNG.chr6.2420* | *NL-FII.chr1.1268* | | | | | *RNL-FvH4.1g15220.1* | | | | | | | | |
| Orthologous gene pairsof TNL | | | | | Orthologous gene pairs of non-TNL | | | | | | | | | | | | | | |
| ID of sequence 1 | ID of sequence 2 | | | | ID of sequence 1 | ID of sequence 2 | ID of sequence 1 | | | | | | | | ID of sequence 2 | | | | |
| *TNL-FMA.chr4.2946* | *TNL-FvH4.4g35420.1* | | | | *CNL-FDA.chr6.26153* | *CNL-FNU.ctg78.2* | *NL-FII.chr2.452* | | | | | *CNL-FNG.chr2.416* | | | | | | | | |
| *TNL-FMA.chr4.57* | *TNL-FDA.chr3.09523* | | | | *CNL-FDA.chr6.26153* | *CNL-FvH4.6g15250.1* | *NL-FII.chr2.452* | | | | | *CNL-FPE.chr7.1798* | | | | | | | | |
| *TNL-FMA.chr4.57* | *TNL-FII.chr5.3371* | | | | *CNL-FDA.chr6.26337* | *CNL-FMA.chr6.1658* | *NL-FII.chr5.1996* | | | | | *CN-FvH4.6g51570.1* | | | | | | | | |
| *TNL-FMA.chr4.57* | *TNL-FNG.chr4.271* | | | | *CNL-FDA.chr6.26337* | *CNL-FNU.ctg78.2* | *NL-FII.chr5.1996* | | | | | *CNL-FDA.chr6.02010* | | | | | | | | |
| *TNL-FMA.chr4.57* | *TNL-FNU.ctg105.95* | | | | *CNL-FDA.chr6.26337* | *CNL-FvH4.6g13450.1* | *NL-FII.chr5.3064* | | | | | *CNL-FDA.chr5.25049* | | | | | | | | |
| *TNL-FMA.chr4.57* | *TNL-FPE.chr5.1953* | | | | *CNL-FDA.chr7.00307* | *CNL-FII.chr3.408* | *NL-FII.chr5.3064* | | | | | *CNL-FNG.chr5.2147* | | | | | | | | |
| *TNL-FMA.chr4.57* | *TNL-FvH4.4g00790.1* | | | | *CNL-FDA.chr7.00307* | *CNL-FPE.chr3.535* | *NL-FII.chr5.3064* | | | | | *CNL-FNU.ctg158.43* | | | | | | | | |
| *TNL-FMA.chr5.2425* | *TNL-FNG.chr5.2639* | | | | *CNL-FDA.chr7.00307* | *CNL-FvH4.7g31140.1* | *NL-FII.chr5.3064* | | | | | *CNL-FvH4.5g23430.1* | | | | | | | | |
| *TNL-FMA.chr5.2425* | *TNL-FvH4.5g35770.1* | | | | *CNL-FDA.chr7.00809* | *CNL-FNU.ctg129.61* | *NL-FII.chr6.1611* | | | | | *CNL-FNU.ctg82.648* | | | | | | | | |
| *TNL-FMA.chr5.2877* | *TNL-FvH4.1g15650.1* | | | | *CNL-FDA.chr7.00809* | *CNL-FvH4.7g26370.1* | *NL-FII.chr6.1611* | | | | | *CNL-FvH4.4g22940.1* | | | | | | | | |
| *TNL-FMA.chr5.364* | *TNL-FvH4.5g03630.1* | | | | *CNL-FDA.chr7.01119* | *CNL-FNU.ctg129.61* | *NL-FII.chr6.1611* | | | | | *NL-FPE.chr3.3367* | | | | | | | | |
| *TNL-FNG.chr1.1006* | *TNL-FDA.chr7.00159* | | | | *CNL-FDA.chr7.01119* | *CNL-FvH4.7g23500.1* | *NL-FII.chr6.1892* | | | | | *NL-FMA.chr6.1747* | | | | | | | | |
| *TNL-FNG.chr1.1006* | *TNL-FvH4.1g11580.1* | | | | *CNL-FDA.chr7.01192* | *CNL-FNU.ctg129.61* | *NL-FII.chr6.1892* | | | | | *NL-FNG.chr6.2521* | | | | | | | | |
| *TNL-FNG.chr1.1446* | *TNL-FNU.ctg172.194* | | | | *CNL-FDA.chr7.01192* | *CNL-FvH4.7g22030.1* | *NL-FII.chr6.1892* | | | | | *NL-FNU.ctg72.1* | | | | | | | | |
| *TNL-FNG.chr1.1446* | *TNL-FvH4.1g16610.1* | | | | *CNL-FDA.chr7.01250* | *CNL-FNU.ctg129.61* | *NL-FII.chr6.1892* | | | | | *NL-FPE.chr0.887* | | | | | | | | |
| *TNL-FNG.chr2.105* | *TNL-FPE.chr0.1078* | | | | *CNL-FDA.chr7.01250* | *CNL-FvH4.7g22030.1* | *NL-FII.chr6.1892* | | | | | *NL-FvH4.6g22120.1* | | | | | | | | |
| *TNL-FNG.chr2.105* | *TNL-FvH4.2g02050.1* | | | | *CNL-FDA.chr7.01259* | *CNL-FNU.ctg129.61* | *NL-FII.chr6.2634* | | | | | *CNL-FMA.chr6.2723* | | | | | | | | |
| *TNL-FNG.chr2.1702* | *TNL-FNU.ctg135.24* | | | | *CNL-FDA.chr7.01259* | *CNL-FvH4.7g22030.1* | *NL-FII.chr6.2634* | | | | | *CNL-FNG.chr6.2058* | | | | | | | | |
| *TNL-FNG.chr2.1702* | *TNL-FvH4.2g20310.1* | | | | *CNL-FDA.chr7.01379* | *CNL-FNU.ctg129.61* | *NL-FII.chr6.2634* | | | | | *CNL-FNU.ctg64.14.1* | | | | | | | | |
| *TNL-FNG.chr2.1729* | *TNL-FII.chr2.1754* | | | | *CNL-FDA.chr7.01379* | *CNL-FvH4.7g20580.1* | *NL-FII.chr6.2634* | | | | | *CNL-FvH4.6g30120.1* | | | | | | | | |
| *TNL-FNG.chr2.1729* | *TNL-FvH4.2g20310.1* | | | | *CNL-FII.chr1.1071* | *CNL-FMA.chr1.1369* | *NL-FMA.chr1.1431* | | | | | *CNL-FII.chr1.1071* | | | | | | | | |
| *TNL-FNG.chr2.2347* | *TNL-FNU.ctg114.235* | | | | *CNL-FII.chr1.1071* | *CNL-FNU.ctg170.24* | *NL-FMA.chr1.1431* | | | | | *CNL-FNU.ctg170.24* | | | | | | | | |
| *TNL-FNG.chr2.2347* | *TNL-FPE.chr5.1409* | | | | *CNL-FII.chr1.1071* | *CNL-FPE.chr6.1273* | *NL-FMA.chr1.1431* | | | | | *CNL-FPE.chr6.1273* | | | | | | | | |
| *TNL-FNG.chr2.2347* | *TNL-FvH4.2g27070.1* | | | | *CNL-FII.chr1.1071* | *N-FvH4.1g12710.1* | *NL-FMA.chr1.1431* | | | | | *N-FvH4.1g12710.1* | | | | | | | | |
| *TNL-FNG.chr2.2392* | *TNL-FvH4.2g27720.1* | | | | *CNL-FII.chr1.1071* | *NL-FNG.chr1.1121* | *NL-FMA.chr1.1431* | | | | | *NL-FNG.chr1.1121* | | | | | | | | |
| *TNL-FNG.chr3.2352* | *TNL-FvH4.7g12160.1* | | | | *CNL-FII.chr1.1471* | *RNL-FNG.chr6.4113* | *NL-FMA.chr4.1335* | | | | | *CNL-FvH4.4g16700.1* | | | | | | | | |
| *TNL-FNG.chr3.3168* | *TNL-FNU.ctg132.96* | | | | *CNL-FII.chr1.1471* | *RNL-FvH4.1g17470.1* | *NL-FMA.chr4.1335* | | | | | *NL-FNG.chr4.1397* | | | | | | | | |
| *TNL-FNG.chr3.3168* | *TNL-FPE.chr1.1274* | | | | *CNL-FII.chr2.1235* | *CNL-FvH4.2g14930.1* | *NL-FMA.chr4.1335* | | | | | *NL-FNU.ctg84.211* | | | | | | | | |
| *TNL-FNG.chr3.3168* | *TNL-FvH4.3g38670.1* | | | | *CNL-FII.chr3.1384* | *CNL-FMA.chr3.1344* | *NL-FMA.chr4.1335* | | | | | *NL-FPE.chr3.4014* | | | | | | | | |
| *TNL-FNG.chr3.3322* | *TNL-FvH4.5g01270.1* | | | | *CNL-FII.chr3.1384* | *CNL-FNG.chr3.1370* | *NL-FMA.chr5.2765* | | | | | *NL-FNU.ctg161.49* | | | | | | | | |
| *TNL-FNG.chr3.3728* | *TNL-FNU.ctg134.31* | | | | *CNL-FII.chr3.1384* | *CNL-FNU.ctg30.47* | *NL-FMA.chr5.2765* | | | | | *NL-FvH4.5g33110.1* | | | | | | | | |
| *TNL-FNG.chr3.3728* | *TNL-FvH4.2g38620.1* | | | | *CNL-FII.chr3.1384* | *CNL-FPE.chr5.124* | *NL-FMA.chr6.1747* | | | | | *NL-FNG.chr6.2521* | | | | | | | | |
| *TNL-FNG.chr4.271* | *TNL-FDA.chr3.09523* | | | | *CNL-FII.chr3.1385* | *CN-FvH4.3g16720.1* | *NL-FMA.chr6.1747* | | | | | *NL-FNU.ctg72.1* | | | | | | | | |
| *TNL-FNG.chr4.271* | *TNL-FII.chr3.744.2* | | | | *CNL-FII.chr3.1385* | *CNL-FMA.chr3.1344* | *NL-FMA.chr6.1747* | | | | | *NL-FPE.chr0.887* | | | | | | | | |
| *TNL-FNG.chr4.271* | *TNL-FNU.ctg105.95* | | | | *CNL-FII.chr3.1385* | *CNL-FNG.chr1.545* | *NL-FMA.chr6.1747* | | | | | *NL-FvH4.6g22120.1* | | | | | | | | |
| *TNL-FNG.chr4.271* | *TNL-FPE.chr5.1953* | | | | *CNL-FII.chr3.1385* | *CNL-FNU.ctg165.171* | *NL-FNG.chr1.1121* | | | | | *CNL-FNU.ctg170.24* | | | | | | | | |
| *TNL-FNG.chr4.271* | *TNL-FvH4.4g00790.1* | | | | *CNL-FII.chr3.1385* | *CNL-FPE.chr5.124* | *NL-FNG.chr1.1121* | | | | | *CNL-FPE.chr6.1273* | | | | | | | | |
| *TNL-FNG.chr4.3018* | *TNL-FNU.ctg81.211* | | | | *CNL-FII.chr3.408* | *CNL-FPE.chr3.535* | *NL-FNG.chr1.1121* | | | | | *N-FvH4.1g12710.1* | | | | | | | | |
| *TNL-FNG.chr5.112* | *TNL-FII.chr3.3603* | | | | *CNL-FII.chr3.408* | *CNL-FvH4.3g04780.1* | *NL-FNG.chr3.2989* | | | | | *CNL-FII.chr7.998* | | | | | | | | |
| *TNL-FNG.chr5.112* | *TNL-FvH4.5g01270.1* | | | | *CNL-FII.chr3.77* | *CNL-FMA.chr3.75* | *NL-FNG.chr3.2989* | | | | | *CNL-FMA.chr3.2744* | | | | | | | | |
| *TNL-FNG.chr5.2639* | *TNL-FvH4.5g35770.1* | | | | *CNL-FII.chr3.77* | *CNL-FNG.chr3.84* | *NL-FNG.chr3.2989* | | | | | *CNL-FvH4.7g11380.1* | | | | | | | | |
| *TNL-FNG.chr5.3204* | *TNL-FDA.chr6.02439* | | | | *CNL-FII.chr3.77* | *CNL-FNU.ctg47.220* | *NL-FNG.chr3.2989* | | | | | *NL-FNU.ctg132.328* | | | | | | | | |
| Orthologous gene pairsof TNL | | | | | Orthologous gene pairs of non-TNL | | | | | | | | | | | | | | | |
| ID of sequence 1 | ID of sequence 2 | | | | ID of sequence 1 | ID of sequence 2 | | ID of sequence 1 | | | | | | | | | ID of sequence 2 | | | |
| *TNL-FNG.chr5.3204* | *TNL-FPE.chr5.2900* | | | | *CNL-FII.chr3.77* | *CNL-FvH4.3g00820.1* | *NL-FNG.chr3.522* | | | | | *NL-FPE.chr3.611* | | | | | | | | |
| *TNL-FNG.chr5.3204* | *TNL-FvH4.5g27640.1* | | | | *CNL-FII.chr3.951* | *CNL-FvH4.3g11490.1* | *NL-FNG.chr4.1397* | | | | | *CNL-FvH4.4g16700.1* | | | | | | | | |
| *TNL-FNG.chr6.2320* | *TNL-FDA.chr2.20071* | | | | *CNL-FII.chr3.951* | *N-FPE.chr3.1891* | *NL-FNG.chr4.1397* | | | | | *NL-FNU.ctg84.211* | | | | | | | | |
| *TNL-FNG.chr6.2320* | *TNL-FMA.chr5.2425* | | | | *CNL-FII.chr3.953* | *CNL-FvH4.3g11490.1* | *NL-FNG.chr4.1397* | | | | | *NL-FPE.chr3.4014* | | | | | | | | |
| *TNL-FNG.chr6.2320* | *TNL-FPE.chr5.1409* | | | | *CNL-FII.chr3.954* | *CNL-FvH4.3g11490.1* | *NL-FNG.chr5.1946* | | | | | *N-FvH4.5g21070.1* | | | | | | | | |
| *TNL-FNG.chr6.2320* | *TNL-FvH4.5g35770.1* | | | | *CNL-FII.chr3.954* | *N-FPE.chr3.1895* | *NL-FNG.chr5.800* | | | | | *CNL-FII.chr7.998* | | | | | | | | |
| *TNL-FNG.chr6.244* | *TNL-FII.chr7.1130* | | | | *CNL-FII.chr3.956* | *CNL-FvH4.3g11490.1* | *NL-FNG.chr5.800* | | | | | *CNL-FvH4.4g16700.1* | | | | | | | | |
| *TNL-FNG.chr6.244* | *TNL-FMA.chr5.2425* | | | | *CNL-FII.chr4.1300* | *CNL-FvH4.4g16700.1* | *NL-FNG.chr5.800* | | | | | *NL-FMA.chr4.1335* | | | | | | | | |
| *TNL-FNG.chr6.244* | *TNL-FvH4.6g03250.1* | | | | *CNL-FII.chr4.1300* | *NL-FMA.chr4.1335* | *NL-FNG.chr6.2521* | | | | | *NL-FNU.ctg72.1* | | | | | | | | |
| *TNL-FNG.chr7.2518* | *TNL-FDA.chr3.05838* | | | | *CNL-FII.chr4.1300* | *NL-FNG.chr4.1397* | *NL-FNG.chr6.2521* | | | | | *NL-FPE.chr0.887* | | | | | | | | |
| *TNL-FNG.chr7.2518* | *TNL-FNU.ctg132.96* | | | | *CNL-FII.chr4.1300* | *NL-FNU.ctg84.211* | *NL-FNG.chr6.2521* | | | | | | | | *NL-FvH4.6g22120.1* | | | | | |
| *TNL-FNG.chr7.2518* | *TNL-FPE.chr1.1274* | | | | *CNL-FII.chr4.1300* | *NL-FPE.chr3.4014* | *NL-FNG.chr7.1223* | | | | | | | | *CNL-FII.chr7.789* | | | | | |
| *TNL-FNG.chr7.2518* | *TNL-FvH4.7g31110.1* | | | | *CNL-FII.chr4.1808* | *CN-FPE.chr1.928* | *NL-FNG.chr7.1223* | | | | | | | | *CNL-FPE.chr7.1124* | | | | | |
| *TNL-FNG.chr7.267* | *TNL-FDA.chr3.20725* | | | | *CNL-FII.chr4.1808* | *CNL-FNG.chr4.1917* | *NL-FNG.chr7.1223* | | | | | | | | *CNL-FvH4.7g15190.1* | | | | | |
| *TNL-FNG.chr7.267* | *TNL-FII.chr2.1158* | | | | *CNL-FII.chr4.1808* | *CNL-FNU.ctg82.648* | *NL-FNG.chr7.723* | | | | | | | | *CNL-FPE.chr0.563* | | | | | |
| *TNL-FNG.chr7.267* | *TNL-FMA.chr1.1780* | | | | *CNL-FII.chr4.1808* | *CNL-FvH4.4g22940.1* | *NL-FNG.chr7.723* | | | | | | | | *CNL-FvH4.7g08100.1* | | | | | |
| *TNL-FNG.chr7.267* | *TNL-FNU.ctg105.95* | | | | *CNL-FII.chr4.1849.2* | *CN-FPE.chr6.878* | *NL-FNU.ctg131.54* | | | | | | | | *CNL-FII.chr7.789* | | | | | |
| *TNL-FNG.chr7.267* | *TNL-FPE.chr3.552* | | | | *CNL-FII.chr4.1849.2* | *CNL-FNU.ctg82.648* | *NL-FNU.ctg131.54* | | | | | | | | *CNL-FPE.chr7.1124* | | | | | |
| *TNL-FNG.chr7.267* | *TNL-FvH4.7g04050.1* | | | | *CNL-FII.chr4.1849.2* | *CNL-FvH4.4g22940.1* | *NL-FNU.ctg131.54* | | | | | | | | *NL-FNG.chr7.1223* | | | | | |
| *TNL-FNG.chr7.552* | *TNL-FMA.chr2.1405* | | | | *CNL-FII.chr4.2076* | *CNL-FMA.chr4.2118* | *NL-FNU.ctg134.34* | | | | | | | | *CNL-FII.chr7.998* | | | | | |
| *TNL-FNG.chr7.552* | *TNL-FNU.ctg134.120* | | | | *CNL-FII.chr4.2076* | *CNL-FNU.ctg82.313* | *NL-FNU.ctg134.34* | | | | | | | | *CNL-FMA.chr7.880* | | | | | |
| *TNL-FNG.chr7.552* | *TNL-FvH4.7g10520.1* | | | | *CNL-FII.chr4.2076* | *CNL-FPE.chr3.1482* | *NL-FNU.ctg134.34* | | | | | | | | *CNL-FPE.chr7.1015* | | | | | |
| *TNL-FNG.chr7.927* | *TNL-FII.chr2.55* | | | | *CNL-FII.chr4.2076* | *CNL-FvH4.4g25750.1* | *NL-FNU.ctg134.34* | | | | | | | | *CNL-FvH4.7g11380.1* | | | | | |
| *TNL-FNG.chr7.927* | *TNL-FNU.ctg134.31* | | | | *CNL-FII.chr4.2124* | *CNL-FMA.chr4.2163* | *NL-FNU.ctg144.28* | | | | | | | | *CNL-FII.chr7.257* | | | | | |
| *TNL-FNG.chr7.927* | *TNL-FvH4.2g38620.1* | | | | *CNL-FII.chr4.2124* | *CNL-FNG.chr4.2225* | *NL-FNU.ctg144.28* | | | | | | | | *CNL-FNG.chr7.204* | | | | | |
| *TNL-FNU.ctg101.35* | *TNL-FMA.chr0.565* | | | | *CNL-FII.chr4.2124* | *CNL-FvH4.4g26300.1* | *NL-FNU.ctg144.28* | | | | | | | | *CNL-FPE.chr6.1639* | | | | | |
| *TNL-FNU.ctg101.35* | *TNL-FvH4.1g22540.1* | | | | *CNL-FII.chr4.2124* | *NL-FNU.ctg82.264* | *NL-FNU.ctg144.28* | | | | | | | | *CNL-FvH4.7g02890.1* | | | | | |
| *TNL-FNU.ctg105.95* | *TNL-FII.chr5.3371* | | | | *CNL-FII.chr4.377* | *CNL-FMA.chr4.679* | *NL-FNU.ctg155.6* | | | | | | | | *CNL-FDA.chr5.23812* | | | | | |
| *TNL-FNU.ctg105.95* | *TNL-FvH4.3g44370.1* | | | | *CNL-FII.chr4.377* | *CNL-FNG.chr0.411* | *NL-FNU.ctg155.6* | | | | | *CNL-FMA.chr4.679* | | | | | | | | |
| *TNL-FNU.ctg109.139* | *TNL-FII.chr7.936* | | | | *CNL-FII.chr4.377* | *CNL-FvH4.4g06030.1* | *NL-FNU.ctg161.49* | | | | | *CNL-FDA.chr3.16699* | | | | | | | | |
| *TNL-FNU.ctg129.394* | *TNL-FPE.chr7.2368* | | | | *CNL-FII.chr5.2001* | *CNL-FDA.chr5.25050* | *NL-FNU.ctg163.12* | | | | | *CNL-FDA.chr1.06545* | | | | | | | | |
| *TNL-FNU.ctg129.394* | *TNL-FvH4.7g17700.1* | | | | *CNL-FII.chr5.2001* | *CNL-FNG.chr5.2146* | *NL-FNU.ctg163.12* | | | | | *CNL-FMA.chr7.880* | | | | | | | | |
| *TNL-FNU.ctg134.120* | *TNL-FPE.chr2.3663* | | | | *CNL-FII.chr5.2001* | *CNL-FNU.ctg158.42* | *NL-FNU.ctg81.827* | | | | | *CNL-FMA.chr3.2744* | | | | | | | | |
| *TNL-FNU.ctg134.16* | *TNL-FDA.chr7.01621* | | | | *CNL-FII.chr5.2001* | *CNL-FvH4.5g23390.1* | *NL-FNU.ctg81.827* | | | | | *CNL-FvH4.4g29930.1* | | | | | | | | |
| *TNL-FNU.ctg134.16* | *TNL-FPE.chr7.2368* | | | | *CNL-FII.chr5.2002* | *CNL-FDA.chr5.25050* | *NL-FNU.ctg81.827* | | | | | *NL-FDA.chr4.05233* | | | | | | | | |
| *TNL-FNU.ctg134.31* | *TNL-FPE.chr7.2368* | | | | *CNL-FII.chr5.2002* | *CNL-FNG.chr5.2145* | *NL-FPE.chr0.1234* | | | | | *CNL-FNU.ctg114.210* | | | | | | | | |
| *TNL-FNU.ctg135.24* | *TNL-FMA.chr2.1405* | | | | *CNL-FII.chr5.2002* | *CNL-FNU.ctg158.40* | *NL-FPE.chr0.1234* | | | | | *CNL-FvH4.6g49940.1* | | | | | | | | |
| *TNL-FNU.ctg160.483* | *TNL-FDA.chr5.03613* | | | | *CNL-FII.chr5.2002* | *CNL-FPE.chr4.1619* | *NL-FPE.chr0.887* | | | | | *NL-FNU.ctg72.1* | | | | | | | | |
| *TNL-FNU.ctg160.483* | *TNL-FII.chr5.2851* | | | | *CNL-FII.chr5.2002* | *CNL-FvH4.5g23400.1* | *NL-FPE.chr0.887* | | | | | *NL-FvH4.6g22120.1* | | | | | | | | |
| *TNL-FNU.ctg160.483* | *TNL-FvH4.5g32970.1* | | | | *CNL-FII.chr5.2003* | *CNL-FDA.chr5.25050* | *NL-FPE.chr2.4140* | | | | | *RNL-FDA.chr2.11777* | | | | | | | | |
| *TNL-FNU.ctg3.21* | *TNL-FDA.chr3.20719* | | | | *CNL-FII.chr5.2003* | *CNL-FNG.chr5.2146* | *NL-FPE.chr2.4140* | | | | | *RNL-FII.chr2.3467* | | | | | | | | |
| *TNL-FNU.ctg3.25* | *TNL-FDA.chr3.20719* | | | | *CNL-FII.chr5.2003* | *CNL-FNU.ctg158.42* | *NL-FPE.chr2.4140* | | | | | *RNL-FMA.chr2.3489* | | | | | | | | |
| Orthologous gene pairsof TNL | | | | | Orthologous gene pairs of non-TNL | | | | | | | | | | | | | | | |
| ID of sequence 1 | ID of sequence 2 | | | | ID of sequence 1 | ID of sequence 2 | | ID of sequence 1 | | | | | | | | | ID of sequence 2 | | | |
| *TNL-FNU.ctg3.25* | *TNL-FvH4.3g44370.1* | | | | *CNL-FII.chr5.2003* | *CNL-FvH4.5g23420.1* | *NL-FPE.chr2.4140* | | | | | *RNL-FNG.chr2.3537* | | | | | | | | |
| *TNL-FNU.ctg3.73* | *TNL-FNG.chr7.2518* | | | | *CNL-FII.chr5.2004* | *CNL-FNG.chr5.2147* | *NL-FPE.chr2.4140* | | | | | *RNL-FNU.ctg122.114* | | | | | | | | |
| *TNL-FNU.ctg3.75* | *TNL-FNG.chr3.3322* | | | | *CNL-FII.chr5.2004* | *CNL-FNU.ctg158.43* | *NL-FPE.chr2.4140* | | | | | *RNL-FvH4.2g40720.1* | | | | | | | | |
| *TNL-FNU.ctg3.77* | *TNL-FvH4.3g44000.1* | | | | *CNL-FII.chr5.2004* | *CNL-FvH4.5g23430.1* | *NL-FPE.chr3.3367* | | | | | *CNL-FII.chr4.1808* | | | | | | | | |
| *TNL-FNU.ctg32.58* | *TNL-FMA.chr2.2281* | | | | *CNL-FII.chr5.2006* | *CNL-FNG.chr5.2149* | *NL-FPE.chr3.3367* | | | | | *CNL-FNG.chr4.1917* | | | | | | | | |
| *TNL-FNU.ctg56.77* | *TNL-FII.chr1.1394* | | | | *CNL-FII.chr5.2006* | *CNL-FNU.ctg158.45* | *NL-FPE.chr3.3367* | | | | | *CNL-FNU.ctg82.648* | | | | | | | | |
| *TNL-FNU.ctg56.77* | *TNL-FvH4.1g16650.1* | | | | *CNL-FII.chr5.2006* | *CNL-FvH4.5g23450.1* | *NL-FPE.chr3.3367* | | | | | *CNL-FvH4.4g22940.1* | | | | | | | | |
| *TNL-FNU.ctg8.247* | *TNL-FII.chr6.322* | | | | *CNL-FII.chr5.2007* | *CNL-FDA.chr5.25047* | *NL-FPE.chr3.4014* | | | | | *CNL-FvH4.4g16700.1* | | | | | | | | |
| *TNL-FNU.ctg81.212* | *TNL-FNG.chr4.3018* | | | | *CNL-FII.chr5.2007* | *CNL-FNG.chr5.2149* | *NL-FPE.chr3.4014* | | | *NL-FNU.ctg84.211* | | | | | | | | | | |
| *TNL-FNU.ctg81.216* | *TNL-FII.chr4.2876* | | | | *CNL-FII.chr5.2007* | *CNL-FNU.ctg158.46* | *NL-FPE.chr7.793* | | | *CNL-FII.chr7.789* | | | | | | | | | | |
| *TNL-FNU.ctg81.216* | *TNL-FMA.chr4.2946* | | | | *CNL-FII.chr5.2007* | *CNL-FvH4.5g23450.1* | *NL-FPE.chr7.793* | | | | | | | | *CNL-FvH4.7g08870.1* | | | | | |
| *TNL-FNU.ctg81.216* | *TNL-FNG.chr4.3018* | | | | *CNL-FII.chr5.2008* | *CNL-FNG.chr5.2150* | *NL-FPE.chr7.793* | | | | | | | | *NL-FNG.chr7.1223* | | | | | |
| *TNL-FPE.chr0.1078* | *TNL-FvH4.2g02050.1* | | | | *CNL-FII.chr5.2008* | *CNL-FNU.ctg158.48* | *NL-FvH4.1g22800.1* | | | | | | | | *CNL-FDA.chr6.02010* | | | | | |
| *TNL-FPE.chr1.1274* | *TNL-FDA.chr3.05837* | | | | *CNL-FII.chr5.2008* | *CNL-FvH4.5g23470.1* | *NL-FvH4.3g35890.1* | | | | | | | | *CNL-FII.chr3.1811* | | | | | |
| *TNL-FPE.chr1.1274* | *TNL-FNU.ctg132.96* | | | | *CNL-FII.chr5.3003* | *CNL-FMA.chr4.2118* | *NL-FvH4.4g15190.1* | | | | | | | | *CN-FII.chr1.1941* | | | | | |
| *TNL-FPE.chr1.1274* | *TNL-FvH4.7g06680.1* | | | | *CNL-FII.chr5.3003* | *CNL-FNG.chr5.2999* | *NL-FvH4.4g15190.1* | | | | | | | | *CN-FPE.chr7.1432* | | | | | |
| *TNL-FPE.chr2.3663* | *TNL-FMA.chr2.1405* | | | | *CNL-FII.chr5.3003* | *CNL-FNU.ctg82.313* | *NL-FvH4.4g15190.1* | | | | | | | | *CNL-FDA.chr6.02010* | | | | | |
| *TNL-FPE.chr2.3663* | *TNL-FvH4.7g10750.1* | | | | *CNL-FII.chr5.3003* | *CNL-FPE.chr1.2047* | *NL-FvH4.4g18270.1* | | | | | | | | *CNL-FMA.chr6.1508* | | | | | |
| *TNL-FPE.chr2.91* | *TNL-FvH4.7g12160.1* | | | | *CNL-FII.chr5.3003* | *CNL-FvH4.5g34680.1* | *NL-FvH4.4g18270.1* | | | | | | | | *CNL-FNU.ctg78.2* | | | | | |
| *TNL-FPE.chr2.959* | *TNL-FII.chr7.936* | | | | *CNL-FII.chr5.3063* | *CNL-FNG.chr5.2146* | *NL-FvH4.4g18270.1* | | | | | | | | *NL-FDA.chr6.26447* | | | | | |
| *TNL-FPE.chr2.959* | *TNL-FNU.ctg109.139* | | | | *CNL-FII.chr5.3063* | *CNL-FNU.ctg158.42* | *NL-FvH4.5g33110.1* | | | | | | | | *NL-FNU.ctg161.49* | | | | | |
| *TNL-FPE.chr2.959* | *TNL-FvH4.2g17330.1* | | | | *CNL-FII.chr5.3063* | *CNL-FvH4.5g23420.1* | *NL-FvH4.6g05490.1* | | | | | | | | *NL-FMA.chr2.856* | | | | | |
| *TNL-FPE.chr3.3124* | *TNL-FDA.chr6.02345* | | | | *CNL-FII.chr6.2256* | *CNL-FDA.chr5.03688* | *NL-FvH4.6g22120.1* | | | | | | | | *NL-FNU.ctg72.1* | | | | | |
| *TNL-FPE.chr3.3124* | *TNL-FII.chr6.322* | | | | *CNL-FII.chr6.2256* | *CNL-FvH4.3g02290.1* | *NL-FVI.CHR2.81* | | | | | | | | *CNL-FPE.chr5.3277* | | | | | |
| *TNL-FPE.chr3.3124* | *TNL-FNU.ctg8.247* | | | | *CNL-FII.chr6.2546* | *CNL-FDA.chr5.03688* | *NL-FVI.CHR2.81* | | | | | | | | *CNL-FvH4.2g00960.1* | | | | | |
| *TNL-FPE.chr3.3124* | *TNL-FvH4.6g05610.1* | | | | *CNL-FII.chr6.2546* | *CNL-FvH4.3g02290.1* | *NL-FVI.CHR2.81* | | | | | | | | *N-FII.chr2.79* | | | | | |
| *TNL-FPE.chr3.552* | *TNL-FII.chr5.3371* | | | | *CNL-FII.chr6.4055* | *CN-FMA.chr6.4269* | *NL-FVI.CHR3.187* | | | | | | | | *CN-FvH4.3g16720.1* | | | | | |
| *TNL-FPE.chr3.552* | *TNL-FMA.chr5.2877* | | | | *CNL-FII.chr6.4055* | *CN-FNU.ctg53.150* | *NL-FVI.CHR3.187* | | | | | | | | *CNL-FII.chr3.1385* | | | | | |
| *TNL-FPE.chr3.552* | *TNL-FvH4.7g04050.1* | | | | *CNL-FII.chr6.4055* | *CNL-FNG.chr5.655* | *NL-FVI.CHR3.187* | | | | | *CNL-FMA.chr3.1344* | | | | | | | | |
| *TNL-FPE.chr5.1409* | *TNL-FNU.ctg114.235* | | | | *CNL-FII.chr6.4055* | *CNL-FvH4.6g48220.1* | *NL-FVI.CHR3.187* | | | | | *CNL-FNG.chr1.545* | | | | | | | | |
| *TNL-FPE.chr5.1409* | *TNL-FvH4.2g27070.1* | | | | *CNL-FII.chr7.1027* | *CN-FPE.chr6.3765* | *NL-FVI.CHR3.187* | | | | | *CNL-FNU.ctg165.171* | | | | | | | | |
| *TNL-FPE.chr5.1953* | *TN-FNG.chr4.253* | | | | *CNL-FII.chr7.1027* | *CNL-FMA.chr6.1184* | *NL-FVI.CHR3.187* | | | | | *CNL-FPE.chr5.124* | | | | | | | | |
| *TNL-FPE.chr5.1953* | *TNL-FDA.chr3.09523* | | | | *CNL-FII.chr7.1027* | *CNL-FNG.chr7.951* | *NL-FVI.CHR3.187* | | | | | *NL-FDA.chr3.21153* | | | | | | | | |
| *TNL-FPE.chr5.1953* | *TNL-FII.chr3.744.2* | | | | *CNL-FII.chr7.1027* | *CNL-FNU.ctg132.293* | *NL-FVI.CHR4.1309* | | | | | *CNL-FDA.chr6.02010* | | | | | | | | |
| *TNL-FPE.chr5.1953* | *TNL-FNU.ctg105.95* | | | | *CNL-FII.chr7.1027* | *CNL-FvH4.6g12200.1* | *NL-FVI.CHR4.1309* | | | | | *CNL-FvH4.6g51610.1* | | | | | | | | |
| *TNL-FPE.chr5.1953* | *TNL-FvH4.4g00790.1* | | | | *CNL-FII.chr7.162.3* | *CNL-FNG.chr7.204* | *NL-FVI.CHR4.1998* | | | | | *CNL-FII.chr4.1808* | | | | | | | | |
| *TNL-FPE.chr5.2900* | *TNL-FII.chr3.3626* | | | | *CNL-FII.chr7.162.3* | *CNL-FNU.ctg146.100* | *NL-FVI.CHR4.1998* | | | | | *CNL-FNG.chr4.1917* | | | | | | | | |
| *TNL-FPE.chr5.2900* | *TNL-FNG.chr7.2518* | | | | *CNL-FII.chr7.162.3* | *CNL-FvH4.7g01950.1* | *NL-FVI.CHR4.1998* | | | | | *CNL-FNU.ctg82.648* | | | | | | | | |
| *TNL-FPE.chr5.2900* | *TNL-FvH4.5g27640.1* | | | | *CNL-FII.chr7.186* | *CNL-FPE.chr7.237* | *NL-FVI.CHR4.1998* | | | | | *CNL-FvH4.4g22940.1* | | | | | | | | |
| *TNL-FPE.chr6.1904* | *TNL-FvH4.3g10020.1* | | | | *CNL-FII.chr7.186* | *CNL-FvH4.7g02450.1* | *NL-FVI.CHR4.1998* | | | | | *NL-FPE.chr3.3367* | | | | | | | | |
| *TNL-FPE.chr6.5657* | *TNL-FMA.chr0.565* | | | | *CNL-FII.chr7.206* | *CNL-FPE.chr7.1571* | *NL-FVI.CHR4.2250* | | | | | *CNL-FII.chr4.2076* | | | | | | | | |
| *TNL-FPE.chr6.5657* | *TNL-FNU.ctg101.35* | | | | *CNL-FII.chr7.206* | *CNL-FvH4.7g02400.1* | *NL-FVI.CHR4.2250* | | | | | *CNL-FMA.chr4.2118* | | | | | | | | |
| Orthologous gene pairsof TNL | | | | | Orthologous gene pairs of non-TNL | | | | | | | | | | | | | | | |
| ID of sequence 1 | ID of sequence 2 | | | | ID of sequence 1 | ID of sequence 2 | | ID of sequence 1 | | | | | | ID of sequence 2 | | | | | | |
| *TNL-FPE.chr6.5657* | *TNL-FvH4.1g22390.1* | | | | *CNL-FII.chr7.207* | *CNL-FPE.chr7.1571* | *NL-FVI.CHR4.2250* | | | | | *CNL-FNG.chr5.2999* | | | | | | | | |
| *TNL-FPE.chr7.2368* | *TNL-FvH4.7g28550.1* | | | | *CNL-FII.chr7.207* | *CNL-FvH4.7g02450.1* | *NL-FVI.CHR4.2250* | | | | | *CNL-FNU.ctg82.313* | | | | | | | | |
| *TNL-FvH4.1g01230.1* | *TNL-FNU.ctg162.30* | | | | *CNL-FII.chr7.210* | *CNL-FPE.chr7.1571* | *NL-FVI.CHR4.2250* | | | | | *CNL-FPE.chr3.1482* | | | | | | | | |
| *TNL-FvH4.1g07020.1* | *TNL-FMA.chr0.565* | | | | *CNL-FII.chr7.210* | *CNL-FvH4.7g02450.1* | *NL-FVI.CHR4.2250* | | | | | *CNL-FvH4.4g25750.1* | | | | | | | | |
| *TNL-FvH4.1g07020.1* | *TNL-FNU.ctg173.410* | | | | *CNL-FII.chr7.257* | *CNL-FNG.chr7.204* | *NL-FVI.CHR6.1794* | | | | | *CNL-FMA.chr6.1508* | | | | | | | | |
| *TNL-FvH4.1g11570.1* | *TNL-FDA.chr7.00159* | | | | *CNL-FII.chr7.257* | *CNL-FNU.ctg145.8* | *NL-FVI.CHR6.1794* | | | | | *CNL-FNU.ctg78.2* | | | | | | | | |
| *TNL-FvH4.1g11570.1* | *TNL-FNG.chr1.1006* | | | | *CNL-FII.chr7.257* | *CNL-FPE.chr6.1639* | *NL-FVI.CHR6.1794* | | | | | *CNL-FvH4.6g15090.1* | | | | | | | | |
| *TNL-FvH4.1g11580.1* | *TNL-FDA.chr7.00159* | | | | *CNL-FII.chr7.257* | *CNL-FvH4.7g02890.1* | *NL-FVI.CHR6.1794* | | | | | *NL-FDA.chr6.26447* | | | | | | | | |
| *TNL-FvH4.1g16600.1* | *TNL-FNG.chr1.1446* | | | | *CNL-FII.chr7.55* | *CNL-FPE.chr7.237* | *NL-FVI.CHR6.2190* | | | | | *NL-FDA.chr6.26794* | | | | | | | | |
| *TNL-FvH4.1g16600.1* | *TNL-FNU.ctg172.194* | | | | *CNL-FII.chr7.55* | *NL-FvH4.7g00850.1* | *NL-FVI.CHR6.2190* | | | | | *NL-FII.chr6.1892* | | | | | | | | |
| *TNL-FvH4.1g16610.1* | *TNL-FDA.chr1.17978* | | | | *CNL-FII.chr7.708* | *CNL-FNU.ctg114.210* | *NL-FVI.CHR6.2190* | | | | | *NL-FMA.chr6.1747* | | | | | | | | |
| *TNL-FvH4.1g16610.1* | *TNL-FII.chr1.1393* | | | | *CNL-FII.chr7.708* | *CNL-FPE.chr0.563* | *NL-FVI.CHR6.2190* | | | | | *NL-FNG.chr6.2521* | | | | | | | | |
| *TNL-FvH4.1g16610.1* | *TNL-FNU.ctg172.194* | | | | *CNL-FII.chr7.708* | *CNL-FvH4.7g08100.1* | *NL-FVI.CHR6.2190* | | | | | | | | *NL-FNU.ctg72.1* | | | | | |
| *TNL-FvH4.1g16620.1* | *TNL-FNU.ctg172.195* | | | | *CNL-FII.chr7.708* | *NL-FNG.chr7.723* | *NL-FVI.CHR6.2190* | | | | | | | | *NL-FPE.chr0.887* | | | | | |
| *TNL-FvH4.1g16640.1* | *TNL-FDA.chr1.17981* | | | | *CNL-FII.chr7.789* | *CNL-FNU.ctg114.210* | *NL-FVI.CHR6.2190* | | | | | | | | *NL-FvH4.6g22120.1* | | | | | |
| *TNL-FvH4.1g16640.1* | *TNL-FNU.ctg172.195* | | | | *CNL-FII.chr7.789* | *CNL-FvH4.7g08870.1* | *NL-FVI.CHR7.2360* | | | | | | | | *NL-FPE.chr7.2369* | | | | | |
| *TNL-FvH4.1g16650.1* | *TNL-FNU.ctg172.196* | | | | *CNL-FII.chr7.789* | *NL-FPE.chr0.1234* | *NL-FVI.CHR7.880* | | | | | | | | *NL-FPE.chr2.1814* | | | | | |
| *TNL-FvH4.1g22370.1* | *TNL-FDA.chr1.07161* | | | | *CNL-FII.chr7.998* | *CNL-FMA.chr7.880* | *RN-FDA.chr1.22178* | | | | | | | | *CNL-FNU.ctg172.27* | | | | | |
| *TNL-FvH4.1g22370.1* | *TNL-FNU.ctg173.410* | | | | *CNL-FII.chr7.998* | *CNL-FvH4.7g11380.1* | *RN-FDA.chr1.22178* | | | | | | | | *RNL-FII.chr1.1265* | | | | | |
| *TNL-FvH4.1g22380.1* | *TNL-FMA.chr0.565* | | | | *CNL-FII.chr7.998* | *NL-FNU.ctg132.328* | *RN-FDA.chr1.22178* | | | | | | | | *RNL-FMA.chr1.1808* | | | | | |
| *TNL-FvH4.1g22380.1* | *TNL-FNU.ctg173.410* | | | | *CNL-FMA.chr1.1369* | *CNL-FNU.ctg170.24* | *RN-FDA.chr1.22178* | | | | | | | | *RNL-FvH4.1g15120.1* | | | | | |
| *TNL-FvH4.1g22390.1* | *TNL-FDA.chr1.07161* | | | | *CNL-FMA.chr1.1369* | *CNL-FPE.chr6.1273* | *RN-FDA.chr1.22186* | | | | | | | | *N-FNU.ctg172.28* | | | | | |
| *TNL-FvH4.1g22390.1* | *TNL-FMA.chr0.565* | | | | *CNL-FMA.chr1.1369* | *N-FvH4.1g12710.1* | *RN-FDA.chr1.22186* | | | | | | | | *RNL-FII.chr1.1266* | | | | | |
| *TNL-FvH4.1g22540.1* | *TNL-FDA.chr1.07161* | | | | *CNL-FMA.chr1.1369* | *NL-FNG.chr1.1121* | *RN-FDA.chr1.22186* | | | | | | | | *RNL-FMA.chr1.1807* | | | | | |
| *TNL-FvH4.2g00540.1* | *TNL-FDA.chr7.00159* | | | | *CNL-FMA.chr2.328* | *CN-FII.chr2.469* | *RN-FDA.chr1.22186* | | | | | | | | *RNL-FvH4.1g15130.1* | | | | | |
| *TNL-FvH4.2g00540.1* | *TNL-FII.chr3.1830* | | | | *CNL-FMA.chr2.328* | *CN-FvH4.2g05720.1* | *RN-FDA.chr6.13755* | | | | | | | | *RN-FNU.ctg52.52* | | | | | |
| *TNL-FvH4.2g00540.1* | *TNL-FPE.chr6.5657* | | | | *CNL-FMA.chr2.328* | *CNL-FNG.chr2.416* | *RN-FDA.chr6.13755* | | | | | | | | *RNL-FNG.chr6.4113* | | | | | |
| *TNL-FvH4.2g00550.1* | *TNL-FDA.chr7.00189* | | | | *CNL-FMA.chr2.328* | *CNL-FNU.ctg104.183* | *RN-FDA.chr6.13755* | | | | | | | | *RNL-FPE.chr5.2037* | | | | | |
| *TNL-FvH4.2g14320.1* | *TNL-FDA.chr6.02345* | | | | *CNL-FMA.chr2.328* | *CNL-FPE.chr7.1798* | *RN-FDA.chr6.13755* | | | | | | | | *RNL-FvH4.6g50310.1* | | | | | |
| *TNL-FvH4.2g14320.1* | *TNL-FNU.ctg8.247* | | | | *CNL-FMA.chr3.1344* | *CN-FvH4.3g16720.1* | *RN-FII.chr3.1087* | | | | | | | | *N-FPE.chr6.5282* | | | | | |
| *TNL-FvH4.2g14320.1* | *TNL-FPE.chr3.3124* | | | | *CNL-FMA.chr3.1344* | *CNL-FNG.chr1.545* | *RN-FII.chr3.1087* | | | | | | | | *RN-FDA.chr6.13755* | | | | | |
| *TNL-FvH4.2g17330.1* | *TNL-FII.chr7.936* | | | | *CNL-FMA.chr3.1344* | *CNL-FNU.ctg165.171* | *RN-FII.chr3.1087* | | | | | | | | *RN-FNU.ctg52.52* | | | | | |
| *TNL-FvH4.2g17330.1* | *TNL-FNU.ctg109.139* | | | | *CNL-FMA.chr3.1344* | *CNL-FPE.chr5.124* | *RN-FII.chr3.1087* | | | | | | | | *RNL-FvH4.6g50310.1* | | | | | |
| *TNL-FvH4.2g27070.1* | *TNL-FNU.ctg114.235* | | | | *CNL-FMA.chr3.2744* | *CNL-FII.chr7.998* | *RN-FII.chr5.904* | | | | | | | | *N-FvH4.5g11130.1* | | | | | |
| *TNL-FvH4.2g27370.1* | *TNL-FDA.chr2.20071* | | | | *CNL-FMA.chr3.2744* | *CNL-FNU.ctg81.894* | *RN-FII.chr5.904* | | | | | | | | *RN-FNG.chr5.1035* | | | | | |
| *TNL-FvH4.2g27370.1* | *TNL-FMA.chr2.2281* | | | | *CNL-FMA.chr3.2744* | *CNL-FPE.chr7.1015* | *RN-FII.chr6.3558* | | | | | | | | *RN-FvH4.6g42370.1* | | | | | |
| *TNL-FvH4.2g27370.1* | *TNL-FNG.chr2.2392* | | | | *CNL-FMA.chr3.2744* | *CNL-FvH4.7g11380.1* | *RN-FII.chr6.3558* | | | | | | | | *RNL-FNG.chr6.3533* | | | | | |
| *TNL-FvH4.2g27370.1* | *TNL-FNU.ctg114.235* | | | | *CNL-FMA.chr3.2975* | *CN-FvH4.3g21750.1* | *RN-FNG.chr1.1331* | | | | | | | | *RNL-FPE.chr6.6304* | | | | | |
| *TNL-FvH4.2g38620.1* | *TNL-FNU.ctg134.31* | | | | *CNL-FMA.chr3.2975* | *CNL-FNG.chr3.2531* | *RN-FNG.chr1.1331* | | | | | | | | *RNL-FvH4.1g15230.1* | | | | | |
| *TNL-FvH4.3g05740.1* | *TNL-FMA.chr5.2425* | | | | *CNL-FMA.chr3.2975* | *CNL-FNU.ctg21.293* | *RN-FNG.chr5.1035* | | | | | | | | *N-FvH4.5g11130.1* | | | | | |
| *TNL-FvH4.3g15720.1* | *TNL-FMA.chr2.2281* | | | | *CNL-FMA.chr3.2975* | *CNL-FPE.chr1.1624* | *RN-FNU.ctg109.400* | | | | | | | | *RNL-FvH4.1g15500.1* | | | | | |
| *TNL-FvH4.3g15720.1* | *TNL-FNU.ctg32.58* | | | | *CNL-FMA.chr3.75* | *CNL-FNG.chr3.84* | *RN-FNU.ctg172.29* | | | | | | | | *N-FDA.chr1.22180* | | | | | |
| Orthologous gene pairsof TNL | | | | | Orthologous gene pairs of non-TNL | | | | | | | | | | | | | | | |
| ID of sequence 1 | ID of sequence 2 | | | | ID of sequence 1 | ID of sequence 2 | | ID of sequence 1 | | | | | | ID of sequence 2 | | | | | | |
| *TNL-FvH4.3g33030.1* | *TN-FNU.ctg10.27* | | | | *CNL-FMA.chr3.75* | *CNL-FNU.ctg47.220* | *RN-FNU.ctg172.29* | | | | | | | | *RNL-FII.chr1.1265* | | | | | |
| *TNL-FvH4.3g33030.1* | *TNL-FDA.chr7.00159* | | | | *CNL-FMA.chr3.75* | *CNL-FvH4.3g00820.1* | *RN-FNU.ctg172.29* | | | | | | | | *RNL-FMA.chr1.1806* | | | | | |
| *TNL-FvH4.3g33030.1* | *TNL-FII.chr2.172* | | | | *CNL-FMA.chr4.2118* | *CNL-FNU.ctg82.313* | *RN-FNU.ctg172.34* | | | | | | | | *RN-FNG.chr1.1331* | | | | | |
| *TNL-FvH4.3g33030.1* | *TNL-FPE.chr0.1078* | | | | *CNL-FMA.chr4.2118* | *CNL-FPE.chr3.1482* | *RN-FNU.ctg172.34* | | | | | | | | *RNL-FDA.chr1.22183* | | | | | |
| *TNL-FvH4.3g36130.1* | *TNL-FDA.chr6.02361* | | | | *CNL-FMA.chr4.2118* | *CNL-FvH4.4g25750.1* | *RN-FNU.ctg172.34* | | | | | | | | *RNL-FII.chr1.1269* | | | | | |
| *TNL-FvH4.3g36130.1* | *TNL-FII.chr6.322* | | | | *CNL-FMA.chr4.2163* | *CNL-FNG.chr4.2225* | *RN-FNU.ctg172.34* | | | | | | | | *RNL-FPE.chr6.6304* | | | | | |
| *TNL-FvH4.3g38670.1* | *TNL-FII.chr3.3626* | | | | *CNL-FMA.chr4.2163* | *CNL-FvH4.4g26300.1* | *RN-FNU.ctg172.34* | | | | | | | | *RNL-FvH4.1g15170.1* | | | | | |
| *TNL-FvH4.3g38670.1* | *TNL-FNG.chr7.2518* | | | | *CNL-FMA.chr4.2163* | *NL-FNU.ctg82.264* | *RN-FNU.ctg172.42* | | | | | | | | *CNL-FDA.chr1.22189* | | | | | |
| *TNL-FvH4.3g38670.1* | *TNL-FNU.ctg132.96* | | | | *CNL-FMA.chr4.679* | *CNL-FNG.chr0.411* | *RN-FNU.ctg172.42* | | | | | | | | *NL-FII.chr1.1268* | | | | | |
| *TNL-FvH4.3g42940.1* | *TNL-FDA.chr3.20747* | | | | *CNL-FMA.chr4.679* | *CNL-FPE.chr3.535* | *RN-FNU.ctg172.42* | | | | | | | | *RNL-FMA.chr1.1806* | | | | | |
| *TNL-FvH4.3g43390.1* | *TNL-FDA.chr3.05894* | | | | *CNL-FMA.chr4.679* | *CNL-FvH4.4g06020.1* | *RN-FNU.ctg172.42* | | | | | | | | *RNL-FvH4.1g15220.1* | | | | | |
| *TNL-FvH4.3g43610.1* | *TNL-FDA.chr3.05894* | | | | *CNL-FMA.chr5.1324* | *CNL-FII.chr5.3003* | *RN-FNU.ctg172.45* | | | | | | | | *RN-FNG.chr1.1331* | | | | | |
| *TNL-FvH4.3g43630.1* | *TNL-FNU.ctg3.77* | | | | *CNL-FMA.chr5.1324* | *CNL-FNU.ctg147.19* | *RN-FNU.ctg172.45* | | | | | | | | *RNL-FDA.chr1.22190* | | | | | |
| *TNL-FvH4.3g43660.1* | *TNL-FDA.chr3.20670* | | | | *CNL-FMA.chr5.1324* | *CNL-FPE.chr1.2047* | *RN-FNU.ctg172.45* | | | | | | | | *RNL-FPE.chr6.6304* | | | | | |
| *TNL-FvH4.3g43660.1* | *TNL-FNU.ctg3.77* | | | | *CNL-FMA.chr5.1324* | *CNL-FvH4.5g16110.1* | *RN-FNU.ctg38.153* | | | | | | | | *RN-FDA.chr6.13755* | | | | | |
| *TNL-FvH4.3g43850.1* | *TNL-FNU.ctg3.77* | | | | *CNL-FMA.chr5.1739* | *CNL-FNG.chr5.1760* | *RN-FNU.ctg38.153* | | | | | | | | *RN-FII.chr3.1087* | | | | | |
| *TNL-FvH4.3g43860.1* | *TNL-FDA.chr3.05838* | | | | *CNL-FMA.chr5.1739* | *CNL-FvH4.5g19000.1* | *RN-FNU.ctg38.153* | | | | | | | | *RNL-FNG.chr6.4113* | | | | | |
| *TNL-FvH4.3g43880.1* | *TNL-FDA.chr3.05838* | | | | *CNL-FMA.chr5.2388* | *CN-FNG.chr5.2692* | *RN-FNU.ctg38.153* | | | | | | | | *RNL-FvH4.6g50310.1* | | | | | |
| *TNL-FvH4.3g43880.1* | *TNL-FNU.ctg3.77* | | | | *CNL-FMA.chr5.2388* | *CNL-FvH4.7g31140.1* | *RN-FNU.ctg52.52* | | | | | | | | *RNL-FNG.chr6.4113* | | | | | |
| *TNL-FvH4.3g43890.1* | *TNL-FDA.chr7.00274* | | | | *CNL-FMA.chr6.1184* | *CNL-FNU.ctg78.2* | *RN-FNU.ctg55.225* | | | | | | | | *RN-FII.chr6.3558* | | | | | |
| *TNL-FvH4.3g43940.1* | *TNL-FMA.chr3.3484* | | | | *CNL-FMA.chr6.1184* | *CNL-FvH4.6g12200.1* | *RN-FNU.ctg55.225* | | *RNL-FDA.chr4.04994* | | | | | | | | | | | |
| *TNL-FvH4.3g43950.1* | *TNL-FDA.chr7.00274* | | | | *CNL-FMA.chr6.1508* | *CNL-FDA.chr6.26337* | *RN-FNU.ctg55.225* | | *RNL-FNG.chr6.3533* | | | | | | | | | | | |
| *TNL-FvH4.3g43960.1* | *TNL-FDA.chr7.00274* | | | | *CNL-FMA.chr6.1508* | *CNL-FNU.ctg78.2* | *RN-FNU.ctg55.225* | | *RNL-FvH4.6g42380.1* | | | | | | | | | | | |
| *TNL-FvH4.3g44000.1* | *TNL-FNU.ctg3.75* | | | | *CNL-FMA.chr6.1508* | *CNL-FvH4.6g15090.1* | *RN-FNU.ctg81.619* | | *RN-FII.chr6.3558* | | | | | | | | | | | |
| *TNL-FvH4.3g44030.1* | *TNL-FNG.chr7.2518* | | | | *CNL-FMA.chr6.1658* | *CNL-FNU.ctg78.2* | *RN-FNU.ctg81.619* | | *RNL-FDA.chr4.04994* | | | | | | | | | | | |
| *TNL-FvH4.3g44030.1* | *TNL-FNU.ctg3.73* | | | | *CNL-FMA.chr6.1658* | *CNL-FvH4.6g13450.1* | *RN-FNU.ctg81.619* | | *RNL-FNG.chr6.3533* | | | | | | | | | | | |
| *TNL-FvH4.3g44050.1* | *TNL-FII.chr3.3603* | | | | *CNL-FMA.chr6.2166* | *CNL-FNG.chr6.2058* | *RN-FNU.ctg81.619* | | | | | | | | *RNL-FvH4.4g31860.1* | | | | | |
| *TNL-FvH4.3g44050.1* | *TNL-FNU.ctg3.77* | | | | *CNL-FMA.chr6.2166* | *CNL-FNU.ctg64.14.1* | *RN-FPE.chr6.6309* | | | | | | | | *RN-FNU.ctg172.29* | | | | | |
| *TNL-FvH4.3g44070.1* | *TNL-FNG.chr3.3322* | | | | *CNL-FMA.chr6.2166* | *CNL-FvH4.6g30120.1* | *RN-FPE.chr6.6309* | | | | | | | | *RN-FvH4.1g15140.1* | | | | | |
| *TNL-FvH4.3g44070.1* | *TNL-FNU.ctg3.75* | | | | *CNL-FMA.chr6.2166* | *NL-FII.chr6.2634* | *RN-FPE.chr6.6309* | | | | | | | | *RNL-FII.chr1.1269* | | | | | |
| *TNL-FvH4.3g44370.1* | *TNL-FNU.ctg3.22* | | | | *CNL-FMA.chr6.2723* | *CN-FNU.ctg65.11* | *RN-FPE.chr6.6309* | | | | | | | | *RNL-FMA.chr1.1806* | | | | | |
| *TNL-FvH4.3g44390.1* | *TNL-FNU.ctg3.21* | | | | *CNL-FMA.chr6.2723* | *CN-FPE.chr6.453* | *RN-FvH4.1g15140.1* | | | | | | | | *RN-FNU.ctg172.29* | | | | | |
| *TNL-FvH4.3g44570.1* | *TNL-FDA.chr3.20747* | | | | *CNL-FMA.chr6.2723* | *CNL-FNG.chr6.2058* | *RN-FvH4.1g15140.1* | | | | | | | | *RNL-FII.chr1.1269* | | | | | |
| *TNL-FvH4.3g44580.1* | *TNL-FDA.chr3.20725* | | | | *CNL-FMA.chr6.2723* | *CNL-FvH4.6g30120.1* | *RN-FvH4.1g15140.1* | | | | | | | | *RNL-FMA.chr1.1806* | | | | | |
| *TNL-FvH4.3g44580.1* | *TNL-FNU.ctg3.22* | | | | *CNL-FMA.chr6.3395* | *CN-FNG.chr5.2228* | *RN-FvH4.1g15200.1* | | | | | | | | *N-FNU.ctg172.28* | | | | | |
| *TNL-FvH4.3g44610.1* | *TNL-FDA.chr3.20747* | | | | *CNL-FMA.chr6.3395* | *CNL-FPE.chr2.397* | *RN-FvH4.1g15200.1* | | | | | | | | *RN-FDA.chr1.22186* | | | | | |
| *TNL-FvH4.3g44670.1* | *TNL-FMA.chr3.3484* | | | | *CNL-FMA.chr6.3395* | *CNL-FvH4.6g34770.1* | *RN-FvH4.1g15200.1* | | | | | | | | *RNL-FII.chr1.1266* | | | | | |
| *TNL-FvH4.3g44680.1* | *TNL-FDA.chr3.20747* | | | | *CNL-FMA.chr6.3395* | *N-FNU.ctg57.541* | *RN-FvH4.1g15200.1* | | | | | | | | *RNL-FMA.chr1.1807* | | | | | |
| *TNL-FvH4.3g45170.1* | *TNL-FDA.chr3.20747* | | | | *CNL-FMA.chr7.880* | *CNL-FvH4.7g11380.1* | *RN-FvH4.1g15240.1* | | | | | | | | *RN-FNG.chr1.1331* | | | | | |
| *TNL-FvH4.3g45170.1* | *TNL-FMA.chr3.3484* | | | | *CNL-FMA.chr7.880* | *NL-FNU.ctg132.328* | *RN-FvH4.1g15240.1* | | | | | | | | *RN-FNU.ctg172.45* | | | | | |
| *TNL-FvH4.3g45380.1* | *TNL-FDA.chr5.03488* | | | | *CNL-FNG.chr0.411* | *CNL-FII.chr3.408* | *RN-FvH4.1g15240.1* | | | | | | | | *RNL-FDA.chr1.22190* | | | | | |
| *TNL-FvH4.3g45380.1* | *TNL-FII.chr5.2969* | | | | *CNL-FNG.chr0.411* | *CNL-FPE.chr3.535* | *RN-FvH4.1g15240.1* | | | | | | | | *RNL-FPE.chr6.6304* | | | | | |
| Orthologous gene pairsof TNL | | | | | Orthologous gene pairs of non-TNL | | | | | | | | | | | | | | | |
| ID of sequence 1 | ID of sequence 2 | | | | ID of sequence 1 | ID of sequence 2 | | ID of sequence 1 | | | | | | ID of sequence 2 | | | | | | |
| *TNL-FvH4.3g45680.1* | *TNL-FDA.chr3.20747* | | | | *CNL-FNG.chr0.411* | *CNL-FvH4.7g31140.1* | | *RN-FvH4.6g42370.1* | | | | | | | *RNL-FDA.chr4.04994* | | | | | |
| *TNL-FvH4.3g45690.1* | *TNL-FDA.chr3.20747* | | | | *CNL-FNG.chr1.177* | *CNL-FII.chr5.3003* | | *RN-FvH4.6g42370.1* | | | | | | | *RNL-FMA.chr1.1807* | | | | | |
| *TNL-FvH4.3g45700.1* | *TNL-FDA.chr3.20747* | | | | *CNL-FNG.chr1.177* | *CNL-FMA.chr5.1324* | | *RN-FvH4.6g42370.1* | | | | | | | *RNL-FNG.chr6.3533* | | | | | |
| *TNL-FvH4.3g45730.1* | *TNL-FDA.chr3.20747* | | | | *CNL-FNG.chr1.177* | *CNL-FNU.ctg147.19* | | *RN-FvH4.6g50330.1* | | | | | | | *RN-FDA.chr6.13755* | | | | | |
| *TNL-FvH4.4g00790.1* | *TNL-FDA.chr3.09523* | | | | *CNL-FNG.chr1.177* | *CNL-FPE.chr1.2047* | | *RN-FvH4.6g50330.1* | | | | | | | *RN-FNU.ctg52.52* | | | | | |
| *TNL-FvH4.4g00790.1* | *TNL-FNU.ctg105.95* | | | | *CNL-FNG.chr1.177* | *CNL-FvH4.5g16110.1* | | *RN-FvH4.6g50330.1* | | | | | | | *RNL-FNG.chr6.4113* | | | | | |
| *TNL-FvH4.4g13420.1* | *TNL-FPE.chr6.1904* | | | | *CNL-FNG.chr1.545* | *CN-FvH4.3g16720.1* | | *RN-FVI.CHR1.1321* | | | | | | | *RN-FNG.chr1.1331* | | | | | |
| *TNL-FvH4.4g35420.1* | *TNL-FNG.chr4.3018* | | | | *CNL-FNG.chr1.545* | *CNL-FNU.ctg165.171* | | *RN-FVI.CHR1.1321* | | | | | | | *RN-FNU.ctg172.45* | | | | | |
| *TNL-FvH4.4g35420.1* | *TNL-FNU.ctg81.212* | | | | *CNL-FNG.chr1.545* | *CNL-FPE.chr5.124* | | *RN-FVI.CHR1.1321* | | *RN-FvH4.1g15240.1* | | | | | | | | | | |
| *TNL-FvH4.5g02970.1* | *TNL-FII.chr3.3603* | | | | *CNL-FNG.chr2.1430* | *CNL-FvH4.6g02820.1* | | *RN-FVI.CHR1.1321* | | *RNL-FDA.chr1.22190* | | | | | | | | | | |
| *TNL-FvH4.5g02970.1* | *TNL-FNG.chr5.112* | | | | *CNL-FNG.chr2.416* | *CN-FvH4.2g05600.1* | | *RN-FVI.CHR1.1321* | | | | | | | *RNL-FII.chr1.1270* | | | | | |
| *TNL-FvH4.5g02970.1* | *TNL-FNU.ctg3.75* | | | | *CNL-FNG.chr2.416* | *CNL-FII.chr3.1811* | | *RN-FVI.CHR1.1321* | | | | | | | *RNL-FPE.chr6.6304* | | | | | |
| *TNL-FvH4.5g07310.1* | *TNL-FMA.chr5.364* | | | | *CNL-FNG.chr2.416* | *CNL-FPE.chr7.1798* | | *RNL-FDA.chr1.22179* | | | | | | | *N-FNU.ctg172.28* | | | | | |
| *TNL-FvH4.5g16070.1* | *TNL-FNU.ctg147.20.1* | | | | *CNL-FNG.chr2.482* | *CN-FPE.chr7.728* | | *RNL-FDA.chr1.22179* | | | | | | | *RNL-FII.chr1.1266* | | | | | |
| *TNL-FvH4.5g27640.1* | *TNL-FII.chr3.3626* | | | | *CNL-FNG.chr2.482* | *CN-FvH4.2g06550.1* | | *RNL-FDA.chr1.22179* | | | | | | | *RNL-FMA.chr1.1807* | | | | | |
| *TNL-FvH4.5g27640.1* | *TNL-FNG.chr7.2518* | | | | *CNL-FNG.chr2.482* | *CNL-FNU.ctg104.299* | | *RNL-FDA.chr1.22179* | | | | | | | *RNL-FvH4.1g15130.1* | | | | | |
| *TNL-FvH4.5g32050.1* | *TNL-FII.chr5.2851* | | | | *CNL-FNG.chr3.1370* | *CNL-FNU.ctg30.47* | | *RNL-FDA.chr1.22182* | | | | | | | *RNL-FII.chr1.1269* | | | | | |
| *TNL-FvH4.5g32050.1* | *TNL-FNU.ctg160.483* | | | | *CNL-FNG.chr3.1370* | *CNL-FPE.chr5.124* | | *RNL-FDA.chr1.22182* | | | | | | | *RNL-FMA.chr1.1806* | | | | | |
| *TNL-FvH4.5g32970.1* | *TNL-FNU.ctg160.481* | | | | *CNL-FNG.chr3.1371* | *CN-FvH4.3g16720.1* | | *RNL-FDA.chr1.22182* | | | | | | | *RNL-FPE.chr6.6304* | | | | | |
| *TNL-FvH4.5g34210.1* | *TNL-FNU.ctg160.481* | | | | *CNL-FNG.chr3.1371* | *CNL-FII.chr3.1385* | | *RNL-FDA.chr1.22182* | | | | | | | *RNL-FvH4.1g15160.1* | | | | | |
| *TNL-FvH4.5g38540.1* | *TNL-FDA.chr5.12803* | | | | *CNL-FNG.chr3.1371* | *CNL-FMA.chr3.1344* | | *RNL-FDA.chr1.22183* | | | | | | | *RNL-FII.chr1.1269* | | | | | |
| *TNL-FvH4.5g38540.1* | *TNL-FII.chr5.3330* | | | | *CNL-FNG.chr3.1371* | *CNL-FNU.ctg165.171* | | *RNL-FDA.chr1.22183* | | | | | | | *RNL-FMA.chr1.1806* | | | | | |
| *TNL-FvH4.5g38600.1* | *TNL-FDA.chr5.12803* | | | | *CNL-FNG.chr3.1371* | *CNL-FPE.chr5.124* | | *RNL-FDA.chr1.22183* | | | | | | | *RNL-FPE.chr6.6304* | | | | | |
| *TNL-FvH4.5g38600.1* | *TNL-FII.chr5.3330* | | | | *CNL-FNG.chr3.1371* | *NL-FDA.chr3.21153* | | *RNL-FDA.chr1.22183* | | | | | | | *RNL-FvH4.1g15170.1* | | | | | |
| *TNL-FvH4.5g38680.1* | *TNL-FNG.chr2.105* | | | | *CNL-FNG.chr3.2531* | *CNL-FNU.ctg21.293* | | *RNL-FDA.chr1.22184* | | | | | | | *RNL-FMA.chr1.1806* | | | | | |
| *TNL-FvH4.5g38870.1* | *TNL-FII.chr5.3371* | | | | *CNL-FNG.chr3.2531* | *CNL-FPE.chr1.1624* | | *RNL-FDA.chr1.22184* | | | | | | | *RNL-FvH4.1g15160.1* | | | | | |
| *TNL-FvH4.5g38870.1* | *TNL-FMA.chr1.1780* | | | | *CNL-FNG.chr3.84* | *CNL-FNU.ctg47.220* | | *RNL-FDA.chr1.22187* | | | | *RNL-FII.chr1.1267* | | | | | | | | |
| *TNL-FvH4.6g01690.1* | *TNL-FDA.chr7.00582* | | | | *CNL-FNG.chr3.84* | *CNL-FvH4.3g00820.1* | | *RNL-FDA.chr1.22187* | | | | *RNL-FMA.chr1.1807* | | | | | | | | |
| *TNL-FvH4.6g01690.1* | *TNL-FNU.ctg134.16* | | | | *CNL-FNG.chr4.1917* | *CN-FPE.chr1.928* | | *RNL-FDA.chr1.22187* | | | | *RNL-FvH4.1g15210.1* | | | | | | | | |
| *TNL-FvH4.6g01690.1* | *TNL-FPE.chr7.2368* | | | | *CNL-FNG.chr4.1917* | *CNL-FNU.ctg82.648* | | *RNL-FDA.chr1.22190* | | | | *RN-FNG.chr1.1331* | | | | | | | | |
| *TNL-FvH4.6g03250.1* | *TNL-FII.chr7.1130* | | | | *CNL-FNG.chr4.1917* | *CNL-FvH4.4g22940.1* | | *RNL-FDA.chr1.22190* | | | | *RNL-FII.chr1.1270* | | | | | | | | |
| *TNL-FvH4.6g03250.1* | *TNL-FMA.chr5.2425* | | | | *CNL-FNG.chr4.2225* | *CNL-FvH4.4g26300.1* | | *RNL-FDA.chr1.22190* | | | | *RNL-FPE.chr6.6304* | | | | | | | | |
| *TNL-FvH4.6g04250.1* | *TNL-FDA.chr6.02345* | | | | *CNL-FNG.chr4.2225* | *NL-FNU.ctg82.264* | | *RNL-FDA.chr1.22190* | | | | *RNL-FvH4.1g15230.1* | | | | | | | | |
| *TNL-FvH4.6g04250.1* | *TNL-FII.chr6.322* | | | | *CNL-FNG.chr5.1760* | *CNL-FvH4.5g18950.1* | | *RNL-FDA.chr1.22196* | | | | *CNL-FNU.ctg172.55* | | | | | | | | |
| *TNL-FvH4.6g04250.1* | *TNL-FNU.ctg8.247* | | | | *CNL-FNG.chr5.1760* | *NL-FNU.ctg155.6* | | *RNL-FDA.chr1.22196* | | | | *RNL-FMA.chr1.1806* | | | | | | | | |
| *TNL-FvH4.6g04730.1* | *TNL-FDA.chr6.02345* | | | | *CNL-FNG.chr5.2089* | *CNL-FDA.chr5.25050* | | *RNL-FDA.chr1.22196* | | | | *RNL-FNG.chr1.1333* | | | | | | | | |
| *TNL-FvH4.6g04730.1* | *TNL-FII.chr6.322* | | | | *CNL-FNG.chr5.2089* | *CNL-FII.chr5.3063* | | *RNL-FDA.chr1.22196* | | | | *RNL-FvH4.1g15330.1* | | | | | | | | |
| *TNL-FvH4.6g04730.1* | *TNL-FNU.ctg8.247* | | | | *CNL-FNG.chr5.2089* | *CNL-FNU.ctg158.42* | | *RNL-FDA.chr1.22201* | | | | *RNL-FNG.chr1.1344* | | | | | | | | |
| *TNL-FvH4.6g05610.1* | *TNL-FNU.ctg8.247* | | | | *CNL-FNG.chr5.2089* | *CNL-FvH4.5g22710.1* | | *RNL-FDA.chr1.22201* | | | | *RNL-FNU.ctg172.65* | | | | | | | | |
| *TNL-FvH4.6g34410.1* | *TNL-FMA.chr5.2425* | | | | *CNL-FNG.chr5.2145* | *CNL-FDA.chr5.25050* | | *RNL-FDA.chr1.22201* | | | | *RNL-FvH4.1g15500.1* | | | | | | | | |
| *TNL-FvH4.6g34410.1* | *TNL-FNG.chr5.2639* | | | | *CNL-FNG.chr5.2145* | *CNL-FNU.ctg158.40* | | *RNL-FDA.chr2.11777* | | | | *RNL-FII.chr2.3467* | | | | | | | | |
| *TNL-FvH4.7g04050.1* | *TNL-FII.chr5.3371* | | | | *CNL-FNG.chr5.2145* | *CNL-FPE.chr4.1619* | | *RNL-FDA.chr2.11777* | | | | *RNL-FMA.chr2.3489* | | | | | | | | |
| Orthologous gene pairsof TNL | | | | | Orthologous gene pairs of non-TNL | | | | | | | | | | | | | | | |
| ID of sequence 1 | ID of sequence 2 | | | | ID of sequence 1 | ID of sequence 2 | | ID of sequence 1 | | | | | | ID of sequence 2 | | | | | | |
| *TNL-FvH4.7g04050.1* | *TNL-FMA.chr5.2877* | | | | *CNL-FNG.chr5.2145* | *CNL-FvH4.5g23400.1* | | *RNL-FDA.chr2.11777* | | | | *RNL-FNG.chr2.3537* | | | | | | | | |
| *TNL-FvH4.7g06680.1* | *TNL-FDA.chr3.05838* | | | | *CNL-FNG.chr5.2146* | *CNL-FNU.ctg158.42* | | *RNL-FDA.chr2.11777* | | | | *RNL-FNU.ctg122.114* | | | | | | | | |
| *TNL-FvH4.7g06680.1* | *TNL-FNU.ctg132.96* | | | | *CNL-FNG.chr5.2146* | *CNL-FvH4.5g23420.1* | | *RNL-FDA.chr2.11777* | | | | *RNL-FPE.chr0.2431* | | | | | | | | |
| *TNL-FvH4.7g09800.1* | *TNL-FII.chr7.936* | | | | *CNL-FNG.chr5.2147* | *CNL-FNU.ctg158.43* | | *RNL-FDA.chr2.11777* | | | | *RNL-FvH4.2g40720.1* | | | | | | | | |
| *TNL-FvH4.7g09800.1* | *TNL-FMA.chr2.1405* | | | | *CNL-FNG.chr5.2147* | *CNL-FvH4.5g23430.1* | | *RNL-FDA.chr4.04994* | | | | *RN-FII.chr6.3558* | | | | | | | | |
| *TNL-FvH4.7g09800.1* | *TNL-FNU.ctg135.24* | | | | *CNL-FNG.chr5.2149* | *CNL-FNU.ctg158.45* | | *RNL-FDA.chr4.04994* | | | | *RNL-FNG.chr6.3533* | | | | | | | | |
| *TNL-FvH4.7g10520.1* | *TNL-FMA.chr2.1405* | | | | *CNL-FNG.chr5.2149* | *CNL-FvH4.5g23450.1* | | *RNL-FDA.chr4.04994* | | | | *RNL-FvH4.6g42380.1* | | | | | | | | |
| *TNL-FvH4.7g10520.1* | *TNL-FNU.ctg134.120* | | | | *CNL-FNG.chr5.2150* | *CNL-FNU.ctg158.48* | | *RNL-FDA.chr6.12990* | | | | *RN-FII.chr6.3558* | | | | | | | | |
| *TNL-FvH4.7g10750.1* | *TNL-FMA.chr2.1405* | | | | *CNL-FNG.chr5.2150* | *CNL-FvH4.5g23470.1* | | *RNL-FDA.chr6.12990* | | | | *RNL-FNG.chr6.3533* | | | | | | | | |
| *TNL-FvH4.7g10750.1* | *TNL-FNG.chr7.552* | | | | *CNL-FNG.chr5.2227* | *CNL-FII.chr5.2008* | | *RNL-FDA.chr6.12990* | | | | | | | *RNL-FvH4.4g31860.1* | | | | | |
| *TNL-FvH4.7g11550.1* | *TNL-FDA.chr7.01621* | | | | *CNL-FNG.chr5.2227* | *CNL-FNU.ctg158.148* | | *RNL-FDA.chr6.13739* | | | | | | | *CNL-FII.chr1.1471* | | | | | |
| *TNL-FvH4.7g11550.1* | *TNL-FII.chr6.136* | | | | *CNL-FNG.chr5.2227* | *CNL-FvH4.5g23470.1* | | *RNL-FDA.chr6.13739* | | | | | | | *RNL-FMA.chr1.1806* | | | | | |
| *TNL-FvH4.7g11550.1* | *TNL-FNU.ctg134.16* | | | | *CNL-FNG.chr5.2999* | *CNL-FMA.chr4.2118* | | *RNL-FDA.chr6.13739* | | | | | | | *RNL-FNG.chr6.4114* | | | | | |
| *TNL-FvH4.7g11550.1* | *TNL-FPE.chr7.2368* | | | | *CNL-FNG.chr5.2999* | *CNL-FNU.ctg82.313* | | *RNL-FDA.chr6.13739* | | | | | | | *RNL-FvH4.6g50140.1* | | | | | |
| *TNL-FvH4.7g12160.1* | *TNL-FDA.chr7.00159* | | | | *CNL-FNG.chr5.2999* | *CNL-FPE.chr1.2047* | | *RNL-FII.chr1.1265* | | | | | | | *CNL-FNU.ctg172.27* | | | | | |
| *TNL-FvH4.7g13090.1* | *TNL-FNG.chr5.2639* | | | | *CNL-FNG.chr5.2999* | *CNL-FvH4.5g34680.1* | | *RNL-FII.chr1.1265* | | | | | | | *RNL-FMA.chr1.1808* | | | | | |
| *TNL-FvH4.7g13640.1* | *TNL-FDA.chr7.14713* | | | | *CNL-FNG.chr5.3271* | *CNL-FDA.chr5.03688* | | *RNL-FII.chr1.1265* | | | | | | | *RNL-FvH4.1g15120.1* | | | | | |
| *TNL-FvH4.7g13640.1* | *TNL-FNG.chr7.2518* | | | | *CNL-FNG.chr5.3271* | *CNL-FvH4.5g32430.1* | | *RNL-FII.chr1.1266* | | | | | | | *N-FNU.ctg172.28* | | | | | |
| *TNL-FvH4.7g13640.1* | *TNL-FNU.ctg132.96* | | | | *CNL-FNG.chr5.3271* | *NL-FNU.ctg161.49* | | *RNL-FII.chr1.1266* | | | | | | | *RNL-FMA.chr1.1807* | | | | | |
| *TNL-FvH4.7g13640.1* | *TNL-FPE.chr1.1274* | | | | *CNL-FNG.chr5.654* | *CNL-FDA.chr6.13552* | | *RNL-FII.chr1.1266* | | | | | | | *RNL-FvH4.1g15130.1* | | | | | |
| *TNL-FvH4.7g17700.1* | *TNL-FPE.chr7.2368* | | | | *CNL-FNG.chr5.654* | *CNL-FII.chr6.4055* | | *RNL-FII.chr1.1267* | | | | | | | *RNL-FMA.chr1.1807* | | | | | |
| *TNL-FvH4.7g21070.1* | *TNL-FDA.chr7.01326* | | | | *CNL-FNG.chr5.654* | *CNL-FvH4.6g48220.1* | | *RNL-FII.chr1.1267* | | | | | | | *RNL-FvH4.1g15210.1* | | | | | |
| *TNL-FvH4.7g21180.1* | *TNL-FDA.chr7.01326* | | | | *CNL-FNG.chr5.655* | *CN-FNU.ctg53.150* | | *RNL-FII.chr1.1269* | | | | | | | *RN-FNG.chr1.1331* | | | | | |
| *TNL-FvH4.7g28540.1* | *TNL-FDA.chr7.01621* | | | | *CNL-FNG.chr5.655* | *CNL-FvH4.6g48220.1* | | *RNL-FII.chr1.1269* | | | | | | | *RNL-FMA.chr1.1806* | | | | | |
| *TNL-FvH4.7g28540.1* | *TNL-FII.chr7.2338* | | | | *CNL-FNG.chr6.2058* | *CN-FNU.ctg65.11* | | *RNL-FII.chr1.1269* | | | | | | | *RNL-FPE.chr6.6304* | | | | | |
| *TNL-FvH4.7g31110.1* | *TNL-FDA.chr3.05838* | | | | *CNL-FNG.chr6.2058* | *CN-FPE.chr6.453* | | *RNL-FII.chr1.1269* | | | | | | | *RNL-FvH4.1g15230.1* | | | | | |
| *TNL-FvH4.7g31110.1* | *TNL-FNU.ctg132.96* | | | | *CNL-FNG.chr6.2058* | *CNL-FvH4.6g30120.1* | | *RNL-FII.chr1.1270* | | | | | | | *RN-FNG.chr1.1331* | | | | | |
| *TNL-FvH4.7g31110.1* | *TNL-FPE.chr1.1274* | | | | *CNL-FNG.chr6.2406* | *CN-FNU.ctg84.31* | | *RNL-FII.chr1.1270* | | | | | | | *RN-FNU.ctg172.45* | | | | | |
| *TNL-FvH4.7g32440.1* | *TNL-FDA.chr7.00189* | | | | *CNL-FNG.chr6.2406* | *CNL-FDA.chr6.26337* | | *RNL-FII.chr1.1270* | | | | | | | *RN-FvH4.1g15240.1* | | | | | |
| *TNL-FvH4.7g32470.1* | *TNL-FDA.chr7.00189* | | | | *CNL-FNG.chr6.2406* | *CNL-FMA.chr6.1508* | | *RNL-FII.chr1.1270* | | | | | | | *RNL-FMA.chr1.1807* | | | | | |
| *TNL-FvH4.7g32480.1* | *TNL-FDA.chr7.00189* | | | | *CNL-FNG.chr6.2406* | *CNL-FvH4.6g15090.1* | | *RNL-FII.chr1.1270* | | | | | | | *RNL-FPE.chr6.6304* | | | | | |
| *TNL-FvH4.7g32760.1* | *TNL-FDA.chr7.00189* | | | | *CNL-FNG.chr6.2420* | *CNL-FII.chr7.1027* | | *RNL-FII.chr2.1682* | | | | | | | *RN-FNU.ctg109.400* | | | | | |
| *TNL-FvH4.7g33140.1* | *TNL-FDA.chr7.00190* | | | | *CNL-FNG.chr6.2420* | *CNL-FMA.chr6.1184* | | *RNL-FII.chr2.1682* | | | | | | | *RNL-FDA.chr1.22182* | | | | | |
| *TNL-FVI.CHR1.1015* | *TNL-FDA.chr7.00159* | | | | *CNL-FNG.chr6.2420* | *CNL-FvH4.6g15250.1* | | *RNL-FII.chr2.1682* | | | | | | | *RNL-FMA.chr2.1492* | | | | | |
| *TNL-FVI.CHR1.1015* | *TNL-FNG.chr1.1006* | | | | *CNL-FNG.chr6.2431* | *CNL-FDA.chr6.26337* | | *RNL-FII.chr2.1682* | | | | | | | *RNL-FNG.chr2.1787* | | | | | |
| *TNL-FVI.CHR1.1015* | *TNL-FvH4.1g11570.1* | | | | *CNL-FNG.chr6.2431* | *CNL-FMA.chr6.1184* | | *RNL-FII.chr2.1682* | | | | | | | *RNL-FPE.chr5.2061* | | | | | |
| *TNL-FVI.CHR1.1444* | *TNL-FDA.chr1.17981* | | | | *CNL-FNG.chr6.2431* | *CNL-FvH4.6g15340.1* | | *RNL-FII.chr2.1682* | | | | | | | *RNL-FvH4.1g15220.1* | | | | | |
| *TNL-FVI.CHR1.1444* | *TNL-FII.chr1.1394* | | | | *CNL-FNG.chr7.204* | *CNL-FNU.ctg145.8* | | *RNL-FII.chr2.3467* | | | | | | | *RNL-FMA.chr2.3489* | | | | | |
| *TNL-FVI.CHR1.1444* | *TNL-FNU.ctg172.196* | | | | *CNL-FNG.chr7.204* | *CNL-FPE.chr6.1639* | | *RNL-FII.chr2.3467* | | | | | | | *RNL-FNG.chr2.3537* | | | | | |
| *TNL-FVI.CHR1.1444* | *TNL-FvH4.1g16650.1* | | | | *CNL-FNG.chr7.204* | *CNL-FvH4.7g02890.1* | | *RNL-FII.chr2.3467* | | | | | | | *RNL-FNU.ctg122.114* | | | | | |
| *TNL-FVI.CHR1.592* | *TNL-FDA.chr1.07161* | | | | *CNL-FNG.chr7.951* | *CN-FPE.chr6.3765* | | *RNL-FII.chr2.3467* | | | | | | | *RNL-FPE.chr0.2431* | | | | | |
| *TNL-FVI.CHR1.592* | *TNL-FMA.chr0.553* | | | | *CNL-FNG.chr7.951* | *CNL-FMA.chr6.1184* | | *RNL-FII.chr2.3467* | | | | | | | *RNL-FvH4.2g40720.1* | | | | | |
| Orthologous gene pairsof TNL | | | | | Orthologous gene pairs of non-TNL | | | | | | | | | | | | | | | |
| ID of sequence 1 | ID of sequence 2 | | | | ID of sequence 1 | ID of sequence 2 | | ID of sequence 1 | | | | | | ID of sequence 2 | | | | | | |
| *TNL-FVI.CHR1.592* | *TNL-FNU.ctg173.410* | | | | *CNL-FNG.chr7.951* | *CNL-FNU.ctg132.293* | | *RNL-FMA.chr1.1806* | | | | | | | *RNL-FPE.chr6.6304* | | | | | |
| *TNL-FVI.CHR1.592* | *TNL-FvH4.1g07020.1* | | | | *CNL-FNG.chr7.951* | *CNL-FvH4.6g12200.1* | | *RNL-FMA.chr1.1806* | | | | | | | *RNL-FvH4.1g15160.1* | | | | | |
| *TNL-FVI.CHR2.1052* | *TNL-FII.chr6.2998* | | | | *CNL-FNU.ctg104.183* | *CNL-FII.chr3.1811* | | *RNL-FMA.chr1.1807* | | | | | | | *N-FNU.ctg172.28* | | | | | |
| *TNL-FVI.CHR2.1052* | *TNL-FMA.chr5.2425* | | | | *CNL-FNU.ctg104.183* | *CNL-FNG.chr2.416* | | *RNL-FMA.chr1.1807* | | | | | | | *RNL-FvH4.1g15130.1* | | | | | |
| *TNL-FVI.CHR2.1052* | *TNL-FNG.chr5.2639* | | | | *CNL-FNU.ctg104.183* | *CNL-FPE.chr7.1798* | | *RNL-FMA.chr1.1808* | | | | | | | *CNL-FNU.ctg172.27* | | | | | |
| *TNL-FVI.CHR2.1052* | *TNL-FvH4.6g34410.1* | | | | *CNL-FNU.ctg132.293* | *CNL-FMA.chr6.1508* | | *RNL-FMA.chr1.1808* | | | | | | | *RNL-FvH4.1g15120.1* | | | | | |
| *TNL-FVI.CHR2.166* | *TNL-FDA.chr2.12488* | | | | *CNL-FNU.ctg134.171* | *CNL-FDA.chr1.06545* | | *RNL-FMA.chr1.1810* | | | | | | | *N-FNU.ctg172.22* | | | | | |
| *TNL-FVI.CHR2.166* | *TNL-FII.chr2.172* | | | | *CNL-FNU.ctg134.171* | *CNL-FII.chr7.998* | | *RNL-FMA.chr1.1810* | | | | | | | *RNL-FDA.chr1.22182* | | | | | |
| *TNL-FVI.CHR2.166* | *TNL-FNG.chr2.105* | | | | *CNL-FNU.ctg134.171* | *CNL-FMA.chr7.880* | | *RNL-FMA.chr1.1810* | | | | | | | *RNL-FII.chr1.1265* | | | | | |
| *TNL-FVI.CHR2.166* | *TNL-FPE.chr0.1078* | | | | *CNL-FNU.ctg146.100* | *CNL-FNG.chr7.204* | | *RNL-FMA.chr1.1810* | | | | | | | *RNL-FvH4.1g15090.1* | | | | | |
| *TNL-FVI.CHR2.166* | *TNL-FvH4.2g02050.1* | | | | *CNL-FNU.ctg147.19* | *CNL-FII.chr5.3003* | | *RNL-FMA.chr2.1492* | | | | | | | *RN-FNU.ctg109.400* | | | | | |
| *TNL-FVI.CHR2.2260* | *TNL-FII.chr7.936* | | | | *CNL-FNU.ctg147.19* | *CNL-FPE.chr3.1482* | | *RNL-FMA.chr2.1492* | | | | | | | *RNL-FDA.chr1.22182* | | | | | |
| *TNL-FVI.CHR2.2260* | *TNL-FMA.chr2.1405* | | | | *CNL-FNU.ctg150.57* | *CNL-FII.chr3.408* | | *RNL-FMA.chr2.1492* | | | | | | | *RNL-FNG.chr2.1787* | | | | | |
| *TNL-FVI.CHR2.2260* | *TNL-FNU.ctg109.139* | | | | *CNL-FNU.ctg150.57* | *CNL-FNG.chr0.411* | | *RNL-FMA.chr2.1492* | | | | | | | *RNL-FPE.chr5.2061* | | | | | |
| *TNL-FVI.CHR2.2260* | *TNL-FPE.chr2.959* | | | | *CNL-FNU.ctg150.57* | *CNL-FvH4.5g18950.1* | | *RNL-FMA.chr2.3489* | | | | | | | *RNL-FNG.chr2.3537* | | | | | |
| *TNL-FVI.CHR2.2260* | *TNL-FvH4.2g17330.1* | | | | *CNL-FNU.ctg154.468* | *CNL-FDA.chr6.02010* | | *RNL-FMA.chr2.3489* | | | | | | | *RNL-FNU.ctg122.114* | | | | | |
| *TNL-FVI.CHR2.2512* | *TNL-FII.chr2.1754* | | | | *CNL-FNU.ctg155.369* | *CNL-FDA.chr5.25050* | | *RNL-FMA.chr2.3489* | | | | | | | *RNL-FPE.chr0.2431* | | | | | |
| *TNL-FVI.CHR2.2512* | *TNL-FMA.chr2.1405* | | | | *CNL-FNU.ctg155.369* | *CNL-FII.chr5.3063* | | *RNL-FMA.chr2.3489* | | | | | | | *RNL-FvH4.2g40720.1* | | | | | |
| *TNL-FVI.CHR2.2512* | *TNL-FNG.chr2.1729* | | | | *CNL-FNU.ctg155.369* | *CNL-FNG.chr5.2089* | | *RNL-FNG.chr1.1333* | | | | | | | *CNL-FNU.ctg172.55* | | | | | |
| *TNL-FVI.CHR2.2512* | *TNL-FvH4.2g20310.1* | | | | *CNL-FNU.ctg155.369* | *CNL-FvH4.5g22710.1* | | *RNL-FNG.chr1.1333* | | | | | | | *RNL-FMA.chr1.1806* | | | | | |
| *TNL-FVI.CHR2.3162* | *TNL-FDA.chr2.20071* | | | | *CNL-FNU.ctg156.8* | *CNL-FDA.chr5.25050* | | *RNL-FNG.chr1.1333* | | | | | | | *RNL-FvH4.1g15330.1* | | | | | |
| *TNL-FVI.CHR2.3162* | *TNL-FMA.chr2.2281* | | | | *CNL-FNU.ctg156.8* | *CNL-FII.chr5.3063* | | *RNL-FNG.chr1.1336* | | | | | | | *RNL-FDA.chr1.22182* | | | | | |
| *TNL-FVI.CHR2.3162* | *TNL-FNG.chr2.2392* | | | | *CNL-FNU.ctg156.8* | *CNL-FNG.chr5.2089* | | *RNL-FNG.chr1.1336* | | | | | | | *RNL-FMA.chr1.1806* | | | | | |
| *TNL-FVI.CHR2.3162* | *TNL-FvH4.2g27720.1* | | | | *CNL-FNU.ctg156.8* | *CNL-FvH4.5g22710.1* | | *RNL-FNG.chr1.1336* | | | | | | | *RNL-FNU.ctg172.65* | | | | | |
| *TNL-FVI.CHR2.4091* | *TNL-FII.chr2.55* | | | | *CNL-FNU.ctg158.148* | *CNL-FII.chr5.2008* | | *RNL-FNG.chr1.1336* | | | | | | | *RNL-FvH4.1g15380.1* | | | | | |
| *TNL-FVI.CHR2.4091* | *TNL-FNG.chr3.3728* | | | | *CNL-FNU.ctg158.148* | *CNL-FvH4.5g23470.1* | | *RNL-FNG.chr1.1337* | | | | | | | *RNL-FDA.chr1.22182* | | | | | |
| *TNL-FVI.CHR2.4091* | *TNL-FNU.ctg134.31* | | | | *CNL-FNU.ctg158.149* | *CNL-FPE.chr7.1124* | | *RNL-FNG.chr1.1337* | | | | | | | *RNL-FII.chr1.1265* | | | | | |
| *TNL-FVI.CHR2.4091* | *TNL-FvH4.2g38620.1* | | | | *CNL-FNU.ctg158.40* | *CNL-FDA.chr5.25050* | | *RNL-FNG.chr1.1337* | | | | | | | *RNL-FMA.chr1.1806* | | | | | |
| *TNL-FVI.CHR3.118* | *TN-FDA.chr3.15822* | | | | *CNL-FNU.ctg158.46* | *CNL-FDA.chr5.25047* | | *RNL-FNG.chr1.1337* | | | | | | | *RNL-FPE.chr6.3526* | | | | | |
| *TNL-FVI.CHR3.118* | *TNL-FMA.chr2.2218* | | | | *CNL-FNU.ctg158.46* | *CNL-FNG.chr5.2149* | | *RNL-FNG.chr1.1337* | | | | | | | *RNL-FvH4.1g15390.1* | | | | | |
| *TNL-FVI.CHR3.118* | *TNL-FNU.ctg32.58* | | | | *CNL-FNU.ctg158.46* | *CNL-FvH4.5g23450.1* | | *RNL-FNG.chr1.1344* | | | | | | | *RNL-FII.chr1.1269* | | | | | |
| *TNL-FVI.CHR3.118* | *TNL-FvH4.3g15720.1* | | | | *CNL-FNU.ctg169.439* | *CNL-FII.chr1.1071* | | *RNL-FNG.chr1.1344* | | | | | | | *RNL-FNU.ctg172.65* | | | | | |
| *TNL-FVI.CHR3.2623* | *TNL-FDA.chr3.20725* | | | | *CNL-FNU.ctg169.439* | *CNL-FMA.chr1.1369* | | *RNL-FNG.chr1.1344* | | | | | | | *RNL-FvH4.1g15500.1* | | | | | |
| *TNL-FVI.CHR3.2623* | *TNL-FNU.ctg3.22* | | | | *CNL-FNU.ctg169.439* | *CNL-FPE.chr6.1273* | | *RNL-FNG.chr2.1787* | | | | | | | *RN-FNU.ctg109.400* | | | | | |
| *TNL-FVI.CHR3.2623* | *TNL-FvH4.3g44390.1* | | | | *CNL-FNU.ctg169.439* | *N-FvH4.1g12710.1* | | *RNL-FNG.chr2.1787* | | | | | | | *RNL-FDA.chr1.22182* | | | | | |
| *TNL-FVI.CHR4.3081* | *TNL-FII.chr4.2876* | | | | *CNL-FNU.ctg169.439* | *NL-FNG.chr1.1121* | | *RNL-FNG.chr2.1787* | | | | | | | *RNL-FPE.chr5.2061* | | | | | |
| *TNL-FVI.CHR4.3081* | *TNL-FMA.chr4.2946* | | | | *CNL-FNU.ctg172.55* | *RNL-FMA.chr1.1806* | | *RNL-FNG.chr2.1787* | | | | | | | *RNL-FvH4.1g15220.1* | | | | | |
| *TNL-FVI.CHR4.3081* | *TNL-FNG.chr4.3018* | | | | *CNL-FNU.ctg30.47* | *CNL-FMA.chr3.1344* | | *RNL-FNG.chr2.3537* | | | | | | | *RNL-FNU.ctg122.114* | | | | | |
| *TNL-FVI.CHR4.3081* | *TNL-FNU.ctg81.211* | | | | *CNL-FNU.ctg30.47* | *CNL-FPE.chr5.124* | | *RNL-FNG.chr2.3537* | | | | | | | *RNL-FPE.chr0.2431* | | | | | |
| *TNL-FVI.CHR4.3082* | *TNL-FII.chr4.2875* | | | | *CNL-FNU.ctg48.6* | *CNL-FII.chr3.1384* | | *RNL-FNG.chr2.3537* | | | | | | | *RNL-FvH4.2g40720.1* | | | | | |
| *TNL-FVI.CHR4.3082* | *TNL-FMA.chr4.2946* | | | | *CNL-FNU.ctg48.6* | *CNL-FMA.chr3.1344* | | *RNL-FNG.chr6.3533* | | | | | | | *RNL-FvH4.6g42380.1* | | | | | |
| *TNL-FVI.CHR4.3082* | *TNL-FNG.chr4.3018* | | | | *CNL-FNU.ctg48.6* | *CNL-FNG.chr3.1370* | | *RNL-FNG.chr6.4113* | | | | | | | *RNL-FDA.chr6.13739* | | | | | |
| Orthologous gene pairsof TNL | | | | | Orthologous gene pairs of non-TNL | | | | | | | | | | | | | | | |
| ID of sequence 1 | ID of sequence 2 | | | | ID of sequence 1 | ID of sequence 2 | | ID of sequence 1 | | | | | | ID of sequence 2 | | | | | | |
| *TNL-FVI.CHR4.3082* | *TNL-FNU.ctg81.212* | | | | *CNL-FNU.ctg48.6* | *CNL-FPE.chr5.124* | | *RNL-FNG.chr6.4113* | | | | | | | *RNL-FvH4.6g50100.1* | | | | | |
| *TNL-FVI.CHR4.3082* | *TNL-FvH4.4g35420.1* | | | | *CNL-FNU.ctg53.160* | *CNL-FDA.chr6.13561* | | *RNL-FNG.chr6.4114* | | | | | | | *CNL-FII.chr1.1471* | | | | | |
| *TNL-FVI.CHR4.3083* | *TNL-FII.chr4.2876* | | | | *CNL-FNU.ctg53.160* | *CNL-FvH4.6g48310.1* | | *RNL-FNG.chr6.4114* | | | | | | | *RNL-FvH4.6g50140.1* | | | | | |
| *TNL-FVI.CHR4.3083* | *TNL-FMA.chr4.2945* | | | | *CNL-FNU.ctg53.185* | *CNL-FDA.chr6.13561* | | *RNL-FNU.ctg172.65* | | | | | | | *RNL-FII.chr1.1269* | | | | | |
| *TNL-FVI.CHR4.3083* | *TNL-FNG.chr4.3018* | | | | *CNL-FNU.ctg53.185* | *CNL-FvH4.6g47050.1* | | *RNL-FPE.chr0.2431* | | | | | | | *RNL-FNU.ctg122.114* | | | | | |
| *TNL-FVI.CHR4.3083* | *TNL-FNU.ctg81.211* | | | | *CNL-FNU.ctg53.289* | *CNL-FDA.chr6.13561* | | *RNL-FPE.chr0.2431* | | | | | | | *RNL-FvH4.2g40720.1* | | | | | |
| *TNL-FVI.CHR5.2738* | *TNL-FDA.chr5.03707* | | | | *CNL-FNU.ctg53.289* | *CNL-FvH4.6g47050.1* | | *RNL-FPE.chr5.2037* | | | | | | | *RN-FNU.ctg52.52* | | | | | |
| *TNL-FVI.CHR5.2738* | *TNL-FNU.ctg160.483* | | | | *CNL-FNU.ctg53.85* | *CNL-FDA.chr6.13561* | | *RNL-FPE.chr5.2037* | | | | | | | *RNL-FII.chr1.1266* | | | | | |
| *TNL-FVI.CHR5.2738* | *TNL-FvH4.5g32050.1* | | | | *CNL-FNU.ctg53.85* | *CNL-FvH4.6g47050.1* | | *RNL-FPE.chr5.2037* | | | | | | | *RNL-FMA.chr1.1807* | | | | | |
| *TNL-FVI.CHR5.3055* | *TNL-FMA.chr5.2425* | | | | *CNL-FNU.ctg64.14.1* | *CNL-FNG.chr6.2058* | | *RNL-FPE.chr5.2037* | | | | | | | *RNL-FvH4.1g15220.1* | | | | | |
| *TNL-FVI.CHR5.3055* | *TNL-FNG.chr5.2639* | | | | *CNL-FNU.ctg81.894* | *CNL-FMA.chr7.880* | | *RNL-FPE.chr5.2061* | | | | | | | *RN-FNU.ctg109.400* | | | | | |
| *TNL-FVI.CHR5.3055* | *TNL-FvH4.5g35770.1* | | | | *CNL-FNU.ctg84.614* | *CNL-FDA.chr6.13561* | | *RNL-FPE.chr5.2061* | | | | | | | *RNL-FvH4.1g15220.1* | | | | | |
| *TNL-FVI.CHR5.3285* | *TNL-FDA.chr5.12803* | | | | *CNL-FNU.ctg84.614* | *CNL-FvH4.6g47050.1* | | *RNL-FPE.chr6.3526* | | | | | | | *RNL-FDA.chr1.22182* | | | | | |
| *TNL-FVI.CHR5.3285* | *TNL-FII.chr5.3330* | | | | *CNL-FPE.chr0.563* | *CNL-FNU.ctg114.210* | | *RNL-FPE.chr6.3526* | | | | | | | *RNL-FMA.chr1.1806* | | | | | |
| *TNL-FVI.CHR5.3285* | *TNL-FNG.chr2.105* | | | | *CNL-FPE.chr0.563* | *CNL-FvH4.7g08100.1* | | *RNL-FPE.chr6.3526* | | | | | | | *RNL-FvH4.1g15390.1* | | | | | |
| *TNL-FVI.CHR5.3285* | *TNL-FvH4.5g38680.1* | | | | *CNL-FPE.chr1.1624* | *CN-FvH4.3g21750.1* | | *RNL-FPE.chr6.5624* | | | | | | | *RN-FDA.chr6.13755* | | | | | |
| *TNL-FVI.CHR5.808* | *TNL-FDA.chr5.03488* | | | | *CNL-FPE.chr1.1624* | *CNL-FvH4.5g34680.1* | | *RNL-FPE.chr6.5624* | | | | | | | *RN-FNU.ctg52.52* | | | | | |
| *TNL-FVI.CHR5.808* | *TNL-FII.chr5.2969* | | | | *CNL-FPE.chr1.2047* | *N-FNU.ctg161.240* | | *RNL-FPE.chr6.5624* | | | | | | | *RNL-FII.chr1.1266* | | | | | |
| *TNL-FVI.CHR5.808* | *TNL-FNU.ctg160.483* | | | | *CNL-FPE.chr1.2047* | *CNL-FNU.ctg114.210* | | *RNL-FPE.chr6.5624* | | | | | | | *RNL-FNG.chr6.3533* | | | | | |
| *TNL-FVI.CHR6.256* | *TNL-FvH4.3g45380.1* | | | | *CNL-FPE.chr1.871* | *CNL-FvH4.6g49940.1* | | *RNL-FPE.chr6.5624* | | | | | | | *RNL-FvH4.1g15220.1* | | | | | |
| *TNL-FVI.CHR6.256* | *TNL-FII.chr7.1130* | | | | *CNL-FPE.chr1.871* | *CNL-FNU.ctg114.210* | | *RNL-FPE.chr6.6304* | | | | | | | *RNL-FvH4.1g15230.1* | | | | | |
| *TNL-FVI.CHR6.256* | *TNL-FMA.chr5.2425* | | | | *CNL-FPE.chr2.3627* | *CNL-FvH4.6g49940.1* | | *RNL-FvH4.1g15090.1* | | | | | | | *N-FNU.ctg172.22* | | | | | |
| *TNL-FVI.CHR6.256* | *TNL-FNG.chr6.244* | | | | *CNL-FPE.chr2.3627* | *CNL-FvH4.6g34770.1* | | *RNL-FvH4.1g15090.1* | | | | | | | *RNL-FDA.chr1.22182* | | | | | |
| *TNL-FVI.CHR7.1039* | *TNL-FvH4.6g03250.1* | | | | *CNL-FPE.chr2.397* | *N-FNU.ctg57.541* | | *RNL-FvH4.1g15090.1* | | | | | | | *RNL-FII.chr1.1265* | | | | | |
| *TNL-FVI.CHR7.1039* | *TNL-FII.chr6.136* | | | | *CNL-FPE.chr2.397* | *CNL-FNU.ctg82.313* | | *RNL-FvH4.1g15120.1* | | | | | | | *CNL-FNU.ctg172.27* | | | | | |
|  |  |  |  |  | *CNL-FPE.chr3.1482* | *CNL-FvH4.4g25750.1* | | *RNL-FvH4.1g15130.1* | | | | | | | *N-FNU.ctg172.28* | | | | | |
|  |  |  |  |  | *CNL-FPE.chr3.1482* | *CNL-FII.chr4.1808* | | *RNL-FvH4.1g15160.1* | | | | | | | *RNL-FII.chr1.1269* | | | | | |
|  |  |  |  |  | *CNL-FPE.chr3.2618* | *CNL-FNG.chr4.1917* | | *RNL-FvH4.1g15160.1* | | | | | | | *RNL-FPE.chr6.6304* | | | | | |
|  |  |  |  |  | *CNL-FPE.chr3.2618* | *CNL-FNU.ctg82.648* | | *RNL-FvH4.1g15170.1* | | | | | | | *RN-FNG.chr1.1331* | | | | | |
|  |  |  |  |  | *CNL-FPE.chr3.2618* | *CNL-FvH4.7g33440.1* | | *RNL-FvH4.1g15170.1* | | | | | | | *RNL-FMA.chr1.1806* | | | | | |
|  |  |  |  |  | *CNL-FPE.chr3.2618* | *CNL-FII.chr7.998* | | *RNL-FvH4.1g15170.1* | | | | | | | *RNL-FPE.chr6.6304* | | | | | |
|  |  |  |  |  | *CNL-FPE.chr3.4782* | *CNL-FMA.chr7.880* | | *RNL-FvH4.1g15170.1* | | | | | | | *RNL-FMA.chr1.1807* | | | | | |
|  |  |  |  |  | *CNL-FPE.chr3.4782* | *CNL-FNU.ctg81.894* | | *RNL-FvH4.1g15210.1* | | | | | | | *RNL-FDA.chr1.22182* | | | | | |
|  |  |  |  |  | *CNL-FPE.chr3.4782* | *CNL-FvH4.4g29310.1* | | *RNL-FvH4.1g15220.1* | | | | | | | *RNL-FMA.chr1.1806* | | | | | |
|  |  |  |  |  | *CNL-FPE.chr3.4782* | *NL-FDA.chr4.05233* | | *RNL-FvH4.1g15220.1* | | | | | | | *RNL-FDA.chr1.22183* | | | | | |
|  |  |  |  |  | *CNL-FPE.chr3.4782* | *CNL-FvH4.3g04780.1* | | *RNL-FvH4.1g15230.1* | | | | | | | *RNL-FMA.chr1.1806* | | | | | |
|  |  |  |  |  | *CNL-FPE.chr3.535* | *CNL-FDA.chr5.25050* | | *RNL-FvH4.1g15230.1* | | | | | | | *CNL-FNU.ctg172.55* | | | | | |
|  |  |  |  |  | *CNL-FPE.chr4.1619* | *CNL-FNU.ctg158.40* | | *RNL-FvH4.1g15330.1* | | | | | | | *RNL-FMA.chr1.1806* | | | | | |
|  |  |  |  |  | *CNL-FPE.chr4.1619* | *CNL-FvH4.5g23400.1* | | *RNL-FvH4.1g15330.1* | | | | | | | *RNL-FDA.chr1.22182* | | | | | |
|  |  |  |  |  | *CNL-FPE.chr4.1619* | *CN-FvH4.3g16720.1* | | *RNL-FvH4.1g15380.1* | | | | | | | *RNL-FMA.chr1.1806* | | | | | |
|  |  |  |  |  | *CNL-FPE.chr5.124* | *CNL-FNU.ctg165.171* | | *RNL-FvH4.1g15380.1* | | | | | | | *RNL-FDA.chr1.22182* | | | | | |
|  |  |  |  |  | *CNL-FPE.chr5.124* | *CNL-FII.chr3.951* | | *RNL-FvH4.1g15390.1* | | | | | | | *RNL-FMA.chr1.1806* | | | | | |
| Orthologous gene pairsof TNL | | | | | Orthologous gene pairs of non-TNL | | | | | | | | | | | | | | | |
| ID of sequence 1 | ID of sequence 2 | | | | ID of sequence 1 | ID of sequence 2 | | ID of sequence 1 | | | | | | ID of sequence 2 | | | | | | |
|  |  |  |  |  | *CNL-FPE.chr5.3277* | *CNL-FvH4.3g11470.1* | | *RNL-FvH4.1g15390.1* | | | | | | | *RNL-FNU.ctg172.65* | | | | | |
|  |  |  |  |  | *CNL-FPE.chr5.3277* | *CNL-FNU.ctg170.24* | | *RNL-FvH4.1g15500.1* | | | | | | | *RNL-FDA.chr6.13739* | | | | | |
|  |  |  |  |  | *CNL-FPE.chr6.1273* | *N-FvH4.1g12710.1* | | *RNL-FvH4.1g17470.1* | | | | | | | *RNL-FNG.chr6.4113* | | | | | |
|  |  |  |  |  | *CNL-FPE.chr6.1273* | *CNL-FNU.ctg145.8* | | *RNL-FvH4.1g17470.1* | | | | | | | *RNL-FNU.ctg122.114* | | | | | |
|  |  |  |  |  | *CNL-FPE.chr6.1639* | *CNL-FvH4.7g02890.1* | | *RNL-FvH4.2g40720.1* | | | | | | | *RN-FII.chr6.3558* | | | | | |
|  |  |  |  |  | *CNL-FPE.chr6.1639* | *CNL-FII.chr3.951* | | *RNL-FvH4.4g31860.1* | | | | | | | *RNL-FDA.chr4.04994* | | | | | |
|  |  |  |  |  | *CNL-FPE.chr6.2574* | *CNL-FvH4.3g11470.1* | | *RNL-FvH4.4g31860.1* | | | | | | | *RNL-FNG.chr6.3533* | | | | | |
|  |  |  |  |  | *CNL-FPE.chr6.2574* | *CNL-FII.chr7.998* | | *RNL-FvH4.4g31860.1* | | | | | | | *RN-FDA.chr6.13755* | | | | | |
|  |  |  |  |  | *CNL-FPE.chr7.1015* | *CNL-FNU.ctg81.894* | | *RNL-FvH4.6g18970.1* | | | | | | | *RN-FNU.ctg52.52* | | | | | |
|  |  |  |  |  | *CNL-FPE.chr7.1015* | *CNL-FvH4.7g11380.1* | | *RNL-FvH4.6g18970.1* | | | | | | | *RNL-FPE.chr5.2037* | | | | | |
|  |  |  |  |  | *CNL-FPE.chr7.1015* | *CNL-FII.chr7.789* | | *RNL-FvH4.6g18970.1* | | | | | | | *RN-FII.chr6.3558* | | | | | |
|  |  |  |  |  | *CNL-FPE.chr7.1124* | *CNL-FNU.ctg114.210* | | *RNL-FvH4.6g18970.1* | | | | | | | *CNL-FII.chr1.1471* | | | | | |
|  |  |  |  |  | *CNL-FPE.chr7.1124* | *CNL-FvH4.7g15240.1* | | *RNL-FvH4.6g42380.1* | | | | | | | *RNL-FDA.chr6.13739* | | | | | |
|  |  |  |  |  | *CNL-FPE.chr7.1124* | *CNL-FII.chr7.55* | | *RNL-FvH4.6g50100.1* | | | | | | | *CNL-FII.chr1.1471* | | | | | |
|  |  |  |  |  | *CNL-FPE.chr7.1571* | *CNL-FvH4.7g27330.1* | | *RNL-FvH4.6g50100.1* | | | | | | | *RNL-FDA.chr6.13739* | | | | | |
|  |  |  |  |  | *CNL-FPE.chr7.1571* | *CN-FvH4.2g05600.1* | | *RNL-FvH4.6g50110.1* | | | | | | | *RNL-FNG.chr6.4114* | | | | | |
|  |  |  |  |  | *CNL-FPE.chr7.1798* | *CNL-FII.chr3.1811* | | *RNL-FvH4.6g50110.1* | | | | | | | *CNL-FII.chr1.1471* | | | | | |
|  |  |  |  |  | *CNL-FPE.chr7.1798* | *NL-FvH4.7g00850.1* | | *RNL-FvH4.6g50110.1* | | | | | | | *RNL-FDA.chr6.13739* | | | | | |
|  |  |  |  |  | *CNL-FPE.chr7.237* | *CNL-FNU.ctg114.210* | | *RNL-FvH4.6g50130.1* | | | | | | | *RNL-FNG.chr6.4114* | | | | | |
|  |  |  |  |  | *CNL-FPE.chr7.333* | *CNL-FvH4.6g49940.1* | | *RNL-FvH4.6g50130.1* | | | | | | | *CNL-FII.chr1.1471* | | | | | |
|  |  |  |  |  | *CNL-FPE.chr7.333* | *CNL-FNU.ctg114.210* | | *RNL-FvH4.6g50130.1* | | | | | | | *RN-FNU.ctg52.52* | | | | | |
|  |  |  |  |  | *CNL-FPE.chr7.335* | *CNL-FvH4.6g49940.1* | | *RNL-FvH4.6g50140.1* | | | | | | | *RNL-FMA.chr1.1806* | | | | | |
|  |  |  |  |  | *CNL-FPE.chr7.335* | *CNL-FDA.chr6.02010* | | *RNL-FvH4.6g50310.1* | | | | | | | *RNL-FNG.chr6.4113* | | | | | |
|  |  |  |  |  | *CNL-FvH4.1g23030.1* | *CNL-FII.chr3.951* | | *RNL-FvH4.6g50310.1* | | | | | | | *CNL-FNU.ctg172.55* | | | | | |
|  |  |  |  |  | *CNL-FvH4.2g00960.1* | *CNL-FII.chr3.953* | | *RNL-FvH4.6g50310.1* | | | | | | | *RNL-FDA.chr1.22196* | | | | | |
|  |  |  |  |  | *CNL-FvH4.2g13150.1* | *CNL-FDA.chr6.13908* | | *RNL-FVI.CHR1.1325* | | | | | | | *RNL-FMA.chr1.1806* | | | | | |
|  |  |  |  |  | *CNL-FvH4.2g17630.1* | *CNL-FDA.chr6.13908* | | *RNL-FVI.CHR1.1325* | | | | | | | *RNL-FNG.chr1.1333* | | | | | |
|  |  |  |  |  | *CNL-FvH4.2g17640.1* | *CNL-FDA.chr2.12208* | | *RNL-FVI.CHR1.1325* | | | | | | | *RNL-FvH4.1g15330.1* | | | | | |
|  |  |  |  |  | *CNL-FvH4.2g36830.1* | *CNL-FDA.chr2.12218* | | *RNL-FVI.CHR1.1325* | | | | | | | *RNL-FDA.chr1.22201* | | | | | |
|  |  |  |  |  | *CNL-FvH4.2g36860.1* | *CNL-FNU.ctg47.220* | | *RNL-FVI.CHR1.1325* | | | | | | | *RNL-FII.chr1.1269* | | | | | |
|  |  |  |  |  | *CNL-FvH4.3g00820.1* | *CNL-FDA.chr6.14104* | | *RNL-FVI.CHR1.1341* | | | | | | | *RNL-FNG.chr1.1344* | | | | | |
|  |  |  |  |  | *CNL-FvH4.3g04780.1* | *CNL-FDA.chr6.14104* | | *RNL-FVI.CHR1.1341* | | | | | | | *RNL-FNU.ctg172.65* | | | | | |
|  |  |  |  |  | *CNL-FvH4.3g05070.1* | *CNL-FII.chr3.408* | | *RNL-FVI.CHR1.1341* | | | | | | | *RNL-FvH4.1g15500.1* | | | | | |
|  |  |  |  |  | *CNL-FvH4.3g05070.1* | *CNL-FPE.chr3.535* | | *RNL-FVI.CHR1.1341* | | | | | | | *RNL-FDA.chr2.11777* | | | | | |
|  |  |  |  |  | *CNL-FvH4.3g05070.1* | *CNL-FII.chr3.951* | | *RNL-FVI.CHR1.1341* | | | | | | | *RNL-FII.chr2.3467* | | | | | |
|  |  |  |  |  | *CNL-FvH4.3g11470.1* | *CNL-FDA.chr4.11333* | | *RNL-FVI.CHR2.4287* | | | | | | | *RNL-FMA.chr2.3489* | | | | | |
|  |  |  |  |  | *CNL-FvH4.4g06020.1* | *CNL-FII.chr4.377* | | *RNL-FVI.CHR2.4287* | | | | | | | *RNL-FNG.chr2.3537* | | | | | |
|  |  |  |  |  | *CNL-FvH4.4g06020.1* | *CNL-FNG.chr0.411* | | *RNL-FVI.CHR2.4287* | | | | | | | *RNL-FNU.ctg122.114* | | | | | |
|  |  |  |  |  | *CNL-FvH4.4g06020.1* | *CNL-FMA.chr4.679* | | *RNL-FVI.CHR2.4287* | | | | | | | *RNL-FPE.chr0.2431* | | | | | |
|  |  |  |  |  | *CNL-FvH4.4g06030.1* | *CNL-FNG.chr0.411* | | *RNL-FVI.CHR2.4287* | | | | | | | *RNL-FvH4.2g40720.1* | | | | | |
|  |  |  |  |  | *CNL-FvH4.4g06030.1* | *NL-FNU.ctg84.211* | | *RNL-FVI.CHR2.4287* | | | | | | | *RNL-FDA.chr1.22184* | | | | | |
|  |  |  |  |  | *CNL-FvH4.4g16700.1* | *CNL-FNU.ctg82.648* | | *RNL-FVI.CHR2.4287* | | | | | | | *RNL-FII.chr2.1682* | | | | | |
| Orthologous gene pairsof TNL | | | | | Orthologous gene pairs of non-TNL | | | | | | | | | | | | | | | |
| ID of sequence 1 | ID of sequence 2 | | | | ID of sequence 1 | ID of sequence 2 | | ID of sequence 1 | | | | | | ID of sequence 2 | | | | | | |
|  |  |  |  |  | *CNL-FvH4.4g22940.1* | *CNL-FPE.chr2.3627* | | *RNL-FVI.CHR6.3601* | | | | | *RNL-FMA.chr2.1492* | | | | | | |
|  |  |  |  |  | *CNL-FvH4.4g22940.1* | *CNL-FNU.ctg82.313* | | *RNL-FVI.CHR6.3601* | | | | | *RNL-FNG.chr2.1787* | | | | | | |
|  |  |  |  |  | *CNL-FvH4.4g25750.1* | *NL-FNU.ctg82.264* | | *RNL-FVI.CHR6.3601* | | | | | *RNL-FPE.chr5.2061* | | | | | | |
|  |  |  |  |  | *CNL-FvH4.4g26300.1* | *CNL-FII.chr7.998* | | *RNL-FVI.CHR6.3601* | | | | | *RNL-FvH4.1g15160.1* | | | | | | |
|  |  |  |  |  | *CNL-FvH4.4g29310.1* | *CNL-FMA.chr7.880* | |  | | | | |  | | | | |  |  | |
|  |  |  |  |  | *CNL-FvH4.4g293* | *CNL-FNU.ctg81.894* | |  | | | | |  | | | | |  |  | |

**Table S5** Results of calculation of NLR gene family Ka, Ks, Ka/Ks

| orthologous genes | | | | | | paralogous gene | | | | | | | | | | | | | |
| --- | --- | --- | --- | --- | --- | --- | --- | --- | --- | --- | --- | --- | --- | --- | --- | --- | --- | --- | --- |
| Seq_ 1 | | Seq_2 | | Ka | | Ks | | Ka/Ks | | Seq_ 1 | | Seq_2 | | Ka | | Ks | | | Ka/Ks |
| CN-FDA.chr1.06861 | | CN-FNG.chr1.338 | | 0.021794 | | 0.049935 | | 0.436441 | | CN-FDA.chr2.11532 | | CNL-FDA.chr6.02198 | | 0.184214 | | 0.314359 | | | 0.585999 |
| CN-FDA.chr2.11532 | | CN-FvH4.1g02740.1 | | 0.11774 | | 0.15742 | | 0.747937 | | CN-FDA.chr2.12212 | | CNL-FDA.chr2.12208 | | 0.17922 | | 0.211681 | | | 0.846652 |
| CN-FDA.chr2.11532 | | CNL-FNG.chr2.1430 | | 0.178504 | | 0.270523 | | 0.659848 | | CN-FDA.chr2.12212 | | CN-FDA.chr2.12213 | | 0.142256 | | 0.228582 | | | 0.622342 |
| CN-FDA.chr2.11532 | | CN-FII.chr6.198 | | 0.167396 | | 0.307245 | | 0.544829 | | CN-FDA.chr2.12217 | | CNL-FDA.chr2.12218 | | 0.27129 | | 0.41083 | | | 0.660346 |
| CN-FDA.chr2.12213 | | CNL-FvH4.2g36860.1 | | 0.152021 | | 0.289201 | | 0.525658 | | CN-FDA.chr3.05842 | | TN-FDA.chr3.05844 | | 0.137443 | | 0.168992 | | | 0.813314 |
| CN-FDA.chr2.17950 | | CN-FMA.chr2.312 | | 0.019902 | | 0.011719 | | 1.698241 | | CN-FDA.chr3.05842 | | N-FDA.chr3.20836 | | 0.082873 | | 0.140065 | | | 0.591672 |
| CN-FDA.chr2.17950 | | CN-FvH4.2g05600.1 | | 0.13627 | | 0.199168 | | 0.684196 | | CN-FDA.chr5.25046 | | CNL-FDA.chr5.25047 | | 0.194602 | | 0.470301 | | | 0.413781 |
| CN-FDA.chr2.17950 | | CN-FNU.ctg104.161 | | 0.083717 | | 0.160039 | | 0.523107 | | CN-FDA.chr6.13604 | | CNL-FDA.chr6.13561 | | 0.104497 | | 0.084492 | | | 1.236776 |
| CN-FDA.chr2.17950 | | CNL-FPE.chr7.1798 | | 0.096045 | | 0.190031 | | 0.505418 | | CN-FDA.chr7.00678 | | CN-FDA.chr7.00892 | | 0.060202 | | 0.112406 | | | 0.535574 |
| CN-FDA.chr2.17950 | | CN-FII.chr2.453 | | 0.035087 | | 0.074405 | | 0.471568 | | CN-FII.chr3.950 | | CNL-FII.chr3.954 | | 0.132033 | | 0.252063 | | | 0.523807 |
| CN-FDA.chr5.25046 | | CNL-FNU.ctg158.46 | | 0.135762 | | 0.215604 | | 0.629683 | | CN-FII.chr3.950 | | CNL-FII.chr3.956 | | 0.159336 | | 0.320431 | | | 0.497257 |
| CN-FDA.chr5.25046 | | CNL-FII.chr5.2007 | | 0.034017 | | 0.062051 | | 0.548217 | | CN-FII.chr3.950 | | CNL-FII.chr3.953 | | 0.136778 | | 0.397487 | | | 0.344107 |
| CN-FDA.chr5.25046 | | CN-FvH4.5g23460.1 | | 0.010113 | | 0.055071 | | 0.183638 | | CN-FII.chr3.950 | | CNL-FII.chr3.951 | | 0.14448 | | 0.49178 | | | 0.293789 |
| CN-FDA.chr6.13604 | | CNL-FNU.ctg53.85 | | 0.060935 | | 0.041049 | | 1.484438 | | CN-FII.chr3.950 | | CN-FII.chr3.955 | | 0.14484 | | 0.503641 | | | 0.287586 |
| CN-FDA.chr6.16615 | | CN-FNG.chr6.3269 | | 0.030345 | | 0.029836 | | 1.017072 | | CN-FII.chr3.955 | | CNL-FII.chr3.951 | | 0.078702 | | 0.128996 | | | 0.610108 |
| CN-FDA.chr6.16615 | | CN-FNU.ctg57.114 | | 0.038982 | | 0.039371 | | 0.99012 | | CN-FII.chr3.955 | | CNL-FII.chr3.956 | | 0.194898 | | 0.443583 | | | 0.439372 |
| CN-FDA.chr6.16615 | | CN-FPE.chr6.3007 | | 0.032006 | | 0.034478 | | 0.928286 | | CN-FII.chr6.2258 | | CN-FII.chr6.2548 | | 0.005782 | | 0.009354 | | | 0.618188 |
| CN-FDA.chr6.16615 | | CN-FII.chr6.3270.2 | | 0.031844 | | 0.048619 | | 0.654974 | | CN-FMA.chr2.312 | | CNL-FMA.chr2.328 | | 0.139826 | | 0.222442 | | | 0.628596 |
| CN-FDA.chr6.17288 | | CN-FNG.chr6.1594 | | 0.23245 | | 0.320362 | | 0.725587 | | CN-FMA.chr3.1343 | | CNL-FMA.chr3.1344 | | 0.465799 | | 0.842583 | | | 0.552822 |
| CN-FDA.chr6.17288 | | CN-FPE.chr6.1820 | | 0.23424 | | 0.325901 | | 0.718744 | | CN-FNG.chr0.49 | | CN-FNG.chr5.2696 | | 0.003585 | | 0.006129 | | | 0.584999 |
| CN-FDA.chr6.17288 | | CN-FII.chr6.2548 | | 0.240395 | | 0.356255 | | 0.674783 | | CN-FNU.ctg144.27 | | CNL-FNU.ctg145.8 | | 0.00576 | | 0.006473 | | | 0.889919 |
| CN-FDA.chr6.17288 | | CN-FNU.ctg65.105 | | 0.090363 | | 0.166276 | | 0.543451 | | CN-FNU.ctg158.39 | | CNL-FNU.ctg158.40 | | 0.113917 | | 0.406325 | | | 0.280359 |
| CN-FDA.chr6.17380 | | CN-FPE.chr6.470 | | 0.005042 | | 0.017556 | | 0.287181 | | CN-FNU.ctg53.159 | | CNL-FNU.ctg53.85 | | 0.033737 | | 0.024057 | | | 1.402355 |
| CN-FDA.chr6.17380 | | CN-FNU.ctg64.1 | | 0.004124 | | 0.019147 | | 0.215392 | | CN-FNU.ctg53.159 | | CNL-FNU.ctg53.289 | | 0.035886 | | 0.037961 | | | 0.945337 |
| CN-FDA.chr6.17380 | | CN-FMA.chr6.2734 | | 0.006427 | | 0.030518 | | 0.210595 | | CN-FNU.ctg53.159 | | CN-FNU.ctg54.29 | | 0.042185 | | 0.052377 | | | 0.805405 |
| CN-FDA.chr6.17380 | | CN-FII.chr6.2648 | | 0.006626 | | 0.048171 | | 0.137544 | | CN-FNU.ctg53.159 | | CN-FNU.ctg53.173 | | 0.048697 | | 0.081112 | | | 0.600358 |
| CN-FDA.chr6.19192 | | CNL-FMA.chr6.3395 | | 0.051594 | | 0.055048 | | 0.937257 | | CN-FNU.ctg53.159 | | CNL-FNU.ctg53.185 | | 0.037827 | | 0.067823 | | | 0.557741 |
| CN-FDA.chr6.19192 | | CNL-FPE.chr2.397 | | 0.044392 | | 0.056101 | | 0.791282 | | CN-FNU.ctg54.29 | | CNL-FNU.ctg53.139 | | 0.095409 | | 0.148415 | | | 0.642852 |
| CN-FDA.chr6.19192 | | N-FNU.ctg57.541 | | 0.041677 | | 0.053594 | | 0.777647 | | CN-FNU.ctg54.29 | | CNL-FNU.ctg53.289 | | 0.071317 | | 0.115284 | | | 0.618621 |
| CN-FDA.chr6.20969 | | CN-FMA.chr6.1745 | | 0.026931 | | 0.033006 | | 0.815945 | | CN-FNU.ctg54.29 | | CNL-FNU.ctg53.185 | | 0.072359 | | 0.117332 | | | 0.616704 |
| CN-FDA.chr6.20969 | | CN-FPE.chr0.885 | | 0.018951 | | 0.024736 | | 0.766143 | | CN-FPE.chr2.3023 | | CNL-FPE.chr6.2574 | | 0.094104 | | 0.12219 | | | 0.770144 |
| CN-FDA.chr6.20969 | | CN-FNU.ctg71.38 | | 0.018944 | | 0.028979 | | 0.653722 | | CN-FPE.chr2.3023 | | CNL-FPE.chr5.3277 | | 0.09006 | | 0.12595 | | | 0.715052 |
| CN-FDA.chr6.20969 | | CN-FII.chr6.1890 | | 0.016706 | | 0.033097 | | 0.504753 | | CN-FPE.chr4.1090 | | CNL-FPE.chr4.1619 | | 0.034516 | | 0.089803 | | | 0.384358 |
| CN-FDA.chr6.20969 | | CN-FNG.chr6.1541 | | 0.035037 | | 0.071174 | | 0.492272 | | CN-FPE.chr4.1617 | | CNL-FPE.chr4.1619 | | 0.098258 | | 0.426048 | | | 0.230626 |
| CN-FII.chr2.453 | | CN-FNU.ctg104.161 | | 0.02062 | | 0.027436 | | 0.751559 | | CN-FvH4.5g23460.1 | | CNL-FvH4.5g23470.1 | | 0.267902 | | 0.649648 | | | 0.41238 |
| CN-FII.chr2.453 | | CNL-FNG.chr2.416 | | 0.042932 | | 0.077095 | | 0.556869 | | CN-FvH4.6g02790.1 | | CNL-FvH4.6g02760.1 | | 0.039841 | | 0.048743 | | | 0.817375 |
| CN-FII.chr2.453 | | CN-FvH4.2g05600.1 | | 0.036224 | | 0.068183 | | 0.53128 | | CN-FvH4.6g02790.1 | | CN-FvH4.1g02740.1 | | 0.122732 | | 0.248796 | | | 0.493302 |
| CN-FII.chr2.453 | | CN-FMA.chr2.312 | | 0.036224 | | 0.068183 | | 0.53128 | | CN-FvH4.6g02790.1 | | N-FvH4.2g17400.1 | | 0.032419 | | 0.087288 | | | 0.371407 |
| CN-FII.chr2.453 | | CNL-FPE.chr7.1798 | | 0.012632 | | 0.033463 | | 0.37749 | | CN-FvH4.7g23850.1 | | CNL-FvH4.7g22030.1 | | 0.166661 | | 0.460727 | | | 0.361736 |
| CN-FII.chr2.551 | | CN-FPE.chr7.728 | | 0.069284 | | 0.110223 | | 0.62858 | | CNL-FDA.chr1.22189 | | RNL-FDA.chr1.22182 | | 0.1485 | | 0.440231 | | | 0.337323 |
| CN-FII.chr2.551 | | CNL-FNU.ctg104.299 | | 0.017428 | | 0.04049 | | 0.430436 | | CNL-FDA.chr2.12098 | | TNL-FDA.chr5.23994 | | 0.231956 | | 0.319194 | | | 0.726694 |
| orthologous genes | | | | | | paralogous gene | | | | | | | | | | | | | |
| Seq_ 1 | | Seq_2 | | Ka | | Ks | | Ka/Ks | | Seq_ 1 | | Seq_2 | | Ka | | Ks | | | Ka/Ks |
| CN-FII.chr2.551 | | CNL-FNG.chr2.482 | | 0.017426 | | 0.040505 | | 0.43023 | | CNL-FDA.chr2.12098 | | TNL-FDA.chr3.09474 | | 0.194327 | | 0.335415 | | | 0.579363 |
| CN-FII.chr2.551 | | CN-FMA.chr2.503 | | 0.0149 | | 0.054673 | | 0.272522 | | CNL-FDA.chr2.12208 | | CNL-FDA.chr2.12209 | | 0.212272 | | 0.325918 | | | 0.651305 |
| CN-FII.chr3.2661 | | CN-FNU.ctg20.194 | | 0.01782 | | 0.008487 | | 2.099779 | | CNL-FDA.chr3.05890 | | TNL-FDA.chr3.05887 | | 0.185884 | | 0.276297 | | | 0.672768 |
| CN-FII.chr3.2661 | | CNL-FMA.chr3.2744 | | 0.076047 | | 0.104995 | | 0.724293 | | CNL-FDA.chr3.05890 | | TNL-FDA.chr3.05894 | | 0.092424 | | 0.167741 | | | 0.550995 |
| CN-FII.chr3.2661 | | CNL-FPE.chr7.1015 | | 0.018856 | | 0.039248 | | 0.480444 | | CNL-FDA.chr3.05890 | | TNL-FDA.chr3.05883 | | 0.161388 | | 0.370184 | | | 0.435967 |
| CN-FII.chr3.2924 | | CNL-FPE.chr1.1624 | | 0.051987 | | 0.074459 | | 0.698189 | | CNL-FDA.chr3.05895 | | TNL-FDA.chr3.05894 | | 0.212549 | | 0.376534 | | | 0.564489 |
| CN-FII.chr3.2924 | | CNL-FNG.chr3.2531 | | 0.07748 | | 0.112401 | | 0.689319 | | CNL-FDA.chr3.16968 | | CNL-FDA.chr3.16978 | | 0.053911 | | 0.062085 | | | 0.868336 |
| CN-FII.chr3.2924 | | CNL-FMA.chr3.2975 | | 0.037138 | | 0.087719 | | 0.42337 | | CNL-FDA.chr3.16968 | | CNL-FDA.chr7.00307 | | 0.101915 | | 0.235915 | | | 0.431997 |
| CN-FII.chr3.2924 | | CN-FvH4.3g21750.1 | | 0.015238 | | 0.048576 | | 0.313697 | | CNL-FDA.chr3.16968 | | CNL-FDA.chr6.14104 | | 0.109453 | | 0.268824 | | | 0.407155 |
| CN-FII.chr3.950 | | CNL-FvH4.3g11490.1 | | 0.139902 | | 0.516198 | | 0.271023 | | CNL-FDA.chr3.16978 | | CNL-FDA.chr7.00307 | | 0.102062 | | 0.235635 | | | 0.433137 |
| CN-FII.chr3.955 | | N-FPE.chr3.1891 | | 0.122095 | | 0.32331 | | 0.37764 | | CNL-FDA.chr4.11333 | | CNL-FDA.chr5.24089 | | 0.063915 | | 0.098389 | | | 0.649615 |
| CN-FII.chr4.1180 | | CN-FPE.chr7.1432 | | 0.023106 | | 0.089146 | | 0.259194 | | CNL-FDA.chr5.23812 | | CNL-FDA.chr7.00307 | | 0.204282 | | 0.452607 | | | 0.451346 |
| CN-FII.chr4.1429 | | CN-FNU.ctg84.31 | | 0.03923 | | 0.049113 | | 0.798761 | | CNL-FDA.chr5.24966 | | CNL-FDA.chr5.25047 | | 0.174957 | | 0.541996 | | | 0.322801 |
| CN-FII.chr4.1429 | | CN-FPE.chr4.843 | | 0.041642 | | 0.054361 | | 0.766023 | | CNL-FDA.chr5.25044 | | CNL-FDA.chr5.24966 | | 0.101003 | | 0.252574 | | | 0.399893 |
| CN-FII.chr4.1429 | | CNL-FMA.chr6.1508 | | 0.1252 | | 0.41681 | | 0.300375 | | CNL-FDA.chr5.25047 | | CNL-FDA.chr5.25044 | | 0.187528 | | 0.549967 | | | 0.340981 |
| CN-FII.chr6.1890 | | CN-FPE.chr0.885 | | 0.005511 | | 0.00813 | | 0.677807 | | CNL-FDA.chr5.25049 | | CNL-FDA.chr5.25050 | | 0.26371 | | 1.336558 | | | 0.197305 |
| CN-FII.chr6.1890 | | CN-FMA.chr6.1745 | | 0.013122 | | 0.024343 | | 0.539059 | | CNL-FDA.chr6.02058 | | CNL-FDA.chr6.02056 | | 0.040038 | | 0.066226 | | | 0.604569 |
| CN-FII.chr6.1890 | | CN-FNG.chr6.1541 | | 0.034616 | | 0.070741 | | 0.489332 | | CNL-FDA.chr6.02459 | | TNL-FDA.chr6.02345 | | 0.059766 | | 0.07007 | | | 0.852937 |
| CN-FII.chr6.1890 | | CN-FNU.ctg71.38 | | 0.005431 | | 0.016239 | | 0.334438 | | CNL-FDA.chr6.13872 | | CNL-FDA.chr7.01119 | | 0.095709 | | 0.119078 | | | 0.803753 |
| CN-FII.chr6.198 | | CNL-FvH4.6g02760.1 | | 0.069626 | | 0.07295 | | 0.954435 | | CNL-FDA.chr6.13872 | | CNL-FDA.chr7.00809 | | 0.099289 | | 0.171896 | | | 0.577612 |
| CN-FII.chr6.198 | | CNL-FNG.chr2.1430 | | 0.063528 | | 0.119299 | | 0.53251 | | CNL-FDA.chr6.13908 | | CNL-FDA.chr6.02010 | | 0.158022 | | 0.327202 | | | 0.482951 |
| CN-FII.chr6.2258 | | CN-FNG.chr6.1594 | | 0.035715 | | 0.044171 | | 0.808563 | | CNL-FDA.chr6.14104 | | CNL-FDA.chr7.00307 | | 0.144068 | | 0.314688 | | | 0.457813 |
| CN-FII.chr6.2258 | | CN-FMA.chr6.2632 | | 0.026035 | | 0.034957 | | 0.744789 | | CNL-FDA.chr6.26146 | | CNL-FDA.chr6.26337 | | 0.163412 | | 0.411183 | | | 0.397419 |
| CN-FII.chr6.2258 | | CN-FNU.ctg65.105 | | 0.04179 | | 0.061096 | | 0.684016 | | CNL-FDA.chr7.00809 | | CNL-FDA.chr7.01119 | | 0.075711 | | 0.117366 | | | 0.645085 |
| CN-FII.chr6.2258 | | CN-FDA.chr6.17288 | | 0.204264 | | 0.314386 | | 0.649724 | | CNL-FDA.chr7.01259 | | CNL-FDA.chr7.01250 | | 0.054381 | | 0.08217 | | | 0.66182 |
| CN-FII.chr6.2258 | | CN-FPE.chr6.1820 | | 0.033937 | | 0.05359 | | 0.633282 | | CNL-FII.chr3.1384 | | CNL-FII.chr3.1385 | | 0.356576 | | 0.781216 | | | 0.456437 |
| CN-FII.chr6.2548 | | CN-FMA.chr6.2632 | | 0.031777 | | 0.038373 | | 0.828113 | | CNL-FII.chr3.953 | | CNL-FII.chr3.954 | | 0.180327 | | 0.415163 | | | 0.434352 |
| CN-FII.chr6.2548 | | CN-FNG.chr6.1594 | | 0.038168 | | 0.052423 | | 0.728072 | | CNL-FII.chr3.953 | | CNL-FII.chr3.951 | | 0.137353 | | 0.340731 | | | 0.403113 |
| CN-FII.chr6.2548 | | CN-FPE.chr6.1820 | | 0.037792 | | 0.053835 | | 0.701998 | | CNL-FII.chr3.954 | | CNL-FII.chr3.951 | | 0.176161 | | 0.377356 | | | 0.466831 |
| CN-FII.chr6.2548 | | CN-FNU.ctg65.105 | | 0.034878 | | 0.057573 | | 0.6058 | | CNL-FII.chr3.954 | | CN-FII.chr3.955 | | 0.193992 | | 0.44388 | | | 0.437038 |
| CN-FII.chr6.2633 | | CNL-FNG.chr6.2058 | | 0.024503 | | 0.037685 | | 0.650205 | | CNL-FII.chr3.956 | | CNL-FII.chr3.954 | | 0.188419 | | 0.354069 | | | 0.532151 |
| CN-FII.chr6.2633 | | CN-FPE.chr6.453 | | 0.041021 | | 0.064487 | | 0.636112 | | CNL-FII.chr3.956 | | CNL-FII.chr3.951 | | 0.17932 | | 0.41535 | | | 0.431733 |
| CN-FII.chr6.2633 | | CN-FNU.ctg65.11 | | 0.026292 | | 0.044107 | | 0.596084 | | CNL-FII.chr5.2001 | | CNL-FII.chr5.3063 | | 0.140779 | | 0.563112 | | | 0.250001 |
| CN-FII.chr6.2633 | | CNL-FMA.chr6.2723 | | 0.026497 | | 0.051138 | | 0.518141 | | CNL-FII.chr5.2001 | | CNL-FII.chr5.2002 | | 0.156083 | | 0.75909 | | | 0.205619 |
| CN-FII.chr6.2648 | | CN-FPE.chr6.470 | | 0.007451 | | 0.047328 | | 0.157434 | | CNL-FII.chr5.2002 | | CNL-FII.chr5.2001 | | 0.156083 | | 0.75909 | | | 0.205619 |
| CN-FII.chr6.2648 | | CN-FMA.chr6.2734 | | 0.006623 | | 0.046235 | | 0.143256 | | CNL-FII.chr5.2002 | | CNL-FII.chr5.2003 | | 0.193891 | | 1.181344 | | | 0.164127 |
| CN-FII.chr6.2648 | | CN-FNU.ctg64.1 | | 0.006068 | | 0.050244 | | 0.120779 | | CNL-FII.chr5.2003 | | CNL-FII.chr5.2001 | | 0.141351 | | 0.559899 | | | 0.252459 |
| CN-FII.chr6.2656 | | CNL-FvH4.6g29480.1 | | 0.128128 | | 0.469899 | | 0.27267 | | CNL-FII.chr5.2003 | | CNL-FII.chr5.2004 | | 0.264651 | | 1.353845 | | | 0.195481 |
| CN-FII.chr6.3270.2 | | CN-FPE.chr6.3007 | | 0.03195 | | 0.03218 | | 0.992852 | | CNL-FII.chr5.2003 | | CNL-FII.chr5.3063 | | 4.54E-04 | | 0.007702 | | | 0.058939 |
| CN-FII.chr6.3270.2 | | CN-FNU.ctg57.114 | | 0.050922 | | 0.072495 | | 0.702417 | | CNL-FII.chr5.2004 | | NL-FII.chr5.3064 | | 0.013855 | | 0.021504 | | | 0.644326 |
| CN-FII.chr6.3270.2 | | CN-FNG.chr6.3269 | | 0.029819 | | 0.054285 | | 0.54931 | | CNL-FII.chr5.2006 | | CNL-FII.chr5.2007 | | 0.148768 | | 0.441838 | | | 0.336702 |
| CN-FMA.chr0.199 | | NL-FvH4.7g00850.1 | | 0.034632 | | 0.046327 | | 0.747561 | | CNL-FII.chr5.2007 | | CNL-FII.chr5.2008 | | 0.174195 | | 0.515955 | | | 0.337616 |
| orthologous genes | | | | | | paralogous gene | | | | | | | | | | | | | |
| Seq_ 1 | | Seq_2 | | Ka | | Ks | | Ka/Ks | | Seq_ 1 | | Seq_2 | | Ka | | Ks | | | Ka/Ks |
| CN-FMA.chr0.199 | | CNL-FPE.chr7.237 | | 0.068219 | | 0.205693 | | 0.331655 | | CNL-FII.chr5.3063 | | NL-FII.chr5.3064 | | 0.275549 | | 1.359988 | | | 0.202612 |
| CN-FMA.chr0.199 | | CNL-FII.chr7.55 | | 0.057626 | | 0.203767 | | 0.282802 | | CNL-FII.chr7.186 | | CNL-FII.chr7.207 | | 0.067018 | | 0.156826 | | | 0.427339 |
| CN-FMA.chr2.312 | | CN-FvH4.2g05600.1 | | 0.003828 | | 0.00275 | | 1.391938 | | CNL-FII.chr7.206 | | CNL-FII.chr7.207 | | 0.284647 | | 0.808854 | | | 0.351914 |
| CN-FMA.chr2.312 | | CNL-FNG.chr2.416 | | 0.02654 | | 0.023566 | | 1.126217 | | CNL-FMA.chr1.1369 | | NL-FMA.chr1.1431 | | 0.006341 | | 0.012126 | | | 0.522921 |
| CN-FMA.chr2.312 | | CN-FNU.ctg104.161 | | 0.039163 | | 0.06908 | | 0.566921 | | CNL-FNG.chr1.545 | | CNL-FNG.chr3.1371 | | 0.065149 | | 0.07143 | | | 0.912077 |
| CN-FMA.chr2.312 | | CNL-FPE.chr7.1798 | | 0.027062 | | 0.077227 | | 0.350417 | | CNL-FNG.chr3.1370 | | CNL-FNG.chr3.1371 | | 0.351601 | | 0.835697 | | | 0.420728 |
| CN-FMA.chr2.503 | | CN-FPE.chr7.728 | | 0.051262 | | 0.136307 | | 0.37608 | | CNL-FNG.chr5.2145 | | CNL-FNG.chr5.2146 | | 0.196687 | | 1.136567 | | | 0.173053 |
| CN-FMA.chr2.503 | | CNL-FNU.ctg104.299 | | 0.006991 | | 0.044032 | | 0.158776 | | CNL-FNG.chr5.2146 | | CNL-FNG.chr5.2147 | | 0.267305 | | 1.366509 | | | 0.195611 |
| CN-FMA.chr2.503 | | CNL-FNG.chr2.482 | | 0.006991 | | 0.044046 | | 0.158711 | | CNL-FNG.chr5.2146 | | CNL-FNG.chr5.2089 | | 0.104938 | | 0.690277 | | | 0.152023 |
| CN-FMA.chr3.1343 | | CNL-FNG.chr3.1370 | | 0.026921 | | 0.038781 | | 0.694177 | | CNL-FNG.chr5.2149 | | CNL-FNG.chr5.2150 | | 0.182208 | | 0.57302 | | | 0.317979 |
| CN-FMA.chr3.1343 | | CNL-FII.chr3.1384 | | 0.021228 | | 0.038063 | | 0.557701 | | CNL-FNG.chr5.2150 | | CNL-FNG.chr5.2227 | | 0.098456 | | 0.25871 | | | 0.380564 |
| CN-FMA.chr3.1343 | | N-FPE.chr5.126 | | 0.023031 | | 0.041414 | | 0.556115 | | CNL-FNG.chr5.654 | | CNL-FNG.chr5.655 | | 0.055932 | | 0.07591 | | | 0.736815 |
| CN-FMA.chr3.1343 | | CNL-FNU.ctg48.6 | | 0.019659 | | 0.038697 | | 0.508008 | | CNL-FNU.ctg145.8 | | NL-FNU.ctg144.28 | | 3.58E-04 | | 0.001239 | | | 0.289075 |
| CN-FMA.chr5.2235 | | CNL-FNU.ctg158.149 | | 0.072941 | | 0.036909 | | 1.976228 | | CNL-FNU.ctg156.8 | | CNL-FNU.ctg155.369 | | 0.001228 | | 0.004108 | | | 0.298945 |
| CN-FMA.chr5.2235 | | CN-FNG.chr5.2228 | | 0.105894 | | 0.071143 | | 1.488455 | | CNL-FNU.ctg158.42 | | CNL-FNU.ctg158.43 | | 0.265004 | | 1.292254 | | | 0.205071 |
| CN-FMA.chr5.2235 | | CN-FvH4.5g24310.1 | | 0.006648 | | 0.019529 | | 0.340388 | | CNL-FNU.ctg158.45 | | CNL-FNU.ctg158.148 | | 0.173489 | | 0.560651 | | | 0.309442 |
| CN-FMA.chr6.1745 | | CN-FPE.chr0.885 | | 0.014424 | | 0.016305 | | 0.884608 | | CNL-FNU.ctg158.45 | | CNL-FNU.ctg158.46 | | 0.152276 | | 0.498053 | | | 0.305742 |
| CN-FMA.chr6.1745 | | CN-FNU.ctg71.38 | | 0.014213 | | 0.024425 | | 0.581902 | | CNL-FNU.ctg158.48 | | CNL-FNU.ctg158.148 | | 0.093398 | | 0.218753 | | | 0.426959 |
| CN-FMA.chr6.1745 | | CN-FNG.chr6.1541 | | 0.044 | | 0.079559 | | 0.55305 | | CNL-FNU.ctg172.27 | | N-FNU.ctg172.28 | | 0.287751 | | 0.705649 | | | 0.407782 |
| CN-FMA.chr6.2632 | | CN-FNG.chr6.1594 | | 0.056388 | | 0.070155 | | 0.803765 | | CNL-FNU.ctg48.6 | | N-FNU.ctg48.4 | | 0.467361 | | 0.979309 | | | 0.477235 |
| CN-FMA.chr6.2632 | | CN-FPE.chr6.1820 | | 0.04453 | | 0.063195 | | 0.704637 | | CNL-FNU.ctg53.139 | | CNL-FNU.ctg53.289 | | 0.075472 | | 0.099971 | | | 0.754939 |
| CN-FMA.chr6.2632 | | CN-FNU.ctg65.105 | | 0.027345 | | 0.053577 | | 0.510387 | | CNL-FNU.ctg53.160 | | CNL-FNU.ctg53.85 | | 0.044117 | | 0.052519 | | | 0.840013 |
| CN-FMA.chr6.2734 | | CN-FPE.chr6.470 | | 0.005549 | | 0.025246 | | 0.219781 | | CNL-FNU.ctg53.160 | | CNL-FNU.ctg53.185 | | 0.034868 | | 0.05782 | | | 0.603033 |
| CN-FMA.chr6.2734 | | CN-FNU.ctg64.1 | | 0.004519 | | 0.027417 | | 0.164843 | | CNL-FNU.ctg53.160 | | CNL-FNU.ctg53.289 | | 0.063442 | | 0.114283 | | | 0.555131 |
| CN-FMA.chr6.4269 | | CNL-FvH4.6g48220.1 | | 0.054375 | | 0.096748 | | 0.56203 | | CNL-FNU.ctg53.185 | | CNL-FNU.ctg53.139 | | 0.052203 | | 0.085526 | | | 0.610377 |
| CN-FMA.chr6.4269 | | CN-FNU.ctg53.150 | | 0.034243 | | 0.064639 | | 0.529752 | | CNL-FNU.ctg53.185 | | CNL-FNU.ctg53.289 | | 0.031941 | | 0.055259 | | | 0.578026 |
| CN-FMA.chr7.847 | | CN-FDA.chr7.14798 | | 0.04413 | | 0.052743 | | 0.836709 | | CNL-FNU.ctg53.85 | | CNL-FNU.ctg53.139 | | 0.052069 | | 0.053109 | | | 0.980419 |
| CN-FMA.chr7.847 | | CN-FvH4.7g11820.1 | | 0.030987 | | 0.045451 | | 0.681774 | | CNL-FNU.ctg53.85 | | CNL-FNU.ctg53.185 | | 0.043941 | | 0.049454 | | | 0.888519 |
| CN-FMA.chr7.847 | | CN-FPE.chr6.3765 | | 0.034738 | | 0.055814 | | 0.6224 | | CNL-FNU.ctg53.85 | | CNL-FNU.ctg53.289 | | 0.031539 | | 0.03965 | | | 0.795439 |
| CN-FMA.chr7.847 | | CNL-FNG.chr7.951 | | 0.026517 | | 0.044185 | | 0.600153 | | CNL-FNU.ctg53.85 | | CN-FNU.ctg54.29 | | 0.082864 | | 0.111822 | | | 0.741033 |
| CN-FMA.chr7.847 | | CNL-FNU.ctg132.293 | | 0.025037 | | 0.043915 | | 0.570127 | | CNL-FNU.ctg84.614 | | CNL-FNU.ctg53.85 | | 0.043823 | | 0.043604 | | | 1.005012 |
| CN-FMA.chr7.847 | | CNL-FII.chr7.1027 | | 0.027926 | | 0.064797 | | 0.430981 | | CNL-FNU.ctg84.614 | | CNL-FNU.ctg53.185 | | 0.046982 | | 0.072974 | | | 0.643818 |
| CN-FNG.chr0.49 | | CN-FNU.ctg161.296 | | 0.010855 | | 0.012352 | | 0.878783 | | CNL-FNU.ctg84.614 | | CNL-FNU.ctg53.160 | | 0.048786 | | 0.085543 | | | 0.570311 |
| CN-FNG.chr0.49 | | CN-FPE.chr5.3016 | | 0.01086 | | 0.018577 | | 0.584596 | | CNL-FNU.ctg84.614 | | CNL-FNU.ctg53.289 | | 0.041443 | | 0.078507 | | | 0.527887 |
| CN-FNG.chr1.338 | | CNL-FPE.chr1.871 | | 0.028649 | | 0.042655 | | 0.671633 | | CNL-FNU.ctg84.614 | | CNL-FNU.ctg53.139 | | 0.061216 | | 0.123438 | | | 0.495925 |
| CN-FNG.chr1.338 | | CNL-FNU.ctg114.210 | | 0.029322 | | 0.057721 | | 0.507991 | | CNL-FPE.chr1.871 | | CNL-FPE.chr7.335 | | 0.124002 | | 0.219862 | | | 0.563998 |
| CN-FNG.chr3.2311 | | NL-FDA.chr6.02338 | | 0.146105 | | 0.161488 | | 0.904743 | | CNL-FPE.chr1.871 | | CNL-FPE.chr7.333 | | 0.127472 | | 0.249655 | | | 0.510593 |
| CN-FNG.chr3.2311 | | TNL-FvH4.3g36130.1 | | 0.034499 | | 0.038594 | | 0.893909 | | CNL-FPE.chr1.871 | | CNL-FPE.chr2.3627 | | 0.238477 | | 0.478929 | | | 0.497937 |
| CN-FNG.chr4.2180 | | CNL-FII.chr4.2076 | | 0.112157 | | 0.1931 | | 0.58082 | | CNL-FPE.chr2.3627 | | CNL-FPE.chr7.333 | | 0.0871 | | 0.091596 | | | 0.950915 |
| CN-FNG.chr4.2180 | | CNL-FMA.chr4.2118 | | 0.128559 | | 0.234765 | | 0.547608 | | CNL-FPE.chr2.3627 | | CNL-FPE.chr7.335 | | 0.100472 | | 0.135621 | | | 0.740833 |
| CN-FNG.chr4.2180 | | CNL-FPE.chr3.1482 | | 0.130066 | | 0.253442 | | 0.513199 | | CNL-FPE.chr7.333 | | CNL-FPE.chr7.335 | | 0.081511 | | 0.126003 | | | 0.6469 |
| CN-FNG.chr4.2180 | | CNL-FNU.ctg82.313 | | 0.06984 | | 0.140427 | | 0.497342 | | CNL-FvH4.2g17630.1 | | CNL-FvH4.2g17640.1 | | 0.07678 | | 0.095694 | | | 0.802355 |
| orthologous genes | | | | | | paralogous gene | | | | | | | | | | | | | |
| Seq_ 1 | | Seq_2 | | Ka | | Ks | | Ka/Ks | | Seq_ 1 | | Seq_2 | | Ka | | Ks | | | Ka/Ks |
| CN-FNG.chr4.2529 | | CNL-FPE.chr3.4782 | | 0.046707 | | 0.035432 | | 1.318224 | | CNL-FvH4.5g23390.1 | | CNL-FvH4.5g23420.1 | | 0.146023 | | 0.584626 | | | 0.249771 |
| CN-FNG.chr4.2529 | | CNL-FNU.ctg81.894 | | 0.040354 | | 0.040934 | | 0.985817 | | CNL-FvH4.5g23420.1 | | CNL-FvH4.5g22710.1 | | 0.106022 | | 0.701146 | | | 0.151213 |
| CN-FNG.chr5.2228 | | N-FvH4.5g24320.1 | | 0.130237 | | 0.191662 | | 0.679514 | | CNL-FvH4.5g23450.1 | | CN-FvH4.5g23460.1 | | 0.193508 | | 0.477707 | | | 0.405077 |
| CN-FNG.chr5.2228 | | CNL-FNU.ctg158.149 | | 0.030087 | | 0.05876 | | 0.512022 | | CNL-FvH4.5g23450.1 | | CNL-FvH4.5g23470.1 | | 0.183731 | | 0.578723 | | | 0.317477 |
| CN-FNG.chr5.2692 | | CN-FNU.ctg161.301 | | 0.029036 | | 0.036965 | | 0.785498 | | CNL-FvH4.6g02730.1 | | CNL-FvH4.6g02760.1 | | 0.059254 | | 0.03809 | | | 1.555627 |
| CN-FNG.chr5.2696 | | CN-FNU.ctg161.296 | | 0.015323 | | 0.008046 | | 1.904527 | | CNL-FvH4.6g02730.1 | | CN-FvH4.6g02790.1 | | 0.045996 | | 0.066048 | | | 0.696402 |
| CN-FNG.chr5.2696 | | CN-FPE.chr5.3016 | | 0.011689 | | 0.007921 | | 1.475761 | | CNL-FvH4.6g02730.1 | | CN-FvH4.1g02740.1 | | 0.140919 | | 0.290402 | | | 0.485254 |
| CN-FNG.chr6.1541 | | CN-FPE.chr0.885 | | 0.035595 | | 0.064626 | | 0.550782 | | CNL-FvH4.6g02760.1 | | CN-FvH4.1g02740.1 | | 0.141165 | | 0.282938 | | | 0.498925 |
| CN-FNG.chr6.1541 | | CN-FNU.ctg71.38 | | 0.036326 | | 0.068742 | | 0.528439 | | CNL-FvH4.6g02820.1 | | CNL-FvH4.6g02760.1 | | 0.05012 | | 0.035713 | | | 1.403426 |
| CN-FNG.chr6.1594 | | CN-FNU.ctg65.105 | | 0.035976 | | 0.065489 | | 0.549346 | | CNL-FvH4.6g02820.1 | | CNL-FvH4.6g02730.1 | | 0.059434 | | 0.048735 | | | 1.21954 |
| CN-FNG.chr6.1594 | | CN-FPE.chr6.1820 | | 0.021823 | | 0.049582 | | 0.44013 | | CNL-FvH4.6g02820.1 | | CN-FvH4.6g02790.1 | | 0.04766 | | 0.075207 | | | 0.63372 |
| CN-FNG.chr6.3269 | | CN-FPE.chr6.3007 | | 0.025752 | | 0.029486 | | 0.873391 | | CNL-FvH4.6g02820.1 | | CN-FvH4.1g02740.1 | | 0.147601 | | 0.272535 | | | 0.541587 |
| CN-FNG.chr6.3269 | | CN-FNU.ctg57.114 | | 0.026799 | | 0.033497 | | 0.80005 | | CNL-FvH4.6g51140.1 | | CNL-FvH4.7g26370.1 | | 0.100859 | | 0.100619 | | | 1.002392 |
| CN-FNG.chr7.813 | | CNL-FII.chr7.789 | | 0.01385 | | 0.024723 | | 0.560229 | | CNL-FvH4.6g51140.1 | | CNL-FvH4.7g23500.1 | | 0.082962 | | 0.098356 | | | 0.843486 |
| CN-FNU.ctg104.161 | | CNL-FPE.chr7.1798 | | 0.048594 | | 0.067657 | | 0.718234 | | CNL-FvH4.7g23500.1 | | CNL-FvH4.7g26370.1 | | 0.08348 | | 0.106557 | | | 0.783436 |
| CN-FNU.ctg104.161 | | CNL-FNG.chr2.416 | | 0.091846 | | 0.170456 | | 0.538825 | | CNL-FvH4.7g27890.1 | | CNL-FvH4.7g27330.1 | | 0.103343 | | 0.247601 | | | 0.417376 |
| CN-FNU.ctg144.27 | | CNL-FvH4.7g02890.1 | | 0.018195 | | 0.013423 | | 1.355489 | | CNL-FVI.CHR1.1874 | | N-FVI.CHR1.1875 | | 0.171309 | | 0.283055 | | | 0.605216 |
| CN-FNU.ctg144.27 | | CNL-FPE.chr6.1639 | | 0.009405 | | 0.008108 | | 1.159879 | | CNL-FVI.CHR7.3757 | | CNL-FVI.CHR7.3758 | | 0.168427 | | 0.36539 | | | 0.460952 |
| CN-FNU.ctg144.27 | | CNL-FNG.chr7.204 | | 0.01351 | | 0.026232 | | 0.514998 | | CNL-FVI.CHR7.3758 | | CN-FVI.CHR7.3759 | | 0.174736 | | 0.325429 | | | 0.53694 |
| CN-FNU.ctg144.27 | | CNL-FII.chr7.257 | | 0.015464 | | 0.0329 | | 0.470044 | | N-FDA.chr1.22180 | | RNL-FDA.chr1.22179 | | 0.351799 | | 0.896504 | | | 0.392412 |
| CN-FNU.ctg158.39 | | CNL-FII.chr5.2001 | | 0.014603 | | 0.02198 | | 0.664387 | | N-FDA.chr2.11533 | | CNL-FDA.chr6.02198 | | 0.573 | | 0.922654 | | | 0.621035 |
| CN-FNU.ctg158.39 | | CNL-FvH4.5g23390.1 | | 0.0224 | | 0.043281 | | 0.517549 | | N-FDA.chr2.12211 | | CN-FDA.chr2.12217 | | 0.182632 | | 0.321164 | | | 0.568657 |
| CN-FNU.ctg158.39 | | CNL-FNG.chr5.2145 | | 0.117125 | | 0.406534 | | 0.288107 | | N-FDA.chr2.12211 | | CNL-FDA.chr2.12209 | | 0.014976 | | 0.03489 | | | 0.429221 |
| CN-FNU.ctg161.301 | | CNL-FDA.chr5.22369 | | 0.031739 | | 0.037021 | | 0.857319 | | N-FDA.chr2.12211 | | CNL-FDA.chr2.12218 | | 0.108607 | | 0.277226 | | | 0.391765 |
| CN-FNU.ctg161.301 | | CNL-FMA.chr5.2388 | | 0.033112 | | 0.04632 | | 0.714838 | | N-FDA.chr2.12211 | | CN-FDA.chr2.12213 | | 0.088799 | | 0.24208 | | | 0.366818 |
| CN-FNU.ctg20.194 | | CNL-FPE.chr7.1015 | | 0.064673 | | 0.065453 | | 0.98808 | | N-FDA.chr3.05877 | | N-FDA.chr3.20666 | | 0.169229 | | 0.286624 | | | 0.590421 |
| CN-FNU.ctg20.194 | | CNL-FMA.chr3.2744 | | 0.122546 | | 0.13505 | | 0.907409 | | N-FDA.chr3.05877 | | TNL-FDA.chr3.05837 | | 0.174111 | | 0.309717 | | | 0.562162 |
| CN-FNU.ctg53.159 | | CN-FDA.chr6.13604 | | 0.03795 | | 0.031021 | | 1.223372 | | N-FDA.chr3.05877 | | NL-FDA.chr3.20676 | | 0.120085 | | 0.229852 | | | 0.522442 |
| CN-FNU.ctg54.29 | | CNL-FDA.chr6.13561 | | 0.099332 | | 0.143235 | | 0.693488 | | N-FDA.chr3.05878 | | N-FDA.chr3.20666 | | 0.169229 | | 0.286624 | | | 0.590421 |
| CN-FNU.ctg63.62 | | CN-FPE.chr6.470 | | 0.001639 | | 0.004274 | | 0.383553 | | N-FDA.chr3.05878 | | TNL-FDA.chr3.05837 | | 0.174111 | | 0.309717 | | | 0.562162 |
| CN-FNU.ctg63.62 | | CN-FDA.chr6.17380 | | 0.004124 | | 0.019147 | | 0.215392 | | N-FDA.chr3.05878 | | NL-FDA.chr3.20676 | | 0.120085 | | 0.229852 | | | 0.522442 |
| CN-FNU.ctg63.62 | | CN-FMA.chr6.2734 | | 0.004519 | | 0.027417 | | 0.164843 | | N-FDA.chr3.20666 | | TNL-FDA.chr3.05837 | | 0.069417 | | 0.1273 | | | 0.545303 |
| CN-FNU.ctg63.62 | | CN-FII.chr6.2648 | | 0.006068 | | 0.050244 | | 0.120779 | | N-FDA.chr3.20666 | | NL-FDA.chr3.20676 | | 0.102071 | | 0.200897 | | | 0.508075 |
| CN-FNU.ctg70.1 | | CN-FDA.chr6.20969 | | 0.018944 | | 0.028979 | | 0.653722 | | N-FDA.chr3.20671 | | TNL-FDA.chr3.20670 | | 0.293032 | | 0.810836 | | | 0.361395 |
| CN-FNU.ctg70.1 | | CN-FPE.chr0.885 | | 0.019829 | | 0.032267 | | 0.614525 | | N-FDA.chr3.20675 | | NL-FDA.chr3.20838 | | 0.27076 | | 0.311562 | | | 0.869042 |
| CN-FNU.ctg70.1 | | CN-FMA.chr6.1745 | | 0.014213 | | 0.024425 | | 0.581902 | | N-FDA.chr3.20742 | | N-FDA.chr3.20833 | | 0.101807 | | 0.197577 | | | 0.515276 |
| CN-FNU.ctg70.1 | | CN-FNG.chr6.1541 | | 0.036326 | | 0.068742 | | 0.528439 | | N-FDA.chr3.20742 | | NL-FDA.chr3.20838 | | 0.050275 | | 0.104853 | | | 0.479485 |
| CN-FNU.ctg70.1 | | CN-FII.chr6.1890 | | 0.005431 | | 0.016239 | | 0.334438 | | N-FDA.chr3.20808 | | TNL-FDA.chr5.03488 | | 0.17754 | | 0.483334 | | | 0.367323 |
| CN-FNU.ctg84.31 | | CNL-FMA.chr6.1508 | | 0.104779 | | 0.443973 | | 0.236003 | | N-FDA.chr3.20832 | | N-FDA.chr3.20836 | | 0.030107 | | 0.028666 | | | 1.050281 |
| CN-FPE.chr0.885 | | CN-FNU.ctg71.38 | | 0.019829 | | 0.032267 | | 0.614525 | | N-FDA.chr3.20832 | | TN-FDA.chr3.05844 | | 0.089672 | | 0.158026 | | | 0.567451 |
| CN-FPE.chr1.928 | | CNL-FvH4.4g22940.1 | | 0.018246 | | 0.020475 | | 0.891171 | | N-FDA.chr3.20832 | | CN-FDA.chr3.05842 | | 0.077864 | | 0.169098 | | | 0.460469 |
| CN-FPE.chr1.928 | | CNL-FNU.ctg82.648 | | 0.021863 | | 0.048993 | | 0.446253 | | N-FDA.chr3.20832 | | NL-FDA.chr3.20838 | | 0.129232 | | 0.393393 | | | 0.328506 |
| orthologous genes | | | | | | paralogous gene | | | | | | | | | | | | | |
| Seq_ 1 | | Seq_2 | | Ka | | Ks | | Ka/Ks | | Seq_ 1 | | Seq_2 | | Ka | | Ks | | | Ka/Ks |
| CN-FPE.chr2.3023 | | CN-FvH4.6g29690.1 | | 0.110367 | | 0.16718 | | 0.660168 | | N-FDA.chr3.20833 | | NL-FDA.chr3.20838 | | 0.084242 | | 0.174028 | | | 0.484071 |
| CN-FPE.chr4.1090 | | CNL-FII.chr5.2002 | | 0.032515 | | 0.083375 | | 0.389988 | | N-FDA.chr3.20833 | | N-FDA.chr3.20832 | | 0.151468 | | 0.450445 | | | 0.336263 |
| CN-FPE.chr4.1090 | | CNL-FNU.ctg158.40 | | 0.034516 | | 0.089803 | | 0.384358 | | N-FDA.chr3.20836 | | TN-FDA.chr3.05844 | | 0.090609 | | 0.121116 | | | 0.748116 |
| CN-FPE.chr4.1090 | | CNL-FNG.chr5.2145 | | 0.030597 | | 0.083064 | | 0.368356 | | N-FDA.chr3.20836 | | NL-FDA.chr3.20838 | | 0.167628 | | 0.391792 | | | 0.427849 |
| CN-FPE.chr4.1617 | | CNL-FvH4.5g23390.1 | | 0.035379 | | 0.075774 | | 0.466897 | | N-FDA.chr3.24469 | | RN-FDA.chr6.13755 | | 0.121148 | | 0.260115 | | | 0.465749 |
| CN-FPE.chr4.1617 | | CNL-FII.chr5.2001 | | 0.031349 | | 0.068678 | | 0.456461 | | N-FDA.chr7.22742 | | TNL-FDA.chr7.00274 | | 0.13274 | | 0.207337 | | | 0.640215 |
| CN-FPE.chr4.1617 | | CNL-FNU.ctg158.40 | | 0.098258 | | 0.431865 | | 0.22752 | | N-FDA.chr7.22742 | | TNL-FDA.chr6.13989 | | 0.337456 | | 0.574896 | | | 0.586986 |
| CN-FPE.chr4.1617 | | CNL-FNG.chr5.2145 | | 0.099934 | | 0.440021 | | 0.227113 | | N-FII.chr2.645 | | TNL-FII.chr5.609 | | 0.186098 | | 0.618284 | | | 0.300991 |
| CN-FPE.chr4.843 | | CN-FNU.ctg84.31 | | 0.093636 | | 0.19414 | | 0.482314 | | N-FII.chr5.1933 | | CNL-FII.chr5.2003 | | 0.155698 | | 0.624444 | | | 0.249338 |
| CN-FPE.chr4.843 | | CNL-FNG.chr6.2406 | | 0.183103 | | 0.508574 | | 0.360033 | | N-FII.chr5.1933 | | CNL-FII.chr5.3063 | | 0.155047 | | 0.637061 | | | 0.243379 |
| CN-FPE.chr4.843 | | CNL-FMA.chr6.1508 | | 0.205999 | | 0.610555 | | 0.337397 | | N-FII.chr6.1246 | | CN-FII.chr4.1429 | | 0.203104 | | 0.578778 | | | 0.350918 |
| CN-FPE.chr5.3016 | | CN-FNU.ctg161.296 | | 0.016531 | | 0.008019 | | 2.061561 | | N-FII.chr7.209 | | CNL-FII.chr7.210 | | 0.232477 | | 0.826545 | | | 0.281264 |
| CN-FPE.chr6.1521 | | CN-FNU.ctg73.111 | | 0.008328 | | 0.023969 | | 0.347438 | | N-FMA.chr2.1406 | | TNL-FMA.chr2.1405 | | 0.034565 | | 0.166483 | | | 0.20762 |
| CN-FPE.chr6.1611 | | CNL-FMA.chr6.1658 | | 0.162151 | | 0.471793 | | 0.343692 | | N-FMA.chr2.311 | | CNL-FMA.chr2.328 | | 0.142812 | | 0.412315 | | | 0.346368 |
| CN-FPE.chr6.1611 | | CNL-FDA.chr6.26146 | | 0.127885 | | 0.411059 | | 0.31111 | | N-FMA.chr2.489 | | N-FMA.chr4.1879 | | 0.092466 | | 0.138348 | | | 0.668357 |
| CN-FPE.chr6.1611 | | CNL-FNU.ctg78.2 | | 0.193143 | | 0.693632 | | 0.278452 | | N-FMA.chr6.2154 | | CN-FMA.chr6.2734 | | 0.004577 | | 0.0031 | | | 1.476469 |
| CN-FPE.chr6.1820 | | CN-FNU.ctg65.105 | | 0.028598 | | 0.055563 | | 0.514707 | | N-FMA.chr6.2173 | | CN-FMA.chr6.2719 | | 0.380976 | | 0.442513 | | | 0.860938 |
| CN-FPE.chr6.3007 | | CN-FNU.ctg57.114 | | 0.029482 | | 0.02603 | | 1.132647 | | N-FNG.chr2.1856 | | RNL-FNG.chr2.1787 | | 0.051972 | | 0.05505 | | | 0.944072 |
| CN-FPE.chr6.3765 | | CNL-FNU.ctg132.293 | | 0.147943 | | 0.265561 | | 0.557094 | | N-FNU.ctg119.817 | | N-FNU.ctg119.818 | | 0.299613 | | 0.474237 | | | 0.631778 |
| CN-FPE.chr6.447 | | N-FNU.ctg65.16 | | 0.171507 | | 0.188721 | | 0.908784 | | N-FNU.ctg119.818 | | N-FNU.ctg119.819 | | 0.395947 | | 0.693435 | | | 0.570994 |
| CN-FPE.chr6.447 | | CN-FII.chr6.2623 | | 0.413658 | | 0.52955 | | 0.781149 | | N-FNU.ctg119.819 | | N-FNU.ctg119.817 | | 0.294659 | | 0.293195 | | | 1.004993 |
| CN-FPE.chr6.453 | | CN-FNU.ctg65.11 | | 0.020365 | | 0.036635 | | 0.5559 | | N-FNU.ctg172.28 | | RN-FNU.ctg172.29 | | 0.395844 | | 1.067739 | | | 0.370731 |
| CN-FPE.chr6.470 | | CN-FNU.ctg64.1 | | 0.001639 | | 0.004274 | | 0.383553 | | N-FNU.ctg48.4 | | CNL-FNU.ctg165.171 | | 0.165813 | | 0.192602 | | | 0.860912 |
| CN-FPE.chr7.2093 | | CN-FNU.ctg161.296 | | 0.016531 | | 0.008019 | | 2.061561 | | N-FPE.chr0.2268 | | CN-FPE.chr0.885 | | 0.079784 | | 0.121353 | | | 0.657458 |
| CN-FPE.chr7.2093 | | CN-FNG.chr5.2696 | | 0.011689 | | 0.007921 | | 1.475761 | | N-FPE.chr3.1891 | | N-FPE.chr3.1892 | | 0.136081 | | 0.344338 | | | 0.395195 |
| CN-FPE.chr7.728 | | CNL-FNU.ctg104.299 | | 0.042904 | | 0.105922 | | 0.405054 | | N-FPE.chr3.1892 | | N-FPE.chr3.1891 | | 0.136081 | | 0.344338 | | | 0.395195 |
| CN-FvH4.1g02740.1 | | CNL-FDA.chr6.02198 | | 0.139435 | | 0.239815 | | 0.581429 | | N-FPE.chr3.1892 | | N-FPE.chr3.1895 | | 0.176754 | | 0.51239 | | | 0.34496 |
| CN-FvH4.1g02740.1 | | CN-FII.chr6.198 | | 0.138998 | | 0.280571 | | 0.495412 | | N-FPE.chr3.3480 | | N-FPE.chr6.572 | | 0.031644 | | 0.044901 | | | 0.704746 |
| CN-FvH4.1g02740.1 | | CNL-FNG.chr2.1430 | | 0.136178 | | 0.288301 | | 0.472345 | | N-FvH4.2g17400.1 | | CNL-FvH4.6g02730.1 | | 0.039151 | | 0.05584 | | | 0.70114 |
| CN-FvH4.2g05600.1 | | CN-FNU.ctg104.161 | | 0.063709 | | 0.132429 | | 0.481083 | | N-FvH4.2g17400.1 | | CNL-FvH4.6g02820.1 | | 0.033816 | | 0.058522 | | | 0.577833 |
| CN-FvH4.3g16720.1 | | CNL-FNU.ctg165.171 | | 0.045512 | | 0.03192 | | 1.425832 | | N-FvH4.2g17400.1 | | CNL-FvH4.6g02760.1 | | 0.037936 | | 0.072978 | | | 0.519822 |
| CN-FvH4.3g21750.1 | | CNL-FNG.chr3.2531 | | 0.080638 | | 0.119087 | | 0.677133 | | N-FvH4.2g17400.1 | | CN-FvH4.1g02740.1 | | 0.128493 | | 0.288562 | | | 0.445287 |
| CN-FvH4.5g23460.1 | | CNL-FNU.ctg158.46 | | 0.13801 | | 0.25005 | | 0.551931 | | N-FvH4.5g21530.1 | | RNL-FvH4.1g15160.1 | | 3.045173 | | 2.129364 | | | 1.430085 |
| CN-FvH4.5g23460.1 | | CNL-FII.chr5.2007 | | 0.03399 | | 0.066884 | | 0.508189 | | N-FvH4.5g21530.1 | | RNL-FvH4.1g15220.1 | | 0.327958 | | 0.599841 | | | 0.546741 |
| CN-FvH4.5g24290.1 | | CNL-FDA.chr5.24966 | | 0.046004 | | 0.060025 | | 0.76642 | | NL-FDA.chr1.18083 | | TNL-FDA.chr3.20747 | | 0.157626 | | 0.248552 | | | 0.634179 |
| CN-FvH4.5g24290.1 | | CNL-FNG.chr5.2227 | | 0.108398 | | 0.150632 | | 0.719621 | | NL-FDA.chr1.18083 | | NL-FDA.chr3.20838 | | 0.13877 | | 0.260115 | | | 0.533492 |
| CN-FvH4.5g24290.1 | | CNL-FNU.ctg158.148 | | 0.044798 | | 0.064417 | | 0.695436 | | NL-FDA.chr3.05440 | | CNL-FDA.chr5.24059 | | 0.074532 | | 0.193782 | | | 0.384619 |
| CN-FvH4.5g24310.1 | | CN-FNG.chr5.2228 | | 0.070372 | | 0.09754 | | 0.721466 | | NL-FDA.chr3.20676 | | TNL-FDA.chr3.05837 | | 0.106136 | | 0.246294 | | | 0.430931 |
| CN-FvH4.5g24310.1 | | CNL-FNU.ctg158.149 | | 0.036197 | | 0.060204 | | 0.601235 | | NL-FDA.chr3.20746 | | TNL-FDA.chr3.20747 | | 0.092347 | | 0.106752 | | | 0.865058 |
| CN-FvH4.6g02790.1 | | CN-FII.chr6.198 | | 0.054976 | | 0.076221 | | 0.721277 | | NL-FDA.chr3.20746 | | NL-FDA.chr3.20838 | | 0.083949 | | 0.106542 | | | 0.787944 |
| CN-FvH4.6g02790.1 | | CNL-FNG.chr2.1430 | | 0.055148 | | 0.106534 | | 0.517655 | | NL-FDA.chr3.20838 | | TNL-FDA.chr3.20747 | | 0.129025 | | 0.190971 | | | 0.675627 |
| CN-FvH4.6g02790.1 | | CNL-FDA.chr6.02198 | | 0.053636 | | 0.104945 | | 0.511081 | | NL-FDA.chr6.02338 | | TNL-FDA.chr6.02345 | | 0.139349 | | 0.239291 | | | 0.582342 |
| orthologous genes | | | | | | paralogous gene | | | | | | | | | | | | | |
| Seq_ 1 | | Seq_2 | | Ka | | Ks | | Ka/Ks | | Seq_ 1 | | Seq_2 | | Ka | | Ks | | | Ka/Ks |
| CN-FvH4.6g15230.1 | | CNL-FDA.chr6.26153 | | 0.061423 | | 0.080047 | | 0.76734 | | NL-FDA.chr7.00111 | | TNL-FDA.chr7.00159 | | 0.095583 | | 0.179183 | | | 0.533436 |
| CN-FvH4.6g15230.1 | | CNL-FNG.chr6.2420 | | 0.111227 | | 0.405754 | | 0.274124 | | NL-FDA.chr7.00111 | | TNL-FDA.chr7.00184 | | 0.096309 | | 0.22489 | | | 0.428248 |
| CN-FvH4.6g29690.1 | | CNL-FPE.chr5.3277 | | 0.068962 | | 0.09442 | | 0.730377 | | NL-FDA.chr7.00111 | | TNL-FDA.chr7.00190 | | 0.081402 | | 0.190411 | | | 0.427506 |
| CN-FvH4.7g02430.1 | | CNL-FII.chr7.207 | | 0.024893 | | 0.028328 | | 0.87875 | | NL-FDA.chr7.00111 | | TNL-FDA.chr7.00189 | | 0.081213 | | 0.205927 | | | 0.394375 |
| CN-FvH4.7g11820.1 | | CNL-FNG.chr7.951 | | 0.015203 | | 0.022191 | | 0.685109 | | NL-FDA.chr7.00705 | | CN-FDA.chr7.00678 | | 0.044267 | | 0.042878 | | | 1.032387 |
| CN-FvH4.7g11820.1 | | CN-FPE.chr6.3765 | | 0.019798 | | 0.029929 | | 0.661511 | | NL-FDA.chr7.00705 | | CN-FDA.chr7.00892 | | 0.061397 | | 0.09921 | | | 0.618864 |
| CN-FvH4.7g11820.1 | | CNL-FNU.ctg132.293 | | 0.011367 | | 0.022232 | | 0.511308 | | NL-FDA.chr7.18714 | | NL-FDA.chr7.14306 | | 0.046063 | | 0.057963 | | | 0.794701 |
| CN-FvH4.7g11820.1 | | CNL-FII.chr7.1027 | | 0.019049 | | 0.040459 | | 0.470811 | | NL-FII.chr1.1268 | | RNL-FII.chr1.1269 | | 0.200494 | | 0.876892 | | | 0.228642 |
| CN-FVI.CHR2.1419 | | CN-FDA.chr6.17380 | | 0.007805 | | 0.038875 | | 0.200766 | | NL-FMA.chr2.856 | | TNL-FMA.chr2.857 | | 0.305642 | | 0.803802 | | | 0.380246 |
| CN-FVI.CHR2.1419 | | CN-FPE.chr6.470 | | 0.005956 | | 0.031236 | | 0.190676 | | NL-FNU.ctg132.328 | | NL-FNU.ctg134.34 | | 0.01644 | | 0.040266 | | | 0.40829 |
| CN-FVI.CHR2.1419 | | CN-FMA.chr6.2734 | | 0.004517 | | 0.025995 | | 0.173768 | | NL-FvH4.4g15190.1 | | CNL-FvH4.1g23030.1 | | 0.226606 | | 0.669547 | | | 0.338447 |
| CN-FVI.CHR2.1419 | | CN-FII.chr6.2648 | | 0.008839 | | 0.056365 | | 0.156812 | | NL-FvH4.6g04350.1 | | TNL-FvH4.6g05610.1 | | 0.135305 | | 0.212907 | | | 0.635515 |
| CN-FVI.CHR2.1419 | | CN-FNU.ctg64.1 | | 0.004927 | | 0.033424 | | 0.14742 | | RN-FDA.chr1.22186 | | RNL-FDA.chr1.22179 | | 0.030674 | | 0.043605 | | | 0.703456 |
| CN-FVI.CHR2.1513 | | CN-FII.chr6.2548 | | 0.068784 | | 0.088568 | | 0.776626 | | RN-FNU.ctg38.153 | | RN-FNU.ctg52.52 | | 0.140929 | | 0.397119 | | | 0.354878 |
| CN-FVI.CHR2.1513 | | CN-FNU.ctg65.105 | | 0.049743 | | 0.066719 | | 0.745555 | | RN-FNU.ctg81.619 | | RN-FNU.ctg55.225 | | 0.164131 | | 0.202665 | | | 0.809862 |
| CN-FVI.CHR2.1513 | | CN-FMA.chr6.2632 | | 0.058605 | | 0.079089 | | 0.741004 | | RNL-FDA.chr1.22179 | | RN-FDA.chr1.22178 | | 0.355071 | | 0.743568 | | | 0.477524 |
| CN-FVI.CHR2.1513 | | CN-FNG.chr6.1594 | | 0.059232 | | 0.081461 | | 0.72712 | | RNL-FDA.chr1.22183 | | RNL-FDA.chr1.22182 | | 0.214215 | | 0.93573 | | | 0.228929 |
| CN-FVI.CHR2.1513 | | CN-FDA.chr6.17288 | | 0.051068 | | 0.074922 | | 0.681614 | | RNL-FDA.chr1.22184 | | RNL-FDA.chr1.22183 | | 0.343018 | | 1.147584 | | | 0.298904 |
| CN-FVI.CHR2.1513 | | CN-FPE.chr6.1820 | | 0.04913 | | 0.075322 | | 0.652265 | | RNL-FDA.chr1.22187 | | RN-FDA.chr1.22186 | | 0.272935 | | 0.571008 | | | 0.477988 |
| CN-FVI.CHR2.1517 | | CNL-FII.chr6.2256 | | 0.112888 | | 0.131646 | | 0.857513 | | RNL-FDA.chr1.22190 | | RNL-FDA.chr1.22183 | | 0.119879 | | 0.471513 | | | 0.254244 |
| CN-FVI.CHR4.1436 | | CNL-FvH4.4g16700.1 | | 0.02539 | | 0.049801 | | 0.509835 | | RNL-FDA.chr1.22190 | | CNL-FDA.chr1.22189 | | 0.219676 | | 0.878165 | | | 0.250153 |
| CN-FVI.CHR4.1436 | | CNL-FII.chr4.1300 | | 0.022756 | | 0.054634 | | 0.416516 | | RNL-FDA.chr4.04994 | | RNL-FDA.chr6.12990 | | 0.076361 | | 0.088858 | | | 0.85936 |
| CN-FVI.CHR4.1436 | | CNL-FDA.chr4.24588 | | 0.021432 | | 0.054678 | | 0.391976 | | RNL-FII.chr1.1265 | | RNL-FII.chr1.1266 | | 0.31023 | | 0.842872 | | | 0.368063 |
| CN-FVI.CHR4.1436 | | NL-FMA.chr4.1335 | | 0.027013 | | 0.071143 | | 0.379692 | | RNL-FII.chr1.1266 | | RNL-FII.chr1.1267 | | 0.244513 | | 0.718046 | | | 0.340525 |
| CN-FVI.CHR4.1436 | | NL-FPE.chr3.4014 | | 0.031838 | | 0.095064 | | 0.334916 | | RNL-FII.chr1.1267 | | NL-FII.chr1.1268 | | 0.261684 | | 0.960306 | | | 0.2725 |
| CN-FVI.CHR5.3012 | | CN-FNU.ctg161.296 | | 0.029843 | | 0.016206 | | 1.841478 | | RNL-FMA.chr1.1806 | | RNL-FMA.chr1.1807 | | 0.265987 | | 0.844174 | | | 0.315086 |
| CN-FVI.CHR5.3012 | | CN-FNG.chr5.2696 | | 0.024813 | | 0.024139 | | 1.027917 | | RNL-FMA.chr1.1807 | | RNL-FMA.chr1.1808 | | 0.286607 | | 0.860271 | | | 0.333159 |
| CN-FVI.CHR5.3012 | | CN-FPE.chr5.3016 | | 0.022436 | | 0.024058 | | 0.932572 | | RNL-FNG.chr1.1336 | | RNL-FNG.chr1.1337 | | 0.477929 | | 1.023627 | | | 0.466898 |
| CN-FVI.CHR6.1776 | | CNL-FNG.chr6.2420 | | 0.02438 | | 0.043125 | | 0.565335 | | RNL-FNG.chr6.4113 | | RNL-FNG.chr6.4114 | | 0.148795 | | 0.288855 | | | 0.515122 |
| CN-FVI.CHR6.1776 | | CNL-FDA.chr6.26153 | | 0.028687 | | 0.056556 | | 0.507239 | | RNL-FNG.chr6.4114 | | RNL-FNG.chr6.4113 | | 0.148795 | | 0.288855 | | | 0.515122 |
| CN-FVI.CHR6.2188 | | CN-FNG.chr6.1541 | | 0.036354 | | 0.073045 | | 0.497686 | | RNL-FPE.chr0.2431 | | NL-FPE.chr2.4140 | | 0.013202 | | 0.014287 | | | 0.924028 |
| CN-FVI.CHR6.2188 | | CN-FDA.chr6.20969 | | 0.020089 | | 0.041634 | | 0.482505 | | RNL-FPE.chr5.2037 | | RNL-FPE.chr6.5624 | | 0.013623 | | 0.017763 | | | 0.766937 |
| CN-FVI.CHR6.2188 | | CN-FMA.chr6.1745 | | 0.015329 | | 0.032658 | | 0.469376 | | RNL-FvH4.1g15130.1 | | RN-FvH4.1g15140.1 | | 0.409838 | | 0.941587 | | | 0.435263 |
| CN-FVI.CHR6.2188 | | CN-FNU.ctg71.38 | | 0.020621 | | 0.052241 | | 0.394729 | | RNL-FvH4.6g50100.1 | | RNL-FvH4.6g50110.1 | | 0.219446 | | 0.4106 | | | 0.534451 |
| CN-FVI.CHR6.2188 | | CN-FII.chr6.1890 | | 0.006527 | | 0.024425 | | 0.267201 | | TN-FDA.chr3.05876 | | TNL-FDA.chr3.05837 | | 0.127317 | | 0.322723 | | | 0.394507 |
| CN-FVI.CHR6.2188 | | CN-FPE.chr0.885 | | 0.005087 | | 0.019121 | | 0.266054 | | TN-FDA.chr3.20720 | | TN-FDA.chr3.20717 | | 0.096487 | | 0.208924 | | | 0.46183 |
| CN-FVI.CHR6.2635 | | CN-FNU.ctg57.114 | | 0.046309 | | 0.072816 | | 0.635979 | | TN-FDA.chr3.20720 | | TN-FDA.chr3.20724 | | 0.026344 | | 0.057897 | | | 0.455018 |
| CN-FVI.CHR6.2635 | | CN-FPE.chr6.3007 | | 0.021851 | | 0.040249 | | 0.542884 | | TN-FDA.chr3.20720 | | TNL-FDA.chr3.20719 | | 0.049897 | | 0.112115 | | | 0.445051 |
| CN-FVI.CHR6.2635 | | CN-FNG.chr6.3269 | | 0.024143 | | 0.049973 | | 0.483126 | | TN-FDA.chr3.20724 | | TNL-FDA.chr3.20719 | | 0.037724 | | 0.097073 | | | 0.388614 |
| CN-FVI.CHR6.2635 | | CN-FII.chr6.3270.2 | | 0.029655 | | 0.064062 | | 0.462919 | | TN-FDA.chr3.20724 | | TN-FDA.chr3.20717 | | 0.079496 | | 0.2067 | | | 0.384596 |
| CN-FVI.CHR6.2635 | | CN-FDA.chr6.16615 | | 0.023241 | | 0.053984 | | 0.430522 | | TN-FDA.chr3.20745 | | NL-FDA.chr3.20838 | | 0.24402 | | 0.467425 | | | 0.522051 |
| CN-FVI.CHR7.3759 | | CNL-FII.chr3.954 | | 0.036069 | | 0.030246 | | 1.192512 | | TN-FDA.chr3.20745 | | N-FDA.chr3.20742 | | 0.063269 | | 0.177804 | | | 0.355836 |
| orthologous genes | | | | | | paralogous gene | | | | | | | | | | | | | |
| Seq_ 1 | | Seq_2 | | Ka | | Ks | | Ka/Ks | | Seq_ 1 | | Seq_2 | | Ka | | Ks | | | Ka/Ks |
| CN-FVI.CHR7.3759 | CNL-FvH4.3g11490.1 | | 0.041427 | | 0.038144 | | 1.086076 | | TN-FDA.chr6.02329 | | TNL-FDA.chr6.02345 | | 0.072004 | | 0.145913 | | 0.493475 | | |
| CNL-FDA.chr1.06545 | TNL-FNU.ctg162.30 | | 0.051724 | | 0.041955 | | 1.232855 | | TN-FDA.chr6.02329 | | TNL-FDA.chr6.02361 | | 0.080296 | | 0.185811 | | 0.43214 | | |
| CNL-FDA.chr1.22189 | RNL-FvH4.1g15220.1 | | 0.031601 | | 0.05892 | | 0.536327 | | TN-FDA.chr6.02460 | | TNL-FDA.chr6.02345 | | 0.031426 | | 0.032762 | | 0.959227 | | |
| CNL-FDA.chr1.22189 | NL-FII.chr1.1268 | | 0.019535 | | 0.055663 | | 0.350955 | | TN-FDA.chr6.02460 | | TN-FDA.chr6.02329 | | 0.04103 | | 0.079394 | | 0.516795 | | |
| CNL-FDA.chr1.22189 | RNL-FMA.chr1.1806 | | 0.14679 | | 0.44549 | | 0.329502 | | TN-FDA.chr6.02460 | | TNL-FDA.chr6.02361 | | 0.02687 | | 0.092469 | | 0.290581 | | |
| CNL-FDA.chr2.12098 | TNL-FII.chr5.302.2 | | 0.131547 | | 0.170947 | | 0.76952 | | TN-FDA.chr6.26557 | | TNL-FDA.chr1.15663 | | 0.326139 | | 0.578177 | | 0.564081 | | |
| CNL-FDA.chr2.12098 | TNL-FMA.chr5.364 | | 0.179714 | | 0.247335 | | 0.7266 | | TN-FDA.chr6.26557 | | TNL-FDA.chr3.09399 | | 0.228428 | | 0.436367 | | 0.523477 | | |
| CNL-FDA.chr2.12209 | CNL-FvH4.2g36810.1 | | 4.209769 | | 2.153276 | | 1.955053 | | TN-FDA.chr6.26557 | | TNL-FDA.chr4.10670 | | 0.362267 | | 0.699217 | | 0.518104 | | |
| CNL-FDA.chr2.17966 | CNL-FMA.chr2.328 | | 0.025135 | | 0.053996 | | 0.465488 | | TN-FDA.chr7.00118 | | TNL-FDA.chr7.00110 | | 0.039255 | | 0.072808 | | 0.539163 | | |
| CNL-FDA.chr2.17966 | CNL-FNU.ctg104.183 | | 0.011477 | | 0.027855 | | 0.412028 | | TN-FDA.chr7.00118 | | TNL-FDA.chr7.00189 | | 0.064114 | | 0.160017 | | 0.400671 | | |
| CNL-FDA.chr2.19060 | CNL-FNU.ctg104.299 | | 0.009277 | | 0.010029 | | 0.924993 | | TN-FDA.chr7.00118 | | TN-FDA.chr7.00120 | | 0.081586 | | 0.205397 | | 0.397211 | | |
| CNL-FDA.chr2.19060 | CNL-FNG.chr2.482 | | 0.010399 | | 0.011923 | | 0.872143 | | TN-FDA.chr7.00118 | | TNL-FDA.chr7.00190 | | 0.07046 | | 0.179728 | | 0.392035 | | |
| CNL-FDA.chr2.19060 | CN-FII.chr2.551 | | 0.016145 | | 0.036084 | | 0.447424 | | TN-FDA.chr7.00120 | | TNL-FDA.chr7.00159 | | 0.081779 | | 0.132965 | | 0.615043 | | |
| CNL-FDA.chr2.19060 | CN-FPE.chr7.728 | | 0.042033 | | 0.099655 | | 0.421783 | | TN-FDA.chr7.00120 | | TNL-FDA.chr7.00189 | | 0.05648 | | 0.096149 | | 0.587421 | | |
| CNL-FDA.chr2.19060 | CN-FMA.chr2.503 | | 0.005903 | | 0.040291 | | 0.14652 | | TN-FDA.chr7.00120 | | TNL-FDA.chr7.00190 | | 0.061594 | | 0.109651 | | 0.561722 | | |
| CNL-FDA.chr3.05890 | TNL-FvH4.3g43610.1 | | 0.068704 | | 0.138848 | | 0.494811 | | TN-FDA.chr7.00120 | | TN-FDA.chr7.00192 | | 0.038989 | | 0.075517 | | 0.516289 | | |
| CNL-FDA.chr3.09595 | CNL-FNG.chr0.411 | | 0.005648 | | 0.018668 | | 0.302556 | | TN-FDA.chr7.00191 | | TNL-FDA.chr7.00189 | | 0.047351 | | 0.085915 | | 0.551139 | | |
| CNL-FDA.chr3.15906 | CNL-FvH4.3g11490.1 | | 0.059973 | | 0.116084 | | 0.516635 | | TN-FDA.chr7.00191 | | TN-FDA.chr7.00120 | | 0.054466 | | 0.100022 | | 0.544541 | | |
| CNL-FDA.chr3.15906 | CNL-FII.chr3.953 | | 0.056819 | | 0.123312 | | 0.460772 | | TN-FDA.chr7.00191 | | TNL-FDA.chr7.00190 | | 0.051254 | | 0.098502 | | 0.520335 | | |
| CNL-FDA.chr3.16968 | CNL-FII.chr3.408 | | 0.062398 | | 0.066199 | | 0.942581 | | TN-FDA.chr7.00191 | | TN-FDA.chr7.00192 | | 0.010596 | | 0.020377 | | 0.520006 | | |
| CNL-FDA.chr3.16968 | CNL-FPE.chr3.535 | | 0.031651 | | 0.03694 | | 0.856832 | | TN-FDA.chr7.00191 | | TNL-FDA.chr7.00159 | | 0.050487 | | 0.111424 | | 0.453103 | | |
| CNL-FDA.chr3.16978 | CNL-FPE.chr3.535 | | 0.050422 | | 0.058151 | | 0.867086 | | TN-FDA.chr7.00192 | | TNL-FDA.chr7.00190 | | 0.073714 | | 0.105301 | | 0.700027 | | |
| CNL-FDA.chr3.16978 | CNL-FII.chr3.408 | | 0.057826 | | 0.073151 | | 0.790496 | | TN-FDA.chr7.00192 | | TNL-FDA.chr7.00189 | | 0.074767 | | 0.110829 | | 0.674615 | | |
| CNL-FDA.chr3.23062 | CNL-FNU.ctg47.220 | | 0.020382 | | 0.014218 | | 1.433517 | | TN-FDA.chr7.00192 | | TNL-FDA.chr7.00159 | | 0.073452 | | 0.109153 | | 0.672925 | | |
| CNL-FDA.chr3.23062 | CNL-FNG.chr3.84 | | 0.0214 | | 0.023209 | | 0.922039 | | TN-FDA.chr7.02893 | | TNL-FDA.chr7.02890 | | 0.086107 | | 0.13837 | | 0.622294 | | |
| CNL-FDA.chr3.23062 | CNL-FMA.chr3.75 | | 0.020636 | | 0.024145 | | 0.854668 | | TN-FDA.chr7.02893 | | TNL-FDA.chr7.02892 | | 0.234486 | | 0.522635 | | 0.448661 | | |
| CNL-FDA.chr3.23062 | CNL-FII.chr3.77 | | 0.02432 | | 0.037173 | | 0.654233 | | TN-FII.chr5.2853 | | TNL-FII.chr5.2851 | | 0.14263 | | 0.440308 | | 0.323933 | | |
| CNL-FDA.chr4.11333 | CNL-FMA.chr4.679 | | 0.079725 | | 0.137258 | | 0.580839 | | TN-FNG.chr3.1991 | | TNL-FNG.chr5.112 | | 0.108135 | | 0.234449 | | 0.461231 | | |
| CNL-FDA.chr4.11333 | CNL-FvH4.4g06030.1 | | 0.072738 | | 0.135246 | | 0.537821 | | TN-FNG.chr3.1991 | | TNL-FNG.chr3.3322 | | 0.108061 | | 0.247438 | | 0.43672 | | |
| CNL-FDA.chr4.11333 | CNL-FII.chr4.377 | | 0.038704 | | 0.076457 | | 0.506212 | | TN-FNG.chr3.2354 | | TNL-FNG.chr3.2352 | | 0.12481 | | 0.288319 | | 0.43289 | | |
| CNL-FDA.chr4.24588 | NL-FNU.ctg84.211 | | 0.129618 | | 0.192793 | | 0.672317 | | TN-FPE.chr4.1012 | | TNL-FPE.chr6.1904 | | 0.184839 | | 0.267896 | | 0.689964 | | |
| CNL-FDA.chr4.24588 | NL-FMA.chr4.1335 | | 0.094871 | | 0.146182 | | 0.64899 | | TN-FPE.chr5.245 | | TNL-FPE.chr5.1409 | | 0.034665 | | 0.067303 | | 0.515068 | | |
| CNL-FDA.chr4.24588 | CNL-FvH4.4g16700.1 | | 0.082695 | | 0.1287 | | 0.642541 | | TN-FvH4.1g22520.1 | | TNL-FvH4.1g22370.1 | | 0.051009 | | 0.062607 | | 0.814748 | | |
| CNL-FDA.chr4.24588 | NL-FPE.chr3.4014 | | 0.111513 | | 0.1757 | | 0.634677 | | TN-FvH4.3g45630.1 | | TNL-FvH4.3g44570.1 | | 3.149107 | | 2.196507 | | 1.433688 | | |
| CNL-FDA.chr4.24588 | CNL-FII.chr4.1300 | | 0.082711 | | 0.137561 | | 0.601263 | | TN-FvH4.5g34230.1 | | TN-FvH4.5g34240.1 | | 0.244193 | | 0.707315 | | 0.345239 | | |
| CNL-FDA.chr4.24588 | NL-FNG.chr4.1397 | | 0.011397 | | 0.021278 | | 0.535617 | | TN-FvH4.5g34240.1 | | TNL-FvH4.3g45380.1 | | 0.100162 | | 0.219793 | | 0.455711 | | |
| CNL-FDA.chr5.07881 | CNL-FNG.chr5.1760 | | 0.065208 | | 0.059779 | | 1.090832 | | TN-FvH4.7g32720.1 | | TNL-FvH4.7g32440.1 | | 0.028076 | | 0.035041 | | 0.801243 | | |
| CNL-FDA.chr5.07881 | CNL-FMA.chr5.1739 | | 0.06562 | | 0.075696 | | 0.866892 | | TN-FvH4.7g32720.1 | | TNL-FvH4.7g32460.1 | | 0.099977 | | 0.193217 | | 0.517434 | | |
| CNL-FDA.chr5.22369 | CNL-FMA.chr5.2388 | | 0.027123 | | 0.039496 | | 0.686748 | | TNL-FDA.chr1.07161 | | TNL-FDA.chr1.04275 | | 0.096233 | | 0.150732 | | 0.638438 | | |
| CNL-FDA.chr5.22369 | CN-FNG.chr5.2692 | | 0.00479 | | 0.01317 | | 0.363669 | | TNL-FDA.chr1.15663 | | TNL-FDA.chr4.10670 | | 0.203427 | | 0.319173 | | 0.637358 | | |
| CNL-FDA.chr5.24059 | TNL-FNG.chr3.3322 | | 0.020281 | | 0.042204 | | 0.480551 | | TNL-FDA.chr1.17978 | | TNL-FDA.chr1.17976 | | 0.083237 | | 0.173269 | | 0.480389 | | |
| CNL-FDA.chr5.24089 | CNL-FvH4.4g06030.1 | | 0.055268 | | 0.089864 | | 0.61501 | | TNL-FDA.chr1.17980 | | TNL-FDA.chr1.17981 | | 0.156728 | | 0.449423 | | 0.348732 | | |
| orthologous genes | | | | | | paralogous gene | | | | | | | | | | | | | |
| Seq_ 1 | | Seq_2 | | Ka | | Ks | | Ka/Ks | | Seq_ 1 | | Seq_2 | | Ka | | Ks | | Ka/Ks | |
| CNL-FDA.chr5.24089 | CNL-FII.chr4.377 | | 0.050633 | | 0.086162 | | 0.587651 | | TNL-FDA.chr2.20071 | | TNL-FDA.chr2.20072 | | 0.376073 | | 0.713515 | | 0.52707 | | |
| CNL-FDA.chr5.24311 | CNL-FNU.ctg150.57 | | 0.065048 | | 0.07914 | | 0.821932 | | TNL-FDA.chr3.05639 | | TNL-FDA.chr6.02439 | | 0.14766 | | 0.329473 | | 0.448169 | | |
| CNL-FDA.chr5.24966 | CNL-FNG.chr5.2227 | | 0.029344 | | 0.069971 | | 0.419375 | | TNL-FDA.chr3.05837 | | TNL-FDA.chr3.05839 | | 0.105375 | | 0.138111 | | 0.76297 | | |
| CNL-FDA.chr5.24966 | CNL-FII.chr5.2008 | | 0.098619 | | 0.253897 | | 0.388423 | | TNL-FDA.chr3.05837 | | TNL-FDA.chr3.05838 | | 0.12039 | | 0.201131 | | 0.598567 | | |
| CNL-FDA.chr5.24966 | CNL-FvH4.5g23470.1 | | 0.097783 | | 0.268749 | | 0.363845 | | TNL-FDA.chr3.05838 | | TNL-FDA.chr3.05837 | | 0.12039 | | 0.201131 | | 0.598567 | | |
| CNL-FDA.chr5.24966 | CNL-FNU.ctg158.148 | | 4.57E-04 | | 0.003138 | | 0.145532 | | TNL-FDA.chr3.05839 | | TNL-FDA.chr3.05838 | | 0.078476 | | 0.115167 | | 0.681413 | | |
| CNL-FDA.chr5.25044 | CNL-FII.chr5.2008 | | 0.013363 | | 0.028019 | | 0.476943 | | TNL-FDA.chr3.09399 | | TNL-FDA.chr1.15663 | | 0.086151 | | 0.115145 | | 0.748194 | | |
| CNL-FDA.chr5.25044 | CNL-FNG.chr5.2150 | | 0.005965 | | 0.013848 | | 0.430733 | | TNL-FDA.chr3.09474 | | TNL-FDA.chr5.23994 | | 0.188281 | | 0.315474 | | 0.596821 | | |
| CNL-FDA.chr5.25044 | CNL-FvH4.5g23470.1 | | 0.012892 | | 0.036052 | | 0.357595 | | TNL-FDA.chr3.09523 | | TNL-FDA.chr4.10670 | | 0.148013 | | 0.278308 | | 0.53183 | | |
| CNL-FDA.chr5.25044 | CNL-FNU.ctg158.48 | | 0.010579 | | 0.035811 | | 0.29542 | | TNL-FDA.chr3.09523 | | TNL-FDA.chr1.15663 | | 0.191757 | | 0.364639 | | 0.525883 | | |
| CNL-FDA.chr5.25047 | CNL-FII.chr5.2006 | | 0.018611 | | 0.031129 | | 0.597859 | | TNL-FDA.chr3.20670 | | TNL-FDA.chr3.20678 | | 0.160565 | | 0.327935 | | 0.489624 | | |
| CNL-FDA.chr5.25047 | CNL-FNU.ctg158.45 | | 0.013498 | | 0.028278 | | 0.47732 | | TNL-FDA.chr3.20670 | | TNL-FDA.chr3.20669 | | 0.343901 | | 0.722597 | | 0.475923 | | |
| CNL-FDA.chr5.25047 | CNL-FvH4.5g23450.1 | | 0.023737 | | 0.053265 | | 0.445632 | | TNL-FDA.chr3.20678 | | TNL-FDA.chr3.20677 | | 0.322478 | | 0.707383 | | 0.455875 | | |
| CNL-FDA.chr5.25047 | CNL-FNG.chr5.2149 | | 0.006901 | | 0.018392 | | 0.375243 | | TNL-FDA.chr3.20725 | | TN-FDA.chr3.20724 | | 0.264798 | | 0.896804 | | 0.295268 | | |
| CNL-FDA.chr5.25049 | CNL-FII.chr5.2004 | | 0.007743 | | 0.031169 | | 0.248414 | | TNL-FDA.chr3.20747 | | NL-FDA.chr3.20746 | | 0.092347 | | 0.106752 | | 0.865058 | | |
| CNL-FDA.chr5.25049 | CNL-FNG.chr5.2147 | | 0.006824 | | 0.037625 | | 0.181378 | | TNL-FDA.chr5.03487 | | TN-FDA.chr5.03486 | | 0.243851 | | 0.623966 | | 0.390808 | | |
| CNL-FDA.chr5.25049 | CNL-FNU.ctg158.43 | | 0.001813 | | 0.018594 | | 0.097523 | | TNL-FDA.chr5.03488 | | TNL-FDA.chr5.03487 | | 0.186364 | | 0.48982 | | 0.380474 | | |
| CNL-FDA.chr5.25050 | CNL-FII.chr5.3063 | | 0.002728 | | 0.010807 | | 0.252414 | | TNL-FDA.chr5.25282 | | TNL-FDA.chr5.25283 | | 0.271453 | | 0.601097 | | 0.451595 | | |
| CNL-FDA.chr5.25050 | CNL-FNU.ctg158.42 | | 0.002728 | | 0.01549 | | 0.176082 | | TNL-FDA.chr6.02361 | | NL-FDA.chr6.02338 | | 0.077274 | | 0.087982 | | 0.8783 | | |
| CNL-FDA.chr5.25050 | CNL-FNG.chr5.2146 | | 0.001363 | | 0.013927 | | 0.097836 | | TNL-FDA.chr6.02361 | | TNL-FDA.chr6.02345 | | 0.130368 | | 0.224309 | | 0.5812 | | |
| CNL-FDA.chr5.25050 | CNL-FvH4.5g23420.1 | | 0.001818 | | 0.034471 | | 0.052738 | | TNL-FDA.chr7.00110 | | TNL-FDA.chr7.00159 | | 0.080387 | | 0.176269 | | 0.456049 | | |
| CNL-FDA.chr6.02198 | CN-FII.chr6.198 | | 0.079392 | | 0.109748 | | 0.723405 | | TNL-FDA.chr7.00110 | | TNL-FDA.chr7.00190 | | 0.075643 | | 0.201875 | | 0.374702 | | |
| CNL-FDA.chr6.02198 | CNL-FNG.chr2.1430 | | 0.093143 | | 0.145259 | | 0.641219 | | TNL-FDA.chr7.00110 | | TNL-FDA.chr7.00189 | | 0.072468 | | 0.212217 | | 0.341481 | | |
| CNL-FDA.chr6.02459 | TNL-FvH4.6g05610.1 | | 0.069079 | | 0.103937 | | 0.664625 | | TNL-FDA.chr7.00114 | | TNL-FDA.chr7.00159 | | 0.107034 | | 0.179666 | | 0.595736 | | |
| CNL-FDA.chr6.13552 | CNL-FNG.chr5.655 | | 0.109702 | | 0.150308 | | 0.729851 | | TNL-FDA.chr7.00114 | | TNL-FDA.chr7.00190 | | 0.102473 | | 0.19622 | | 0.522236 | | |
| CNL-FDA.chr6.13552 | CN-FNU.ctg53.150 | | 0.103268 | | 0.1757 | | 0.587749 | | TNL-FDA.chr7.00114 | | TNL-FDA.chr7.00184 | | 0.058361 | | 0.115462 | | 0.505459 | | |
| CNL-FDA.chr6.13552 | CNL-FvH4.6g48220.1 | | 0.060148 | | 0.107156 | | 0.561313 | | TNL-FDA.chr7.00114 | | TNL-FDA.chr7.00189 | | 0.075 | | 0.178362 | | 0.42049 | | |
| CNL-FDA.chr6.13552 | CN-FMA.chr6.4269 | | 0.062621 | | 0.148897 | | 0.420565 | | TNL-FDA.chr7.00184 | | TNL-FDA.chr7.00159 | | 0.099683 | | 0.152208 | | 0.654911 | | |
| CNL-FDA.chr6.13552 | CNL-FII.chr6.4055 | | 0.069846 | | 0.168806 | | 0.413762 | | TNL-FDA.chr7.00184 | | TNL-FDA.chr7.00190 | | 0.108762 | | 0.17315 | | 0.628137 | | |
| CNL-FDA.chr6.13561 | CNL-FNU.ctg53.139 | | 0.01646 | | 0.028718 | | 0.573173 | | TNL-FDA.chr7.00189 | | TNL-FDA.chr7.00159 | | 0.076558 | | 0.100507 | | 0.761715 | | |
| CNL-FDA.chr6.13872 | CNL-FvH4.6g51140.1 | | 0.008593 | | 0.030193 | | 0.28461 | | TNL-FDA.chr7.00189 | | TNL-FDA.chr7.00190 | | 0.071723 | | 0.0966 | | 0.742472 | | |
| CNL-FDA.chr6.13967 | CNL-FvH4.6g52150.1 | | 0.027324 | | 0.018046 | | 1.514154 | | TNL-FDA.chr7.00189 | | TNL-FDA.chr7.00184 | | 0.08884 | | 0.154595 | | 0.574664 | | |
| CNL-FDA.chr6.14104 | CNL-FvH4.6g53580.1 | | 2.949368 | | 3.13079 | | 0.942052 | | TNL-FDA.chr7.00190 | | TNL-FDA.chr7.00159 | | 0.0884 | | 0.105064 | | 0.841391 | | |
| CNL-FDA.chr6.14104 | CNL-FPE.chr3.535 | | 0.114767 | | 0.254364 | | 0.451193 | | TNL-FDA.chr7.00190 | | TN-FDA.chr7.00191 | | 0.051254 | | 0.098502 | | 0.520335 | | |
| CNL-FDA.chr6.14104 | CNL-FII.chr3.408 | | 0.113727 | | 0.263712 | | 0.431253 | | TNL-FII.chr1.1393 | | TNL-FII.chr1.1394 | | 0.216 | | 0.546858 | | 0.394984 | | |
| CNL-FDA.chr6.26337 | CNL-FMA.chr6.1658 | | 0.047065 | | 0.051044 | | 0.922053 | | TNL-FII.chr1.757 | | TNL-FII.chr3.3419 | | 0.260108 | | 0.416154 | | 0.625028 | | |
| CNL-FDA.chr7.00307 | CNL-FII.chr3.408 | | 0.175425 | | 0.385633 | | 0.454901 | | TNL-FII.chr3.1830 | | TNL-FII.chr3.1831 | | 0.095693 | | 0.240153 | | 0.398467 | | |
| CNL-FDA.chr7.00307 | CNL-FPE.chr3.535 | | 0.180394 | | 0.402926 | | 0.44771 | | TNL-FII.chr3.1830 | | TNL-FII.chr7.1056 | | 0.116231 | | 0.435366 | | 0.266974 | | |
| CNL-FDA.chr7.00809 | CNL-FvH4.7g26370.1 | | 0.057431 | | 0.092619 | | 0.620081 | | TNL-FII.chr3.3626 | | TNL-FII.chr3.3603 | | 0.133658 | | 0.300055 | | 0.445445 | | |
| CNL-FDA.chr7.01119 | CNL-FvH4.7g23500.1 | | 0.032521 | | 0.040738 | | 0.798295 | | TNL-FII.chr3.744.2 | | TNL-FII.chr3.3419 | | 0.210661 | | 0.37777 | | 0.557644 | | |
| CNL-FDA.chr7.01259 | CNL-FvH4.7g22030.1 | | 0.092154 | | 0.11498 | | 0.801477 | | TNL-FII.chr4.2875 | | TNL-FII.chr4.2876 | | 0.160275 | | 0.460095 | | 0.348352 | | |
| CNL-FDA.chr7.01259 | CNL-FNU.ctg129.61 | | 0.062573 | | 0.08319 | | 0.752164 | | TNL-FII.chr5.2969 | | TNL-FII.chr5.2970 | | 0.188821 | | 0.46894 | | 0.402655 | | |
| orthologous genes | | | | | | paralogous gene | | | | | | | | | | | | | |
| Seq_ 1 | | Seq_2 | | Ka | | Ks | | Ka/Ks | | Seq_ 1 | | Seq_2 | | Ka | | Ks | | Ka/Ks | |
| CNL-FDA.chr7.01379 | CNL-FvH4.7g20580.1 | | 0.080521 | | 0.093998 | | 0.856627 | | TNL-FII.chr5.2970 | | TN-FII.chr5.2971 | | 0.224414 | | 0.486932 | | 0.460874 | | |
| CNL-FDA.chr7.20574 | TNL-FII.chr7.790 | | 0.021422 | | 0.038605 | | 0.554919 | | TNL-FII.chr5.3370 | | TNL-FII.chr5.3371 | | 0.159403 | | 0.232135 | | 0.686683 | | |
| CNL-FII.chr1.1071 | CNL-FPE.chr6.1273 | | 0.011903 | | 0.03066 | | 0.388234 | | TNL-FII.chr7.936 | | TN-FII.chr7.937 | | 0.118886 | | 0.215279 | | 0.552238 | | |
| CNL-FII.chr1.1071 | NL-FNG.chr1.1121 | | 0.012454 | | 0.039114 | | 0.318398 | | TNL-FMA.chr0.553 | | TNL-FMA.chr0.554 | | 0.148143 | | 0.252593 | | 0.586489 | | |
| CNL-FII.chr1.1071 | N-FvH4.1g12710.1 | | 0.010245 | | 0.033191 | | 0.308678 | | TNL-FMA.chr0.554 | | TNL-FMA.chr0.565 | | 0.004809 | | 0.012434 | | 0.386806 | | |
| CNL-FII.chr1.1071 | CNL-FNU.ctg170.24 | | 0.006657 | | 0.025357 | | 0.262532 | | TNL-FMA.chr1.1780 | | TNL-FMA.chr5.2877 | | 0.087519 | | 0.113551 | | 0.770746 | | |
| CNL-FII.chr1.1071 | CNL-FMA.chr1.1369 | | 0.006146 | | 0.023478 | | 0.261763 | | TNL-FMA.chr2.1405 | | N-FMA.chr2.1406 | | 0.034565 | | 0.166483 | | 0.20762 | | |
| CNL-FII.chr3.1384 | CNL-FNG.chr3.1370 | | 0.019196 | | 0.017006 | | 1.12877 | | TNL-FMA.chr2.2280 | | TNL-FMA.chr2.2281 | | 0.413307 | | 0.781304 | | 0.528996 | | |
| CNL-FII.chr3.1384 | CNL-FNU.ctg30.47 | | 0.013845 | | 0.022367 | | 0.618995 | | TNL-FMA.chr4.2945 | | TNL-FMA.chr4.2946 | | 0.194524 | | 0.585475 | | 0.332249 | | |
| CNL-FII.chr3.1385 | CNL-FNU.ctg165.171 | | 0.037499 | | 0.024307 | | 1.542737 | | TNL-FNG.chr3.3322 | | TNL-FNG.chr5.112 | | 0.114676 | | 0.164467 | | 0.697256 | | |
| CNL-FII.chr3.1385 | CNL-FNG.chr1.545 | | 0.031262 | | 0.030881 | | 1.012355 | | TNL-FNG.chr4.271 | | TN-FNG.chr4.253 | | 0.186521 | | 0.406074 | | 0.459327 | | |
| CNL-FII.chr3.1385 | CNL-FPE.chr5.124 | | 0.022086 | | 0.02254 | | 0.979876 | | TNL-FNU.ctg162.30 | | NL-FNU.ctg163.12 | | 0.057379 | | 0.04745 | | 1.209263 | | |
| CNL-FII.chr3.1385 | CNL-FMA.chr3.1344 | | 0.033157 | | 0.036485 | | 0.908768 | | TNL-FNU.ctg172.194 | | TNL-FNU.ctg172.195 | | 0.265879 | | 0.74664 | | 0.356101 | | |
| CNL-FII.chr3.1385 | CN-FvH4.3g16720.1 | | 0.021483 | | 0.025644 | | 0.837737 | | TNL-FNU.ctg172.195 | | TNL-FNU.ctg172.196 | | 0.173787 | | 0.415029 | | 0.418733 | | |
| CNL-FII.chr3.408 | CNL-FPE.chr3.535 | | 0.056663 | | 0.068577 | | 0.826265 | | TNL-FNU.ctg173.410 | | TN-FNU.ctg173.411 | | 0.132938 | | 0.287096 | | 0.463043 | | |
| CNL-FII.chr3.77 | CNL-FNU.ctg47.220 | | 0.019716 | | 0.035136 | | 0.561124 | | TNL-FNU.ctg3.21 | | TNL-FNU.ctg3.22 | | 0.118991 | | 0.214468 | | 0.554818 | | |
| CNL-FII.chr3.77 | CNL-FNG.chr3.84 | | 0.012674 | | 0.02593 | | 0.488764 | | TNL-FNU.ctg3.21 | | TNL-FNU.ctg3.25 | | 0.130918 | | 0.31094 | | 0.421039 | | |
| CNL-FII.chr3.77 | CNL-FMA.chr3.75 | | 0.01117 | | 0.029596 | | 0.377408 | | TNL-FNU.ctg3.22 | | TNL-FNU.ctg3.21 | | 0.118991 | | 0.214468 | | 0.554818 | | |
| CNL-FII.chr3.951 | CNL-FvH4.3g11490.1 | | 0.01292 | | 0.028619 | | 0.45146 | | TNL-FNU.ctg3.75 | | TNL-FNU.ctg3.73 | | 0.41161 | | 0.776866 | | 0.529834 | | |
| CNL-FII.chr3.953 | CNL-FvH4.3g11490.1 | | 0.05487 | | 0.116593 | | 0.470612 | | TNL-FNU.ctg81.212 | | TNL-FNU.ctg81.211 | | 0.158849 | | 0.499771 | | 0.317844 | | |
| CNL-FII.chr3.954 | N-FPE.chr3.1895 | | 0.022719 | | 0.027234 | | 0.834196 | | TNL-FNU.ctg81.216 | | TNL-FNU.ctg81.211 | | 0.037008 | | 0.077344 | | 0.47849 | | |
| CNL-FII.chr3.954 | CNL-FvH4.3g11490.1 | | 0.045721 | | 0.063286 | | 0.72245 | | TNL-FNU.ctg81.216 | | TNL-FNU.ctg81.212 | | 0.137682 | | 0.415031 | | 0.331739 | | |
| CNL-FII.chr4.1300 | NL-FNG.chr4.1397 | | 0.018185 | | 0.03092 | | 0.588123 | | TNL-FPE.chr7.2368 | | NL-FPE.chr7.2369 | | 0.307358 | | 0.662926 | | 0.463639 | | |
| CNL-FII.chr4.1300 | NL-FNU.ctg84.211 | | 0.015307 | | 0.04657 | | 0.328701 | | TNL-FvH4.1g16600.1 | | TNL-FvH4.1g16610.1 | | 0.159908 | | 0.279731 | | 0.571649 | | |
| CNL-FII.chr4.1300 | NL-FMA.chr4.1335 | | 0.009234 | | 0.032368 | | 0.285286 | | TNL-FvH4.1g22370.1 | | TNL-FvH4.1g22380.1 | | 0.154803 | | 0.256637 | | 0.603196 | | |
| CNL-FII.chr4.1300 | CNL-FvH4.4g16700.1 | | 0.007786 | | 0.032017 | | 0.243168 | | TNL-FvH4.1g22380.1 | | TNL-FvH4.1g22390.1 | | 0.309302 | | 0.619198 | | 0.49952 | | |
| CNL-FII.chr4.1300 | NL-FPE.chr3.4014 | | 0.007775 | | 0.034958 | | 0.22241 | | TNL-FvH4.1g22390.1 | | TNL-FvH4.1g22540.1 | | 0.073348 | | 0.10879 | | 0.674215 | | |
| CNL-FII.chr4.1808 | CN-FPE.chr1.928 | | 0.018249 | | 0.025897 | | 0.704654 | | TNL-FvH4.3g43630.1 | | TNL-FvH4.3g43610.1 | | 2.49908 | | 2.382757 | | 1.048819 | | |
| CNL-FII.chr4.1808 | CNL-FvH4.4g22940.1 | | 0.174182 | | 0.258349 | | 0.67421 | | TNL-FvH4.3g43860.1 | | TNL-FvH4.3g44000.1 | | 3.297768 | | 2.38844 | | 1.38072 | | |
| CNL-FII.chr4.1808 | CNL-FNU.ctg82.648 | | 0.07131 | | 0.132979 | | 0.53625 | | TNL-FvH4.3g43860.1 | | TNL-FvH4.3g43940.1 | | 0.088019 | | 0.150923 | | 0.583206 | | |
| CNL-FII.chr4.1808 | CNL-FNG.chr4.1917 | | 0.086839 | | 0.239749 | | 0.362208 | | TNL-FvH4.3g43940.1 | | TNL-FvH4.3g44000.1 | | 2.934817 | | 2.710492 | | 1.082762 | | |
| CNL-FII.chr4.1849.2 | CN-FPE.chr6.878 | | 0.010763 | | 0.043347 | | 0.248296 | | TNL-FvH4.3g44070.1 | | TNL-FvH4.3g44000.1 | | 2.327835 | | 2.260947 | | 1.029584 | | |
| CNL-FII.chr4.2076 | CNL-FMA.chr4.2118 | | 0.066852 | | 0.127245 | | 0.525378 | | TNL-FvH4.3g45170.1 | | TNL-FvH4.3g44570.1 | | 0.141418 | | 0.257893 | | 0.54836 | | |
| CNL-FII.chr4.2076 | CNL-FNU.ctg82.313 | | 0.054915 | | 0.10753 | | 0.510696 | | TNL-FvH4.5g38870.1 | | TNL-FvH4.1g15650.1 | | 0.16201 | | 0.292932 | | 0.553065 | | |
| CNL-FII.chr4.2076 | CNL-FPE.chr3.1482 | | 0.050405 | | 0.113795 | | 0.442941 | | TNL-FvH4.7g32440.1 | | TNL-FvH4.7g32460.1 | | 0.07947 | | 0.162765 | | 0.48825 | | |
| CNL-FII.chr4.2124 | CNL-FvH4.4g26300.1 | | 0.029061 | | 0.028177 | | 1.03135 | | TNL-FvH4.7g33190.1 | | TNL-FvH4.7g32460.1 | | 0.075183 | | 0.186545 | | 0.403026 | | |
| CNL-FII.chr4.2124 | CNL-FMA.chr4.2163 | | 0.025038 | | 0.033604 | | 0.745078 | | TNL-FVI.CHR2.3162 | | NL-FVI.CHR2.3163 | | 0.456604 | | 0.854475 | | 0.534369 | | |
| CNL-FII.chr4.2124 | NL-FNU.ctg82.264 | | 0.013502 | | 0.019609 | | 0.688548 | | TNL-FVI.CHR4.3081 | | TNL-FVI.CHR4.3082 | | 0.14575 | | 0.424436 | | 0.343398 | | |
| CNL-FII.chr4.2124 | CNL-FNG.chr4.2225 | | 0.01368 | | 0.03224 | | 0.424319 | | TNL-FVI.CHR4.3082 | | TNL-FVI.CHR4.3083 | | 0.143735 | | 0.424497 | | 0.338601 | | |
| CNL-FII.chr4.377 | CNL-FvH4.4g06030.1 | | 0.043944 | | 0.092677 | | 0.474159 | |  | |  | |  | |  | |  | | |
| CNL-FII.chr5.2001 | CNL-FvH4.5g23390.1 | | 0.015182 | | 0.041487 | | 0.365953 | |  | |  | |  | |  | |  | | |
| CNL-FII.chr5.2001 | CNL-FNU.ctg158.42 | | 0.141065 | | 0.558072 | | 0.252772 | |  | |  | |  | |  | |  | | |
| orthologous genes | | | | | | paralogous gene | | | | | | | | | | | | | |
| Seq_ 1 | | Seq_2 | | Ka | | Ks | | Ka/Ks | | Seq_ 1 | | Seq_2 | | Ka | | Ks | | Ka/Ks | |
| CNL-FII.chr5.2001 | CNL-FNG.chr5.2146 | | 0.14048 | | 0.561504 | | 0.250185 | |  | |  | |  | |  | |  | | |
| CNL-FII.chr5.2001 | CNL-FDA.chr5.25050 | | 0.13872 | | 0.568531 | | 0.243998 | |  | |  | |  | |  | |  | | |
| CNL-FII.chr5.2002 | CNL-FNG.chr5.2145 | | 0.003181 | | 0.026227 | | 0.121277 | |  | |  | |  | |  | |  | | |
| CNL-FII.chr5.2002 | CNL-FPE.chr4.1619 | | 0.004318 | | 0.046186 | | 0.093491 | |  | |  | |  | |  | |  | | |
| CNL-FII.chr5.2002 | CNL-FNU.ctg158.40 | | 0.003863 | | 0.047778 | | 0.080851 | |  | |  | |  | |  | |  | | |
| CNL-FII.chr5.2003 | CNL-FDA.chr5.25050 | | 0.003183 | | 0.012367 | | 0.257398 | |  | |  | |  | |  | |  | | |
| CNL-FII.chr5.2003 | CNL-FNU.ctg158.42 | | 0.005008 | | 0.021774 | | 0.230011 | |  | |  | |  | |  | |  | | |
| CNL-FII.chr5.2003 | CNL-FNG.chr5.2146 | | 0.002728 | | 0.013925 | | 0.195883 | |  | |  | |  | |  | |  | | |
| CNL-FII.chr5.2003 | CNL-FvH4.5g23420.1 | | 0.004097 | | 0.034466 | | 0.11886 | |  | |  | |  | |  | |  | | |
| CNL-FII.chr5.2004 | CNL-FNU.ctg158.43 | | 0.007743 | | 0.029583 | | 0.26172 | |  | |  | |  | |  | |  | | |
| CNL-FII.chr5.2004 | CNL-FNG.chr5.2147 | | 0.007287 | | 0.027972 | | 0.260509 | |  | |  | |  | |  | |  | | |
| CNL-FII.chr5.2006 | CNL-FNU.ctg158.45 | | 0.018119 | | 0.028495 | | 0.635852 | |  | |  | |  | |  | |  | | |
| CNL-FII.chr5.2006 | CNL-FvH4.5g23450.1 | | 0.021918 | | 0.052058 | | 0.421035 | |  | |  | |  | |  | |  | | |
| CNL-FII.chr5.2006 | CNL-FNG.chr5.2149 | | 0.014377 | | 0.034359 | | 0.418446 | |  | |  | |  | |  | |  | | |
| CNL-FII.chr5.2007 | CNL-FNU.ctg158.46 | | 0.035866 | | 0.097045 | | 0.369581 | |  | |  | |  | |  | |  | | |
| CNL-FII.chr5.2008 | CNL-FNG.chr5.2150 | | 0.009194 | | 0.032754 | | 0.280693 | |  | |  | |  | |  | |  | | |
| CNL-FII.chr5.2008 | CNL-FvH4.5g23470.1 | | 0.009645 | | 0.039312 | | 0.245353 | |  | |  | |  | |  | |  | | |
| CNL-FII.chr5.2008 | CNL-FNU.ctg158.48 | | 0.009456 | | 0.041664 | | 0.226956 | |  | |  | |  | |  | |  | | |
| CNL-FII.chr5.3003 | CNL-FPE.chr1.2047 | | 0.060666 | | 0.208536 | | 0.290915 | |  | |  | |  | |  | |  | | |
| CNL-FII.chr5.3003 | CNL-FNG.chr5.2999 | | 0.038707 | | 0.157972 | | 0.245025 | |  | |  | |  | |  | |  | | |
| CNL-FII.chr5.3063 | CNL-FNG.chr5.2146 | | 0.003184 | | 0.012362 | | 0.257527 | |  | |  | |  | |  | |  | | |
| CNL-FII.chr5.3063 | CNL-FNU.ctg158.42 | | 0.004552 | | 0.020193 | | 0.225428 | |  | |  | |  | |  | |  | | |
| CNL-FII.chr5.3063 | CNL-FvH4.5g23420.1 | | 0.003641 | | 0.032856 | | 0.110805 | |  | |  | |  | |  | |  | | |
| CNL-FII.chr6.4055 | CNL-FNG.chr5.655 | | 0.112055 | | 0.173721 | | 0.645027 | |  | |  | |  | |  | |  | | |
| CNL-FII.chr6.4055 | CNL-FvH4.6g48220.1 | | 0.078617 | | 0.152292 | | 0.516227 | |  | |  | |  | |  | |  | | |
| CNL-FII.chr6.4055 | CN-FMA.chr6.4269 | | 0.086854 | | 0.216176 | | 0.401773 | |  | |  | |  | |  | |  | | |
| CNL-FII.chr6.4055 | CN-FNU.ctg53.150 | | 0.069514 | | 0.201872 | | 0.344344 | |  | |  | |  | |  | |  | | |
| CNL-FII.chr7.1027 | CN-FPE.chr6.3765 | | 0.012385 | | 0.029682 | | 0.417254 | |  | |  | |  | |  | |  | | |
| CNL-FII.chr7.1027 | CNL-FNG.chr7.951 | | 0.005942 | | 0.0267 | | 0.222553 | |  | |  | |  | |  | |  | | |
| CNL-FII.chr7.162.3 | CNL-FNU.ctg146.100 | | 0.019169 | | 0.035075 | | 0.546529 | |  | |  | |  | |  | |  | | |
| CNL-FII.chr7.162.3 | CNL-FvH4.7g01950.1 | | 0.026575 | | 0.054681 | | 0.485992 | |  | |  | |  | |  | |  | | |
| CNL-FII.chr7.257 | CNL-FPE.chr6.1639 | | 0.024886 | | 0.035837 | | 0.694415 | |  | |  | |  | |  | |  | | |
| CNL-FII.chr7.257 | CNL-FvH4.7g02890.1 | | 0.007377 | | 0.02782 | | 0.265157 | |  | |  | |  | |  | |  | | |
| CNL-FII.chr7.257 | CNL-FNG.chr7.204 | | 0.006878 | | 0.030328 | | 0.226786 | |  | |  | |  | |  | |  | | |
| CNL-FII.chr7.257 | CNL-FNU.ctg145.8 | | 0.006127 | | 0.029795 | | 0.205652 | |  | |  | |  | |  | |  | | |
| CNL-FII.chr7.55 | CNL-FPE.chr7.237 | | 0.018888 | | 0.069952 | | 0.270017 | |  | |  | |  | |  | |  | | |
| CNL-FII.chr7.55 | NL-FvH4.7g00850.1 | | 0.025648 | | 0.112861 | | 0.227255 | |  | |  | |  | |  | |  | | |
| CNL-FII.chr7.708 | CNL-FPE.chr0.563 | | 0.050783 | | 0.041446 | | 1.225291 | |  | |  | |  | |  | |  | | |
| CNL-FII.chr7.708 | NL-FNG.chr7.723 | | 0.057221 | | 0.098398 | | 0.581521 | |  | |  | |  | |  | |  | | |
| CNL-FII.chr7.998 | NL-FNU.ctg132.328 | | 0.028337 | | 0.040652 | | 0.697075 | |  | |  | |  | |  | |  | | |
| CNL-FII.chr7.998 | CNL-FMA.chr7.880 | | 0.022141 | | 0.040962 | | 0.540538 | |  | |  | |  | |  | |  | | |
| CNL-FMA.chr1.1369 | CNL-FPE.chr6.1273 | | 0.010219 | | 0.01876 | | 0.544734 | |  | |  | |  | |  | |  | | |
| orthologous genes | | | | | | paralogous gene | | | | | | | | | | | | | |
| Seq_ 1 | | Seq_2 | | Ka | | Ks | | Ka/Ks | | Seq_ 1 | | Seq_2 | | Ka | | Ks | | Ka/Ks | |
| CNL-FMA.chr1.1369 | N-FvH4.1g12710.1 | | 0.006505 | | 0.01309 | | 0.496951 | |  | |  | |  | |  | |  | | |
| CNL-FMA.chr1.1369 | NL-FNG.chr1.1121 | | 0.010983 | | 0.031079 | | 0.353372 | |  | |  | |  | |  | |  | | |
| CNL-FMA.chr1.1369 | CNL-FNU.ctg170.24 | | 0.00563 | | 0.018016 | | 0.312483 | |  | |  | |  | |  | |  | | |
| CNL-FMA.chr2.328 | CNL-FNU.ctg104.183 | | 0.027365 | | 0.043861 | | 0.623903 | |  | |  | |  | |  | |  | | |
| CNL-FMA.chr2.328 | CN-FII.chr2.469 | | 0.021392 | | 0.044499 | | 0.480729 | |  | |  | |  | |  | |  | | |
| CNL-FMA.chr3.1344 | CNL-FNU.ctg165.171 | | 0.050532 | | 0.041384 | | 1.221063 | |  | |  | |  | |  | |  | | |
| CNL-FMA.chr3.1344 | CN-FvH4.3g16720.1 | | 0.014037 | | 0.012241 | | 1.146708 | |  | |  | |  | |  | |  | | |
| CNL-FMA.chr3.1344 | CNL-FNG.chr1.545 | | 0.054971 | | 0.057248 | | 0.960215 | |  | |  | |  | |  | |  | | |
| CNL-FMA.chr3.1344 | CNL-FPE.chr5.124 | | 0.027313 | | 0.033855 | | 0.806742 | |  | |  | |  | |  | |  | | |
| CNL-FMA.chr3.2744 | CNL-FPE.chr7.1015 | | 0.060498 | | 0.11499 | | 0.526111 | |  | |  | |  | |  | |  | | |
| CNL-FMA.chr3.2975 | CNL-FPE.chr1.1624 | | 0.014657 | | 0.021066 | | 0.695793 | |  | |  | |  | |  | |  | | |
| CNL-FMA.chr3.2975 | CNL-FNG.chr3.2531 | | 0.031839 | | 0.060549 | | 0.525848 | |  | |  | |  | |  | |  | | |
| CNL-FMA.chr3.2975 | CN-FvH4.3g21750.1 | | 0.003605 | | 0.020756 | | 0.173662 | |  | |  | |  | |  | |  | | |
| CNL-FMA.chr3.75 | CNL-FNG.chr3.84 | | 0.010608 | | 0.013232 | | 0.801697 | |  | |  | |  | |  | |  | | |
| CNL-FMA.chr3.75 | CNL-FNU.ctg47.220 | | 0.01759 | | 0.025834 | | 0.680872 | |  | |  | |  | |  | |  | | |
| CNL-FMA.chr4.2118 | CNL-FNU.ctg82.313 | | 0.069273 | | 0.097613 | | 0.709674 | |  | |  | |  | |  | |  | | |
| CNL-FMA.chr4.2118 | CNL-FPE.chr3.1482 | | 0.02632 | | 0.045874 | | 0.573752 | |  | |  | |  | |  | |  | | |
| CNL-FMA.chr4.2163 | CNL-FvH4.4g26300.1 | | 0.005919 | | 0.003031 | | 1.9528 | |  | |  | |  | |  | |  | | |
| CNL-FMA.chr4.2163 | NL-FNU.ctg82.264 | | 0.031937 | | 0.044028 | | 0.725377 | |  | |  | |  | |  | |  | | |
| CNL-FMA.chr4.2163 | CNL-FNG.chr4.2225 | | 0.025016 | | 0.040147 | | 0.623113 | |  | |  | |  | |  | |  | | |
| CNL-FMA.chr5.1324 | CNL-FNU.ctg147.19 | | 0.018428 | | 0.034821 | | 0.529214 | |  | |  | |  | |  | |  | | |
| CNL-FMA.chr5.1739 | CNL-FNG.chr5.1760 | | 0.071446 | | 0.052036 | | 1.373031 | |  | |  | |  | |  | |  | | |
| CNL-FMA.chr5.2388 | CN-FNG.chr5.2692 | | 0.020062 | | 0.020927 | | 0.958629 | |  | |  | |  | |  | |  | | |
| CNL-FMA.chr6.1184 | CNL-FNU.ctg78.2 | | 0.027129 | | 0.036604 | | 0.741152 | |  | |  | |  | |  | |  | | |
| CNL-FMA.chr6.2166 | CNL-FNU.ctg64.14.1 | | 0.023121 | | 0.03425 | | 0.675066 | |  | |  | |  | |  | |  | | |
| CNL-FMA.chr6.2723 | CN-FPE.chr6.453 | | 0.028679 | | 0.046732 | | 0.613696 | |  | |  | |  | |  | |  | | |
| CNL-FMA.chr6.2723 | CN-FNU.ctg65.11 | | 0.026735 | | 0.059062 | | 0.45266 | |  | |  | |  | |  | |  | | |
| CNL-FMA.chr6.2723 | CNL-FNG.chr6.2058 | | 0.015323 | | 0.038781 | | 0.395115 | |  | |  | |  | |  | |  | | |
| CNL-FMA.chr6.3395 | CNL-FPE.chr2.397 | | 0.021952 | | 0.007159 | | 3.066599 | |  | |  | |  | |  | |  | | |
| CNL-FMA.chr7.880 | NL-FNU.ctg132.328 | | 0.005559 | | 0.019403 | | 0.286527 | |  | |  | |  | |  | |  | | |
| CNL-FNG.chr1.545 | CNL-FPE.chr5.124 | | 0.040861 | | 0.032541 | | 1.255679 | |  | |  | |  | |  | |  | | |
| CNL-FNG.chr1.545 | CN-FvH4.3g16720.1 | | 0.036328 | | 0.029712 | | 1.222667 | |  | |  | |  | |  | |  | | |
| CNL-FNG.chr1.545 | CNL-FNU.ctg165.171 | | 0.040669 | | 0.038139 | | 1.066338 | |  | |  | |  | |  | |  | | |
| CNL-FNG.chr2.1430 | CNL-FvH4.6g02820.1 | | 0.075844 | | 0.084746 | | 0.894959 | |  | |  | |  | |  | |  | | |
| CNL-FNG.chr2.416 | CN-FvH4.2g05600.1 | | 0.103363 | | 0.15089 | | 0.685024 | |  | |  | |  | |  | |  | | |
| CNL-FNG.chr2.416 | CNL-FPE.chr7.1798 | | 0.048665 | | 0.087063 | | 0.558957 | |  | |  | |  | |  | |  | | |
| CNL-FNG.chr2.482 | CNL-FNU.ctg104.299 | | 0.006625 | | 0.010134 | | 0.653775 | |  | |  | |  | |  | |  | | |
| CNL-FNG.chr2.482 | CN-FPE.chr7.728 | | 0.042901 | | 0.099505 | | 0.431148 | |  | |  | |  | |  | |  | | |
| CNL-FNG.chr3.1370 | CNL-FNU.ctg30.47 | | 0.010347 | | 0.011342 | | 0.912232 | |  | |  | |  | |  | |  | | |
| CNL-FNG.chr3.1371 | CNL-FNU.ctg165.171 | | 0.052959 | | 0.035836 | | 1.477825 | |  | |  | |  | |  | |  | | |
| CNL-FNG.chr3.1371 | CN-FvH4.3g16720.1 | | 0.020049 | | 0.01469 | | 1.364861 | |  | |  | |  | |  | |  | | |
| CNL-FNG.chr3.1371 | NL-FDA.chr3.21153 | | 0.025737 | | 0.024103 | | 1.067787 | |  | |  | |  | |  | |  | | |
| orthologous genes | | | | | | paralogous gene | | | | | | | | | | | | | |
| Seq_ 1 | | Seq_2 | | Ka | | Ks | | Ka/Ks | | Seq_ 1 | | Seq_2 | | Ka | | Ks | | Ka/Ks | |
| CNL-FNG.chr3.1371 | CNL-FII.chr3.1385 | | 0.031941 | | 0.037464 | | 0.852576 | |  | |  | |  | |  | |  | | |
| CNL-FNG.chr3.1371 | CNL-FMA.chr3.1344 | | 0.027713 | | 0.035181 | | 0.787725 | |  | |  | |  | |  | |  | | |
| CNL-FNG.chr3.1371 | CNL-FPE.chr5.124 | | 0.015688 | | 0.023238 | | 0.675103 | |  | |  | |  | |  | |  | | |
| CNL-FNG.chr3.2531 | CNL-FPE.chr1.1624 | | 0.03864 | | 0.051922 | | 0.744186 | |  | |  | |  | |  | |  | | |
| CNL-FNG.chr3.84 | CNL-FNU.ctg47.220 | | 0.016844 | | 0.024899 | | 0.676485 | |  | |  | |  | |  | |  | | |
| CNL-FNG.chr4.1917 | CNL-FvH4.4g22940.1 | | 0.088815 | | 0.224194 | | 0.396151 | |  | |  | |  | |  | |  | | |
| CNL-FNG.chr4.1917 | CN-FPE.chr1.928 | | 0.066426 | | 0.177467 | | 0.374303 | |  | |  | |  | |  | |  | | |
| CNL-FNG.chr4.1917 | CNL-FNU.ctg82.648 | | 0.084037 | | 0.225636 | | 0.372444 | |  | |  | |  | |  | |  | | |
| CNL-FNG.chr4.2225 | CNL-FvH4.4g26300.1 | | 0.028461 | | 0.034757 | | 0.818847 | |  | |  | |  | |  | |  | | |
| CNL-FNG.chr4.2225 | NL-FNU.ctg82.264 | | 0.007175 | | 0.039657 | | 0.180926 | |  | |  | |  | |  | |  | | |
| CNL-FNG.chr5.2089 | CNL-FvH4.5g22710.1 | | 0.005531 | | 0.01724 | | 0.320828 | |  | |  | |  | |  | |  | | |
| CNL-FNG.chr5.2089 | CNL-FII.chr5.3063 | | 0.105476 | | 0.689984 | | 0.152867 | |  | |  | |  | |  | |  | | |
| CNL-FNG.chr5.2089 | CNL-FNU.ctg158.42 | | 0.105731 | | 0.696138 | | 0.151883 | |  | |  | |  | |  | |  | | |
| CNL-FNG.chr5.2089 | CNL-FDA.chr5.25050 | | 0.103613 | | 0.688479 | | 0.150495 | |  | |  | |  | |  | |  | | |
| CNL-FNG.chr5.2145 | CNL-FPE.chr4.1619 | | 0.004092 | | 0.035693 | | 0.114656 | |  | |  | |  | |  | |  | | |
| CNL-FNG.chr5.2145 | CNL-FNU.ctg158.40 | | 0.003637 | | 0.037265 | | 0.097603 | |  | |  | |  | |  | |  | | |
| CNL-FNG.chr5.2146 | CNL-FNU.ctg158.42 | | 0.004095 | | 0.023354 | | 0.175354 | |  | |  | |  | |  | |  | | |
| CNL-FNG.chr5.2146 | CNL-FvH4.5g23420.1 | | 0.003184 | | 0.036071 | | 0.08828 | |  | |  | |  | |  | |  | | |
| CNL-FNG.chr5.2147 | CNL-FNU.ctg158.43 | | 0.006824 | | 0.036024 | | 0.189433 | |  | |  | |  | |  | |  | | |
| CNL-FNG.chr5.2149 | CNL-FNU.ctg158.45 | | 0.011478 | | 0.028317 | | 0.40533 | |  | |  | |  | |  | |  | | |
| CNL-FNG.chr5.2149 | CNL-FvH4.5g23450.1 | | 0.020897 | | 0.058228 | | 0.358877 | |  | |  | |  | |  | |  | | |
| CNL-FNG.chr5.2150 | CNL-FvH4.5g23470.1 | | 0.009652 | | 0.037616 | | 0.256585 | |  | |  | |  | |  | |  | | |
| CNL-FNG.chr5.2150 | CNL-FNU.ctg158.48 | | 0.007785 | | 0.039613 | | 0.196518 | |  | |  | |  | |  | |  | | |
| CNL-FNG.chr5.2227 | CNL-FNU.ctg158.148 | | 0.028863 | | 0.06999 | | 0.412389 | |  | |  | |  | |  | |  | | |
| CNL-FNG.chr5.2227 | CNL-FII.chr5.2008 | | 0.099695 | | 0.253942 | | 0.392589 | |  | |  | |  | |  | |  | | |
| CNL-FNG.chr5.2227 | CNL-FvH4.5g23470.1 | | 0.099642 | | 0.265406 | | 0.375431 | |  | |  | |  | |  | |  | | |
| CNL-FNG.chr5.2999 | CNL-FPE.chr1.2047 | | 0.01796 | | 0.056007 | | 0.32067 | |  | |  | |  | |  | |  | | |
| CNL-FNG.chr5.654 | CNL-FvH4.6g48220.1 | | 0.093964 | | 0.137101 | | 0.685364 | |  | |  | |  | |  | |  | | |
| CNL-FNG.chr5.654 | CNL-FDA.chr6.13552 | | 0.135066 | | 0.234536 | | 0.575884 | |  | |  | |  | |  | |  | | |
| CNL-FNG.chr5.654 | CNL-FII.chr6.4055 | | 0.116903 | | 0.209002 | | 0.559336 | |  | |  | |  | |  | |  | | |
| CNL-FNG.chr5.655 | CNL-FvH4.6g48220.1 | | 0.091993 | | 0.114254 | | 0.805159 | |  | |  | |  | |  | |  | | |
| CNL-FNG.chr5.655 | CN-FNU.ctg53.150 | | 0.08215 | | 0.146572 | | 0.560474 | |  | |  | |  | |  | |  | | |
| CNL-FNG.chr6.2058 | CN-FNU.ctg65.11 | | 0.01268 | | 0.022378 | | 0.566613 | |  | |  | |  | |  | |  | | |
| CNL-FNG.chr6.2058 | CN-FPE.chr6.453 | | 0.019356 | | 0.036657 | | 0.528035 | |  | |  | |  | |  | |  | | |
| CNL-FNG.chr6.2406 | CNL-FMA.chr6.1508 | | 0.119941 | | 0.388035 | | 0.309098 | |  | |  | |  | |  | |  | | |
| CNL-FNG.chr6.2406 | CN-FNU.ctg84.31 | | 0.124319 | | 0.471947 | | 0.263416 | |  | |  | |  | |  | |  | | |
| CNL-FNG.chr7.204 | CNL-FPE.chr6.1639 | | 0.022847 | | 0.031192 | | 0.732467 | |  | |  | |  | |  | |  | | |
| CNL-FNG.chr7.204 | CNL-FNU.ctg145.8 | | 0.005526 | | 0.024507 | | 0.225476 | |  | |  | |  | |  | |  | | |
| CNL-FNG.chr7.204 | CNL-FvH4.7g02890.1 | | 0.007072 | | 0.032144 | | 0.220009 | |  | |  | |  | |  | |  | | |
| CNL-FNG.chr7.951 | CN-FPE.chr6.3765 | | 0.007049 | | 0.013069 | | 0.539384 | |  | |  | |  | |  | |  | | |
| CNL-FNU.ctg155.369 | CNL-FNG.chr5.2089 | | 0.006164 | | 0.018631 | | 0.330858 | |  | |  | |  | |  | |  | | |
| CNL-FNU.ctg155.369 | CNL-FvH4.5g22710.1 | | 0.006163 | | 0.018641 | | 0.330636 | |  | |  | |  | |  | |  | | |
| orthologous genes | | | | | | paralogous gene | | | | | | | | | | | | | |
| Seq_ 1 | | Seq_2 | | Ka | | Ks | | Ka/Ks | | Seq_ 1 | | Seq_2 | | Ka | | Ks | | Ka/Ks | |
| CNL-FNU.ctg156.8 | CNL-FNG.chr5.2089 | | 0.004927 | | 0.018638 | | 0.26435 | |  | |  | |  | |  | |  | | |
| CNL-FNU.ctg156.8 | CNL-FvH4.5g22710.1 | | 0.004926 | | 0.018647 | | 0.264173 | |  | |  | |  | |  | |  | | |
| CNL-FNU.ctg158.148 | CNL-FII.chr5.2008 | | 0.097827 | | 0.252886 | | 0.386843 | |  | |  | |  | |  | |  | | |
| CNL-FNU.ctg158.148 | CNL-FvH4.5g23470.1 | | 0.096992 | | 0.267719 | | 0.362289 | |  | |  | |  | |  | |  | | |
| CNL-FNU.ctg169.439 | CNL-FPE.chr6.1273 | | 0.021431 | | 0.035255 | | 0.607883 | |  | |  | |  | |  | |  | | |
| CNL-FNU.ctg169.439 | N-FvH4.1g12710.1 | | 0.010243 | | 0.019754 | | 0.518524 | |  | |  | |  | |  | |  | | |
| CNL-FNU.ctg169.439 | CNL-FMA.chr1.1369 | | 0.00872 | | 0.018008 | | 0.484202 | |  | |  | |  | |  | |  | | |
| CNL-FNU.ctg169.439 | CNL-FII.chr1.1071 | | 0.009751 | | 0.025346 | | 0.384714 | |  | |  | |  | |  | |  | | |
| CNL-FNU.ctg169.439 | NL-FNG.chr1.1121 | | 0.013196 | | 0.041759 | | 0.316004 | |  | |  | |  | |  | |  | | |
| CNL-FNU.ctg48.6 | CNL-FNG.chr3.1370 | | 0.010095 | | 0.013637 | | 0.740316 | |  | |  | |  | |  | |  | | |
| CNL-FNU.ctg48.6 | CNL-FII.chr3.1384 | | 0.013712 | | 0.02296 | | 0.597232 | |  | |  | |  | |  | |  | | |
| CNL-FNU.ctg53.185 | CNL-FDA.chr6.13561 | | 0.058206 | | 0.077814 | | 0.748022 | |  | |  | |  | |  | |  | | |
| CNL-FNU.ctg53.289 | CNL-FDA.chr6.13561 | | 0.078861 | | 0.096737 | | 0.815203 | |  | |  | |  | |  | |  | | |
| CNL-FNU.ctg53.85 | CNL-FDA.chr6.13561 | | 0.057622 | | 0.050554 | | 1.139801 | |  | |  | |  | |  | |  | | |
| CNL-FNU.ctg84.614 | CNL-FDA.chr6.13561 | | 0.066622 | | 0.106663 | | 0.624602 | |  | |  | |  | |  | |  | | |
| CNL-FPE.chr1.1624 | CN-FvH4.3g21750.1 | | 0.016105 | | 0.04794 | | 0.335933 | |  | |  | |  | |  | |  | | |
| CNL-FPE.chr1.2047 | N-FNU.ctg161.240 | | 0.027683 | | 0.024973 | | 1.108518 | |  | |  | |  | |  | |  | | |
| CNL-FPE.chr1.871 | CNL-FNU.ctg114.210 | | 0.051803 | | 0.075239 | | 0.688517 | |  | |  | |  | |  | |  | | |
| CNL-FPE.chr2.3627 | CNL-FNU.ctg114.210 | | 0.134407 | | 0.289255 | | 0.464665 | |  | |  | |  | |  | |  | | |
| CNL-FPE.chr3.1482 | CNL-FNU.ctg82.313 | | 0.048286 | | 0.079161 | | 0.609968 | |  | |  | |  | |  | |  | | |
| CNL-FPE.chr3.4782 | CNL-FNU.ctg81.894 | | 0.030584 | | 0.034124 | | 0.896272 | |  | |  | |  | |  | |  | | |
| CNL-FPE.chr3.4782 | NL-FDA.chr4.05233 | | 0.044395 | | 0.057544 | | 0.771511 | |  | |  | |  | |  | |  | | |
| CNL-FPE.chr4.1619 | CNL-FNU.ctg158.40 | | 4.53E-04 | | 0.00304 | | 0.149157 | |  | |  | |  | |  | |  | | |
| CNL-FPE.chr5.124 | CNL-FNU.ctg165.171 | | 0.042521 | | 0.025921 | | 1.640421 | |  | |  | |  | |  | |  | | |
| CNL-FPE.chr5.124 | CN-FvH4.3g16720.1 | | 0.018631 | | 0.023593 | | 0.789682 | |  | |  | |  | |  | |  | | |
| CNL-FPE.chr6.1273 | N-FvH4.1g12710.1 | | 0.017873 | | 0.029475 | | 0.606374 | |  | |  | |  | |  | |  | | |
| CNL-FPE.chr6.1273 | CNL-FNU.ctg170.24 | | 0.009093 | | 0.018794 | | 0.483837 | |  | |  | |  | |  | |  | | |
| CNL-FPE.chr6.1639 | CNL-FNU.ctg145.8 | | 0.018178 | | 0.019338 | | 0.939996 | |  | |  | |  | |  | |  | | |
| CNL-FPE.chr6.1639 | CNL-FvH4.7g02890.1 | | 0.024552 | | 0.033935 | | 0.723498 | |  | |  | |  | |  | |  | | |
| CNL-FPE.chr7.1571 | CNL-FvH4.7g27330.1 | | 0.062446 | | 0.078584 | | 0.794641 | |  | |  | |  | |  | |  | | |
| CNL-FPE.chr7.1798 | CN-FvH4.2g05600.1 | | 0.038212 | | 0.108545 | | 0.352041 | |  | |  | |  | |  | |  | | |
| CNL-FPE.chr7.237 | NL-FvH4.7g00850.1 | | 0.020685 | | 0.095196 | | 0.217286 | |  | |  | |  | |  | |  | | |
| CNL-FPE.chr7.333 | CNL-FNU.ctg114.210 | | 0.109999 | | 0.242508 | | 0.453588 | |  | |  | |  | |  | |  | | |
| CNL-FPE.chr7.335 | CNL-FNU.ctg114.210 | | 0.111352 | | 0.221055 | | 0.503731 | |  | |  | |  | |  | |  | | |
| CNL-FvH4.2g17640.1 | CNL-FDA.chr6.13908 | | 0.055129 | | 0.075133 | | 0.733751 | |  | |  | |  | |  | |  | | |
| CNL-FvH4.4g16700.1 | NL-FNU.ctg84.211 | | 0.017829 | | 0.030678 | | 0.581153 | |  | |  | |  | |  | |  | | |
| CNL-FvH4.4g22940.1 | CNL-FNU.ctg82.648 | | 0.075559 | | 0.123049 | | 0.614056 | |  | |  | |  | |  | |  | | |
| CNL-FvH4.4g26300.1 | NL-FNU.ctg82.264 | | 0.030386 | | 0.030121 | | 1.0088 | |  | |  | |  | |  | |  | | |
| CNL-FvH4.4g29310.1 | NL-FDA.chr4.05233 | | 2.601162 | | 2.475932 | | 1.050579 | |  | |  | |  | |  | |  | | |
| CNL-FvH4.5g22710.1 | CNL-FNU.ctg158.42 | | 0.107043 | | 0.682964 | | 0.156733 | |  | |  | |  | |  | |  | | |
| CNL-FvH4.5g22710.1 | CNL-FII.chr5.3063 | | 0.106433 | | 0.679472 | | 0.156641 | |  | |  | |  | |  | |  | | |
| CNL-FvH4.5g22710.1 | CNL-FDA.chr5.25050 | | 0.104921 | | 0.675433 | | 0.155338 | |  | |  | |  | |  | |  | | |
| orthologous genes | | | | | | paralogous gene | | | | | | | | | | | | | |
| Seq_ 1 | | Seq_2 | | Ka | | Ks | | Ka/Ks | | Seq_ 1 | | Seq_2 | | Ka | | Ks | | Ka/Ks | |
| CNL-FvH4.5g23390.1 | CNL-FNU.ctg158.42 | | 0.146248 | | 0.563935 | | 0.259335 | |  | |  | |  | |  | |  | | |
| CNL-FvH4.5g23390.1 | CNL-FNG.chr5.2146 | | 0.145685 | | 0.573883 | | 0.253859 | |  | |  | |  | |  | |  | | |
| CNL-FvH4.5g23390.1 | CNL-FDA.chr5.25050 | | 0.143993 | | 0.580702 | | 0.247964 | |  | |  | |  | |  | |  | | |
| CNL-FvH4.5g23420.1 | CNL-FNU.ctg158.42 | | 0.00364 | | 0.034466 | | 0.105621 | |  | |  | |  | |  | |  | | |
| CNL-FvH4.5g23450.1 | CNL-FNU.ctg158.45 | | 0.02365 | | 0.048935 | | 0.483289 | |  | |  | |  | |  | |  | | |
| CNL-FvH4.5g23470.1 | CNL-FNU.ctg158.48 | | 0.010009 | | 0.037863 | | 0.264359 | |  | |  | |  | |  | |  | | |
| CNL-FvH4.5g33290.1 | NL-FNU.ctg161.49 | | 0.201166 | | 0.324076 | | 0.620736 | |  | |  | |  | |  | |  | | |
| CNL-FvH4.6g02730.1 | CNL-FNG.chr2.1430 | | 0.090708 | | 0.109099 | | 0.83143 | |  | |  | |  | |  | |  | | |
| CNL-FvH4.6g02730.1 | CNL-FDA.chr6.02198 | | 0.083691 | | 0.101291 | | 0.826243 | |  | |  | |  | |  | |  | | |
| CNL-FvH4.6g02730.1 | CN-FII.chr6.198 | | 0.072252 | | 0.092122 | | 0.78431 | |  | |  | |  | |  | |  | | |
| CNL-FvH4.6g02760.1 | CNL-FDA.chr6.02198 | | 0.075613 | | 0.074463 | | 1.015441 | |  | |  | |  | |  | |  | | |
| CNL-FvH4.6g02760.1 | CNL-FNG.chr2.1430 | | 0.083895 | | 0.100567 | | 0.834214 | |  | |  | |  | |  | |  | | |
| CNL-FvH4.6g02820.1 | CNL-FDA.chr6.02198 | | 0.077222 | | 0.093808 | | 0.823195 | |  | |  | |  | |  | |  | | |
| CNL-FvH4.6g02820.1 | CN-FII.chr6.198 | | 0.079897 | | 0.1147 | | 0.696572 | |  | |  | |  | |  | |  | | |
| CNL-FvH4.6g48220.1 | CN-FNU.ctg53.150 | | 0.036864 | | 0.065675 | | 0.561311 | |  | |  | |  | |  | |  | | |
| CNL-FvH4.6g53580.1 | CNL-FII.chr3.408 | | 3.09675 | | 2.905408 | | 1.065857 | |  | |  | |  | |  | |  | | |
| CNL-FvH4.6g53580.1 | CNL-FPE.chr3.535 | | 3.080185 | | 3.045695 | | 1.011324 | |  | |  | |  | |  | |  | | |
| CNL-FvH4.7g01950.1 | CNL-FNU.ctg146.100 | | 0.049417 | | 0.086951 | | 0.568336 | |  | |  | |  | |  | |  | | |
| CNL-FvH4.7g02890.1 | CNL-FNU.ctg145.8 | | 0.006619 | | 0.029457 | | 0.224687 | |  | |  | |  | |  | |  | | |
| CNL-FvH4.7g22030.1 | CNL-FNU.ctg129.61 | | 0.075279 | | 0.084169 | | 0.894379 | |  | |  | |  | |  | |  | | |
| CNL-FvH4.7g22140.1 | CNL-FNU.ctg129.61 | | 0.076634 | | 0.086483 | | 0.886115 | |  | |  | |  | |  | |  | | |
| CNL-FvH4.7g22140.1 | CNL-FDA.chr7.01250 | | 0.068925 | | 0.079558 | | 0.866347 | |  | |  | |  | |  | |  | | |
| CNL-FvH4.7g26370.1 | CNL-FDA.chr7.01119 | | 0.08421 | | 0.097181 | | 0.866527 | |  | |  | |  | |  | |  | | |
| CNL-FvH4.7g27890.1 | CNL-FPE.chr7.1571 | | 0.104338 | | 0.254871 | | 0.409377 | |  | |  | |  | |  | |  | | |
| CNL-FVI.CHR1.1117 | NL-FNG.chr1.1121 | | 0.010245 | | 0.036387 | | 0.281569 | |  | |  | |  | |  | |  | | |
| CNL-FVI.CHR1.1117 | CNL-FPE.chr6.1273 | | 0.008539 | | 0.038649 | | 0.220941 | |  | |  | |  | |  | |  | | |
| CNL-FVI.CHR1.1117 | CNL-FII.chr1.1071 | | 0.005119 | | 0.034546 | | 0.148173 | |  | |  | |  | |  | |  | | |
| CNL-FVI.CHR1.1117 | N-FvH4.1g12710.1 | | 0.006504 | | 0.05034 | | 0.129203 | |  | |  | |  | |  | |  | | |
| CNL-FVI.CHR1.1117 | CNL-FMA.chr1.1369 | | 0.004093 | | 0.034531 | | 0.118526 | |  | |  | |  | |  | |  | | |
| CNL-FVI.CHR1.1117 | CNL-FNU.ctg170.24 | | 0.003578 | | 0.034588 | | 0.103454 | |  | |  | |  | |  | |  | | |
| CNL-FVI.CHR1.1853 | CNL-FDA.chr6.02010 | | 0.116236 | | 0.237929 | | 0.488532 | |  | |  | |  | |  | |  | | |
| CNL-FVI.CHR1.1874 | CNL-FvH4.1g23030.1 | | 0.07363 | | 0.113235 | | 0.650239 | |  | |  | |  | |  | |  | | |
| CNL-FVI.CHR1.1874 | CNL-FDA.chr6.02010 | | 0.123185 | | 0.289247 | | 0.425881 | |  | |  | |  | |  | |  | | |
| CNL-FVI.CHR1.572 | NL-FDA.chr3.21153 | | 0.03241 | | 0.01609 | | 2.014317 | |  | |  | |  | |  | |  | | |
| CNL-FVI.CHR1.572 | CNL-FNU.ctg165.171 | | 0.021649 | | 0.012586 | | 1.720123 | |  | |  | |  | |  | |  | | |
| CNL-FVI.CHR1.572 | CNL-FPE.chr5.124 | | 0.043854 | | 0.029362 | | 1.493564 | |  | |  | |  | |  | |  | | |
| CNL-FVI.CHR1.572 | CNL-FII.chr3.1385 | | 0.038673 | | 0.027483 | | 1.407151 | |  | |  | |  | |  | |  | | |
| CNL-FVI.CHR1.572 | CNL-FMA.chr3.1344 | | 0.052132 | | 0.049782 | | 1.047195 | |  | |  | |  | |  | |  | | |
| CNL-FVI.CHR1.572 | CNL-FNG.chr1.545 | | 0.028179 | | 0.027593 | | 1.021268 | |  | |  | |  | |  | |  | | |
| CNL-FVI.CHR1.572 | CN-FvH4.3g16720.1 | | 0.062214 | | 0.074869 | | 0.830977 | |  | |  | |  | |  | |  | | |
| CNL-FVI.CHR2.1431 | NL-FII.chr6.2634 | | 0.061398 | | 0.113243 | | 0.542178 | |  | |  | |  | |  | |  | | |
| CNL-FVI.CHR2.1471 | CNL-FPE.chr6.2574 | | 0.055473 | | 0.050879 | | 1.090282 | |  | |  | |  | |  | |  | | |
| orthologous genes | | | | | | paralogous gene | | | | | | | | | | | | | |
| Seq_ 1 | | Seq_2 | | Ka | | Ks | | Ka/Ks | | Seq_ 1 | | Seq_2 | | Ka | | Ks | | Ka/Ks | |
| CNL-FVI.CHR2.2054 | CNL-FII.chr2.1235 | | 0.00724 | | 0.017031 | | 0.425089 | |  | |  | |  | |  | |  | | |
| CNL-FVI.CHR2.2280 | CNL-FDA.chr6.13908 | | 0.052742 | | 0.079843 | | 0.660568 | |  | |  | |  | |  | |  | | |
| CNL-FVI.CHR2.2280 | CNL-FvH4.2g17640.1 | | 0.015628 | | 0.038924 | | 0.401505 | |  | |  | |  | |  | |  | | |
| CNL-FVI.CHR2.3860 | CNL-FDA.chr6.02010 | | 0.063509 | | 0.064431 | | 0.985692 | |  | |  | |  | |  | |  | | |
| CNL-FVI.CHR2.555 | CNL-FDA.chr2.19060 | | 0.013763 | | 0.015763 | | 0.873133 | |  | |  | |  | |  | |  | | |
| CNL-FVI.CHR2.555 | CNL-FNG.chr2.482 | | 0.015859 | | 0.025937 | | 0.611464 | |  | |  | |  | |  | |  | | |
| CNL-FVI.CHR2.555 | CNL-FNU.ctg104.299 | | 0.014873 | | 0.027645 | | 0.538 | |  | |  | |  | |  | |  | | |
| CNL-FVI.CHR2.555 | CN-FII.chr2.551 | | 0.021817 | | 0.043031 | | 0.507021 | |  | |  | |  | |  | |  | | |
| CNL-FVI.CHR2.555 | CN-FPE.chr7.728 | | 0.045885 | | 0.104359 | | 0.439688 | |  | |  | |  | |  | |  | | |
| CNL-FVI.CHR2.555 | CN-FMA.chr2.503 | | 0.012395 | | 0.048248 | | 0.256896 | |  | |  | |  | |  | |  | | |
| CNL-FVI.CHR3.185 | CNL-FNG.chr3.1370 | | 0.017527 | | 0.028737 | | 0.609897 | |  | |  | |  | |  | |  | | |
| CNL-FVI.CHR3.185 | CNL-FII.chr3.1384 | | 0.01866 | | 0.03548 | | 0.525923 | |  | |  | |  | |  | |  | | |
| CNL-FVI.CHR3.185 | CNL-FNU.ctg30.47 | | 0.010253 | | 0.022177 | | 0.462307 | |  | |  | |  | |  | |  | | |
| CNL-FVI.CHR3.618 | CNL-FNG.chr3.2531 | | 0.030187 | | 0.052539 | | 0.574571 | |  | |  | |  | |  | |  | | |
| CNL-FVI.CHR3.618 | CNL-FPE.chr1.1624 | | 0.018068 | | 0.038312 | | 0.471608 | |  | |  | |  | |  | |  | | |
| CNL-FVI.CHR3.618 | CN-FvH4.3g21750.1 | | 0.04094 | | 0.090204 | | 0.453857 | |  | |  | |  | |  | |  | | |
| CNL-FVI.CHR3.618 | CN-FII.chr3.2924 | | 0.038151 | | 0.087859 | | 0.434234 | |  | |  | |  | |  | |  | | |
| CNL-FVI.CHR3.618 | CNL-FMA.chr3.2975 | | 0.014831 | | 0.047369 | | 0.313102 | |  | |  | |  | |  | |  | | |
| CNL-FVI.CHR5.1412 | CNL-FNU.ctg154.468 | | 0.015442 | | 0.044729 | | 0.345232 | |  | |  | |  | |  | |  | | |
| CNL-FVI.CHR5.1617 | CNL-FNG.chr5.1760 | | 0.069091 | | 0.045929 | | 1.504288 | |  | |  | |  | |  | |  | | |
| CNL-FVI.CHR5.1617 | CNL-FMA.chr5.1739 | | 0.067748 | | 0.058665 | | 1.154825 | |  | |  | |  | |  | |  | | |
| CNL-FVI.CHR5.1617 | CNL-FDA.chr5.07881 | | 0.066597 | | 0.06395 | | 1.04139 | |  | |  | |  | |  | |  | | |
| CNL-FVI.CHR5.2975 | N-FMA.chr5.2639 | | 0.024384 | | 0.023764 | | 1.026062 | |  | |  | |  | |  | |  | | |
| CNL-FVI.CHR5.2975 | CNL-FNG.chr5.2999 | | 0.014523 | | 0.031584 | | 0.459824 | |  | |  | |  | |  | |  | | |
| CNL-FVI.CHR5.2975 | CNL-FPE.chr1.2047 | | 0.042977 | | 0.098 | | 0.438542 | |  | |  | |  | |  | |  | | |
| CNL-FVI.CHR5.2975 | CNL-FII.chr5.3003 | | 0.064445 | | 0.188149 | | 0.34252 | |  | |  | |  | |  | |  | | |
| CNL-FVI.CHR5.3016 | CN-FNG.chr5.2692 | | 0.022561 | | 0.026521 | | 0.850688 | |  | |  | |  | |  | |  | | |
| CNL-FVI.CHR5.3016 | CNL-FMA.chr5.2388 | | 0.034433 | | 0.043987 | | 0.782801 | |  | |  | |  | |  | |  | | |
| CNL-FVI.CHR5.3016 | CNL-FDA.chr5.22369 | | 0.025074 | | 0.036974 | | 0.678151 | |  | |  | |  | |  | |  | | |
| CNL-FVI.CHR6.1106 | CNL-FDA.chr6.26146 | | 0.123093 | | 0.280541 | | 0.438771 | |  | |  | |  | |  | |  | | |
| CNL-FVI.CHR6.1768 | CNL-FDA.chr6.26146 | | 0.073577 | | 0.122412 | | 0.601064 | |  | |  | |  | |  | |  | | |
| CNL-FVI.CHR6.3578 | CNL-FPE.chr7.335 | | 0.086076 | | 0.135687 | | 0.634369 | |  | |  | |  | |  | |  | | |
| CNL-FVI.CHR6.3578 | CNL-FNU.ctg114.210 | | 0.124503 | | 0.288327 | | 0.431813 | |  | |  | |  | |  | |  | | |
| CNL-FVI.CHR7.1021 | CNL-FMA.chr7.880 | | 0.006352 | | 0.035465 | | 0.179115 | |  | |  | |  | |  | |  | | |
| CNL-FVI.CHR7.1021 | CNL-FII.chr7.998 | | 0.006349 | | 0.04074 | | 0.155849 | |  | |  | |  | |  | |  | | |
| CNL-FVI.CHR7.1021 | NL-FNU.ctg132.328 | | 0.006212 | | 0.040638 | | 0.152856 | |  | |  | |  | |  | |  | | |
| CNL-FVI.CHR7.1051 | CN-FPE.chr6.3765 | | 0.007929 | | 0.016417 | | 0.482989 | |  | |  | |  | |  | |  | | |
| CNL-FVI.CHR7.1051 | CNL-FII.chr7.1027 | | 0.007933 | | 0.025529 | | 0.310725 | |  | |  | |  | |  | |  | | |
| CNL-FVI.CHR7.1051 | CNL-FNG.chr7.951 | | 0.005277 | | 0.026742 | | 0.197345 | |  | |  | |  | |  | |  | | |
| CNL-FVI.CHR7.1336 | CNL-FPE.chr7.1124 | | 0.050972 | | 0.10783 | | 0.472711 | |  | |  | |  | |  | |  | | |
| CNL-FVI.CHR7.171 | CNL-FNU.ctg146.100 | | 0.025452 | | 0.074614 | | 0.34112 | |  | |  | |  | |  | |  | | |
| CNL-FVI.CHR7.171 | CNL-FII.chr7.162.3 | | 0.022308 | | 0.070346 | | 0.317123 | |  | |  | |  | |  | |  | | |
| orthologous genes | | | | | | paralogous gene | | | | | | | | | | | | | |
| Seq_ 1 | | Seq_2 | | Ka | | Ks | | Ka/Ks | | Seq_ 1 | | Seq_2 | | Ka | | Ks | | Ka/Ks | |
| CNL-FVI.CHR7.171 | CNL-FvH4.7g01950.1 | | 0.012753 | | 0.041255 | | 0.309128 | |  | |  | |  | |  | |  | | |
| CNL-FVI.CHR7.2604 | CN-FvH4.7g31270.1 | | 0.022868 | | 0.039146 | | 0.58418 | |  | |  | |  | |  | |  | | |
| CNL-FVI.CHR7.264 | CNL-FPE.chr6.1639 | | 0.022068 | | 0.043627 | | 0.505824 | |  | |  | |  | |  | |  | | |
| CNL-FVI.CHR7.264 | CNL-FvH4.7g02890.1 | | 0.007525 | | 0.03377 | | 0.222834 | |  | |  | |  | |  | |  | | |
| CNL-FVI.CHR7.264 | CNL-FII.chr7.257 | | 0.007629 | | 0.039465 | | 0.193306 | |  | |  | |  | |  | |  | | |
| CNL-FVI.CHR7.264 | CNL-FNG.chr7.204 | | 0.007026 | | 0.037347 | | 0.18812 | |  | |  | |  | |  | |  | | |
| CNL-FVI.CHR7.264 | CNL-FNU.ctg145.8 | | 0.006275 | | 0.036808 | | 0.170484 | |  | |  | |  | |  | |  | | |
| CNL-FVI.CHR7.2923 | CNL-FDA.chr3.23062 | | 0.027133 | | 0.021523 | | 1.260654 | |  | |  | |  | |  | |  | | |
| CNL-FVI.CHR7.2923 | CNL-FNU.ctg47.220 | | 0.020985 | | 0.021414 | | 0.979951 | |  | |  | |  | |  | |  | | |
| CNL-FVI.CHR7.2923 | CNL-FII.chr3.77 | | 0.023823 | | 0.029556 | | 0.806043 | |  | |  | |  | |  | |  | | |
| CNL-FVI.CHR7.2923 | CNL-FNG.chr3.84 | | 0.023278 | | 0.03139 | | 0.741575 | |  | |  | |  | |  | |  | | |
| CNL-FVI.CHR7.2923 | CNL-FMA.chr3.75 | | 0.019977 | | 0.028664 | | 0.696927 | |  | |  | |  | |  | |  | | |
| CNL-FVI.CHR7.2926 | CNL-FDA.chr3.23062 | | 0.020896 | | 0.025021 | | 0.835141 | |  | |  | |  | |  | |  | | |
| CNL-FVI.CHR7.2926 | CNL-FNG.chr3.84 | | 0.014349 | | 0.026666 | | 0.538117 | |  | |  | |  | |  | |  | | |
| CNL-FVI.CHR7.2926 | CNL-FMA.chr3.75 | | 0.014095 | | 0.02942 | | 0.47909 | |  | |  | |  | |  | |  | | |
| CNL-FVI.CHR7.2926 | CNL-FII.chr3.77 | | 0.015436 | | 0.032321 | | 0.477592 | |  | |  | |  | |  | |  | | |
| CNL-FVI.CHR7.2926 | CNL-FNU.ctg47.220 | | 0.010359 | | 0.023089 | | 0.448656 | |  | |  | |  | |  | |  | | |
| CNL-FVI.CHR7.3757 | CNL-FII.chr3.951 | | 0.10507 | | 0.276383 | | 0.380161 | |  | |  | |  | |  | |  | | |
| CNL-FVI.CHR7.3757 | N-FPE.chr3.1891 | | 0.014601 | | 0.051868 | | 0.281493 | |  | |  | |  | |  | |  | | |
| CNL-FVI.CHR7.3758 | CNL-FDA.chr3.15906 | | 0.042916 | | 0.086647 | | 0.495302 | |  | |  | |  | |  | |  | | |
| CNL-FVI.CHR7.3762 | CNL-FvH4.3g11490.1 | | 0.007995 | | 0.013839 | | 0.577699 | |  | |  | |  | |  | |  | | |
| CNL-FVI.CHR7.810 | CN-FNG.chr7.813 | | 0.021485 | | 0.026606 | | 0.807526 | |  | |  | |  | |  | |  | | |
| CNL-FVI.CHR7.810 | CNL-FII.chr7.789 | | 0.011269 | | 0.026773 | | 0.420907 | |  | |  | |  | |  | |  | | |
| N-FDA.chr1.22180 | RN-FPE.chr6.6309 | | 0.027411 | | 0.022379 | | 1.224854 | |  | |  | |  | |  | |  | | |
| N-FDA.chr1.22180 | RNL-FMA.chr1.1806 | | 0.020868 | | 0.028093 | | 0.742818 | |  | |  | |  | |  | |  | | |
| N-FDA.chr1.22180 | RN-FvH4.1g15140.1 | | 0.020868 | | 0.028093 | | 0.742818 | |  | |  | |  | |  | |  | | |
| N-FDA.chr2.08227 | CNL-FII.chr2.1235 | | 0.03941 | | 0.056455 | | 0.698076 | |  | |  | |  | |  | |  | | |
| N-FDA.chr2.11387 | RNL-FII.chr2.1682 | | 0.020853 | | 0.019673 | | 1.059955 | |  | |  | |  | |  | |  | | |
| N-FDA.chr2.11387 | RNL-FPE.chr5.2061 | | 0.020889 | | 0.039613 | | 0.527327 | |  | |  | |  | |  | |  | | |
| N-FDA.chr2.11387 | RN-FNU.ctg109.400 | | 0.020889 | | 0.039613 | | 0.527327 | |  | |  | |  | |  | |  | | |
| N-FDA.chr2.11387 | RNL-FNG.chr2.1787 | | 0.020889 | | 0.060233 | | 0.346804 | |  | |  | |  | |  | |  | | |
| N-FDA.chr2.11533 | CNL-FNG.chr2.1430 | | 0.530435 | | 0.765779 | | 0.692673 | |  | |  | |  | |  | |  | | |
| N-FDA.chr2.11533 | CN-FII.chr6.198 | | 0.303243 | | 0.662762 | | 0.457544 | |  | |  | |  | |  | |  | | |
| N-FDA.chr2.12211 | CNL-FvH4.2g36810.1 | | 4.949903 | | 2.114716 | | 2.340694 | |  | |  | |  | |  | |  | | |
| N-FDA.chr3.05877 | TNL-FII.chr3.3603 | | 0.070788 | | 0.096389 | | 0.734394 | |  | |  | |  | |  | |  | | |
| N-FDA.chr3.05877 | TNL-FvH4.3g44070.1 | | 0.124661 | | 0.230537 | | 0.54074 | |  | |  | |  | |  | |  | | |
| N-FDA.chr3.05877 | TNL-FNU.ctg3.75 | | 0.120085 | | 0.229852 | | 0.522442 | |  | |  | |  | |  | |  | | |
| N-FDA.chr3.05878 | TNL-FII.chr3.3603 | | 0.070788 | | 0.096389 | | 0.734394 | |  | |  | |  | |  | |  | | |
| N-FDA.chr3.05878 | TNL-FvH4.3g44070.1 | | 0.124661 | | 0.230537 | | 0.54074 | |  | |  | |  | |  | |  | | |
| N-FDA.chr3.05878 | TNL-FNU.ctg3.75 | | 0.120085 | | 0.229852 | | 0.522442 | |  | |  | |  | |  | |  | | |
| N-FDA.chr3.06067 | N-FNG.chr3.3429 | | 0.002233 | | 0.032152 | | 0.069443 | |  | |  | |  | |  | |  | | |
| N-FDA.chr3.06067 | N-FPE.chr6.572 | | 0.002682 | | 0.043278 | | 0.061971 | |  | |  | |  | |  | |  | | |
| orthologous genes | | | | | | paralogous gene | | | | | | | | | | | | | |
| Seq_ 1 | | Seq_2 | | Ka | | Ks | | Ka/Ks | | Seq_ 1 | | Seq_2 | | Ka | | Ks | | Ka/Ks | |
| N-FDA.chr3.06067 | N-FMA.chr3.3384 | | 0.002681 | | 0.06925 | | 0.038717 | |  | |  | |  | |  | |  | | |
| N-FDA.chr3.20666 | TNL-FvH4.3g44000.1 | | 2.441044 | | 2.534583 | | 0.963095 | |  | |  | |  | |  | |  | | |
| N-FDA.chr3.20666 | TNL-FNU.ctg3.75 | | 0.095153 | | 0.198638 | | 0.479025 | |  | |  | |  | |  | |  | | |
| N-FDA.chr3.20666 | TNL-FII.chr3.3626 | | 0.0137 | | 0.037719 | | 0.363221 | |  | |  | |  | |  | |  | | |
| N-FDA.chr3.20808 | TNL-FvH4.3g45380.1 | | 0.074254 | | 0.06915 | | 1.073815 | |  | |  | |  | |  | |  | | |
| N-FDA.chr3.20808 | TN-FII.chr5.2971 | | 0.134089 | | 0.250906 | | 0.534417 | |  | |  | |  | |  | |  | | |
| N-FDA.chr3.20833 | TN-FvH4.3g45630.1 | | 3.331356 | | 1.905007 | | 1.748737 | |  | |  | |  | |  | |  | | |
| N-FDA.chr3.24469 | RN-FNU.ctg52.52 | | 0.118508 | | 0.271739 | | 0.436111 | |  | |  | |  | |  | |  | | |
| N-FDA.chr5.07880 | CNL-FMA.chr5.1739 | | 0.063945 | | 0.024733 | | 2.585429 | |  | |  | |  | |  | |  | | |
| N-FDA.chr5.07880 | CNL-FNG.chr5.1760 | | 0.072186 | | 0.062172 | | 1.161071 | |  | |  | |  | |  | |  | | |
| N-FDA.chr6.17361 | N-FMA.chr6.2173 | | 0.020599 | | 0.014035 | | 1.467609 | |  | |  | |  | |  | |  | | |
| N-FDA.chr6.17361 | N-FvH4.6g30050.1 | | 0.041634 | | 0.043438 | | 0.958471 | |  | |  | |  | |  | |  | | |
| N-FDA.chr6.17361 | CN-FPE.chr6.447 | | 0.444787 | | 0.643266 | | 0.691451 | |  | |  | |  | |  | |  | | |
| N-FDA.chr6.17361 | CN-FII.chr6.2623 | | 0.166468 | | 0.256327 | | 0.649438 | |  | |  | |  | |  | |  | | |
| N-FDA.chr6.17361 | N-FNU.ctg65.16 | | 0.220874 | | 0.405677 | | 0.544458 | |  | |  | |  | |  | |  | | |
| N-FDA.chr7.14603 | TNL-FNU.ctg132.96 | | 0.033154 | | 0.072069 | | 0.460023 | |  | |  | |  | |  | |  | | |
| N-FDA.chr7.14603 | TNL-FPE.chr1.1274 | | 0.199827 | | 0.622618 | | 0.320946 | |  | |  | |  | |  | |  | | |
| N-FII.chr1.1277 | CNL-FNU.ctg172.55 | | 0.056536 | | 0.047617 | | 1.187315 | |  | |  | |  | |  | |  | | |
| N-FII.chr1.1277 | RNL-FNG.chr1.1333 | | 0.049882 | | 0.04219 | | 1.182315 | |  | |  | |  | |  | |  | | |
| N-FII.chr1.1277 | RNL-FDA.chr1.22196 | | 0.053701 | | 0.052573 | | 1.021455 | |  | |  | |  | |  | |  | | |
| N-FII.chr1.1913 | TNL-FMA.chr0.553 | | 0.070174 | | 0.046201 | | 1.518902 | |  | |  | |  | |  | |  | | |
| N-FII.chr1.1913 | TNL-FNU.ctg173.410 | | 0.046352 | | 0.038525 | | 1.203163 | |  | |  | |  | |  | |  | | |
| N-FII.chr1.1913 | TN-FvH4.1g22520.1 | | 0.028619 | | 0.051572 | | 0.554937 | |  | |  | |  | |  | |  | | |
| N-FII.chr1.1913 | TNL-FDA.chr1.07161 | | 0.146362 | | 0.323913 | | 0.451856 | |  | |  | |  | |  | |  | | |
| N-FII.chr2.1082 | N-FPE.chr2.64 | | 0.069331 | | 0.104899 | | 0.660929 | |  | |  | |  | |  | |  | | |
| N-FII.chr2.2318 | TNL-FNG.chr2.2347 | | 0.022868 | | 0.016855 | | 1.356755 | |  | |  | |  | |  | |  | | |
| N-FII.chr2.2318 | TNL-FDA.chr2.20138 | | 0.022868 | | 0.016855 | | 1.356755 | |  | |  | |  | |  | |  | | |
| N-FII.chr2.2318 | TNL-FPE.chr5.1409 | | 0.013606 | | 0.016998 | | 0.800441 | |  | |  | |  | |  | |  | | |
| N-FII.chr2.2318 | TNL-FMA.chr2.2218 | | 0.022868 | | 0.034097 | | 0.670668 | |  | |  | |  | |  | |  | | |
| N-FII.chr2.2318 | TNL-FNU.ctg114.235 | | 0.013616 | | 0.034292 | | 0.397067 | |  | |  | |  | |  | |  | | |
| N-FII.chr2.645 | TNL-FDA.chr3.09474 | | 0.075663 | | 0.150253 | | 0.503571 | |  | |  | |  | |  | |  | | |
| N-FII.chr2.645 | N-FMA.chr2.489 | | 0.067527 | | 0.172305 | | 0.391902 | |  | |  | |  | |  | |  | | |
| N-FII.chr4.1428 | CN-FNU.ctg84.31 | | 0.071413 | | 0.093257 | | 0.765765 | |  | |  | |  | |  | |  | | |
| N-FII.chr5.1933 | CNL-FvH4.5g22710.1 | | 0.004372 | | 0.007687 | | 0.568771 | |  | |  | |  | |  | |  | | |
| N-FII.chr5.1933 | CNL-FNG.chr5.2089 | | 0.001091 | | 0.003829 | | 0.28496 | |  | |  | |  | |  | |  | | |
| N-FII.chr5.1933 | CNL-FNU.ctg158.42 | | 0.156477 | | 0.618246 | | 0.253098 | |  | |  | |  | |  | |  | | |
| N-FII.chr5.1933 | CNL-FDA.chr5.25050 | | 0.153731 | | 0.636108 | | 0.241675 | |  | |  | |  | |  | |  | | |
| N-FII.chr5.2769 | TNL-FvH4.5g32050.1 | | 0.097261 | | 0.226452 | | 0.4295 | |  | |  | |  | |  | |  | | |
| N-FII.chr5.2769 | TNL-FDA.chr5.03707 | | 0.09615 | | 0.231854 | | 0.4147 | |  | |  | |  | |  | |  | | |
[truncated: 83,020 more chars]
