# Supplementary figures and images for: Evolutionary analysis of TIR- and non-TIR-NBS-LRR disease resistance genes in wild strawberries
Source: Front Plant Sci. 2024 Nov 21;15:1452251. doi: 10.3389/fpls.2024.1452251 (PMC11617207; doi:10.3389/fpls.2024.1452251)

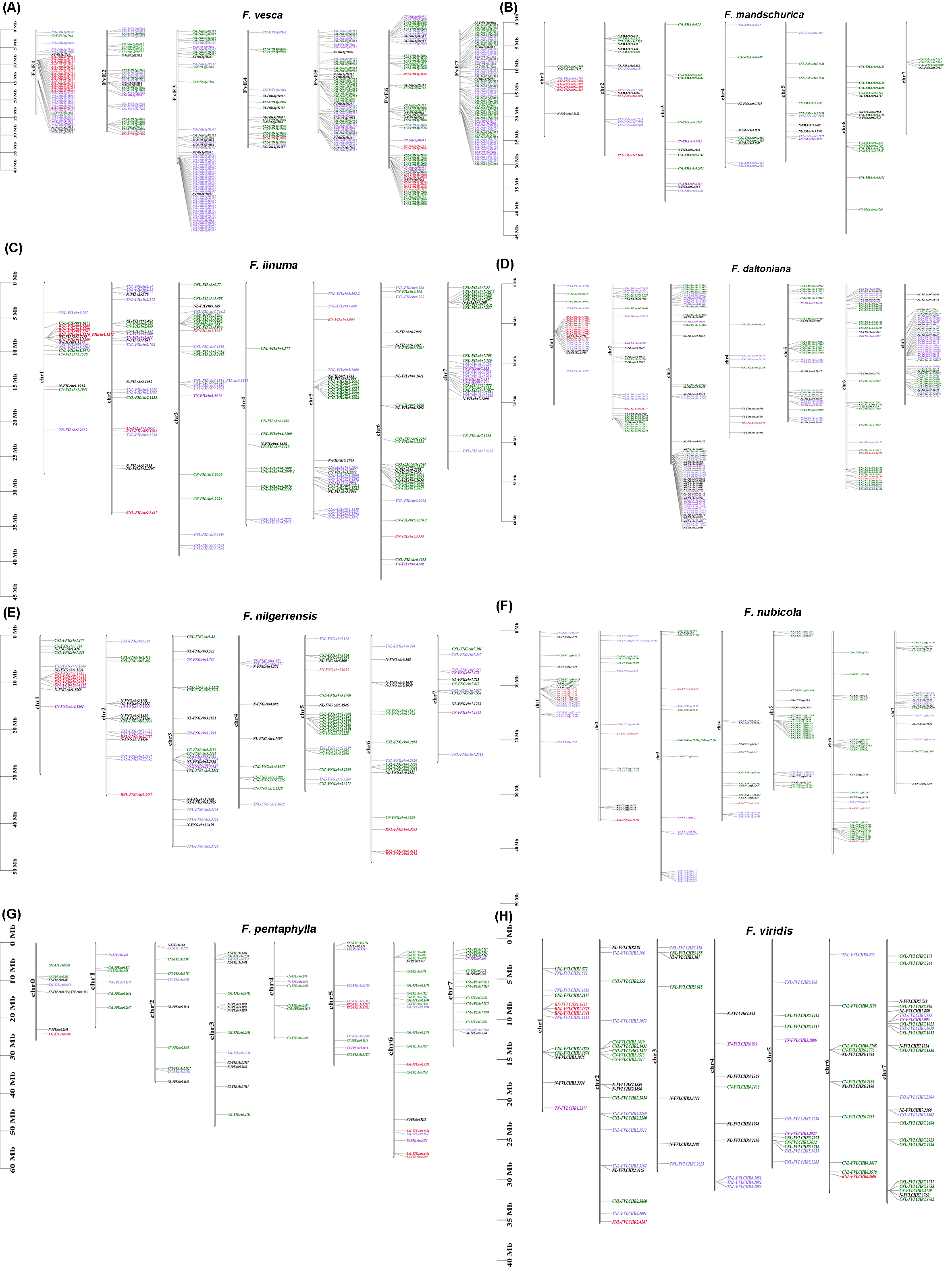

Supplement: Supplementary Figure S1 — Chromosomal mapping analysis of NLR gene family in eight species of strawberries. [file Image1.png]

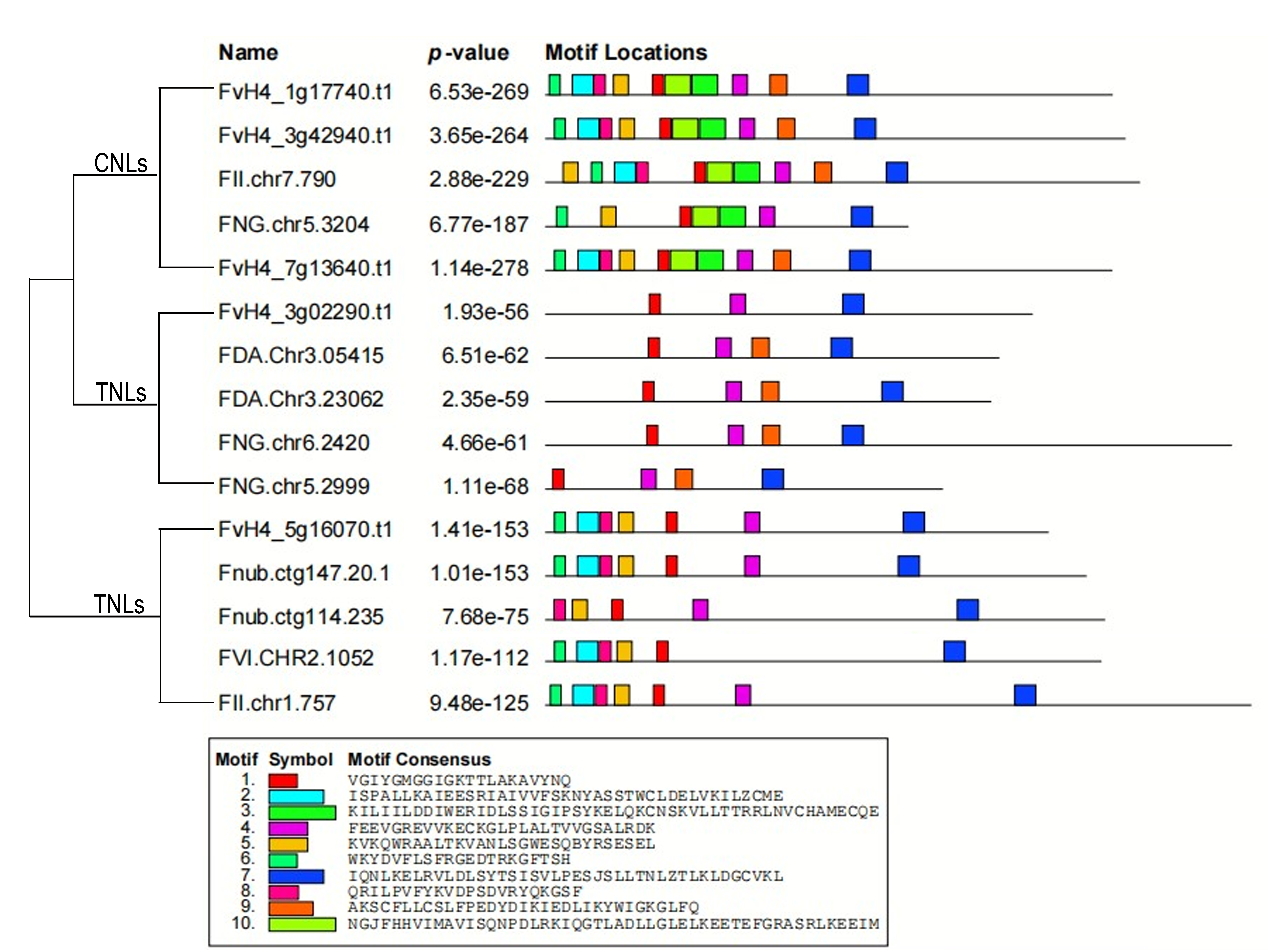

Supplement: Supplementary Figure S2 — Motif analysis of different TNL cluster genes. [file Image2.png]

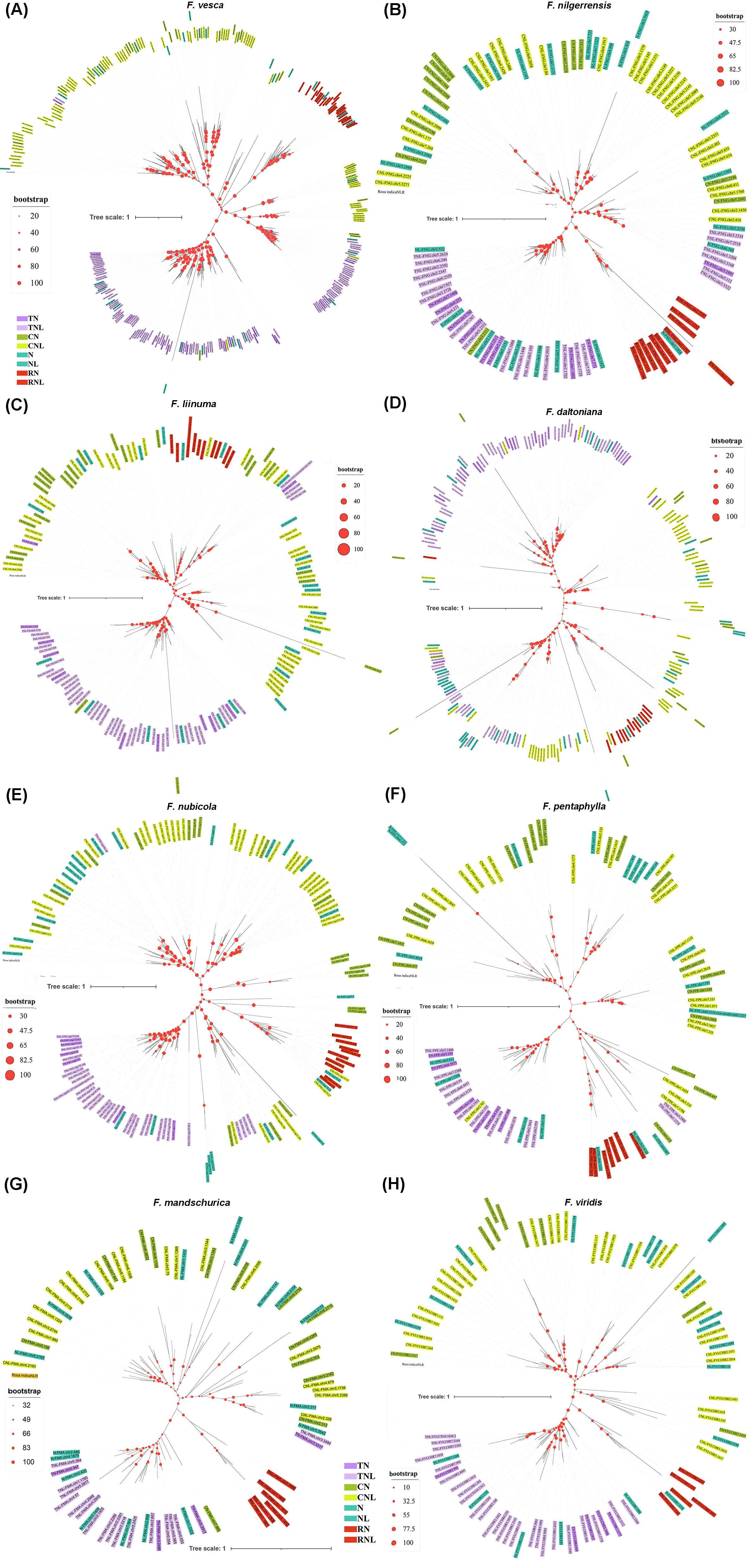

Supplement: Supplementary Figure S3 — Evolutionary tree of the NLR gene family in eight species of strawberries. [file Image3.png]

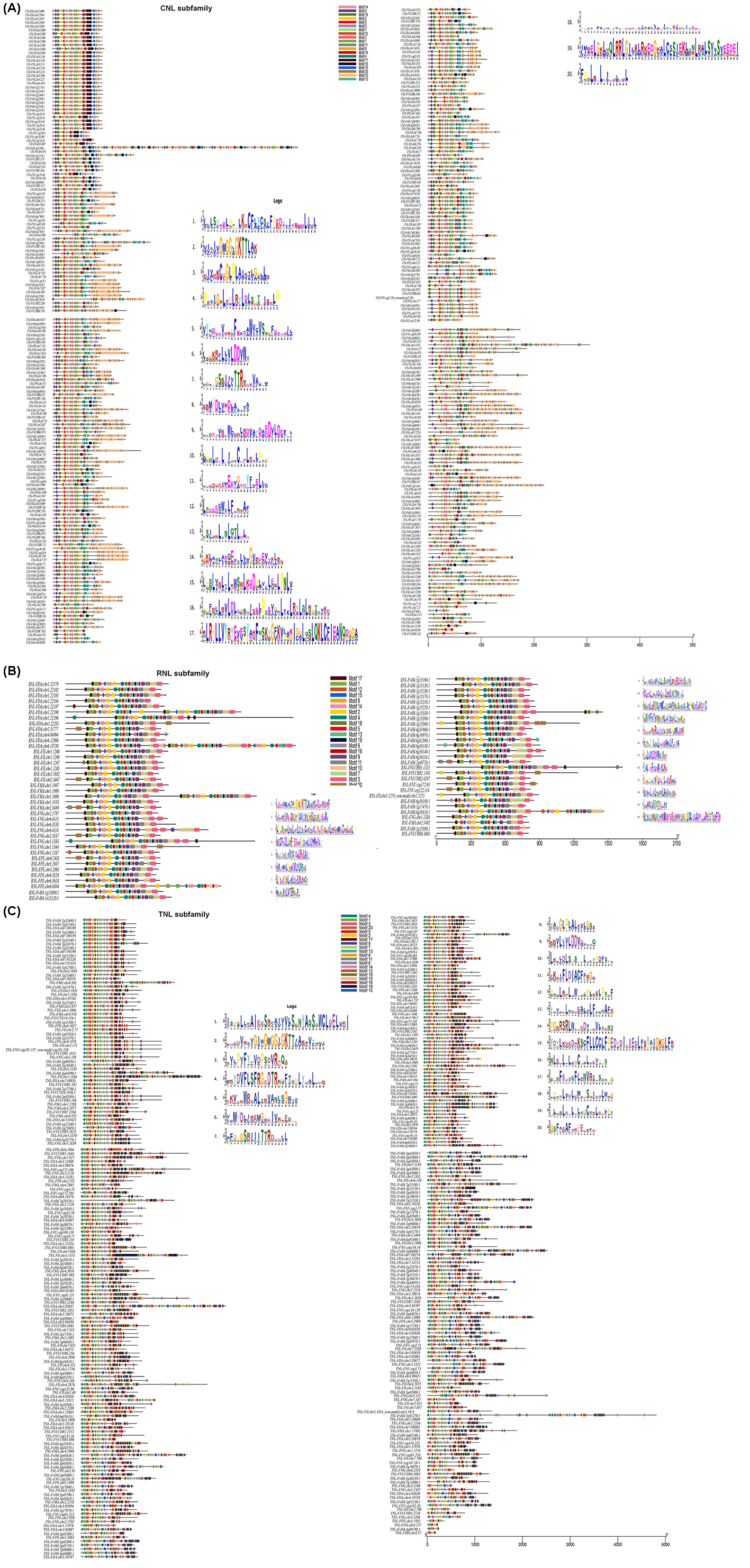

Supplement: Supplementary Figure S4 — Motif distribution map of three subfamily (TNL, CNL, RNL) genes in eight strawberries. [file Image4.png]

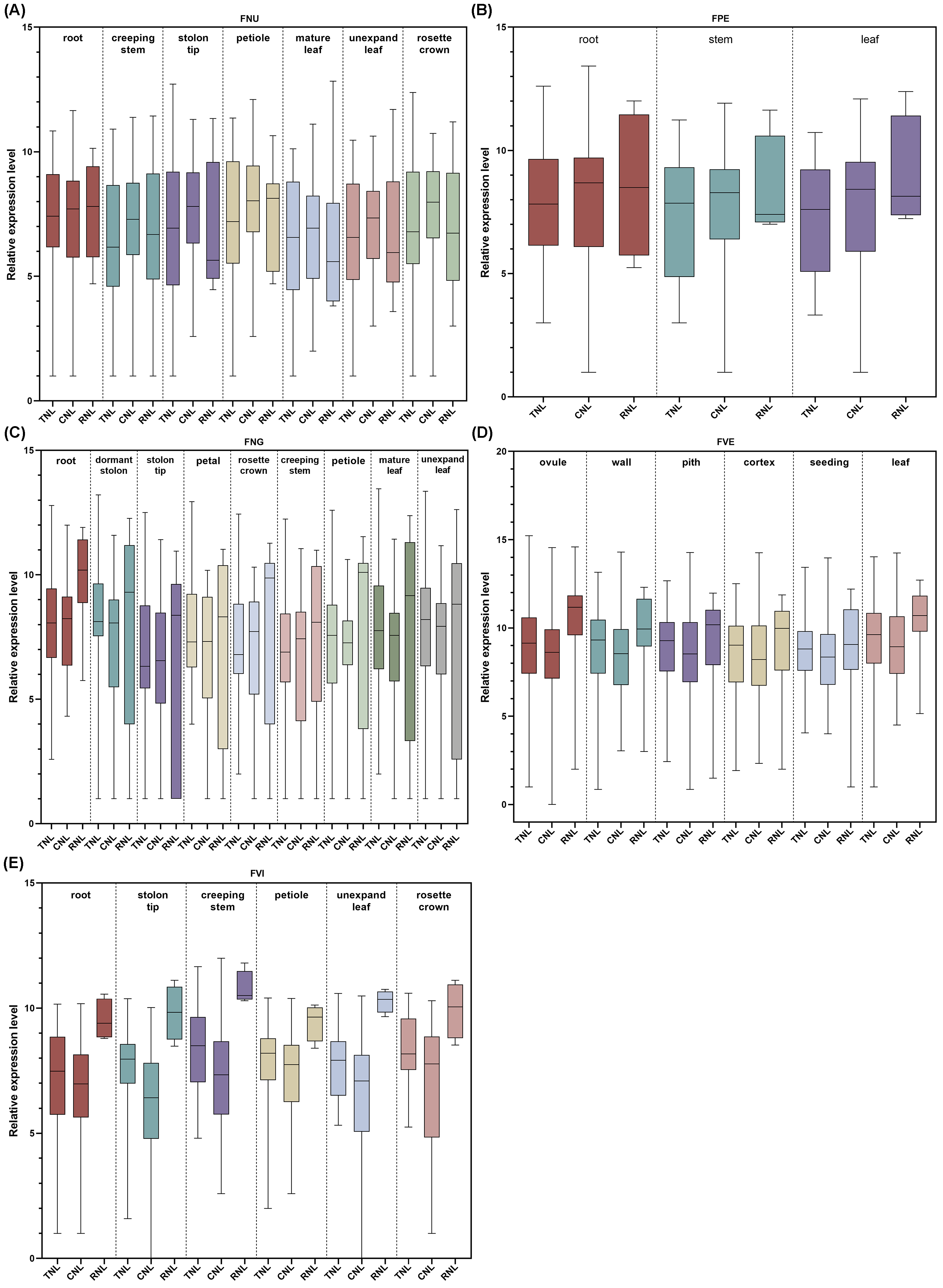

Supplement: Supplementary Figure S5 — Expression levels of NLRs in different parts of five strawberry species under normalized conditions. [file Image5.png]
